# PEPSeek-mediated identification of novel epitopes from viral and bacterial pathogens and the impact on host cell immunopeptidomes

**DOI:** 10.1016/j.mcpro.2025.100937

**Published:** 2025-03-03

**Authors:** John A. Cormican, Lobna Medfai, Magdalena Wawrzyniuk, Martin Pasen, Hassnae Afrache, Constance Fourny, Sahil Khan, Pascal Gneiße, Wai Tuck Soh, Arianna Timelli, Emanuele Nolfi, Yvonne Pannekoek, Andrew Cope, Henning Urlaub, Alice J. A. M. Sijts, Michele Mishto, Juliane Liepe

**Affiliations:** 1 Research group of Quantitative and Systems Biology, Max-Planck-Institute for Multidisciplinary Sciences, 37077 Göttingen, Germany; 2 Göttingen Graduate Center for Neurosciences, Biophysics, and Molecular Biosciences, University of Göttingen, Germany; 3 Department of Biomolecular Health Sciences, Faculty of Veterinary Medicine, Utrecht University, Yalelaan 1, 3584 CL Utrecht, The Netherlands; 4 Centre for Inflammation Biology and Cancer Immunology, King's College London, SE1 1UL London, United Kingdom; 5 Peter Gorer Department of Immunobiology, King's College London, SE1 1UL London, United Kingdom; 6 Research group of Molecular Immunology, Francis Crick Institute, NW1 1AT London, United Kingdom; 7 Georg-August University School of Science (GAUSS), University of Göttingen, Germany; 8 Department of Medical Microbiology and Infection Prevention, Amsterdam UMC location University of Amsterdam, Amsterdam Institute for Infection and Immunity, 1105 AZ Amsterdam, The Netherlands; 9 Centre for Rheumatic Diseases, King's College London, SE1 1UL London, United Kingdom; 10 Research group of Bioanalytical Mass Spectrometry, Max-Planck-Institute for Multidisciplinary Sciences, 37077 Göttingen, Germany; 11 Bioanalytics, Department of Clinical Chemistry, University Medical Center Göttingen, 3705 Göttingen, Germany; 12 Göttingen Center for Molecular Biosciences, University of Göttingen, Germany; 13 Leibniz Institute for Immunotherapy, Franz-Josef-Strauss-Allee 11, 93053 Regensburg, Germany; 14 Facility for Data Sciences and Biostatistics, Max-Planck-Institute for Multidisciplinary Sciences, 37077 Göttingen, Germany

## Abstract

Here, we develop PEPSeek, a web-server based software to allow higher performance in the identification of pathogen-derived epitope candidates detected via mass spectrometry in MHC class I immunopeptidomes. We apply it to human and mouse cell lines infected with either SARS-CoV-2, *Listeria monocytogenes* or *Chlamydia trachomatis*, thereby identifying a large number of novel antigens and epitopes that we prove to be recognized by CD8^+^ T cells. In infected cells, we identified antigenic peptide features that suggested how processing and presentation of pathogenic antigens differ between pathogens. The quantitative tools of PEPSeek also helped to define how *C. trachomatis* infection cycle could impact on the antigenic landscape of the host human cell system, likely reflecting metabolic changes occurred in the infected cells.

## Graphical abstract

**Graphical Abstract:**
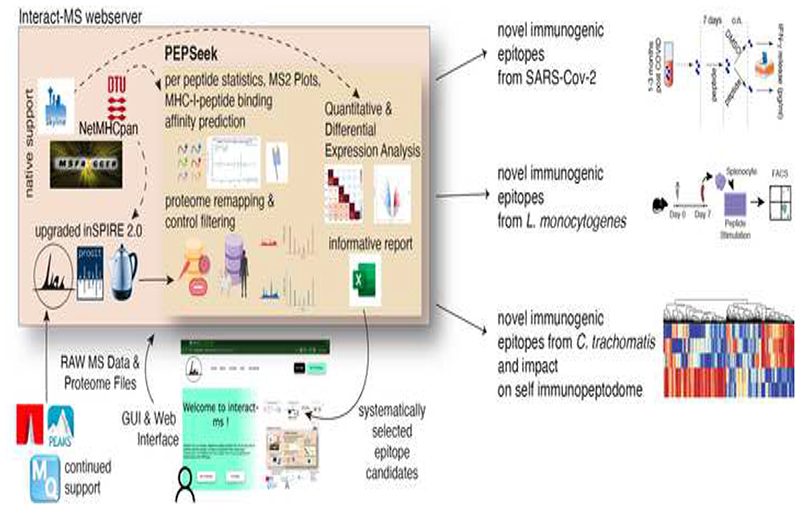

## Introduction

The last years reminded part of the world of what the other part never forgot: infections can be fatal, and the availability of effective vaccines can strongly reduce the impact of epidemics on the population. The development of novel strategies for vaccination capable of stimulating both CD4^+^ and CD8^+^ T cell responses, as well as the systematic prediction and identification of the most immunogenic epitopes for vaccine development are pillars of the research in this field. There is an evident potential impact on the worldwide population when robust investments are provided to both academia and industry on epitope discovery and cognate translational applications (1). For those pathogens that trigger a CD8^+^ T cell response, it is pivotal to determine what pathogen-derived peptides are shown by Major Histocompatibility Complex class I (MHC-I) molecules – a.k.a. MHC-I immunopeptidomes – and are potentially immunogenic. The identification of canonical MHC-I-presented epitopes, *i.e*. antigenic peptides derived from known proteins of a given pathogen able to trigger a CD8^+^ T cell response, has been coupled to novel discovery of noncanonical peptides such as cryptic peptides and post-translationally spliced peptides in recent years (2-4).

Mass spectrometry (MS) has made massive improvements, with thousands of peptides identifiable in MHC-I immunopeptidomes by applying various search engines and computational workflows. In recent years, novel computational tools, relying on spectral prediction and rescoring via Percolator (5), have been developed to provide highly sensitive identification in MHC-I immunopeptidomics (6-11).

While this core technology has obvious potential, identifying epitopes from pathogens within MHC-I immunopeptidomes demands meticulous examination of the results. This process could be optimized by creating a specialized workflow. To this end, we developed an immunopeptidomics-focused workflow named Pathogen Epitope Seeker (PEPSeek) with an interactive web-server to allow ease of access for users without computational experience. Demonstrating its utility, we employed PEPSeek to the MHC-I immunopeptidomes of human and mouse cells infected with the infamous Severe Acute Respiratory Syndrome Coronavirus-2 (SARS-CoV-2) and two intracellular bacteria with different infection pathways, *i.e*., *L. monocytogenes* and *C. trachomatis*. These three pathogens covered diverse forms of infection and activation of CD8^+^ T cells, showcasing the diverse applications of PEPSeek in analyzing MHC-I immunopeptidomes.

SARS-CoV-2, similar to several other coronaviruses, penetrates the host cell upon binding of the spike protein to the cellular entry receptors. The virus expresses and replicates its genomic RNA to produce full-length copies that are incorporated into newly produced viral particles. During this phase, viral proteins can be degraded by proteasomes and the peptide products can be presented via canonical MHC-I antigen processing and presentation pathway (APP) to CD8^+^ T cells (12). A CD8^+^ T cell response against SARS-CoV-2 antigens has been extensively investigated during the last pandemic and is also detectable months after the infection event (13-15).

*L. monocytogenes*, a Gram-positive bacterium, infects the cytosol of intestinal epithelial cells and phagocytes, spreading via the host cell cytoskeleton to neighboring cells. It secretes listeriolysin O (LLO) and phospholipase C (Plc) to enter the cytosol of host cells (16). Bacterial clearance during *L. monocytogenes* infection is mediated by CD8^+^ T cells specific for the secreted bacterial antigens. Pathogen-derived canonical and noncanonical epitopes recognized by CD8^+^ T cells are processed by a standard APP, involving the degradation of cytosolic bacterial antigens by proteasomes, translocation of generated fragments into the endoplasmic reticulum (ER) followed by binding of motif-conforming peptides to host MHC-I molecules that are transported to the cell surface (4). Different *L. monocytogenes* epitopes recognized by CD8^+^ T cells have been described (3, 16-19), and, based on knowledge acquired in Listeria models, *L. monocytogenes*-based vectors have been proposed for various vaccination strategies (16, 19-22).

*C. trachomatis* is an obligate intracellular bacterium that replicates within a vacuole called an inclusion inside epithelial cells. It has a two-phase life cycle, switching between the infectious elementary body (EB) and the non-infectious reticulate body (RB) (23, 24). RBs modify the inclusion by secreting proteins via a secretion machinery designated as the type 3 system (T3SS) into the cytoplasm and inclusion membrane, hijacking host resources for bacterial benefit (25). After replication, RBs revert to EBs, which are dispersed via extrusion of the inclusion from the host cell or cell lysis (23, 24). Although the host immune response usually fails to eliminate *C. trachomatis* infection, vaccine-induced immune memory may protect against this pathogen. Both CD4^+^ T cells and antibodies aid in resistance, whereas the impact of CD8^+^ T cells is less clear (26-28). Nevertheless, both infection and vaccination have been observed to provoke CD8^+^ T cell responses, which controlled infection in experimental settings (29-32).

By applying PEPSeek, we identified many novel antigenic peptides and cognate antigens from these three different pathogens, demonstrated the immunogenicity for several of them and identified features suggesting different APPs. Exploiting the quantitative tools of PEPSeek we also shed light on how infection cycles could impact on which self antigens infected human cells show on their MHC-I molecules.

## Experimental Procedures

### Experimental design and statistical rational

For the analysis of the immunogenicity of epitope candidates in mice infected with *L. monocytogenes*, we applied the following experimental design: mouse infection experiments included one infected and one uninfected mouse group, and were performed twice with n=4 for each mouse group. *Ex vivo* analysis was performed twice, controls in ex vivo analysis of IFN-γ^+^ CD8^+^ T cell specificity included samples stimulated with an irrelevant peptide and with medium only. Means of CD8 T cell responses and s.e.m. were calculated in Graphpad Prism version 9.

For comparison of PSM quality metrics, the Mann-Whitney U test was typically carried out as many of these metrics are not normally distributed.

For the analysis of the immunogenicity of novel SARS-COV-2-derived epitope candidates identified by PEPSeek, we applied the following experimental design: we stimulated PBMCs for 7 days by pulsing them either with pools of synthetic epitope candidates (grouped per antigen), a pool of random peptides not predicted to bind the donor’s MHC-I haplotype, or DMSO (negative control reference) at day 1 of our experiment to expand the peptide-specific T cell clones. On the seventh day, we pulsed the PBMCs again with either each peptide separately (10 μM), a pool of random peptides, or DMSO (negative control reference). IFN-γ secretion was measured in the cell supernatant by IFN-γ enzyme-linked immunosorbent assay (ELISA).

We considered a PBMC response biologically significant (and hence the antigenic peptide as immunogenic epitope) when the IFN-γ concentration of the PBMCs stimulated and re-stimulated with the peptides was higher than the PBMCs stimulated with the peptides and restimulated with DMSO, those stimulated and restimulated with DMSO and those stimulated and restimulated with a pool of random peptides. The PBMCs stimulated and restimulated with the peptides and those stimulated with the peptides and restimulated with DMSO were cultured together during the first 7-day stimulation, therefore the comparison of these two conditions should exclude effects due to potentially peptide-independent activation of PBMCs in a given well during the 7 days.

### Use of inSPIRE 2.0 workflow

The core technology of inSPIRE remains the same as in the original publication (10), which has recently been benchmarked as best performing PSM rescoring tool in tryptic proteomics (33). Target and decoy PSMs are exported from the original search engine with no q-value cut off applied. Prosit is then used to predict MS2 spectra and indexed retention times and the features are generated describing the agreement between predicted and experimental spectra/retention times. Optionally, NetMHCpan predicted binding affinity can also be used as a feature. inSPIRE further improves its feature set with Prosit *delta* features describing the uniqueness of the match between a spectrum and sequence, described in detail in our previous publication (10). All of these features are provided for Percolator rescoring which yields more sensitive peptide identification.

inSPIRE 1.0 supported rescoring of PEAKS, Mascot, and MaxQuant search engine results, with support for MSFragger results was added in inSPIRE 1.5 update (34). In inSPIRE 2.0 we enabled automatic execution of MSFragger via inSPIRE. By default, this search is performed on the user supplied search database as well as the MaxQuant contaminants list.

We did, however, significantly optimize the underlying software, primarily by rewriting inSPIRE 2.0 to rely primarily on the polars library as opposed to the less efficient pandas. We also improved inSPIRE multi-processing making it more efficient to process datasets across multiple cores.

### Evaluation and comparison of posterior error probability (PEP) and q-value cut offs for PEPSeek development

In order to illustrate the importance of the use of posterior error probability and q-value, we showed that the relation between PEP and q-value varies significantly across the datasets used in this study. This variation also existed when the search engine score was the primary metric compared to when upgraded inSPIRE rescoring was applied. This analysis helped to elucidate the impact of dataset composition on the quality of PSMs assigned at 1% FDR. A large fraction of target PSMs achieving a very low PEP value (below 0.001) may allow a small number of PSMs to be assigned at 1% FDR with extremely high PEP values. This issue may be exacerbated in the case of spectral predictor informed rescoring, where the wealth of information gained allows very high confidence in many PSMs.

While the global FDR cut-off is still valuable when investigating the general make up of a proteome or immunopeptidome, we believe that it should be treated with a certain degree of caution when spectral informed rescoring is used. We also note that in the majority of cases when PEPSeek was applied, the PEP cut-off of 0.1 was more stringent than the commonly used 0.01 q-value (*i.e*., 1% FDR) cut off. When seeking a very small number of epitope candidates, which may be expensive or intensive in downstream testing, we believe PEP is the most relevant metric.

### External tools supported within PEPSeek and the Integrated-MS webserver

In order to simplify analysis by PEPSeek we added support for a number of external tools: (i) for raw file conversion, PEPSeek automatically install the ThermoRawFileParser as this tool is available under a fully open-source license; (ii) PEPseek can also execute searches via MSFragger, predict MHC-peptide binding affinity using NetMHCpan, and quantify identifications through Skyline. However, these tools are not available under fully open-source licenses. Hence, the user must first agree to the relevant license agreements before installation. An instructional video for installation of PEPSeek and its dependencies is available online (https://quantsysbio.github.io/interact-ms.html).

### Comparison between the peptide identification by PEPSeek and either the original method or the standard method applied

For the *L. monocytogenes* infected immunopeptidome data, the identifications from the original paper were retrieved from Supplementary Data 1 (sheet name “Immunopeptide ID overview”) from Mayer *et al*. (35). These identified peptides could be matched to PSMs in our search results since PEAKS DB was used in the original study and in our reanalysis. The original peptide identifications were outer joined the final PEPSeek candidates list to identify shared identifications and peptides identified by one tool but not the other. These differences were inspected manually to establish (i) which peptides were excluded by PEPSeek due to presence in control files, (ii) which peptides were excluded by PEPSeek due to a lower confidence in the identification, (iii) which peptides were identified only by PEPSeek.

For the SARS-CoV-2 infected immunopeptidome data from Nagler *et al*. (36) the identified peptides were retrieved from Table S1 of the original publication. The original MaxQuant search results were retrieved from the related Pride archive PXD025499. Specifically, msms.txt files were selected from “Calu-3 infection.zip” and “HEK293T infection.zip”.

For the SARS-CoV-2 infected immunopeptidome data from Weingarten-Gabbay *et al*. (37), the identified peptides were retrieved from the worksheets “A549 + SARS 24h R1” and “HEK293T + SARS 24h R1” of Table S1 of the original publication and filtered based on the species tab to remove HOMO SAPIENS, smORFeomeHuman, and UNREADABLE. The information extracted contained all required details on the PSM level. To note, we excluded the dataset derived from the IHW01070 cell line because the analysis carried out by applying PEPSeek suggested that this dataset was not informative.

For the datasets generated in this study, original identifications were taken as the PEAKS DB identified peptides at 1% FDR with the -10lgP cut off at 1% FDR extracted from the PEAKS Studio GUI manually. The -10lgP cutoffs used for human and mouse cells infected with *C. trachomatis* and *L. monocytogenes* were 20.3 and 20.4 respectively.

Comparisons of PSM quality (spectral angle, spearman correlation, etc.) were not carried out against peptides identified in the original studies and not reidentified by PEPSeek due to the very small number (n = 10) and the fact that multiple of these peptides contained post translational modifications which were not within the Prosit training data.

### MHC-I-peptide binding affinity prediction

MHC-I-peptide binding affinity was predicted by applying NetMHCpan 4.1, executed via the PEPSeek “predictBinding” pipeline. NetMHCpan input files were generated as part of the PEPSeek “prepare” pipeline. When using binding affinity predictions as a validation (*i.e*., comparing number of predicted HLA-I-peptide binders for PEPSeek compared to Prosit-rescoring) we only considered NetMHCpan predictions for peptides with length between 8 to 14 residues due to software limitations. For inSPIRE-affinity, we generated predictions for all peptides possible as null values were not allowed in the Percolator input file.

For our validation and reporting pipelines, we defined a peptide predicted by NetMHCpan to bind a given HLA-I complex, by evaluating against the %Rank value, according to Reynisson *et al*. (38). The %Rank is a transformation on the original prediction, allowing comparison across HLA-I-peptide binding specificities. This system defined a ‘strong MHC-I binder’ as a peptide with a %Rank < 0.5% for a given HLA-I allele, and a ‘HLA-I binder’ as a peptide with a %Rank < 2% for a given MHC-I allele. We, however, used the %Rank based on the binding affinity prediction as opposed to the default %Rank on eluted ligand prediction.

### Peptide quantification with PEPSeek

Identified peptide sequences were quantified and processed using the PEPSeek quantification pipeline which was developed for this study. PSMs identified at 0.01 q-value were written to .ssl format for input to Skyline. In order to quantify peptides which were generated via non-specific cleavage, a .fasta file was generated with one peptide per sequence and Skyline was executed with no *in silico* cleavage of those sequences. Skyline was then executed via docker image from within the PEPSeek “quantify” pipeline. The measured retention time window was 1 minute, the precursor and the precursor with a +H adduct were considered by Skyline, and precursor charges from +1 to +6 were considered.

After Skyline execution, the quantification results were pivoted to give a table of peptide quantification per .raw file. The quantification values were combined with quality control metrics (signal to noise ratio and isotope dot product of the quantification) and a flag indicating from which .raw files the peptide was identified by PEPSeek. These results were then filtered to retain only measurements with an isotope dot product greater than 0.5 and a ratio of signal to background of greater than 4. Normalization was applied by equalizing medians across raw files after a log_2_ transformation had been applied.

### Antigen overrepresentation and network analysis

String version 12.0 (https://string-db.org/) was used to test for over-presentation of *C. trachomatis* and *L. monocytogenes* derived antigens detected by PEPSeek. Organism was set to ‘Chlamydia trachomatis’ and ‘Listeria monocytogenes EGD-e’, respectively. Functional enrichments were filtered for 1% false discovery rate (FDR). Lollipop plots were generated in *R*. Full String networks were exported as .svg files and further annotated in Adobe Illustrator.

### Limitations and caveats of PEPSeek and inSPIRE software

While we believe that the method presented has great potential as the algorithm of choice for analyzing infected and uninfected MHC-I immunopeptidome, it is important to also document the limitations of the software. These can primarily be split into two categories; known limitations which are not supported by PEPSeek/inSPIRE and applications which are theoretically supported but have not yet been investigated.

One limitation which was relevant in this application was current lack of support for TMT-labelled samples. Hence, only unlabeled samples from Mayer *et al*. were investigated. Although it has been recently published a Prosit model that predicts spectra for TMT labelled samples (39), that model was not publicly released when we developed our software. More generally, PEPSeek/inSPIRE is relatively limited for investigating PTMs, as it only supports variable oxidation of methionine and forces carbamidomethylation of cysteine due to the constraints of Prosit. The requirement for carbamidomethylation of cysteine can be an issue as immunopeptidome samples are not commonly alkylated unlike proteomics samples. Though even without this issue cysteines are typically underrepresented in the immunopeptidome (40).

Other applications which we have not explored in this study include the application of PEPSeek to MHC-II samples and analysis of MS data from Bruker generated data compared to ThermoFisher. Theoretically PEPSeek could be applied in both cases but we did not find a need in this study. While our original release of inSPIRE compared favorably to the performance of rescoring tools available at the time, there have been recent releases such as Oktoberfest (9) and MSBooster (11). In the future it may be important to reevaluate the various rescoring tools available across varying applications, including MHC-I and MHC-II immunopeptidomics and using different MS instruments.

### Qualitative peptide sequence motif analysis

In order to map peptides detected by PEPSeek to the HLA-I allele they most likely corresponded to, we evaluated all 9mer peptides derived from *C. trachomatis* and *L. monocytogenes* as well as those from the self MHC-I immunopeptidomes, identified by PEPSeek. We added 1,000 randomly sampled peptides derived from detected self-antigens, which were predicted to bind to one of the HeLa cells' three MHC-I alleles—namely HLA-A*68:02, -B*15:03, and -C*12:03. IC_50_ prediction was performed using NetMHCpan with IC_50_ cut-offs of 500 nM for HLA-A*68:02, -B*15:03, and -C*12:03. Furthermore, we included 500 sampled peptides from *C. trachomatis* and *L. monocytogenes* predicted to bind these MHC-I alleles. After one-hot-encoding of all detected and predicted sequences, we performed UMAP using the *R* package *uwot* version 0.1.16 (UMAP parameters: n_neighbors = 7, metric ="euclidean", negative_sample_rate = 10, repulsion_strength = 1, min_dist = 0.3, n_threads = 6, ret_model = T, verbose = T, init = "agspectral", spread = 0.8), followed by k-means clustering (k = 7; using *kmean* function from *R* package *stats*). Resulting clusters were grouped into three distinct clusters that resembled sequence motifs that are characteristic of the HLA-B*15:03, -A*68:02, and -C*12:03 alleles. Sequence motifs and difference motifs were computed in *R* using the package ‘*DiffLogo*’ version 2.18.0. An alternative to the UMAP approach for binding motif deconvolution is assignment by lowest predicted NetMHCpan binding affinity or by alternative clustering methods, such as Gibbs Clustering (41) or learning mixture models via maximum likelihood estimation (42). We here chose the UMAP approach to allow visualization of similarity and divergence of peptide sequences derived from pathogens compare to host peptides.

### Quantitative peptide sequence motif analysis

The heatmap of normalized MS1 intensities of self-peptides derived from HeLa cells infected with *C. trachomatis* and *L. monocytogenes* pre and post infection was computed for all self-peptides detected and quantified across both datasets. Median normalized peptide MS1 intensities were further scaled by the maximum detected MS1 intensity per peptide across all datasets. Heatmaps were generated in *R* using the *heatmap* function from the *stats* package (parameters: scale='none', Colv=NA).

Median normalized peptide MS1 intensities were used to compute abundance fold changes between infected and control samples. Peptides were grouped into 7 clusters according to their abundance fold change upon infection as illustrated in the violin plots in the cognate figure. For *C. trachomatis*, the 7 clusters were defined by performing separate KMeans clusterings (random seed: 42) on the abundance log2 fold change (log2fc) of 24h post infection and control samples, and 48h post infection and control samples. The clusters were matched on centroid value and only peptides belonging to both matched clusters were used for further analysis.

For *L. monocytogenes*, the 7 clusters were defined by performing a KMeans clustering on the abundance log2 fold change of infected and control samples.

For each group peptides were aligned at their C-terminus and difference motifs between each group and group 4 (*i.e*., peptides that do not change their abundance upon infection) were computed using the *R* package ‘*DiffLogo*’ version 2.18.0. and are displayed for the C-terminal amino acids. A fisher’s exact test was used to determine significant differences of amino acid frequencies at the C-term between each group and group 4. The resulting p-values were adjusted using the Benjamini-Hochberg procedure and amino acids with p-values < 0.05 were plotted as opaque, amino acids with p-values > 0.05 were plotted as transparent.

For each group peptides were aligned at their C-terminus and difference motifs between each group and group 4 (*i.e*., peptides that do not change their abundance upon infection) were computed using the *R* package ‘*DiffLogo*’ version 2.18.0. and are displayed for the C-terminal amino acids.

### Quantitative antigen gene set enrichment analysis

To access antigen presentability, we aggregated self-antigen peptide abundance information to protein level. Antigen presentability was defined as the summed log_2_ fold changes of all detected peptides derived from the same antigen, based on median normalised peptide’s MS1 intensities. Antigens were mapped to their corresponding genes. The resulting antigen presentability rates were subjected to gene set enrichment analysis using the *fgsea* function from the *R* package ‘*fgsea*’ version 1.20.0. Employed gene sets were exported from *String* version 12.0 (https://string-db.org/; all human gene sets). Enriched terms were first collapsed into most significant terms, filtered for p-value < 0.01 and then compared across datasets.

### Synthetic peptides

Peptides for synthesis were initially screened to predict the success of Fmoc-based peptide synthesis (43). For 5 out of 23 epitope candidates from *L. monocytogenes*, the synthesis was unsuccessful and therefore these candidates could not be further tested. The *L. monocytogenes* synthetic peptide library was prepared by pooling all peptides together, with each peptide at 1 µM concentration in a buffer containing 2% ACN and 0.05% TCA. For the SARS-CoV-2 peptides, each synthetic peptide was resuspended in DMSO and series of dilutions were performed in buffer containing 2% ACN and 0.05% TFA.

### MS measurements

MS data of MHC-I immunopeptidomes were collected using either Orbitrap Fusion or Orbitrap Fusion Lumos mass spectrometer coupled to an Ultimate 3000 RSLCnano System (both from ThermoFisherScientific). In detail, each immunopeptidome sample was resuspended with 30 µL of 1% ACN and 0.1% TFA and subsequently sonicated at room temperature for 1 min. The sample was then spun at 13,000 rpm for 1 min. The pH of the sample was checked with a pH indicator to ensure the sample solution was acidic. The supernatant was then transferred into a HPLC sample glass vial. The glass vial was then spun at 5,000 rpm for 1 min before being loaded into the HPLC autosampler. The sample was loaded and separated by a nanoflow HPLC (Ultimate 3000 RSLC) on an Easy-spray C18 nano column (30 cm length, 75 μm internal diameter). Peptides were eluted with a linear gradient of 5% – 45% buffer B (80% ACN, 0.08% formic acid) at a flow rate of 300 nl/min over 90 minutes at 50°C. The instrument was programmed within Xcalibur 4.4 (Obitrap Fusion) or 4.5 (Obitrap Fusion Lumos) to acquire MS data in a Data Dependent Acquisition mode using a method by defining 3s cycle time between a full MS scan and MS/MS fragmentation. We acquired one full-scan MS spectrum at a resolution of 120,000 with a normalized automatic gain control (AGC) target value of 250% and a scan range of 350-1,550 m/z. The MS/MS fragmentation was conducted using HCD collision energy (30%) with an orbitrap resolution of 30,000. The normalized AGC target value was set up at 200% with a max injection time of 120 ms. A dynamic exclusion of 30s and 1 - 4 included charged states were defined within this method.

The *L. monocytogenes* synthetic peptide library measurements were acquired using Orbitrap Fusion Lumos mass spectrometer using the same method described above and a peptide concentration of 10 pmol and 1 pmol.

MS data of SARS-CoV-2 synthetic peptides at 1 pmol each were collected using Orbitrap Fusion Lumos mass spectrometer coupled to an Ultimate 3000 RSLCnano System. The sample was loaded and separated by a nanoflow HPLC (Ultimate 3000 RSLC) on an Easy-spray C18 nano column (30 cm length, 75 μm internal diameter). Single peptides were eluted with a linear gradient of 5% – 55% buffer B (80% ACN, 0.08% formic acid) at a flow rate of 300 nl/min over 35 minutes at 50°C, to also allow a quality check of the peptide purity. The instrument was programmed within Xcalibur 4.5 to acquire MS data in a Data Dependent Acquisition mode using Top 30 precursor ion. We acquired one full-scan MS spectrum at a resolution of 120,000 with a normalized automatic gain control (AGC) target value of 250% and a scan range of 350-1,600 m/z. The MS/MS fragmentation was conducted using HCD collision energy (35%) with an orbitrap resolution of 30,000. The normalized AGC target value was set up at 200% with a dynamic maximum injection time mode. A dynamic exclusion of 30s and 1 - 7 included charged states were defined within this method.

### MS software settings

For all MSFragger searches discussed in this text, RAW MS files were searched using MSFragger version 3.7, although a preliminary analysis had also been performed with MSFragger 3.6 (the results of which are also available in the online repository). Furthermore, these searches were performed without rescoring with MSBooster which is also available in FragPipe. For all PEAKS DB searches, RAW MS files were searched using PEAKS DB, version 10.6.

Precursor mass tolerance was set to 10 ppm for all the public datasets analyzed and 5 ppm for all of the newly generated datasets in this study. Minimum peptide length was set to 7, and maximum peptide length was set to 30. The mass tolerance for the fragment ions was set to 0.02 Da in all cases except for the datasets from Nagler *et al*. which used 0.05 Da. The upgraded inSPIRE rescoring was performed on the pepXML files from MSFragger considering the top 10 hits, using only the basic feature provided for any baseline comparison and with the full upgraded inSPIRE feature set for all other identifications. Oxidation of methionine was set as the only variable PTM, and carbamidomethylation of cysteine was set as a fixed modification for all MSFragger searches. For the newly generated datasets oxidation of methionine, carbamidomethylation of cysteine, N-terminal acetylation, and deamidation of asparagine/glutamine all set as variable modifications (modifications not recognized by Prosit were filtered within the upgraded inSPIRE). Results were exported for all PSMs with PEAKS DB −10lgP score greater than 0.

The number of proteins in search database was 21,246 (20,351 host, 895 pathogen) for the novel dataset of HeLa cells infected with *C. trachomatis* and 28,063 (25,216 host, 2847 pathogen) for the novel dataset of murine cells infected with *L. monocytogenes*. For previously published datasets, the fasta file reused from Mayer *et al*. (HeLa and HCT cells infected with *L. monocytogenes*), Nagler *et al*. (HEK293 and Calu3 cells infected with SARS-CoV-2) and Weingarten-Gabbay *et al*. (HEK293 and A549 cells infected with SARS-CoV-2) contained 23,198 (20,351 host, 2,847 pathogen), 91,541 (91,478 host, 63 pathogen), and 42,311 (42,259 host, 52 pathogen) entries respectively. For the novel datasets these databases were downloaded from uniprot and for the previously published datasets the fasta files were taken from the same repository as the .raw files.

For benchmarking PEAKS DB, MSFragger and MaxQuant search engines, the same settings were used but with carbamidomethylation as a fixed modification and oxidation as a variable modification to match Prosit requirements and our previous benchmarking approach for inSPIRE (10). The identifications from all other tools were simply extracted from the previous results with all settings described previously.

In order to provide the experimental spectra to the upgraded inSPIRE pipeline, RAW files were converted to mgf using ThermoRawFileParser, version 1.4.0 (44)

Percolator, version 3.05.0 was used for all rescoring via the upgraded inSPIRE (5, 45).

### Literature analysis for the identification of novel antigenic peptides and antigens

To determine whether an epitope candidate identified by PEPSeek in MHC-I immunopeptidomes was known in literature as antigenic peptide, the requirement was a positive identification in MHC-I immunopeptidomes and/or a peptide-specific T cell recognition by T cell assays. Positive functional B cell assays and MHC-I-synthetic peptide binding affinity assays were not considered as proof of antigenicity, *i.e*. proof of being presented at the cell surface by MHC-I complexes. The core of the literature search for the SARS-CoV-2-derived epitope candidates and antigens was the IEDB database by selecting In epitope tab -> selected ‘linear peptide’, in assay tab -> selected ’T cell assays’ only, in epitope source tab -> selected ‘SARS-CoV-2 (ID:2697049, SARS2)’ as the organism, in MHC restriction tab -> selection ‘Class I’, in host tab -> selected ‘human’, in disease tab -> selected ‘infectious’. In addition, we used the database of SARS-CoV-2-derived peptides identified in the other experiments performed by Nagler *et al*. and Weingarten-Gabbay *et al*. (36, 37).

The core of the literature search for the *L. monocytogenes*- and *C. trachomatis*-derived epitope candidates and antigens was the IEDB database by selecting: *Listeria* (ID 1637) or *C. trachomatis* (ID 813) as pathogen, MHC-I restriction, and then either T cell assay to search for described epitopes, or MHC ligand assay with filter on ligand elution/mass spectrometry to search for ligands previously identified in MHC-I immunopeptidomes. In addition, the data reported in Mayer *et al*. (35) were searched.

### Cell lines and infections

For the SARS-CoV-2-infected human MHC-I immunopeptidome datasets, the description of the human HEK293T, Calu-3, IHW01070 and A549 cells and how they were infected with SARS-CoV-2 was reported in the original papers (36, 37).

For the *L. monocytogenes-*infected human MHC-I immunopeptidome dataset, the description of the human HeLa and HCT116 cells and how they were infected with *L. monocytogenes* was reported in the original paper (35).

The mouse-derived macrophage cell line Ana-1 was maintained in complete RMPI 1640 (Life technologies) containing 10% Fetal Bovine Serum (FBS) without antibiotics. *L. monocytogenes*, strain 10403S, were grown in Brain-Heart Infusion broth (Sigma Aldrich) and harvested at early log phase for infection. 1 ml of listeria at OD600 = 0.1 per 10 cm dish [or scaled appropriately] as allowed to infect Ana-1 cell line for 30 min. Cell culture medium was then replaced with gentamycin-containing RPMI (10% FBS) to prevent growth of extracellular listeria and infection continued for another 6 h. Cells were then harvested in and washed with ice-cold PBS.

For the *C. trachomatis*-infected human MHC-I immunopeptidome dataset, the human epithelial cell line HeLa was maintained in complete RMPI 1640 (Life technologies) containing 5% Fetal Bovine serum (FBS) and 10 ug/ml of Gentamicin (Thermo fisher). Prior to infection, medium was replaced with complete RPMI supplemented with 1% HEPES (Life technologies), 1% non-essential amino acids (NEAA) (Thermo Fisher), 1% L-Glutamin (Thermo Fisher) and 1% sodium pyruvate (Thermo Fisher). Cells were infected by centrifugation with *C. trachomatis* serovar D/UW-3/CX at a multiplicity of infection of 4, following the protocol described in (46) for 24 h or 48 h then harvested by scraping.

### MHC-I immunopeptidome elution

For the SARS-CoV-2 infected human cells, the MHC-I immunopeptidome elution protocol is described in (36, 37). For *L. monocytogenes* infected human cells, the MHC-I immunopeptidome elution protocol is described in (35).

For *L. monocytogenes* infected mouse-derived macrophage cell line Ana-1, H2-Kb-bound peptides were isolated from 0.48 10^9^ ANA-1 murine cell line using anti-H2-Kb antibody (Y3 clone, Biocell), using the protocol described above. Briefly, MHC-I-bound peptides were isolated from the respective cells after cell lysis for 1h on ice in lysis buffer (PBS, 0.25% Sodium Deoxycholate, 0.2 mM Iodacetamide, 1 mM EDTA, 1:200 protease inhibitor cocktail, 1 mM PMSF and 1% Octyl β-D-glucopyranoside). Cell lysate was pre-cleared by centrifugation for 30 min at 4600 rpm at 4°C. Soluble lysate was loaded at 4°C on Protein-A Sepharose 4B beads (Sigma) crosslinked with the respective antibody in BioRAD^®^ Poly-Prep^®^ chromatography columns. Flow through was loaded four times. Beads were then washed three times with low salt washing buffer (0.15 M NaCl and 0.02 M Tris [pH 8]), three times with high salt washing buffer (0.4 M NaCl and 0.02 M Tris [pH 8]), three times with low salt washing buffer then with 2 mL of 0.1 M Tris (pH 8). MHC-I bound peptides were eluted with 400 µL of 1% trifluoracetic acid (TFA). C18 Sep-Vac 1cc tC18 cartridge hydrophobicity chromatography column (Waters) was used to elute the peptides from the MHC-I molecules. The column was washed with 1 mL of 80% of acetonitrile (ACN)/0.1 % TFA and equilibrated with 2 × 1mL of 0.1% TFA. Sample was loaded on the C18 column and two washes with 1 mL of 0.1%TFA were performed. Peptides were eluted with 250 µL of 30% ACN/0.1% TFA and dried using speed vacuum concentrator. 30 µL of 1% ACN/0.1% TFA was used to resuspend the peptides for MS measurement.

For *C. trachomatis*-infected HeLa, MHC-I-bound peptides were isolated from 5.10^8^ uninfected, 24h and 48h *C. trachomatis*-infected cells using pan-HLA class I antibody, W6/32 (produced in house). Briefly, cells were lysed using PierceIP lysis buffer (Thermofisher Scientific) containing EDTA-free Protease Inhibitor Cocktail (Sigma Aldrich). After 1h of centrifugation at 3000rpm at 4°C, the supernatants were incubated in succession with three different CNBr-activated and tris-blocked Sepharose 4B beads (Sigma Aldrich), which were non-Ig coupled, coupled with normal mouse Ig, and coupled with W6/32 antibodies, respectively. Beads were then loaded into Econo-Column® Chromatography Columns which were pre-washed with 1mM HCL [pH 4], milliQ and with cold PiereIP Lysis buffer (without protease inhibitors). Beads-loaded columns were then washed with 5 bed volumes (b.v) of Pierce IP lysis buffer, 4 b.v of low salt washing buffer (0.12 M NaCl 0.02 M Tris, [pH 8]), 8 b.v of high salt washing buffer (1 M NaCl 0.02 M Tris, [pH 8]), 4 b.v of low salt washing buffer (0.02 M Tris, [pH 8]) and finally with 4 b.v of low salt washing buffer (0.01 M Tris, [pH 8]). Finally, MHC-I-peptide complexes were eluted with 3 b.v of 10% acetic acid and peptides were collected by passage through Amicon Ultra-4 centrifugal filter devices (10 kD). Filtrates were concentrated using vacuum centrifugation.

### Human PBMC stimulation, IFN-γ quantification, peptide selection and synthesis

PBMCs were isolated from fresh peripheral blood by applying a standard Ficoll protocol. Frozen PBMCs were thawed in complete RPMI medium [CM; RPMI+L-Glutamine (Gibco), 25mM Hepes, 1% non-essential amino-acids (GIBCO), 100 U/mL penicillin, 100 mg/mL streptomycin (GIBCO), 1 mM sodium pyruvate (GIBCO) and 5% human serum (Merck)]. Cells were plated at 150*10^3^ cells per well in 96 well plates in 200 µL of CM supplemented with IL-2 (20 U/mL), IL-7 (40 U/mL) and IL-15 (10 U/mL). Depending on the number of PBMCs, several wells for the same condition were prepared. Either synthetic peptide pools at final concentration of 1 µM per peptide or DMSO were added to the PBMC culture for the stimulation. Cells were incubated for seven days at 37 °C, 5% CO_2_. Every two days, half of the medium was replaced with CM supplemented with IL-2 (40 U/mL), IL-7 (80 U/mL), and IL-15 (20 U/mL). On day seven, cells were counted and plated at 40*10^3^ cells in a new 96 well plate in 200 µL of CM without cytokines. Either individual synthetic epitope candidates or the pool of random peptides at 10 µM or DMSO were added to the cell culture. For each restimulation condition at least 2 wells (technical replicates) were prepared. Cells were incubated overnight at 37 °C, 5% CO_2_. Supernatants were harvested for analysis of IFN-γ secretion using a human IFN-γ-ELISA kit (BD Bioscience) as instructed.

Peptides were selected among the SARS-CoV-2-derived epitope candidates identified only by PEPSeek in the MHC-I immunopeptidomes of infected human cell lines, also considering the prediction of a successful peptide synthesis (43). The selection of 8 out of 14 epitope candidates not proved to be antigenic so far was driven by the objective to have peptides that could be identified by the latest PEPSeek version and a previous one that used an older MSFragger version (3.6) and had taken into account only y- and b-ions during the search (the default setting in FragPipe), whereas the optimized settings used in the final version included y-, b-, and a-ions. Similarly, one might expect new users to use different search engine versions and different settings.

All synthetic peptides were synthesized using Fmoc solid phase chemistry and their sequence was analyzed to predict the likelihood of a successful synthesis as described elsewhere (43).

Peptides were selected for each donor to reach the best match between the predicted MHC-I-peptide binding affinity (see below), the MHC-I haplotypes of the human host cells used to generate the MHC-I immunopeptidomes and the MHC-I haplotype of each donor. For the peptides Spike_153-160_ and R1AB_7012-7022_ the MHC-I was HLA-B*18:01 and - B*07:02, respectively, whereas for the NP-derived epitope candidates the match was with the HLA-B*07:02 because they all shared a peptide subsequence common to a known NP-derived epitope predicted to bind the same MHC-I variant.

For the Spike_153-160_ and R1AB_7012-7022_ epitope candidates, PBMCs were stimulated and restimulated with the single synthetic peptide unless the donors expressed both HLA-B*18:01 and -B*07:02. For all NP-derived epitope candidates, PBMCs were stimulated with a peptide pool (1 µM per peptide) and restimulated with a single synthetic peptide (10 µM). For the stimulation and restimulation with synthetic random peptides, a pool of 3 synthetic peptides not predicted to bind the specific MHC-I haplotype of a donor was used.

### Donors and MHC-I allele genotyping

200 µL of donor blood was used to extract genomic DNA using a QIAamp DNA Blood kit (Qiagen) as instructed. Extracted DNA quality and concentration was measured using a Nanodrop spectrophotometer (Thermofisher scientific). Two to three µg of DNA were used for MHC genotyping, which was carried out using Illumina NGS system. Briefly, PCR reaction was performed for exons two and three. Each PCR reaction included two primer pairs: an inner and outer target specific pair which contains the Illumina adaptors, barcodes (indexes) for each sequence and the sequencing primers (Illumina) binding site. After the PCR, samples were pooled per PCR mix and purified using Ampure XP beads (Beckman coulter). The concentration was measured using Picogreen and normalized to 4 nM. The mixes were pooled in a certain ratio and denatured by NaOH according to Illumina protocol. Library was loaded into MiSeq or NovaSeq. Sequencing data was demultiplexed and a fastq-file was generated for each sample. SeqPilot software (JSI medical system) was used for the assignment of the MHC haplotype.

Peripheral blood from donors was collected accordingly to the ethically approved protocols (RESCM-20/21-5960 and REC22/EE/0230) and human studies abided to the Declaration of Helsinki principles. All donors (n = 8; average age = 39.8; female:male ratio = 5:3) had blood withdrawal between 1 and 3 months after a resolved and diagnosed COVID-19 event. For a single donor included in this study, PBMCs withdrawn before COVID diagnosis, within 3 months after COVID-19 event resolution and longer than 6 months after COVID-19 resolution was carried out.

### Mouse splenocyte stimulation and IFN-γ quantification

Six- to eight-week-old female C57BL/6 NCRL mice were purchased from Charles River laboratories. All animal experiments were approved by Utrecht University Animal Ethics Committee (AVD1080020198224) and were conducted under the 3R principles. Mice were intravenously infected with 2000 CFU of *L. monocytogenes* (strain 10403S), diluted from a log phase culture in BHI. Spleens were collected 7 days after infection. Splenocytes from infected and uninfected groups were incubated with 1 µg/ml of synthetic Listeria peptides in RPMI media. After 2h of incubation at 37°C, Brefeldin (B7651, Sigma-Aldrich) was added, followed by an additional 4h incubation at 37°C. For staining, cell suspensions were first blocked with Fc Block (2.4G2, in house produced). Extracellular staining was performed with anti-CD8 (clone Ly-2; APC, eBioscience) and ViaKrome808 (Beckman Coulter, Indianapolis, IN, USA) in FACS Buffer (1X PBS supplemented with 2% FCS). Cells were fixed, permeabilized following manufacturer’s instructions (BD Bioscience) and stained with anti-IFN-γ (clone XMG1.2; PE; Invitrogen). Flow cytometry was performed using the Beckman Coulter Cytoflex LX at the Flow Cytometry and Cell Sorting Facility at the Faculty of Veterinary Medicine at Utrecht University. Acquired data were analyzed using FlowJo Software v.10.9 (FlowJo LLC, Ashland, OR, USA). The percentage of IFN-γ-producing CD8^+^ T cells in the spleens was calculated by subtracting background of splenocytes incubated without stimuli per individual mouse. GraphPad Prism version 9 was used for graphs generation and statistical analysis.

We considered responses to the tested synthetic epitope candidates as immunologically significant when the frequency of IFN-γ^+^ CD8^+^ splenocytes reacting to an epitope candidate was significantly higher for infected mice compared to: (i) uninfected mice, (ii) infected unstimulated mouse splenocytes, (iii) infected mouse splenocytes stimulated with the irrelevant peptide OVA_257-264_. For an immunologically significant response, all three criteria should have been met in all experiments performed (each experiment contained 8 mice).

## Results

### PEPSeek optimizes the identification of pathogen-derived antigenic peptides in MHC-I immunopeptidomes

**Figure 1:**
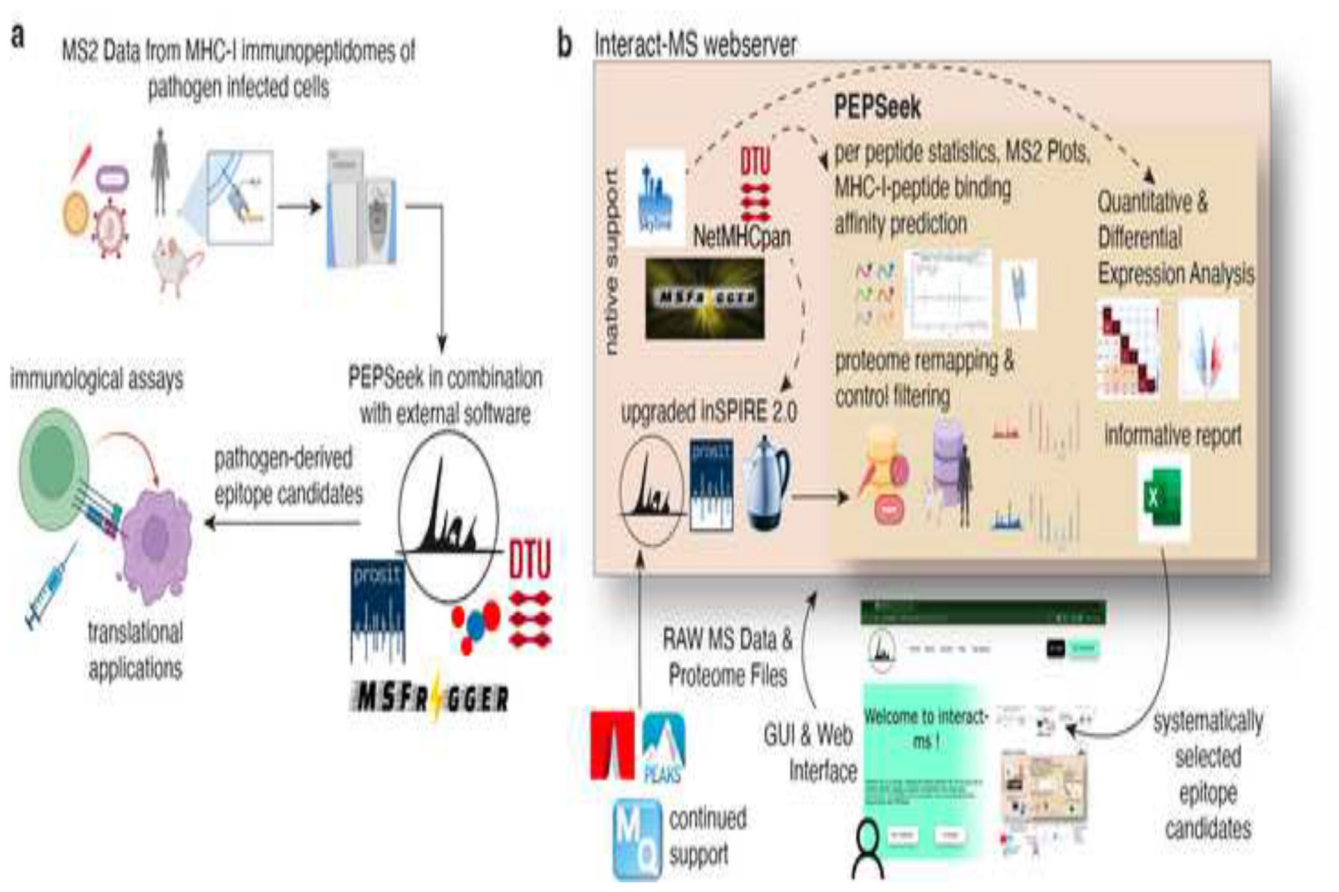
The PEPSeek workflow. (**a**) A simplified workflow demonstrating the practical application of PEPSeek on MHC-I immunopeptidomes of infected cells whereby the user obtains results from a single execution without needing to execute multiple intermediate steps. (**b**) Overview of the PEPSeek software integrated in the Interact-MS webserver platform. PEPSeek uses the rescoring core of the upgraded inSPIRE with the addition of native support for rescoring with MSFragger, prediction via NetMHCpan, and MS/MS file conversion with the ThermoRawFileParser. The platform also utilises an API framework to allow local execution via a browser-based GUI or web-based execution on a remote server.

We developed PEPSeek to enhance MS2 spectrum identification and enable users, regardless of bioinformatics expertise, to directly identify pathogen-specific peptides in immunopeptidomes, extrapolate their immunologically relevant features, and thereby generate a pool of epitope candidates ready to be tested *ex vivo* for recognition by pathogen-specific CD8^+^ T cells (Fig. 1a). PEPSeek allows the user to go directly from RAW MS data to potential pathogen-derived epitope candidates without the need of manual filtering, as well as to have sufficient information to manually and visually inspect the peptide spectrum matches (PSMs) of the epitope candidates identified in the MS data (Fig. 1b).

PEPSeek uses an upgrade of the original inSPIRE workflow. The use of inSPIRE gives the benefits of PSM rescoring using spectral prediction, retention time prediction, and (optionally) MHC-I predicted binding affinity. This enhanced rescoring approach implemented in inSPIRE 2.0 further adds an option to directly integrate MSFragger (47) while maintaining flexibility through compatibility with other major MS search engines. To note, MSFragger integration is achieved directly using the MSFragger standalone .jar file, not making use of the rescoring tool MSBooster (11), which has broader application also encompassing various proteomics use cases. The improved performance of the upgraded inSPIRE 2.0 in combination with representative search engines is reported in Fig. S1, S2. PEPSeek makes use of ThermoRawFileParser (44), NetMHCpan (38), and Skyline (48) for comprehensive data analysis within the platform (Fig. 1b). The integration with Skyline enables the PEPSeek quantitative analysis tool kit, which could be applied to discover, for instance, quantitative antigenic changes during an infection cycle in both the pathogen and host cell (see below). Due to its dependencies and main intended use cases, PEPSeek has several limitations and requirements, which are listed in detail in the Experimental Procedures section.

In PEPSeek, we make use of posterior error probability (PEP) in addition to the commonly used q-value (49-51) for selecting epitope candidates. While q-value estimates the false discovery rate (FDR) across multiple PSMs, PEP assesses the accuracy of each individual PSM (52). For instance, by applying a q-value cut-off of 0.01, we would have 990 PSMs correct and 10 wrong, which could be acceptable if the objective was an exhaustive identification of antigenic peptides. However, for identifying pathogen-specific epitopes for further immunogenicity testing, PEP offers additional specificity. We performed a preliminary benchmarking of the two statistical strategies on MHC-I immunopeptidomes of human and mouse infected cells (Table S1), thereby identifying a PEP cut-off of 0.1 as more stringent than the standard 1% q-value cut-off for most datasets (Fig. S3). In order to ensure maximum stringency in PEPSeek, we imposed this PEP cut off on top of the standard 1% q-value cut off (Fig. S3). In this study PSM level PEP threshold was used. However, users could also employ the more stringent peptide level PEP threshold, which is an option available in PEPSeek.

After applying PEP filtering, PEPSeek remaps all peptides to the host and pathogen proteomes’ reference database, also considering peptides that map to both proteomes and isoleucine (I)/ leucine (L) redundant peptides. PEPSeek eliminates all putative pathogen-derived peptides that could have also originated from the host proteome and all those that were detected in the control (uninfected) samples, since they could be either contaminants not included in the MS contaminant lists or noncanonical peptides produced by the host cell not included in the reference database. It then analyzes the remaining pathogen-specific peptides for MS quality and MHC-I binding affinity. Final outputs are provided as .xlsx files, as well as a .pdf file with spectral plotting of all epitope candidates’ PSMs, allowing full transparency and the ability of the user for further manual evaluation. The same information and graphics are also provided for all host peptides’ identifications.

To enhance accessibility, PEPSeek is distributed within our interact-ms web server platform, with an interactive GUI for both local and remote use, meaning a single installation could be accessed by an entire research team. Online (video) tutorials are provided for installation, execution, and analysis of results (see https://quantsysbio.github.io/interact-ms.html).

### Identification of novel antigen and epitope candidates in MHC-I immunopeptidome analysis of infected cells by applying PEPSeek

As a proof-of-principle, we applied the PEPSeek platform to MHC-I immunopeptidomes of: (i) human HEK293T, Calu-3, IHW01070 and A549 cells either infected or not infected with SARS-CoV-2 (36, 37) (ii) human HeLa and HCT116 cells either infected or not infected with *L. monocytogenes* (35), (iii) mouse Ana-1 cells either infected or not infected with *L. monocytogenes*, (iv) human HeLa cells either infected with *C. trachomatis* for 24 h and 48 h or not infected (t = 0 h; Table S1). We compared the list of pathogen-derived epitope candidates and cognate antigens identified by PEPSeek using either MSFragger or PEAKS DB to those reported in previous studies (35-37) or obtained using a standard PEAKS DB search for MHC-I immunopeptidomes generated in this study (Table S1). For the comparison with original studies, we matched the search engine employed in combination with PEPSeek to what was originally used; opting for PEAKS DB when that was the original search engine used in the cognate study, or MSFragger when MaxQuant or Spectrum Mill was used in the original study to provide a performance comparison within open access search engines. All comparisons between PEPSeek and the original studies were based on results extracted from the original publications (see Experimental Procedures for details)

**Figure 2:**
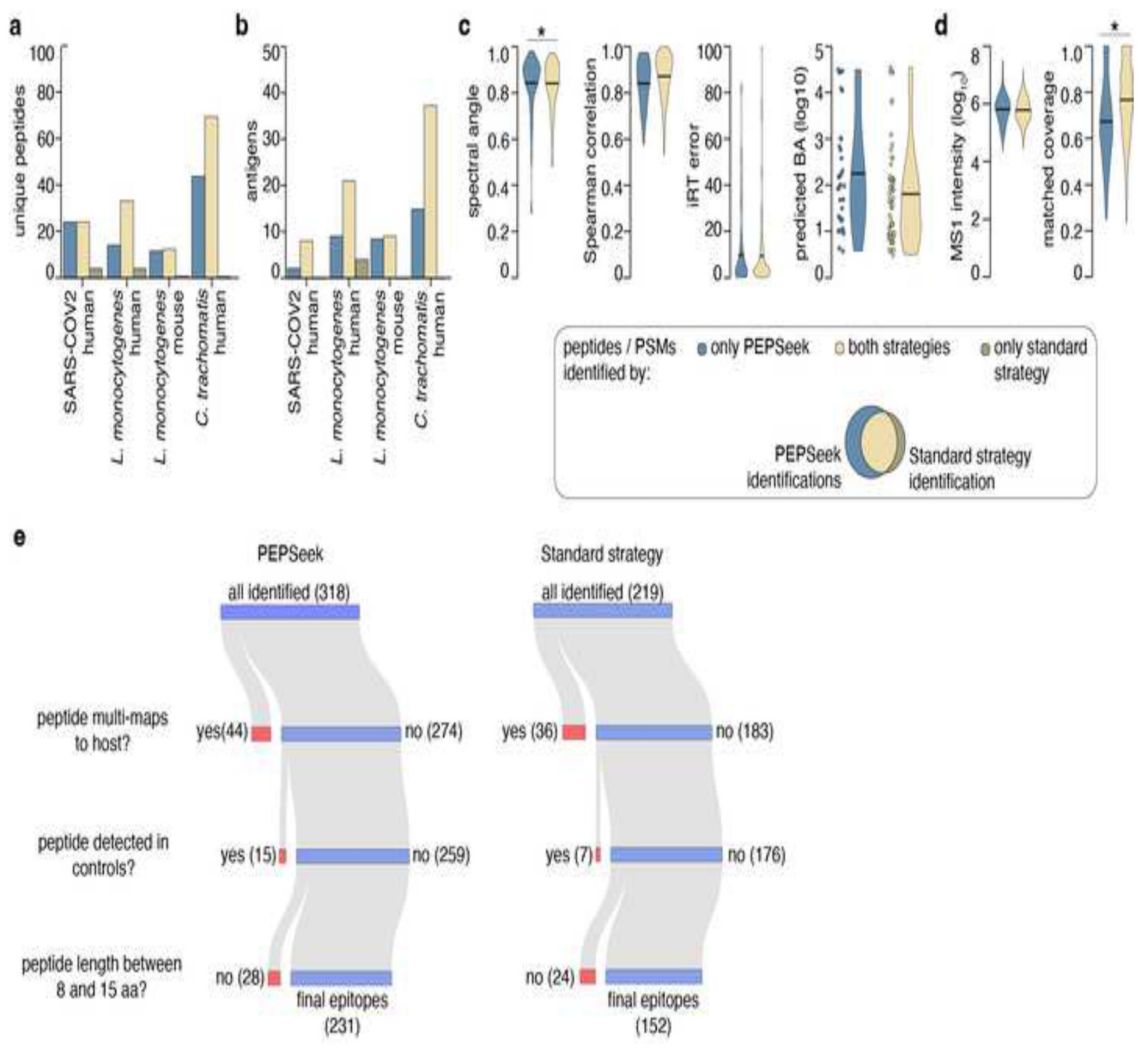
Identification of pathogen-derived novel epitope candidates and cognate antigens by PEPSeek. (**a-d**) Comparison of the outcome of the analyses carried out on the MHC-I immunopeptidomes of human and mouse cells either infected or not infected with either SARS-CoV-2, *C. trachomatis* or *L. monocytogenes*. (**a**,**b**) The number of pathogen-derived epitope candidates (**a**) and cognate antigens (**b**) gained, shared or lost by applying PEPSeek to the MHC-I immunopeptidomes of infected (and not infected) cells compared to the (original) standard search strategies. (**c**) Distribution of spectral angles, Spearman correlation and iRTs between measured and Prosit-predicted MS2 spectra and MS1 precursors, respectively, among the pathogen-derived epitope candidates identified only by PEPSeek (n = 149 PSMs) and those identified by both PEPSeek and the (original) standard search strategies (n = 496 PSMs) as well NetMHCpan predicted binding affinities for 9 amino acid-long peptides identified only by PEPSeek (n = 32 peptides) and those identified by both PEPSeek and the standard strategy (n = 56 peptides). (**d**) MS2 ion coverage and MS1 precursor’s ion intensity of the PSMs of pathogen-derived epitope candidates identified only by PEPSeek and those identified by both PEPSeek and the (original) standard search strategies. In (**c**,**d**), statistically significant p-values are denoted by * (two sided Mann-Whitney U test, p-value < 0.05). (**e**) Sankey diagram illustrating the peptide candidates retained or removed at each PEPSeek filtering step. Filtering is shown for all identified peptides which map to the pathogen proteome using inSPIRE rescoring within PEPSeek (left) or the standard method (right).

By analyzing the MHC-I immunopeptidome datasets of infected and not infected cells with PEPSeek, we obtained an overall 57% and 38% increase of identified epitope candidates and cognate antigens compared to the original analysis or analysis using a standard search engine strategy (Fig. 2a,b and File S1-S2). A similar, although less strong, rise in the global identification rate of peptides and cognate proteins was observed for the self-immunopeptidomes (Fig. S4a; n = 47,469 unique peptides and 282,568 PSMs identified by PEPSeek). Importantly these increases were calculated based on an analysis of the same dataset(s) by the benchmarked methods. The distribution of spectral angles, Spearman correlation, iRT prediction errors between experimental and Prosit-predicted MS2 spectra and MS1 precursor of pathogen-derived epitope candidates identified only by PEPSeek (n = 93 unique peptides and n = 159 PSMs) was comparable to those identified by both strategies (n = 138 unique peptides and n = 497 PSMs; Fig. 2c, File S1, File S3). This similarity was also observed for self-peptides (Fig. S4b). We observed slightly higher NetMHCpan predicted binding affinity for 9 amino acid-long peptides identified only by PEPSeek (32 peptides) compared to those identified by both strategies (56 peptides) (Fig. 2c). However, this might be due to the small data set, as no difference was observed when considering the entire host immunopeptidome (Fig. S4b). For datasets replacing MaxQuant/Spectrum Mill with MSFragger as the preliminary search engine, the improvement was attributed to PEPSeek rather than the change in search engine (Fig. S4c,d).

Only nine pathogen-derived epitope candidates were solely identified in the original studies or by applying a standard search strategy (Fig. 2a, File S1). The novel epitope candidates and self-peptides only identified by PEPSeek had, on average, MS2 spectra with significantly lower MS2 ion coverage and similar MS1 intensity than those identified by both strategies (Fig. 2d, Fig. S4b). This, combined with the robust metrics shown in Fig. 2c and Fig. S4b, suggested that PEPSeek was particularly useful for identifying peptides which had incomplete fragmentation. In such cases, PEPSeek’s use of relative intensity predictions from Prosit could have been key for their identification. In order to illustrate the effect of PEPSeek’s filtering steps to as well as the increase in peptides detected, we provide an overview of peptide gain and loss across PEPSeek steps Fig. 2e. This shows how identified peptides mapping to the pathogen proteome are excluded to due various conditions suggesting they may not be suitable epitope targets with MHC-I system.

To note, the IHW01070 cell line dataset was excluded from the above analysis, as PEPSeek analysis deemed it non-informative. The MS2 spectrum of the single SARS-CoV-2 peptide [NEVAKNLNESL], identified in the original study (36), was re-assigned to the human Nucleoprotein TPR-derived peptide [RELQELQDSL] by PEPSeek (Fig. S5a), supported by better spectral metrics (Fig. S5b) and better predicted binding affinity to the specific MHC-I allele (Fig. S5c), indicating a more likely match with the human peptide. This can serve to highlight the ability of PEPSeek to avoid false identifications, as well as boosting correct identifications. No additional SARS-CoV-2 peptides were found in that dataset.

### Immunogenicity of novel SARS-COV-2-derived epitope candidates identified by PEPSeek

**Figure 3:**
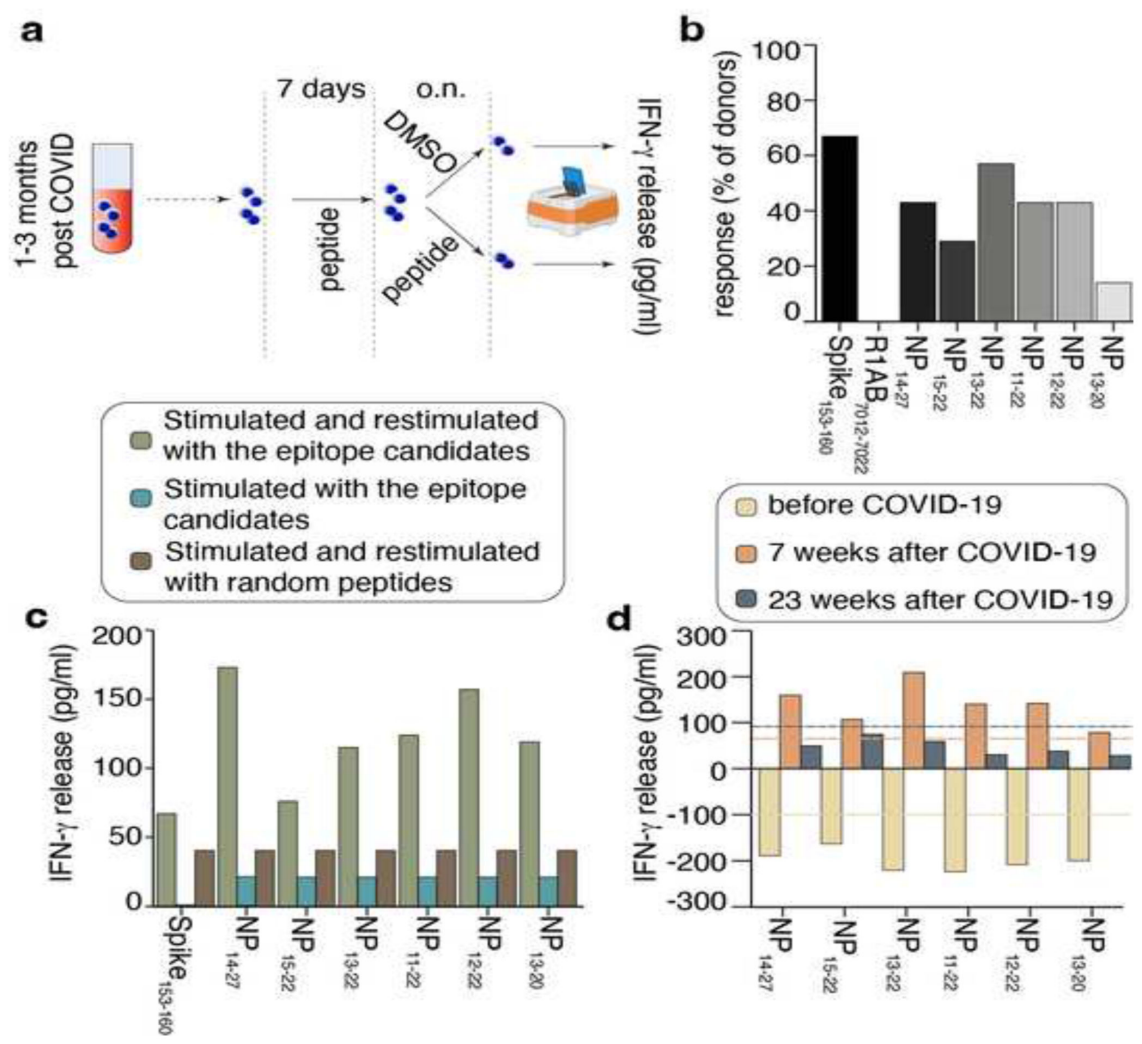
Immunogenicity of a pool of SARS-CoV-2-derived epitope candidates identified only by PEPSeek in human MHC-I immunopeptidomes. (**a**) Experimental design. (**b**) Frequency of donors that showed a significant IFN-γ^+^ secretion by PBMCs stimulated and restimulated with SARS-CoV-2-derived epitope candidates. The peripheral blood of MHC-I-peptide matched donors (n = 3 for Spike1_53-160_ and R1AB_7012-7022_, and n = 7 donors for any other epitope candidates) were withdrawn within 3 months from a resolved COVID-19 infection. (**c**) The IFN-γ^+^ secretion by PBMCs stimulated (and restimulated) with synthetic peptides is shown. The IFN-γ^+^ background concentration (stimulation and restimulation by DMSO) has been subtracted. Values are the mean of 2 technical replicates. The PBMCs of the donor MM-HD-63 were here tested. (**d**) The IFN-γ^+^ secretion by PBMCs of a donor (IDEA-038) whose blood was withdrawn in a longitudinal study. The IFN-γ^+^ concentration of the samples stimulated and restimulated with the epitope candidates subtracted the background IFN-γ^+^ concentration is reported. The IFN-γ^+^ concentration of the samples stimulated and restimulated with the random peptide pool is shown as dash line for each sample. Values are the mean of 2 technical replicates. All experimental results are shown in File S5.

To estimate the immunological relevance of the epitope candidates identified by PEPSeek, we initially focused on SARS-CoV-2. For this analysis, PEPSeek was applied with MSFragger via the interact-ms webserver. By analyzing the MHC-I immunopeptidomes of four infected and not infected human cell lines (Table S1) with PEPSeek, we identified an additional 24 unique SARS-CoV-2-derived epitope candidates, which represented an increase of 86% on those identified by applying the original standard search engine to those MHC-I immunopeptidomes (File S1). Among them, 14 have never been confirmed as antigenic peptides, *i.e*., they have been neither identified in immunopeptidomes nor were proven to be recognized by T cells upon co-culture with antigen-presenting cells or infected cells, although they were all derived from known SARS-CoV-2 antigens (File S1). Among them, we selected (see Experimental Procedures for details and Table S2), successfully synthesized and confirmed by MS, 8 epitope candidates comprised of 1 peptide derived from the Spike glycoprotein (Spike), 1 peptide derived from replicase polyprotein 1ab (RA1B), and 6 peptides derived from the nucleocapsid phosphoprotein (NP; see File S4). We tested the response against the 8 synthetic epitope candidates in human peripheral blood mononuclear cells (PBMCs) of MHC-I-matched donors collected after 1 – 3 months of a resolved diagnosed coronavirus disease 2019 (COVID-19) episode. All donors were vaccinated against SARS-CoV-2 (Table S3). The PBMCs were stimulated as described in the Experimental Procedures section, where also the statistical analysis is described in details. We detected an immunologically significant response of PBMCs against 7 out of the 8 tested epitope candidates in at least one donor, and for 5 out of 8 epitope candidates in at least 30% of the tested donors (Fig. 3b, File S5). Among the latter, 4 epitope candidates triggered an average IFN-γ secretion higher than 100 pg/ml compared to the negative control reference in PBMCs of those donors that showed a peptide-specific response (File S5). A representative assay outcome from PBMCs of a donor collected within 3 months of resolved COVID-19 is shown in Fig. 3c. For the donor who showed an immunologically significant response to most of the tested synthetic epitope candidates (for 6 out of 8) in PBMCs collected within 7 weeks of a resolved COVID-19, we also tested the IFN-γ release by PBMCs collected before COVID-19 diagnosis and 23 weeks after it. An immunologically significant response against all 6 NP-derived epitope candidates was only observed in the PBMCs collected within 3 months of resolved COVID-19 in this donor (Fig. 3d, File S5).

### Immunogenicity of novel *L. monocytogenes*-derived epitope candidates identified by PEPSeek

**Figure 4:**
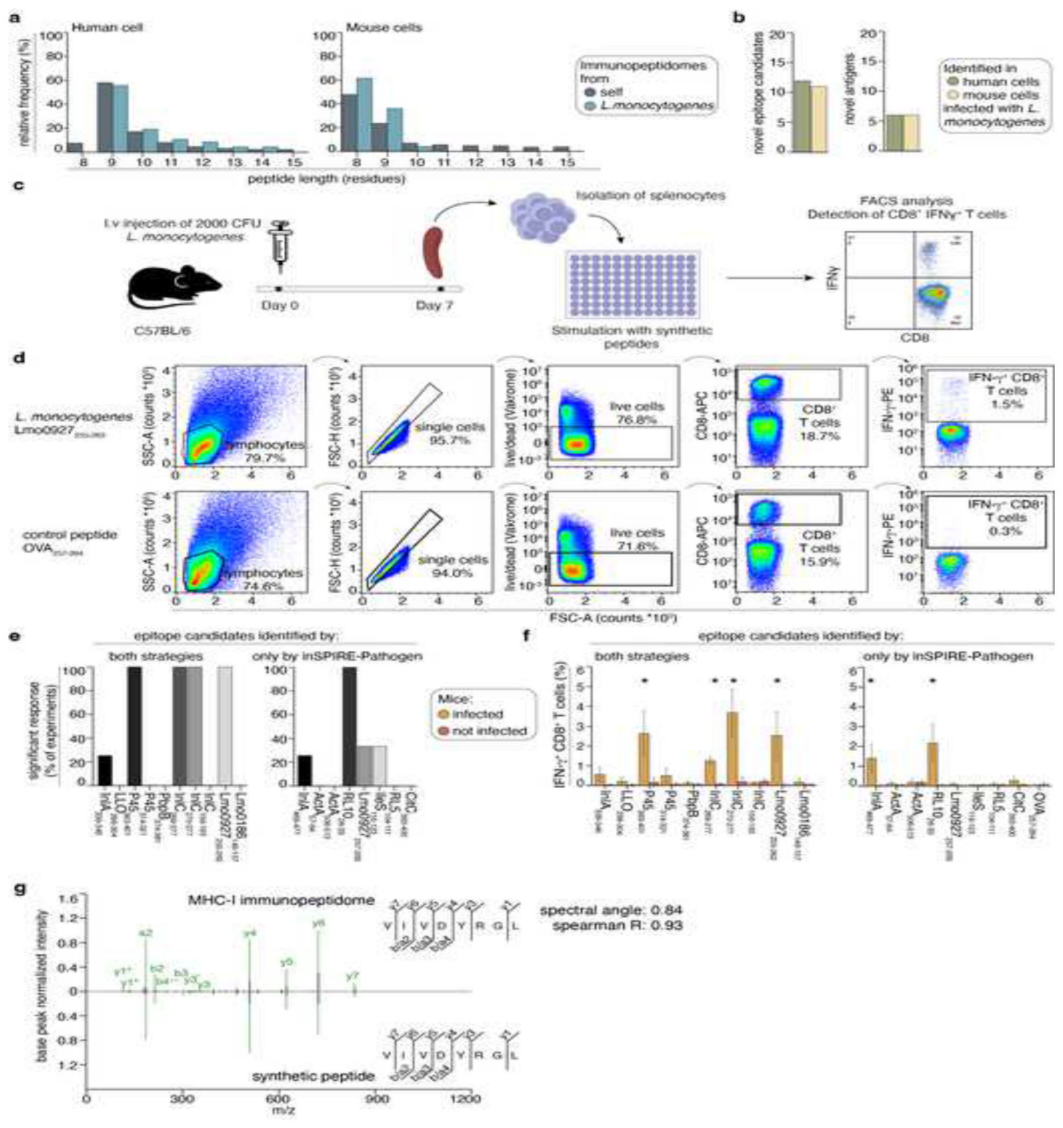
Features and immunogenicity of *L. monocytogenes* derived epitope candidates identified in human and mouse MHC-I immunopeptidomes by PEPSeek. (**a**) Peptide length distribution of *L. monocytogenes*-derived epitope candidates and the human and mouse self MHC-I immunopeptidomes. (**b**) Number of *L. monocytogenes* epitope candidates and antigens (never positively identified before in MHC-I immunopeptidomics and T cell assays) identified only by applying PEPSeek. (**c**) Experimental design. (**d**) Representative FACS plots showing the frequencies of IFN-γ^+^ CD8^+^ cells among splenocytes of an infected mouse, stimulated *ex vivo* with either the epitope candidate Lmo0927_255-263_ or the control Ova_257-264_ peptide. (**e**) Frequency of experiments (out of 3-4 experiments) showing a statistically significant higher frequency of IFN-γ^+^ CD8^+^ T cells specific for the indicated *L. monocytogenes* epitope candidates in infected compared to not infected mouse spleens (n = 4 mice per group). (**f**) Frequencies of *L. monocytogenes* epitope candidate-specific IFN-γ^+^ CD8^+^ T cells in the spleens of infected and not infected mice in a representative experiment, corrected for background measured in unstimulated samples. Mean values (n = 4) and SEM (bars) are shown. Statistically significant p-values are denoted by * (Two-way ANOVA and Sidak's multiple comparisons test comparing the peptides of interest to OVA _257-264_). Of note, CD8^+^ T cells responses to InlA_469-477_ tested significant in this but not in other experiments. All experimental results are shown in File S7. **(g)** MS2 spectra of the *L. monocytogenes* RL10_26-33_ epitope identified only by PEPSeek in the mouse MHC-I immunopeptidome and of its cognate synthetic peptide. Detected *m/z* and charges in the MS2 spectra matched to potential y-, b-, or a-ions and shared peaks between the immunopeptidomes and the synthetic peptide are indicated in green. Matched peaks of unknown origin are indicated in black. Peaks not matched between MS2 spectra of the immunopeptidome and the synthetic peptide but matched to either one or the other are marked in grey. Double charged ions are marked as ^++^. Ions’ neutral loss of water and of ammonia are symbolized by ° and *, respectively. The y-, b-, or a-ions matched in the cognate sample are reported in the peptide sequence in the central panels. Spectral angles and Spearman correlation values are indicated.

As a second application of PEPSeek, we moved from viruses to intracellular bacteria and analyzed the features of the *L. monocytogenes*-derived epitope candidates identified by PEPSeek in both human (n = 47) and mouse (n = 23) MHC-I immunopeptidomes (File S1). In this case, PEPSeek was applied with PEAKS DB search engine to match the search engine of choice in the previous study on *L. monocytogenes* infected immunopeptidomes (35). For both the mouse and human datasets, the *L. monocytogenes*-derived epitope candidates had a similar peptide length distribution to the host-derived MHC-I immunopeptidome (Fig. 4a). Among the *L. monocytogenes*-derived epitope candidates in the human MHC-I immunopeptidomes of two cell lines, 12 were never identified before and corresponded to six *L. monocytogenes’* proteins that, so far, have not been described as antigens, *i.e*., they were neither identified in MHC-I immunopeptidomes nor were recognized by T cells in activation/cytotoxic assays (Fig. 4b; see Experimental Procedures for the criteria for defining an antigen as such). Among the 23 *L. monocytogenes*-derived epitope candidates in the MHC-I immunopeptidomes of the mouse macrophage cell line Ana-1, 11 were identified only by PEPSeek in this dataset and have never been previously described in other studies. Eight of them were derived from *L. monocytogenes’* proteins that were not yet known as antigens (Fig. 4b, File S1-S2). Five of these eight peptides localize to the bacterial cytoplasm. This contrasts with the 11 novel epitope candidates identified by both search engine strategies, which mainly localized to the bacterial periphery or were secreted into the host cell cytosol (File S1-S2).

We confirmed the correct PEPSeek identifications of the 23 *L. monocytogenes*-derived epitope candidates by comparison of their MS2 spectra to those of corresponding synthetic peptides (File S6). For 5 out of 23 epitope candidates the synthesis was unsuccessful (Table S2) and therefore these candidates could not be further tested. To test the immunogenicity of the remaining 18 *L. monocytogenes*-derived epitope candidates, C57BL/6 mice were infected with *L. monocytogenes* or left uninfected. Seven days later, proportions of splenic CD8^+^ T cells responding to the epitope candidates (Table S2, File S1) were determined *ex vivo* by stimulating the splenocytes with synthetic peptides for 6 h followed by intracellular IFN-γ cytokine staining combined with flow cytometry analysis (Fig. 4c,d).

We repeated the experiment at least 3 times for each peptide. Responses were considered immunologically significant when peptide-specific IFN-γ^+^ CD8^+^ T cell responses were significantly higher in mice infected and stimulated *ex vivo* with a given synthetic peptide compared to (i) not infected mice stimulated *ex vivo* with the same synthetic peptide, as well as to (ii) infected mice stimulated *ex vivo* with the control peptide OVA_257-264_ [SIINFEKL], in all experiments. Five out of the 18 synthetic peptides tested triggered an immunologically significant CD8^+^ T cell activation (Fig. 4e, Table S2, File S7). The IFN-γ^+^ CD8^+^ T cell frequency for these epitopes was larger than the response detected against the well-known LLO_296-305_ epitope (Fig. 4f), which for years has been the reference epitope for studies on *L. monocytogenes* in C57BL/6 mouse models. Among these 5 epitopes, RL10_26-34_ was identified only by PEPSeek, and derived from a cytoplasmic *L. monocytogenes*’ protein that was not previously described as an antigen. Confirmation of the correct identification of this epitope via MS2 comparison with the cognate synthetic peptide is shown in Fig. 4g.

### PEPSeek sheds light on the difference between the infection cycle of *C. trachomatis* and *L. monocytogenes*

In our final application, we applied PEPSeek to MHC-I immunopeptidomes of HeLa cells either infected or not infected with *C. trachomatis*. This dataset was newly generated for this study and so PEPSeek was applied with PEAKS DB due to superior benchmarking performance (Fig S2). We identified additional 44 *C. trachomatis*-derived antigenic peptides compared to the standard search strategy, representing an increase of 64% of the *C. trachomatis*-derived antigenic peptides (Fig. 2a, File S1). None of these antigenic peptides were described before (File S1). The 113 antigenic peptides identified by PEPSeek derived from 52 *C. trachomatis* proteins, 50 of which were not known to be antigenic (Fig. 2b, File S2).

Beyond the identification of potential epitope candidates, PEPSeek enabled qualitative and quantitative analysis that could decipher, for example, how the different infection cycle mechanisms of pathogens are reflected in the MHC-I immunopeptidomes of infected cells. As a proof of principle, we compared the qualitative and quantitative antigenic landscape of *C. trachomatis* and *L. monocytogenes* presented by infected HeLa cell lines (Table S1).

**Figure 5:**
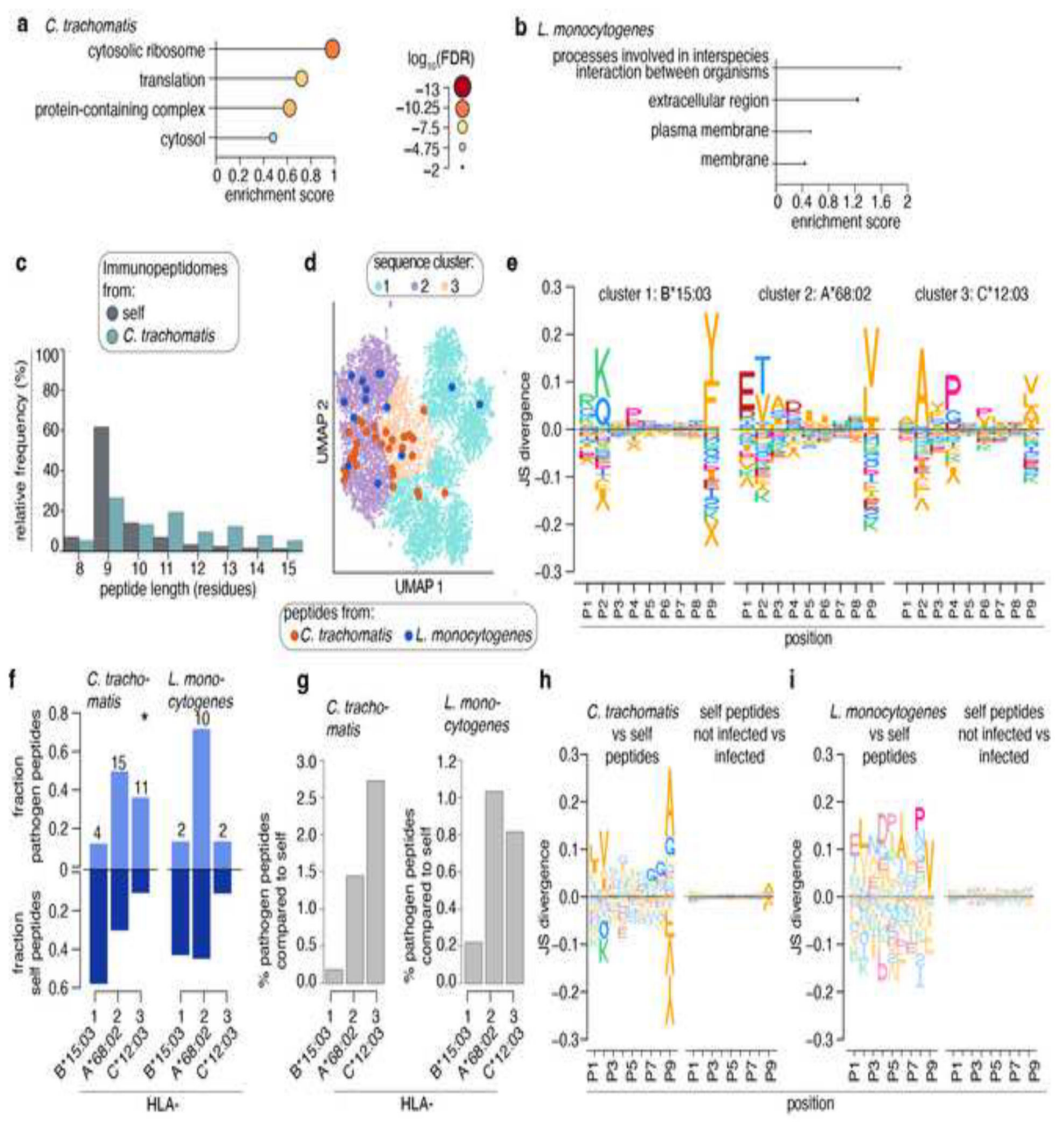
Different impact of *C. trachomatis* and *L. monocytogenes* on peptide repertoire in MHC-I immunopeptidomes of HeLa cells in a qualitative analysis. (**a-b**) Overrepresentation analysis of *C. trachomatis* (**a**) and *L. monocytogenes* (**b**) antigens identified by PEPSeek in the MHC-I immunopeptidomes of infected HeLa cells. Shown are selected gene sets with their respective enrichment score and FDR (see also File S8, S9). (**c**) Length distribution of antigenic peptides derived from *C. trachomatis* and the self MHC-I immunopeptidomes of HeLa cells detected by PEPSeek. (**d**) Sequence clustering. UMAP of one-hot-encoded, 9 amino acid long peptides derived from self, *C. trachomatis* and *L. monocytogenes*, as well as sampled self peptides predicted to be MHC-I binders. Each dot represents a single peptide sequence. *C. trachomatis* and *L. monocytogenes* peptides identified by PEPSeek are indicated in red and dark blue, respectively. (**e**) Peptide sequence motifs derived from the peptide sequence clusters in (**d**) and their corresponding MHC-I alleles. (**f**) Relative fraction of *C. trachomatis-* and *L. monocytogenes-*derived (light blue) and self-derived (dark blue) antigenic peptides. Numbers on top of bars indicate the number of pathogen-derived epitope candidates. Pearson's chi-squared test was performed to compare the peptide distribution among HLA-I alleles. Only the *C. trachomatis*-derived peptides in the HLA-C*12:03 cluster were significantly overrepresented (labelled by *, p-value = 3.7 10^-7^) than the self-peptides. (**g**) Percentage of pathogen peptides in the host immunopeptidome per MHC-I allele. (**h**) Sequence motif comparison between *C. trachomatis* and self-peptides (left) and between self-peptides detected after 24 / 48 h post infection and in control samples (right) shown as difference motifs. (**i**) Sequence motif comparison between *L. monocytogenes* and self-peptides (left) and between self-peptides in immunopeptidomes of infected vs not infected cells (right) shown as difference motifs. In (**h**,**i**) amino acids displayed with colors are statistically significant (Fisher’s exact test, p-value < 0.05), whereas the transparent amino acids are not. In (**c-i**) only 9 amino acid-long peptides were considered.

The *C. trachomatis* antigenic landscape was dominated by a network of antigens (28 out of 52) derived from members of the translational functional group, *i.e*., mainly ribosome-associated antigens present at both 24 h and 48 h post-infection (Fig. 5a, Fig. S6a, File S8). This class of proteins is known to be expressed throughout the developmental cycle and classified as members of the constitutive expressed gene cluster (53). A second functional network (n = 6) was represented by protein-folding chaperones and stress response proteins. Pathogenesis-associated proteins (n = 7) were also notable, especially those from the T3SS (n = 5), including inclusion membrane proteins (IncE; CT_529; CT_618), T3SS needle components (CT_579, CopD) vital for effector protein translocation, and chaperones (CT_667, CdsG) that promote the secretion of T3SS apparatus proteins (25, 53) (Fig. 5a, Fig. S6a, File S8). In contrast, in the MHC-I immunopeptidomes of HeLa cell lines infected with *L. monocytogenes*, membrane and extracellular (secreted) proteins were the most enriched, although to a lesser extent than the *C. trachomatis* antigens (Fig. 5b, Fig. S6b, File S9). Furthermore, *L. monocytogenes* antigens in the HeLa MHC-I immunopeptidomes were mainly derived from the extracellular region (Fig. S6b), reflecting the secretion of these virulence-associated antigens into the host cell cytosol, where they have direct access to the standard MHC-I APP.

The antigenic landscape of the two pathogens also differed in terms of antigenic peptide features: *C. trachomatis*-derived peptides were significantly longer, on average, than the self MHC-I immunopeptidomes (Fig. 5c), in contrast to what we observed for the *L. monocytogenes*-derived peptides (Fig. 4a). Hence, we investigated if pathogen-derived peptides also differed in their sequence motifs compared to self-peptides. To align peptides identified by PEPSeek with the most probable corresponding MHC-I allele, we considered all detected 9 amino acid-long peptides derived from *C. trachomatis* and *L. monocytogenes* as well as those derived from the self MHC-I immunopeptidomes. Upon encoding the 9 amino acid-long peptide sequences from both pathogens and self, peptides were grouped into three distinct clusters, exhibiting sequence motifs that were characteristic of the HeLa cell’s HLA-B*15:03, -A*68:02, and -C*12:03 alleles (Figure 5d,e, details in Experimental Procedures). In HeLa cells, the distribution of *L. monocytogenes*-derived peptides did not significantly differ from that of self-peptides across MHC-I clusters. In contrast, *C. trachomatis*-derived peptides were significantly more represented in the HLA-C*12:03 cluster than the self-peptides (Fig. 5f). Considering only 9 amino acid-long peptides, *C. trachomatis* peptides made up 0.8% of the immunopeptidome (0.19% for HLA-B15:03, 1.44% for HLA-A68:02, and 2.72% for HLA-C12:03). *L. monocytogenes* peptides accounted for 0.6% (0.22% for HLA-B15:03, 1.03% for HLA-A68:02, and 0.82% for HLA-C*12:03) (Fig. 5g).

Comparing the peptide sequence motifs across all identified peptides, *C. trachomatis* peptides and self-peptides differed strongly in the anchor site at P9 (Fig. 5h), with a prevalence of amino acids such as alanine (A) and glycine (G), unlike the typical sequence motifs of HeLa cell’s MHC-I immunopeptidomes (Fig. 5e). Self-peptides in either infected or not infected cells shared similar sequence pattern, with slight variations at P9 (Fig. 5h). In HeLa cells infected with *L. monocytogenes*, the sequence patterns of pathogen and self-peptides differed in all residues, likely due to the low number of *L. monocytogenes*-derived 9 amino acid-long peptide peptides included in this analysis. No differences in self peptides between infected and not infected cells with *L. monocytogenes* were observed (Fig. 5i).

### PEPSeek quantification toolkit sheds light on the different impact of pathogen infection on the self-immunopeptidomes of human cell lines

**Figure 6:**
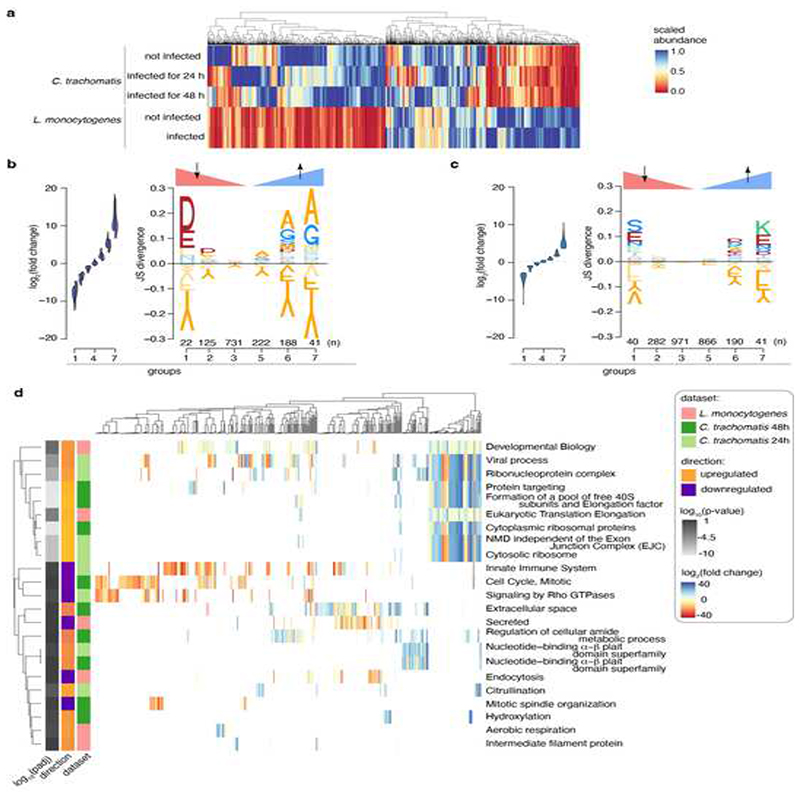
Different impact of *C. trachomatis* and *L. monocytogenes* on self-peptide repertoire of MHC-I immunopeptidomes of HeLa cells based on a quantitative analysis. (**a**) Heatmap of normalized MS1 intensities of self-peptides of HeLa cells either infected or not infected with *C. trachomatis* and *L. monocytogenes*. Shown are all antigenic peptides quantified across both HeLa datasets. For each peptide, the abundance was scaled across both datasets and all conditions, so that the sample with highest abundance equals 1 (*i.e*., abundances of each peptide were divided by max abundance detected for that peptide across all datasets/conditions). (**b-c**) Peptide sequence diversity depending on abundance fold change upon infection of HeLa cells with *C. trachomatis* (**b**) and *L. monocytogenes* (**c**). In (**b**), fold changes from 0- to 24-hour time points are shown in blue and fold changes for 0- to 48-hour time points are shown in purple. Peptides were grouped into 7 clusters according to the fold change in their abundance upon infection as illustrated in the violin plots. For each group, peptides were aligned at their C-termini and difference in peptide sequence motifs between each group and group 4 (*i.e*., peptides that did not change their abundance upon infection) are displayed for the C-terminal amino acids. In (**b**,**c**) amino acids displayed with colors are statistically significant (Fisher’s exact test, p-value < 0.05), whereas the transparent amino acids are not. (**d**) Different impact of *C. trachomatis* and *L. monocytogenes* on self-antigenic landscape of MHC-I immunopeptidomes of HeLa cells based on a quantitative analysis. Gene set enrichment analysis of self-antigens in the MHC-I immunopeptidome of HeLa cells upon *C. trachomatis* and *L. monocytogenes* infection. Detected self-antigens were mapped to their respective genes. Shown are significantly enriched gene sets (rows) with their respective genes (columns) colored by their detected log_2_ fold-changes. White color indicates genes not present in each gene set.

To investigate the potential impact of pathogen infection on self MHC-I immunopeptidomes, we exploited the quantitative toolkit of PEPSeek and analyzed the self-immunopeptidomes of infected and not infected HeLa cells considering all self-peptides identified by PEPSeek. In agreement with what we observed at the qualitative level (Fig. 5i), the abundance of antigenic self-peptides of HeLa cells either infected or not infected with *L. monocytogenes* were comparable (Fig. 6a). In contrast, we could observe a stronger degree of variation in the abundance of self-peptides upon *C. trachomatis* infection (Fig. 6a). To further investigate this potential phenomenon, we clustered the self-peptides identified and quantified by PEPSeek into 7 groups based on degree of variation in their abundance upon infection with either *C. trachomatis* or *L. monocytogenes* (see Experimental Procedures for details). Among the self peptides either downregulated or upregulated upon *C. trachomatis* infection, the C-terminal residue had the largest variation compared to peptides whose abundance was not altered upon infection, with a prevalence of amino acids such as A and G among the most upregulated self peptides (Fig. 6b). These amino acids were those overrepresented in the C-terminal residues of *C. trachomatis* immunopeptidomes (Fig. 5h). Spectral metrics of peptides with A/G at their C-term are comparable to those of all other identified peptides for both *C. trachomatis* - derived peptides and host-derived peptides, indicating reliable identification (Fig. S6c). This phenomenon was less evident in the self immunopeptidomes of HeLa cells upon infection with *L. monocytogenes* (Fig. 6c).

When we considered the self antigens rather than the single peptides in the MHC-I immunopeptidomes of either infected or not infected HeLa cells, we observed an overlap in the self antigenic landscape between the different conditions without a significant overrepresentation of self proteins involved in specific pathways (Fig. S7a). We then aggregated peptide fold changes (extracted from the quantitative toolkit of PEPSeek) to antigen fold changes and investigated the potential impact that the pathogens could have induced in the HeLa cell metabolism and the self antigenic landscape (Fig. S7b). Mapping antigens to genes facilitated gene set enrichment analysis (Fig. 6d), showing that upon *C. trachomatis* infection HeLa’s self-antigenic landscape was depauperated of antigens associated with innate immune system, mitotic cell cycle, signaling by Rho GTPases and mitotic spindle organization. In contrast, antigens associated with viral processes, ribonucleoproteins, ribosomes and translation, Nonsense-Mediated mRNA Decay (NMD) pathway, extracellular space proteins, citrullination and hydroxylation, regulation of cellular amide metabolic process, as well as nucleotide−binding α-β plait domain superfamily (ATP/GTP binding proteins) were upregulated. Upon *L. monocytogenes* infection, HeLa’s self antigenic landscape was positively enriched in antigens associated with translation, aerobic respiration, intermediate filament proteins and general developmental processes, whereas we observed a downregulation of secreted protein and endocytosis pathways (Fig. 6d).

## Discussion

In this study, we have demonstrated the power and precision of our specialized PEPSeek software suite for novel target discovery of pathogen epitopes. We believe that the combination of inSPIRE’s highly performant rescoring approach with automated and interpretable selection of epitope candidates can accelerate pathogen epitope discovery studies in the future. In this study, PEPSeek enabled novel antigenic peptide discoveries in all investigated datasets, increasing pathogen peptide identification rates by 57% across studies and antigen identification rates by 38% compared to standard approaches. Pathogen-derived antigenic peptides exclusively identified by PEPSeek were immunogenic both in human donors with resolved COVID-19 and in a mouse model after *L. monocytogenes* infection, thereby demonstrating the utility of PEPSeek in identifying epitope candidates that are potentially immunogenic.

The application of PEPSeek also gave the opportunity to significantly increase our knowledge of the pathogen antigenic landscape and showed that the variety of bacterial antigens presented at the cell surface was far more varied than generally appreciated. For example, while protective CD8^+^ T cell responses to *L. monocytogenes* were supposed to be directed to the secreted (extracellular region) antigens (16), we found that approximately half of the MHC I-associated *L. monocytogenes* epitope candidates derived from membrane-associated antigens and proteins from the bacterial cytosol. Moreover, the RL10_26-33_ epitope, identified by applying PEPSeek and derived from the bacterial 50S ribosomal protein L10, was detected by roughly 2% of CD8^+^ splenocytes in *L. monocytogenes* infected mice (Fig. 4), indicating that not only the secreted antigens but also the full *L. monocytogenes* antigenic landscape may be a target of protective immune responses. This hypothesis is also supported by the evidence that prime boost vaccination with a predicted epitope of a bacterial surface antigen can trigger specific CD8^+^ T cell responses and immune protection against *L. monocytogenes* in mice (35).

For *C. trachomatis*, the importance of CD8^+^ T cells in immune protection is presently unclear (26). Despite the previous identification of a few membrane and inclusion-associated chlamydia CD8^+^ T cell antigens (27, 29, 30, 32, 54, 55), the secluded location of the bacterium within a membrane-bound inclusion suggested a poor access of *C. trachomatis* proteins to the MHC-I APP. Contradicting this notion, our analyses detected 113 epitope candidates derived from 52 *C. trachomatis* antigens located in the bacterial cytosol, the inclusion, or associated with the bacterial cell surface. This agrees with previous findings on *C. muridarum* infections (56). The remarkable abundance of epitope candidates derived from ribosomal proteins in our dataset may be explained by enhanced protein synthesis in response to stress, thereby ensuring efficient bacterium replication. However, beyond their conventional function in the protein synthesis machinery, *C. trachomatis* ribosomal proteins can possess extra-ribosomal functions (57, 58), indicating a possible response to infection stress and potential effects on immune regulation (59-62).

By applying PEPSeek, we could also shed light on how *C. trachomatis* and *L. monocytogenes* infections affect human antigen presentation. *L. monocytogenes* antigens appeared to follow the typical APP, whereas *C. trachomatis* altered antigenic peptide patterns. We observed presentation of longer peptides, preferentially presented by HLA-C*12:03 and with distinct C-terminal amino acids (enriched in A and G). In comparison to HLA-A and -B complexes, HLA-C molecules are generally poorly represented at the cell surface. This is hypothesized to be caused by a prolonged retention in the ER and a unique trafficking signal in the cytoplasmic tail of HLA-C allomorphs, which targets surface-expressed HLA-C complexes for internalization and lysosomal degradation (63). *Chlamydia* infection has been shown to manipulate both these organelles to secure a protected intracellular niche and acquire host cell factors for bacterial replication (64, 65). Close contacts between inclusion and ER may well facilitate the loading of retained HLA-C complexes with alternate cargo and augment their cell surface transport. Manipulation of the endosomal membrane trafficking by *C. trachomatis* inclusion proteins could provide an opportunity for intersection with recycling endosomes/lysosomes and enhance MHC-I APP by involving alternative proteolytic enzymes, such as cathepsins and metalloproteases. This may explain the partially different peptide sequence motifs and length of MHC-I presented *C. trachomatis*-derived epitope candidates. In addition to these non-conventional pathways, *C. trachomatis* antigens that ‘escape’ from the replication niche may be processed by the standard MHC-I APP or taken up by autophagosomes which may shuttle the bacteria and their antigens to recycling, MHC-containing endosomes (66).

Based on the antigenic landscape revealed by the quantitative toolkit of PEPSeek, we also showed evidence that the *C. trachomatis* infection cycle could impinge upon key metabolic pathways in the host cells such as translation and NMD, which are then reflected in the self antigenic landscape of infected cells. This extends the observation that *C. trachomatis* alters host gene expression and protein synthesis associated with the NMD pathway (67), transcriptionally downregulates pathways involved in mitotic and centrosome processes and upregulates others associated with viral infection, interferon regulation, lipid biosynthesis, cellular stress, and translation (68, 69).

Taken together, PEPSeek allows fast and reliable identification of antigenic (and immunogenic) peptides and provides a user-friendly toolkit to decipher qualitative and quantitative changes in host cells’ immunopeptidomes upon pathogen infection.

## Supplementary Material

File S1

File S2

File S3

File S4

File S5

File S6

File S7

File S8

File S9

File S10

File S11

Table S1

## Data Availability

The MS files of the *L. monocytogenes* infected and uninfected human HeLa and HCT116 cell lines are available at the ProteomeXchange Consortium via the PRIDE (70) partner repository with the dataset identifier PXD031451 as described in the original paper (35). The MS files of the SARS-CoV-2 infected an uninfected human HEK293T, Calu-3, IHW01070 and A549 cell lines are available at the ProteomeXchange Consortium via the PRIDE (70) partner repository with the dataset identifier PXD025499 and in the public proteomics repository MassIVE (https://massive.ucsd.edu) with the dataset identifier MSV000087225 as described in the original papers (36, 37). The MS files of the MHC-I immunopeptidomes of *L. monocytogenes* infected and uninfected mouse Ana-1 and of *C. trachomatis* infected and not infected human HeLa cell lines are available at the MassIVE online repository with the dataset identifier MSV000094354. In the dataset identifier MSV000094354 also the MS search files generated by all the described combination of search engine and rescoring strategies are reported. PEPSeek and inSPIRE have been implemented with Python and is available at GitHub (https://github.com/QuantSysBio/inSPIRE). The interact-ms software has been implemented with Python, Javascript, HTML, and CSS and is available at GitHub (https://quantsysbio.github.io/interact-ms.html). Analyses were carried out in Python 3.11. Figures have been generated in Python using the Plotly library and Logomaker for the sequence logo plots (71). Postprocessing was done with Adobe Illustrator v26.2. Final MS analysis was carried out with PEAKS X Pro 10.6 and MSFragger 3.7.0, though preliminary results using MSFragger 3.6.0 are also available in the linked MassIVE repository. Rescoring was carried out with Percolator version 3.05.0. Preprocessing of MS RAW files was performed ThermoRawFileParser version 1.4.0 through the inSPIRE “convert” pipeline.

## Highlights

- PEPSeek enables sensitive pathogen peptide identification in infected IP data.
- PEPSeek identified novel peptides and antigens for three pathogen.
- Immunogenicity confirmed for SARS-Cov-2 and *L. monocytogenes* peptides.
- Quantative analysis revealed *C. trachomatis* impact on host cell immunopeptidome.

## In Brief Statement

PEPSeek (Pathogen EPitope Seeker) is a novel software that enables highly sensitive identification of pathogen-derived epitope candidates detected via mass spectrometry in MHC class I immunopeptidomes. PEPSeek is released along with the interact-ms webserver to enable easier access. PEPseek discovered novel peptides and antigens from 3 pathogens. The immunogenicity of PEPSeek identified peptides was confirmed for SARS-Cov-2 and *L. monocytogenes* while for *C. trachomatis*, qualitative and quantitative analysis revealed the impact of infection upon the host cell immunopeptidome
