# Supplementary material for: PEPSeek-mediated identification of novel epitopes from viral and bacterial pathogens and the impact on host cell immunopeptidomes
Source: Mol Cell Proteomics. Author manuscript; Available in PMC 2025 Aug 8. (PMC12002930; doi:10.1016/j.mcpro.2025.100937)

File S3. Pair plots of experimental MS2 spectra and cognate Prosit-predicted MS2 spectra for all pathogen-derived epitope candidates identified in this study. Comparisons between the relevant experimentally measured MS2 spectrum and the cognate Prosit predicted MS2 spectrum for all pathogen epitope candidates identified in this study. For each comparison the experimental spectrum is shown on the positive y-axis. Detected peaks in the MS2 spectra which are m/z matched to Prosit predicted peaks of the corresponding peptide are indicated in black. Other potential y-, b-, or a-ions are indicated in green for the putative peptide. The precursor ion is shown in pink if present. Peaks of unknown origin are indicated in grey. The corresponding Prosit predicted spectrum for the cognate peptide is shown on the negative y-axis. Predicted peaks matched to the experimental spectrum are indicated in blue while predicted peaks absent from the experimental spectrum are indicated in orange. Double charged ions are marked as $^{++}$. Ions' neutral loss of water and of ammonia are symbolized by ^{O} and *, respectively.

inSPIRE Spectral Plotting for chlamydia-hela-240117

Experimental Spectrum Colour Code:

- ● Experimental peak matched to a Prosit predicted peak.
- ● Possible ion unknown to Prosit.
- ● Precursor matched peak.
- ● Experimental peak not matched to any potential ion.

Prosit Spectrum Colour Code:

- ● Prosit predicted peak matched to experimental spectrum.
- ● Prosit predicted peak not matched to experimental spectrum.

Additional Notes:

° indicates an ion with loss of H_2O.

* indicates an ion with loss of NH_3.

Source Fu_HeLa_inf_2_R2 **Scan** 3613
Peptide SHVEYETANRHYA **Charge** 3 **Spectral Angle** 0.86

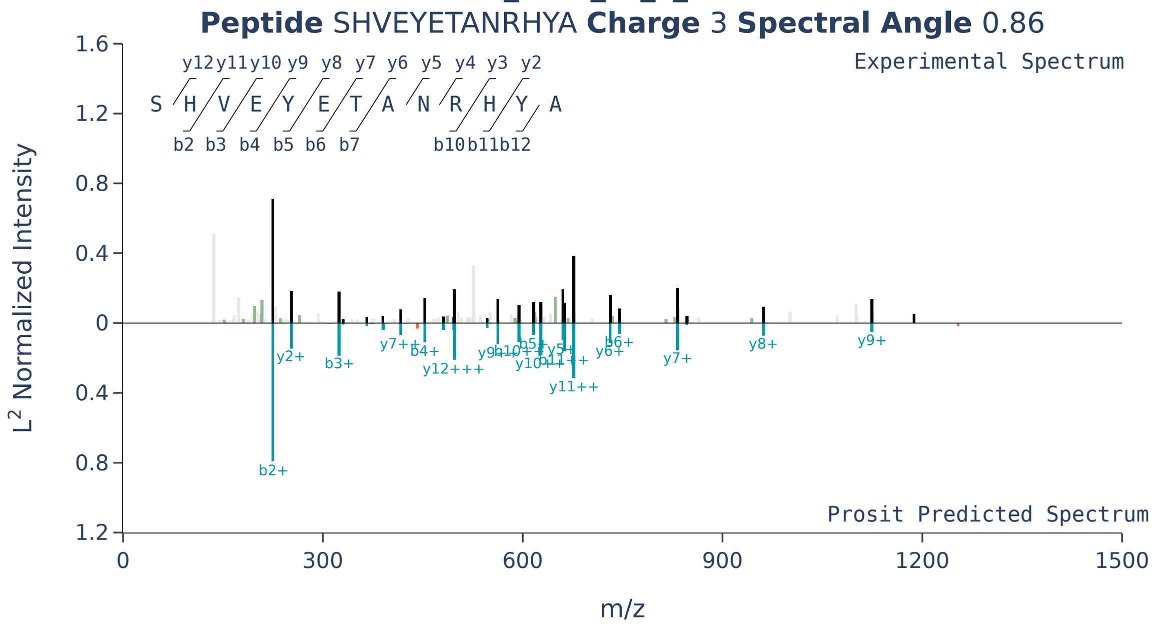

Source Fu_HeLa_inf_2_R2 **Scan** 2082
Peptide IRKISKPVQHHKEIA **Charge** 4 **Spectral Angle** 0.73

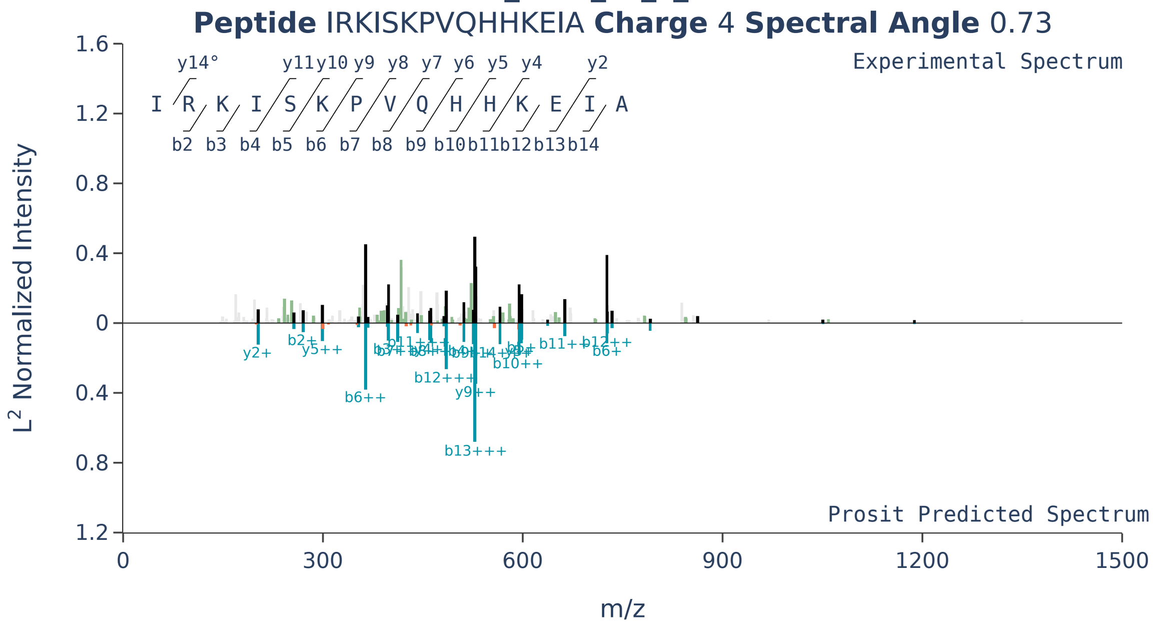

Source Fu_HeLa_inf_2_R1 **Scan** 9340
Peptide ERAAHVNAQFRF **Charge** 3 **Spectral Angle** 0.88

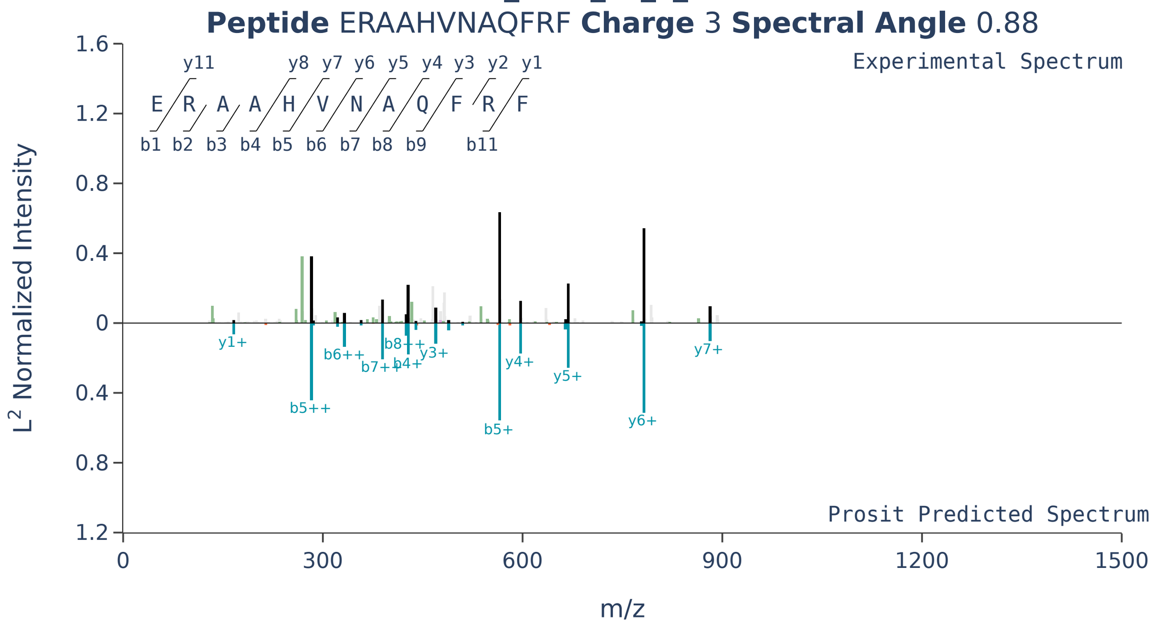

Source Fu_HeLa_inf_2_R2 **Scan** 5369
Peptide PAKVTRSALESAA **Charge** 2 **Spectral Angle** 0.86

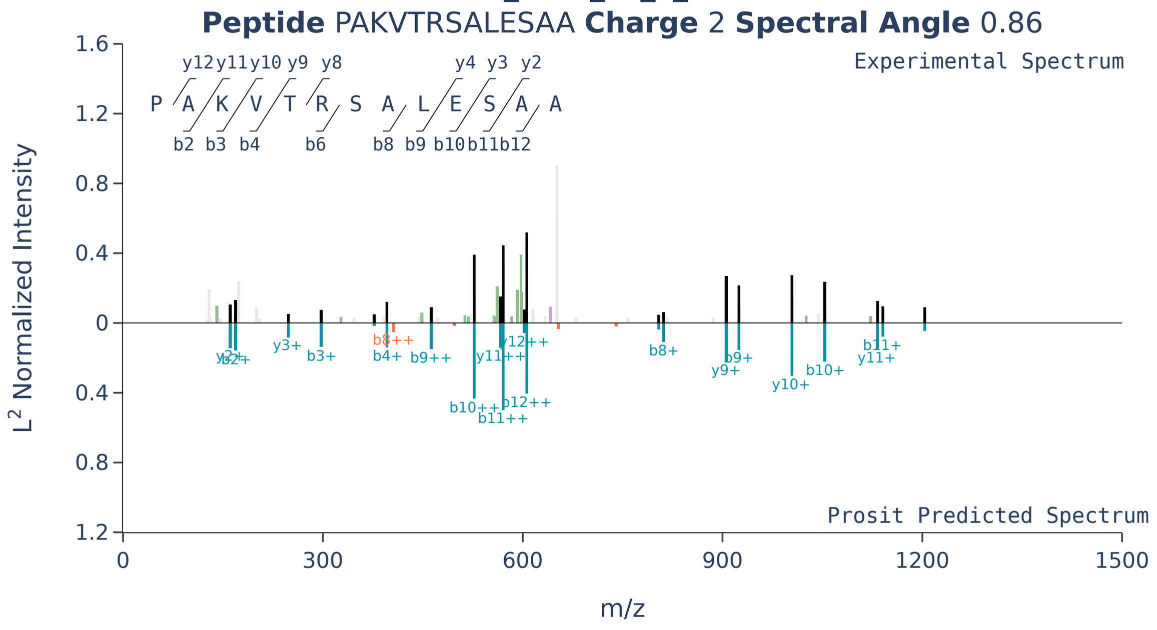

Source Fu_HeLa_inf_2_R1 **Scan** 5717
Peptide GIILPDTAKKKQDRA **Charge** 4 **Spectral Angle** 0.83

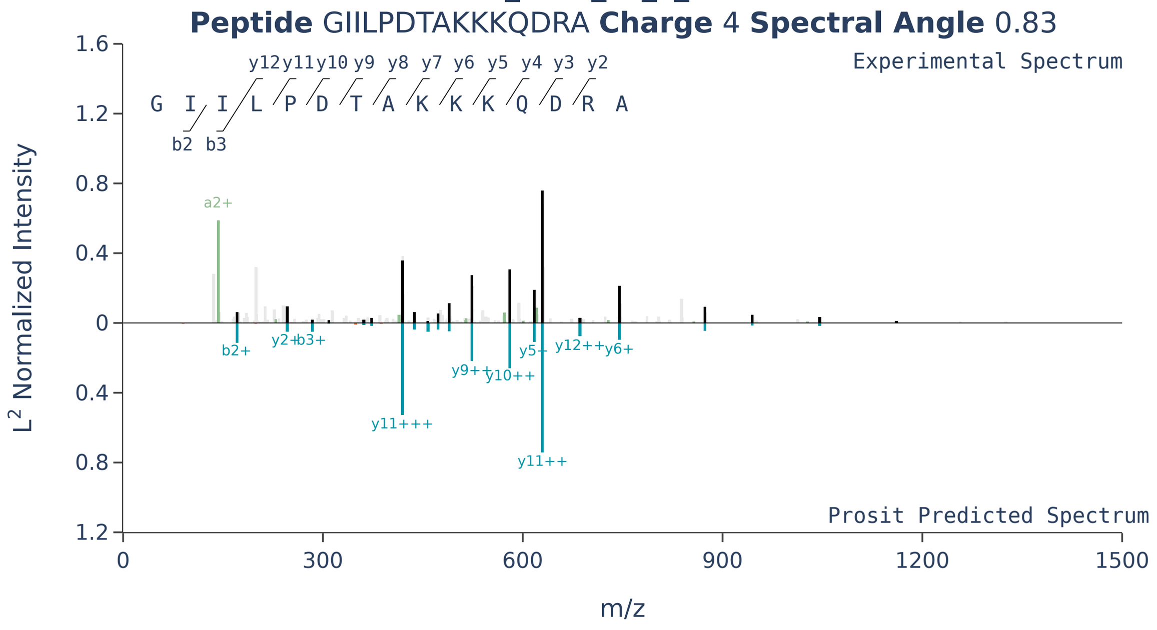

Source Fu_HeLa_inf_2_R2 **Scan** 7862
Peptide AAGFVVKRM **Charge** 2 **Spectral Angle** 0.94

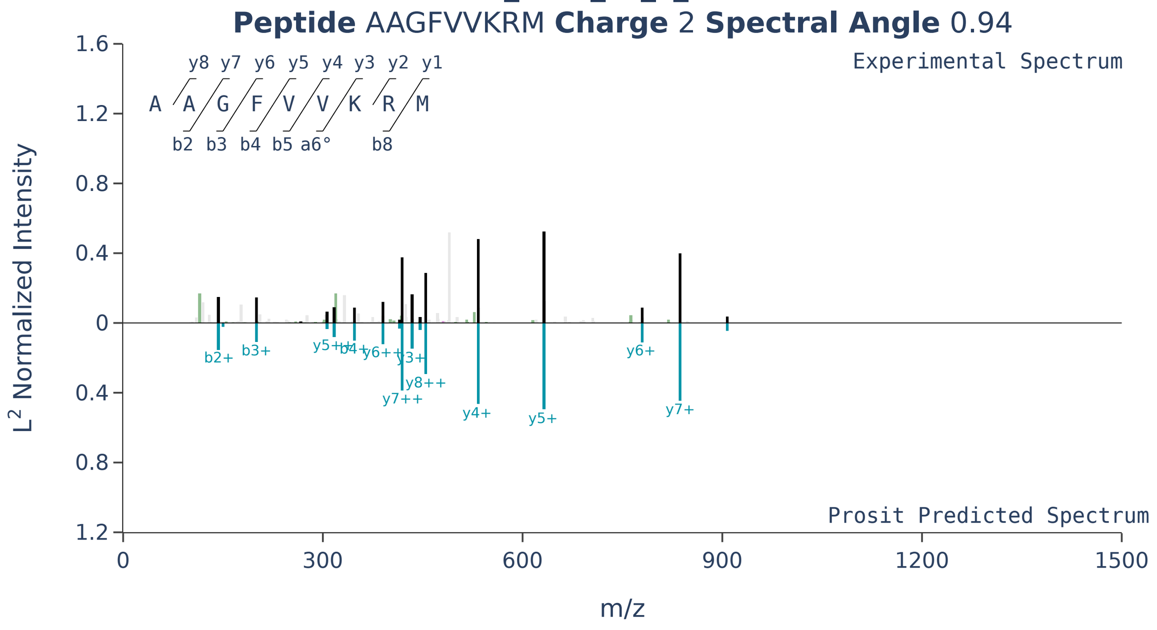

Source Fu_HeLa_inf_2_R2 **Scan** 2001
Peptide IRKISKPVQH **Charge** 3 **Spectral Angle** 0.84

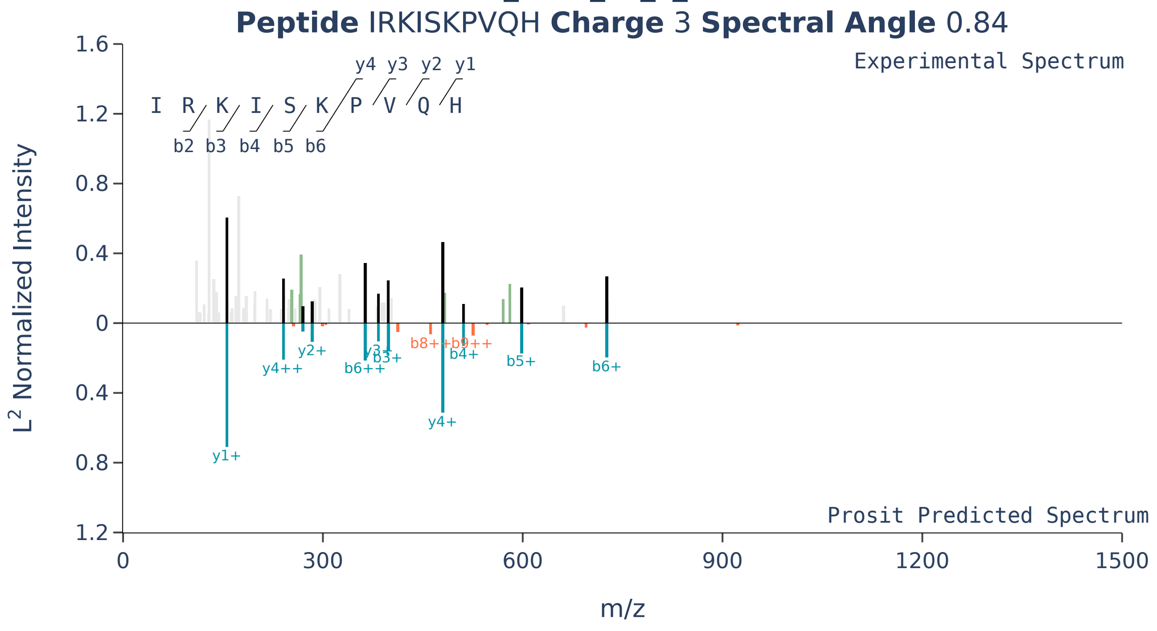

Source Fusion_Infected_HeLa_24h_PI_R2 **Scan** 3428
Peptide IRGISKKKKGIGL **Charge** 3 **Spectral Angle** 0.76

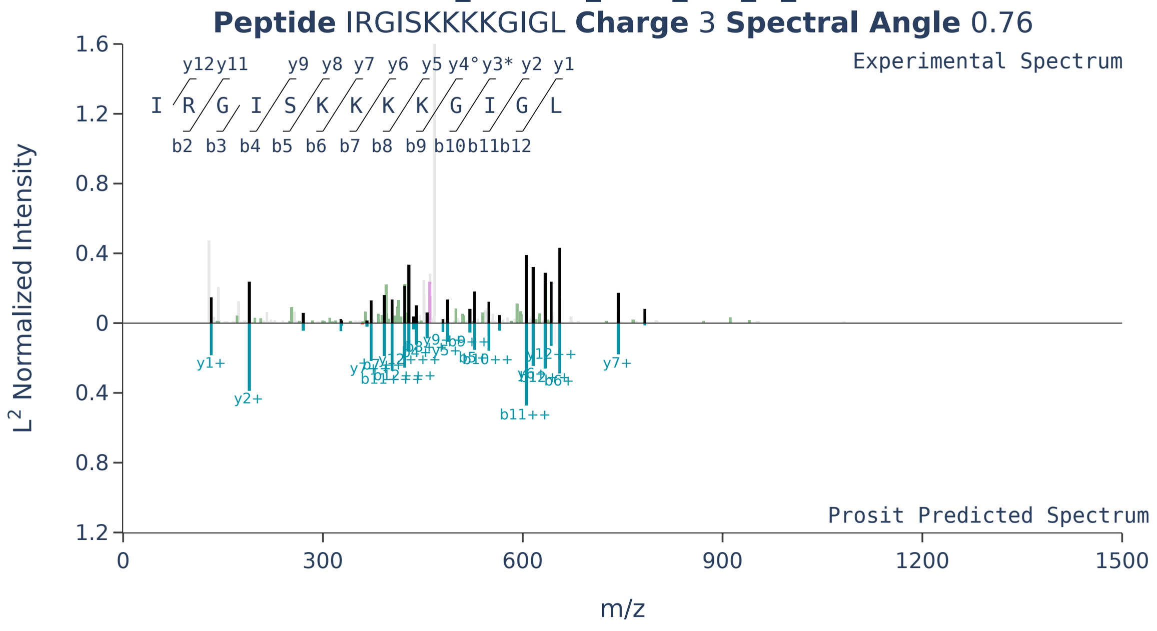

Source Fu_HeLa_inf_2_R1 **Scan** 1988
Peptide YVINKKDPNRKQ **Charge** 3 **Spectral Angle** 0.87

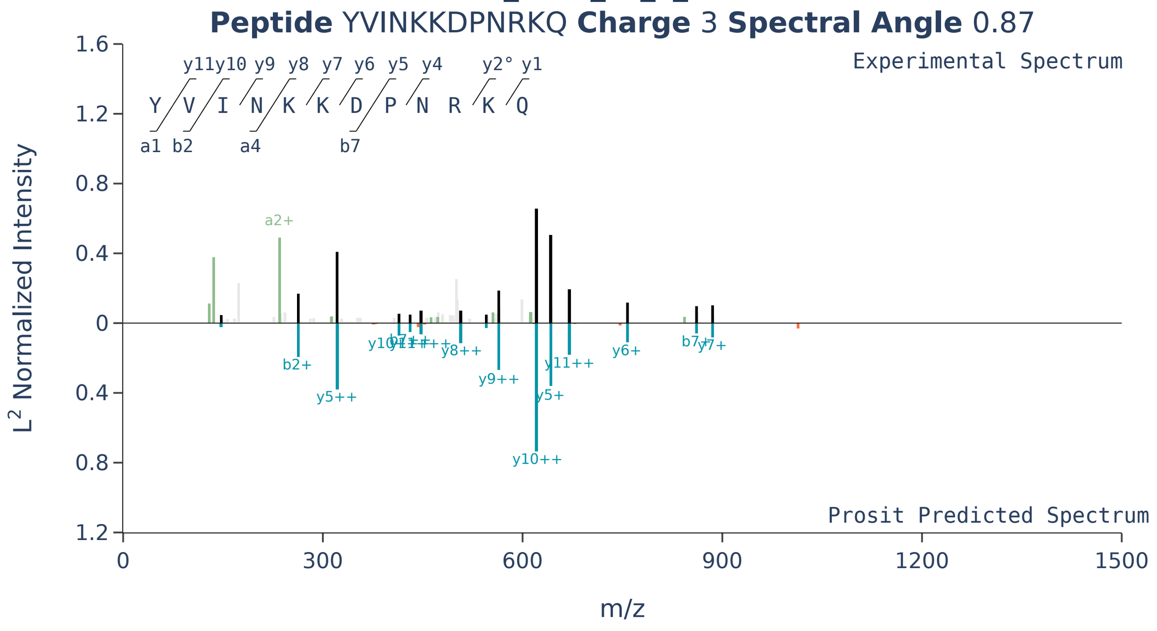

Source Fusion_Infected_HeLa_24h_PI_R2 **Scan** 6487
Peptide ESAERMVEGTA **Charge** 2 **Spectral Angle** 0.92

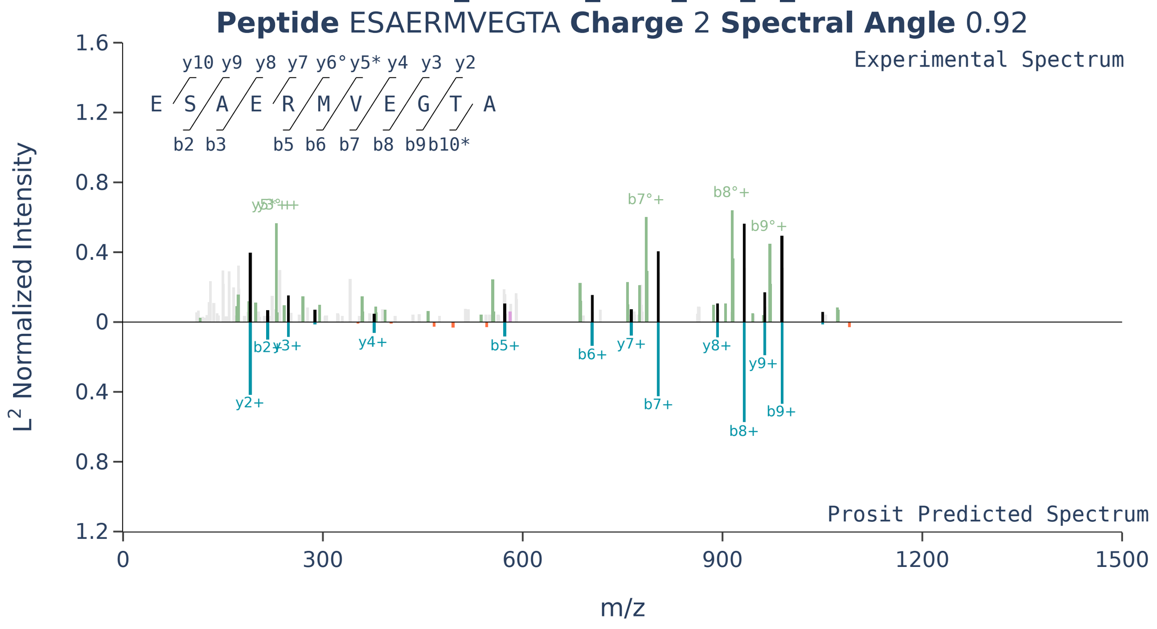

Source Fu_HeLa_inf_2_R1 **Scan** 13388
Peptide EAIYTEGLRNVTA **Charge** 2 **Spectral Angle** 0.89

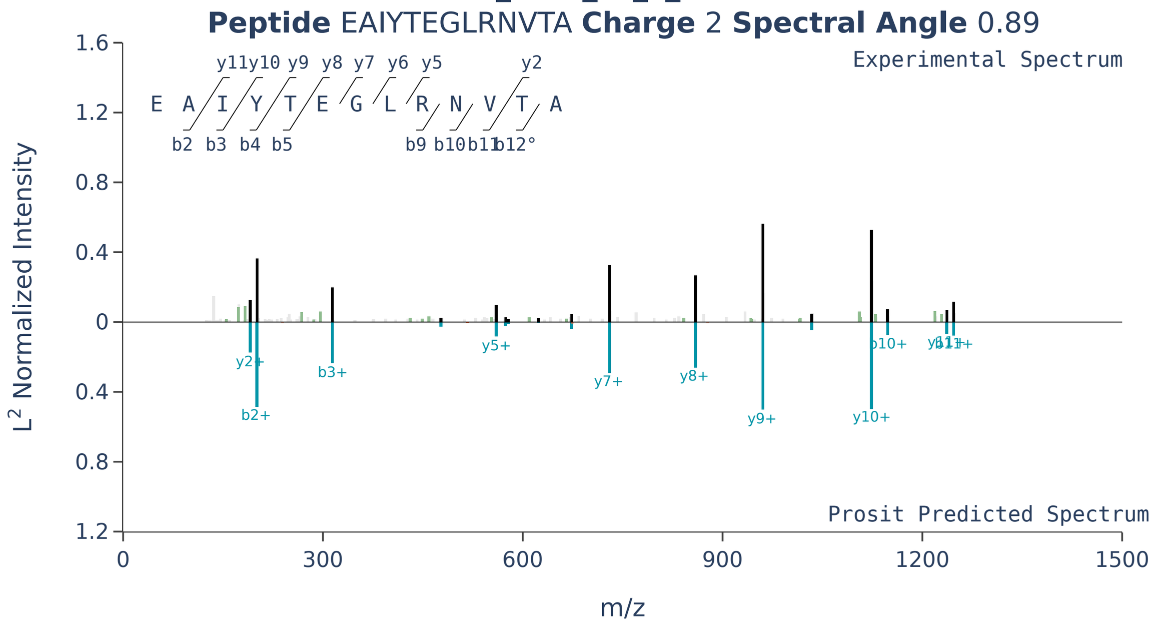

Source Fu_HeLa_inf_2_R1 **Scan** 3945
Peptide ESASKTLSQTA **Charge** 2 **Spectral Angle** 0.9

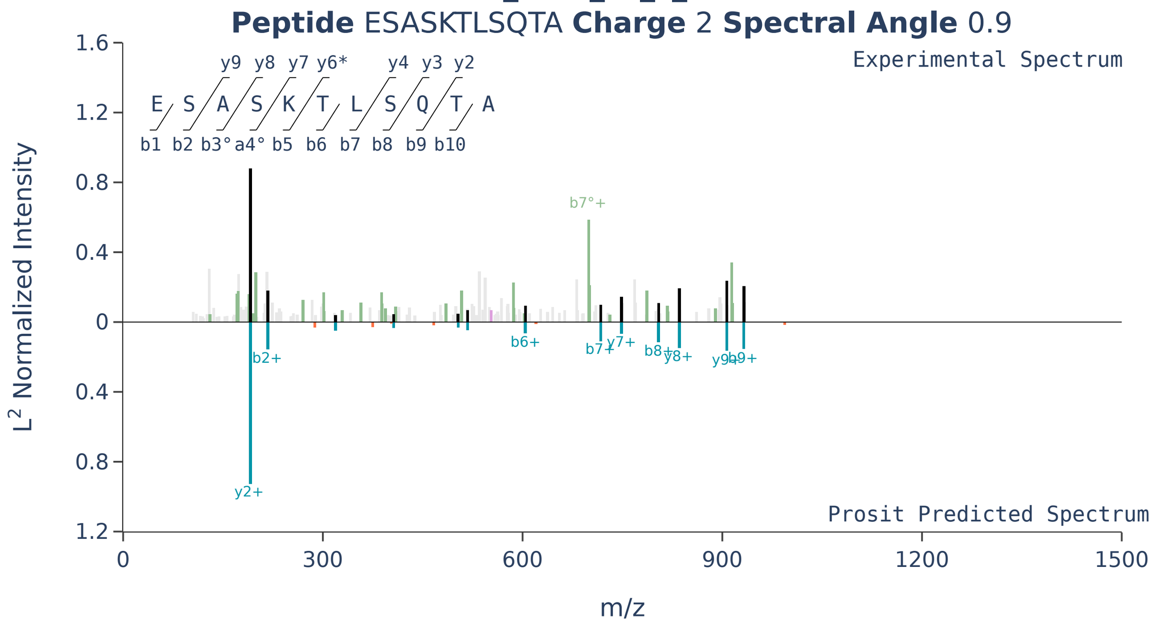

Source Fu_HeLa_inf_2_R2 **Scan** 11021
Peptide IYTEGLRNVTA **Charge** 2 **Spectral Angle** 0.95

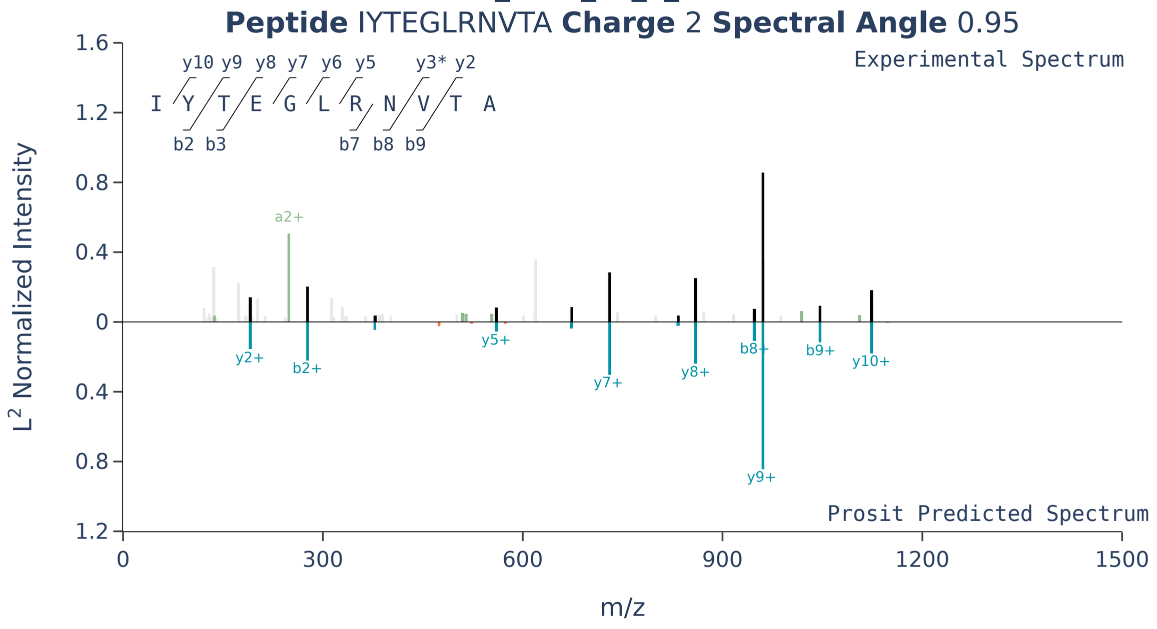

Source Fusion_Infected_HeLa_24h_PI_R2 **Scan** 1836
Peptide IRGISKKKKGIG **Charge** 3 **Spectral Angle** 0.82

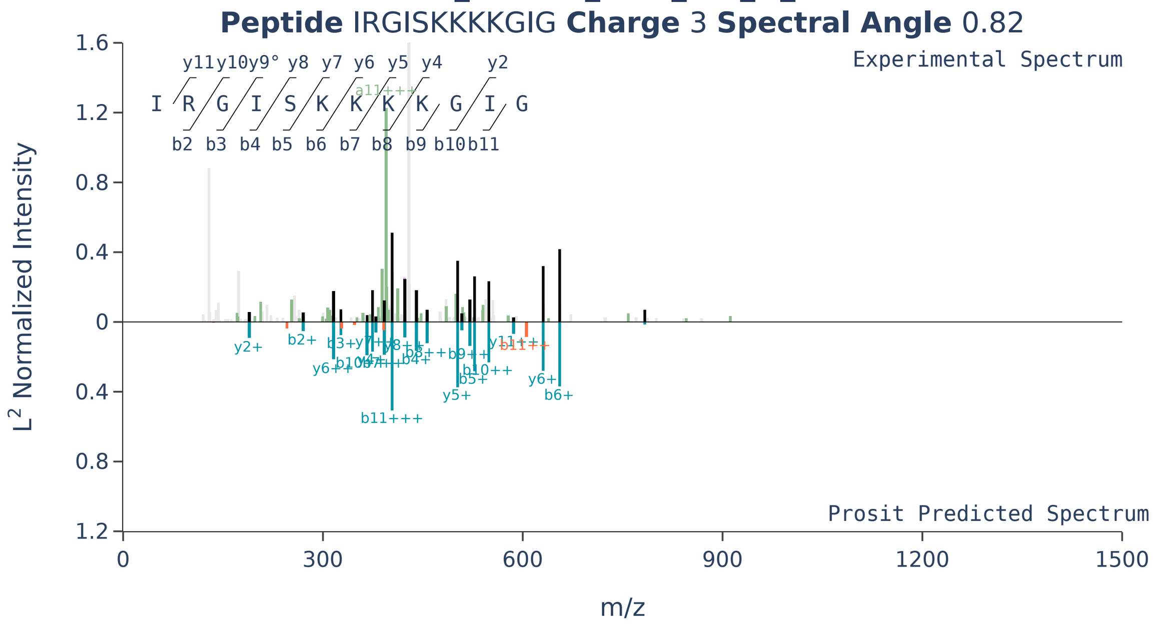

Source Fu_HeLa_inf_2_R2 **Scan** 1746
Peptide TAKKAPVRKVAA **Charge** 3 **Spectral Angle** 0.71

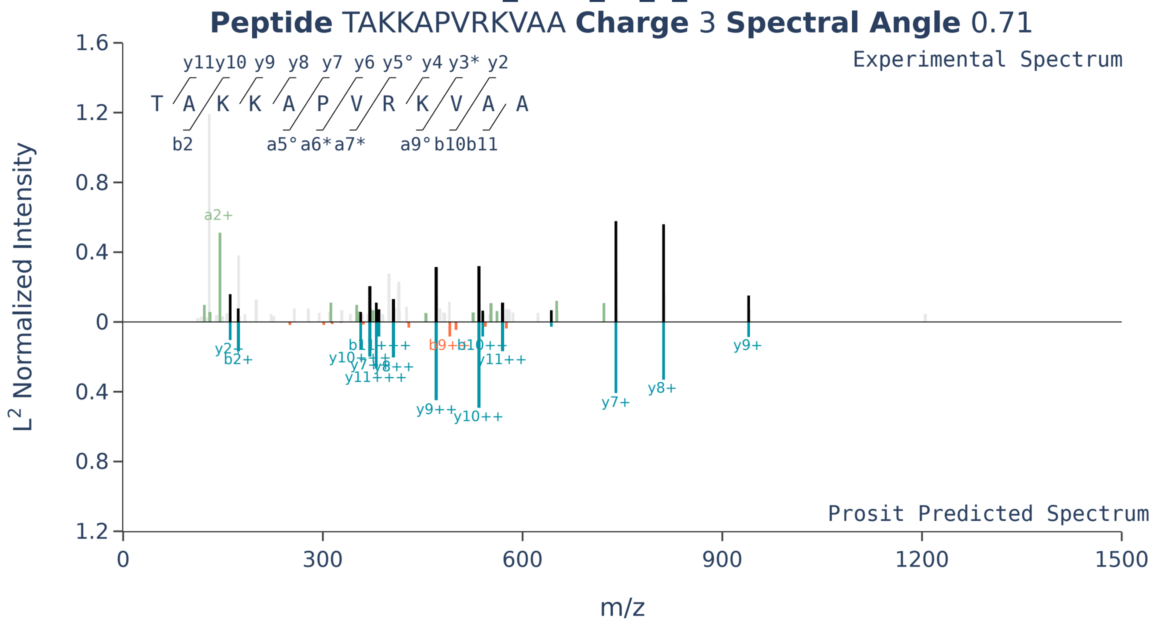

Source Fu_HeLa_inf_2_R1 **Scan** 1956
Peptide TISQDHKIHPNH **Charge** 3 **Spectral Angle** 0.88

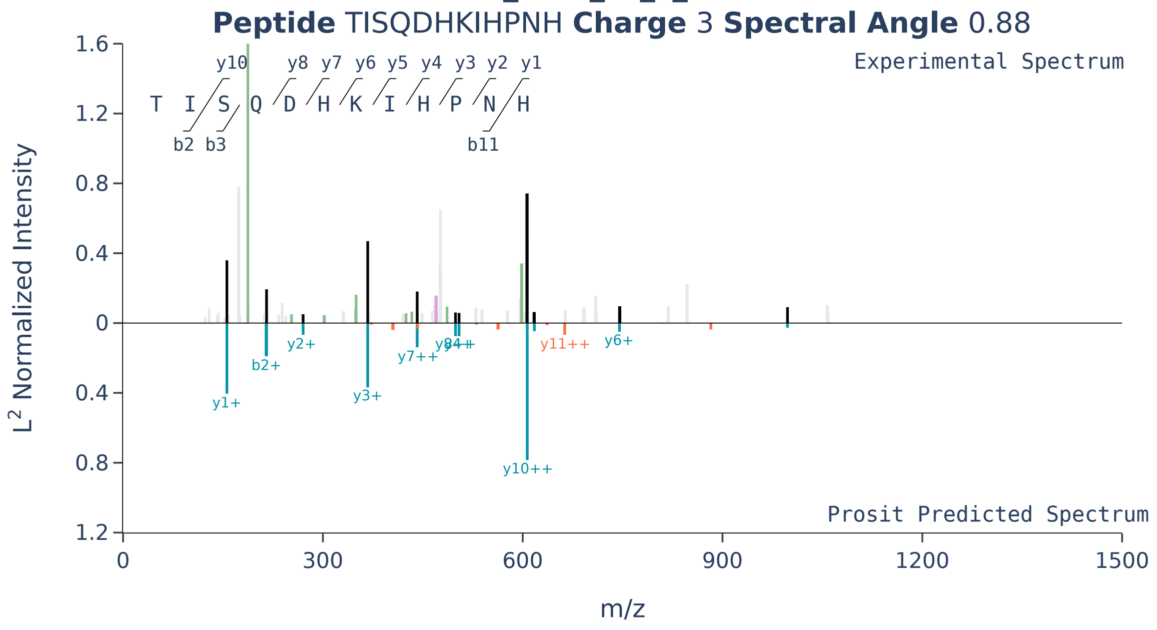

Source Fu_HeLa_inf_2_R1 **Scan** 5740
Peptide SKETFQRNKPHINIG **Charge** 3 **Spectral Angle** 0.8

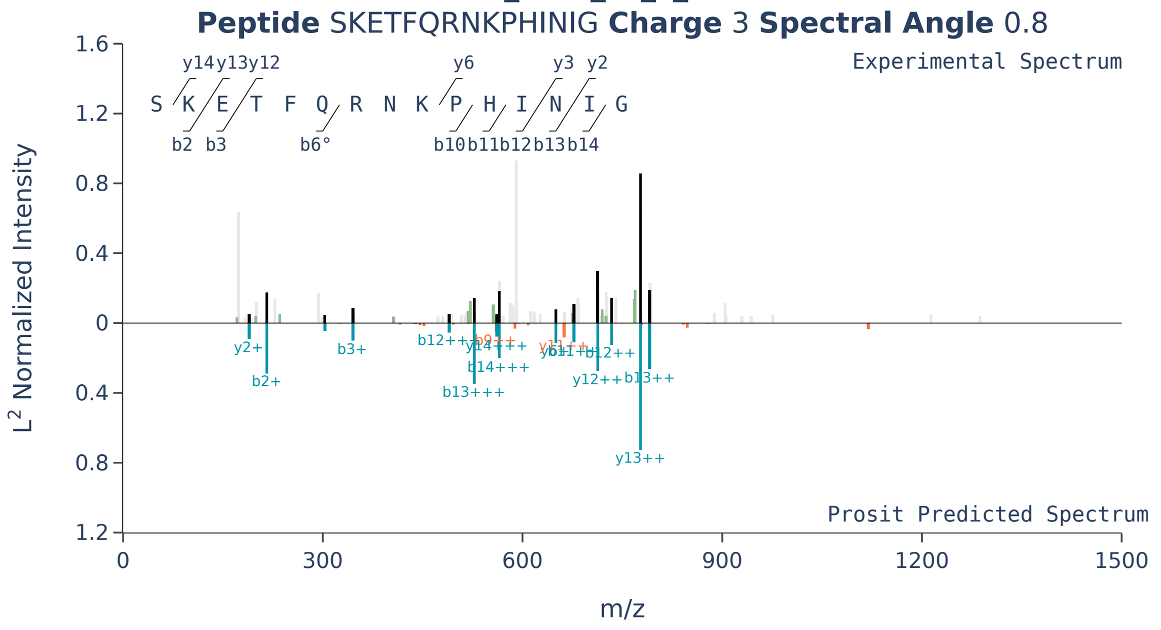

Source Fu_HeLa_inf_2_R1 **Scan** 7363
Peptide HTFEVHNGRKFL **Charge** 3 **Spectral Angle** 0.82

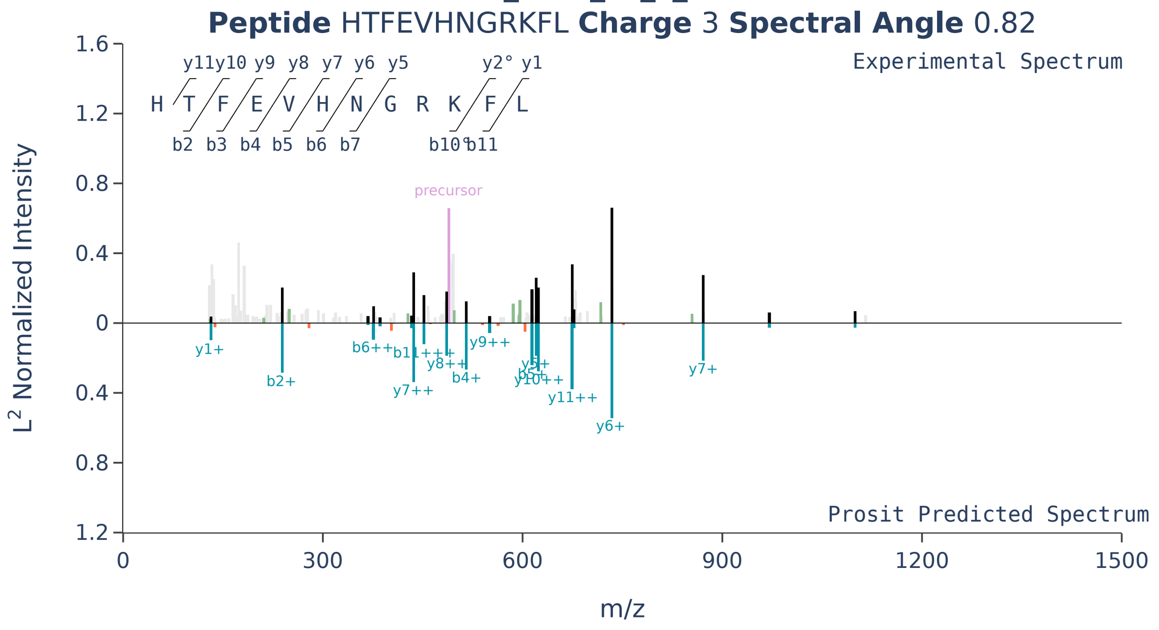

Source Fusion_Infected_HeLa_24h_PI_R1 **Scan** 3629
Peptide TVAAQDAAKAA **Charge** 2 **Spectral Angle** 0.84

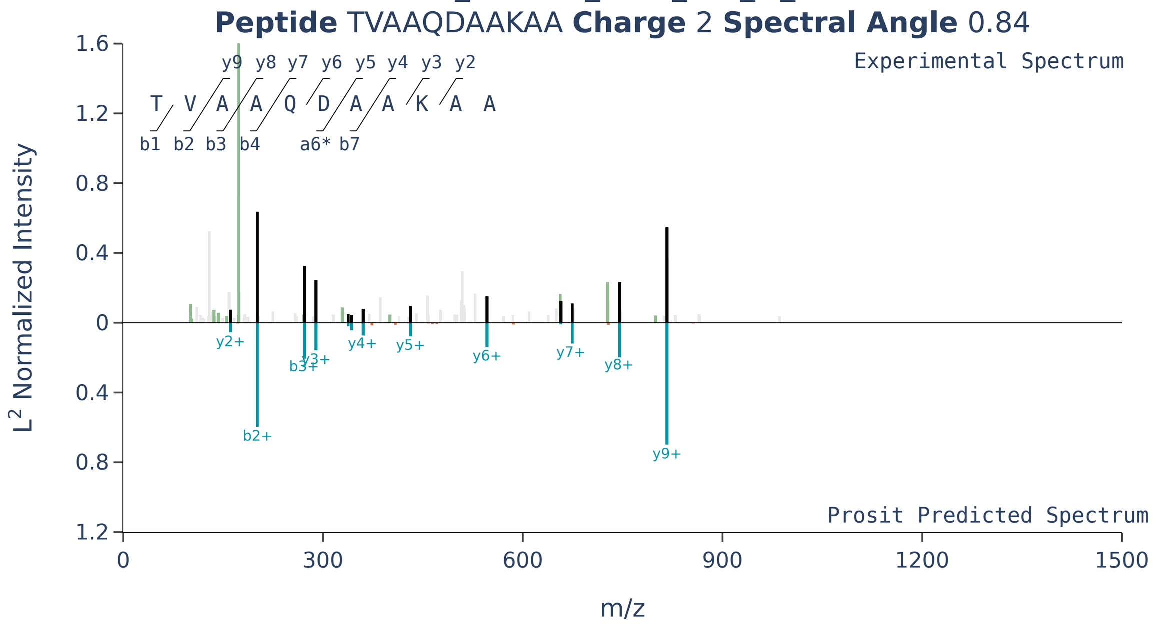

Source Fusion_Infected_HeLa_24h_PI_R2 **Scan** 5217
Peptide EAVKVTLGPKGRHV **Charge** 3 **Spectral Angle** 0.63

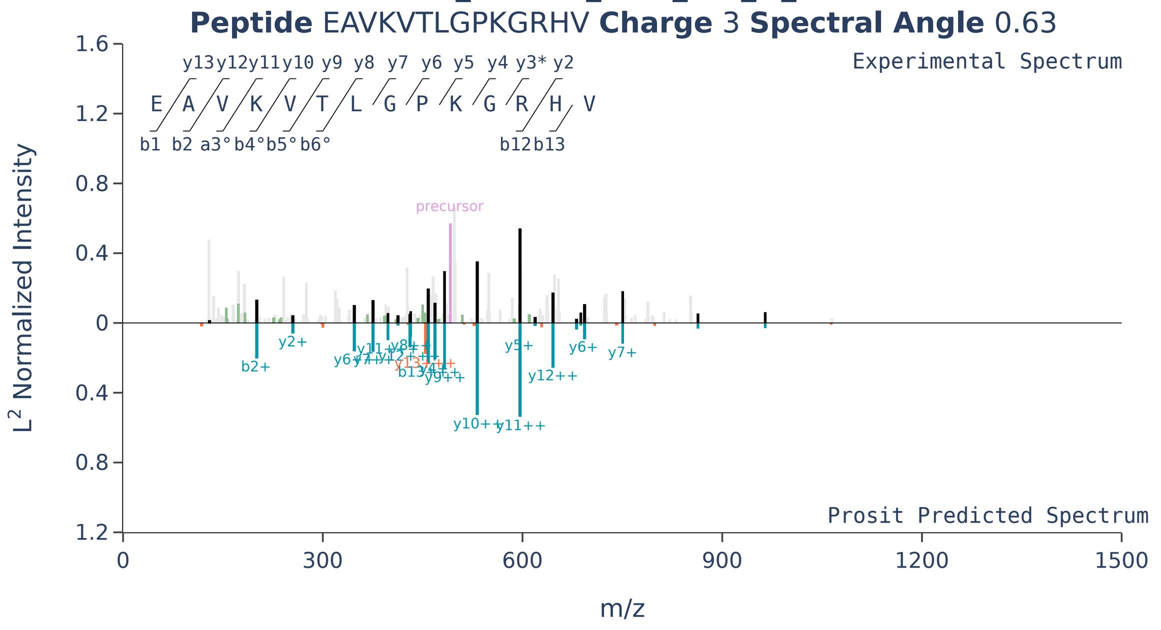

Source Fu_HeLa_inf_2_R2 **Scan** 7026
Peptide ETFQRNKPHINIG **Charge** 3 **Spectral Angle** 0.87

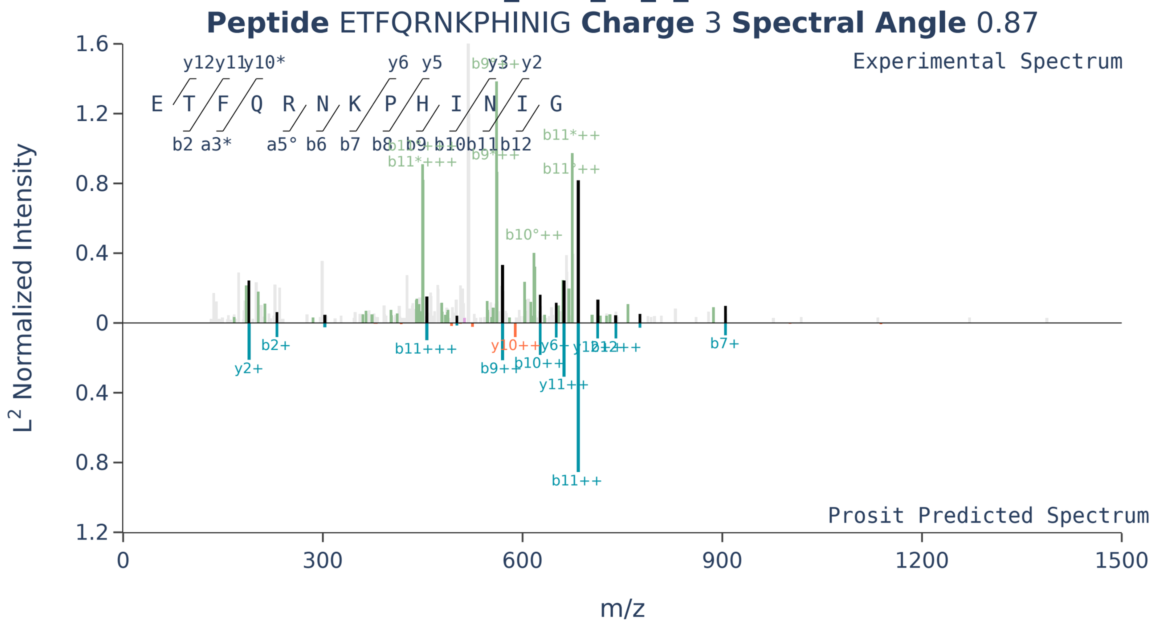

Source Fu_HeLa_inf_2_R1 **Scan** 4300
Peptide ETFQRNKPHI **Charge** 3 **Spectral Angle** 0.89

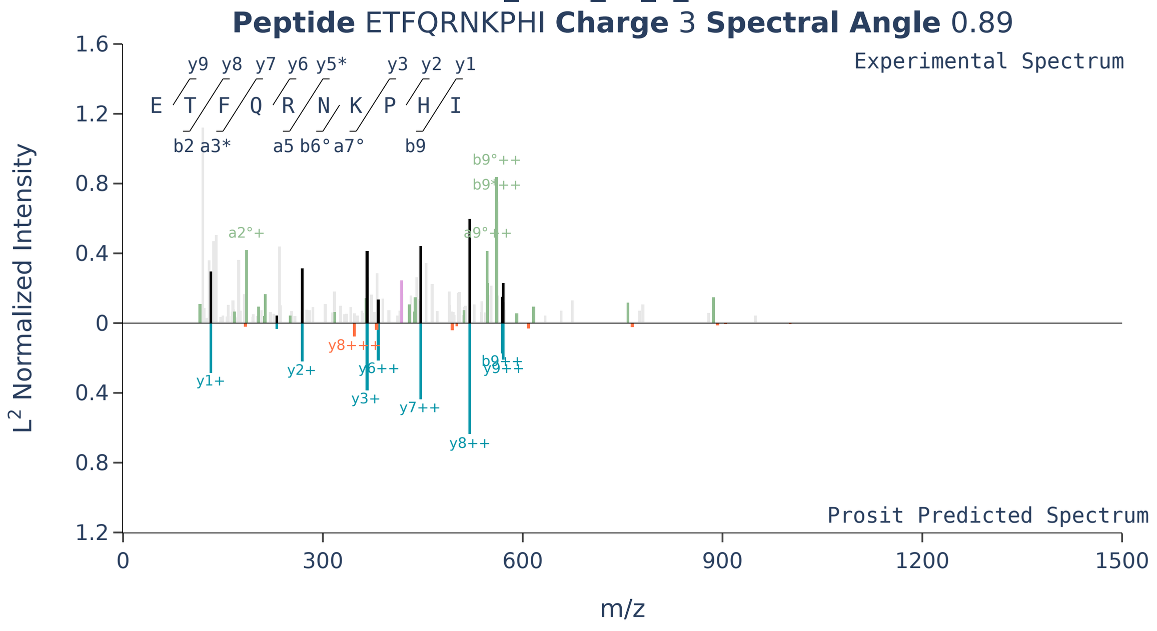

Source Fusion_Infected_HeLa_24h_PI_R1 **Scan** 12474
Peptide LSGDGLADFRD **Charge** 2 **Spectral Angle** 0.85

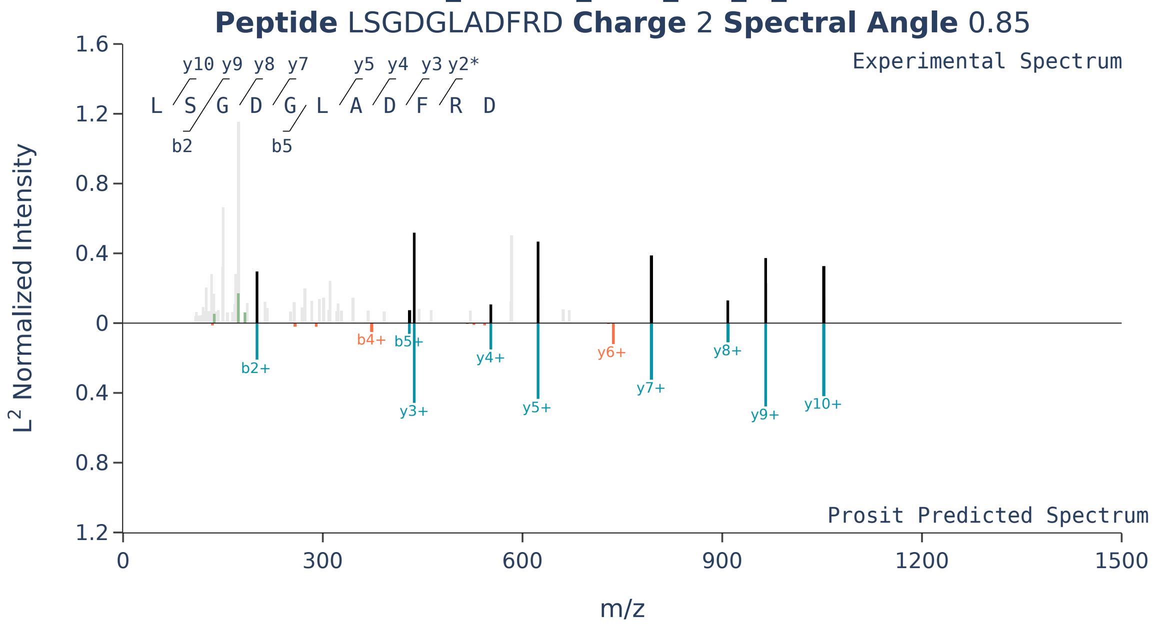

Source Fu_HeLa_inf_2_R2 **Scan** 10106
Peptide PIVQESSSKESPLPS **Charge** 2 **Spectral Angle** 0.78

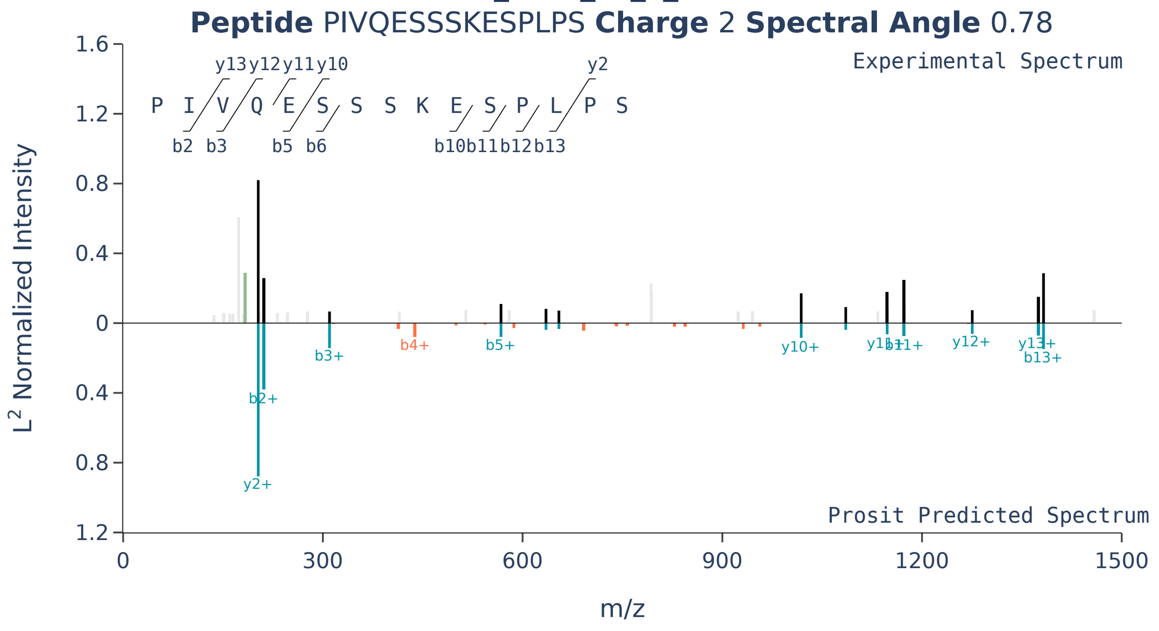

Source Fu_HeLa_inf_2_R1 **Scan** 6229
Peptide VTNPEKTLA **Charge** 2 **Spectral Angle** 0.93

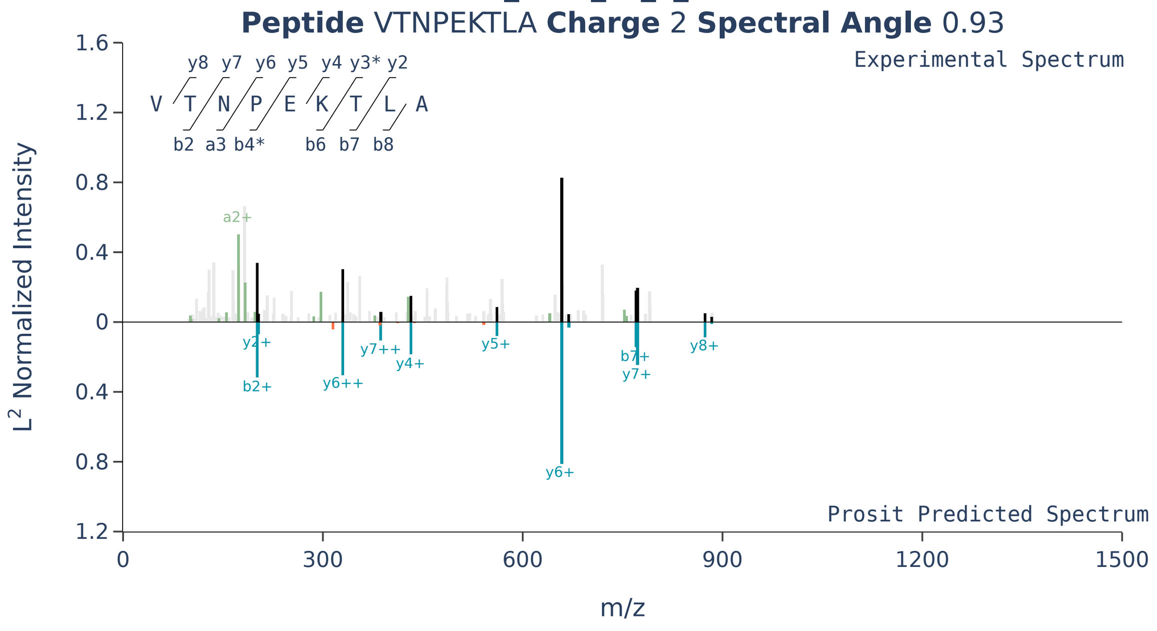

Source Fu_HeLa_inf_2_R2 **Scan** 3665
Peptide RVYSHPQYA **Charge** 2 **Spectral Angle** 0.95

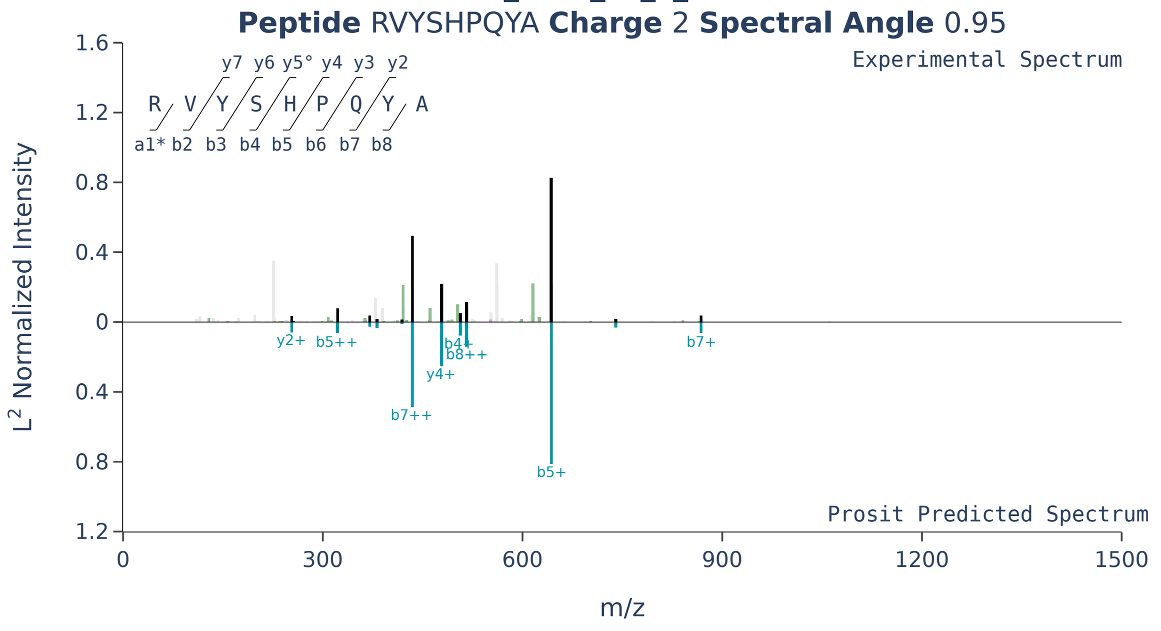

Source Fu_HeLa_inf_2_R1 **Scan** 7716
Peptide ETFQRNKPHINI **Charge** 2 **Spectral Angle** 0.8

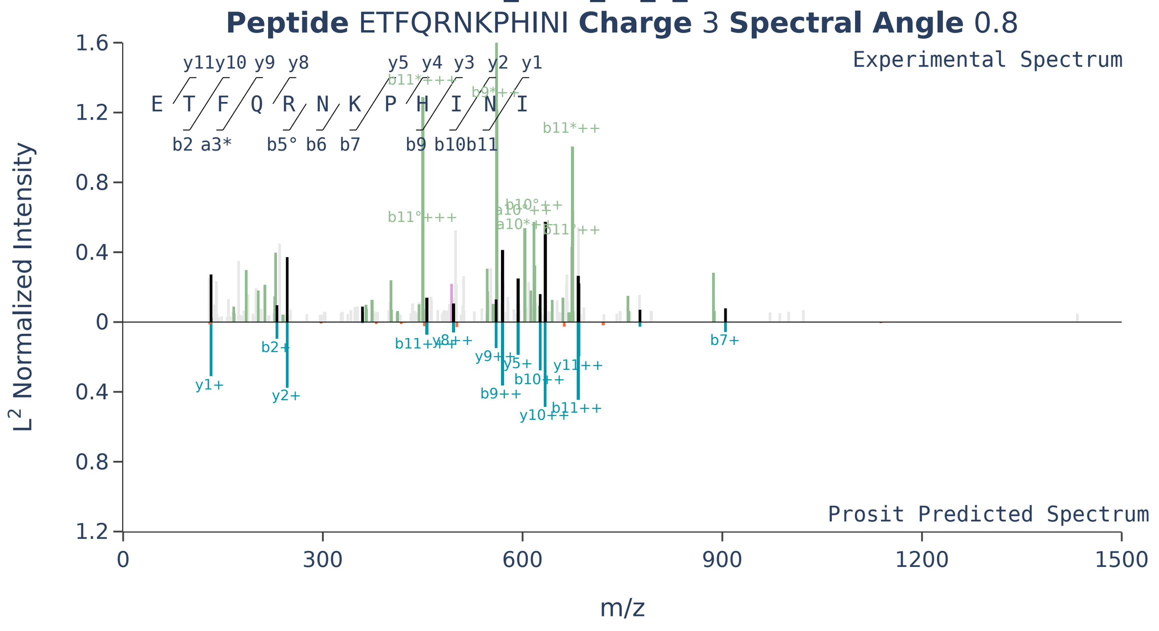

Source Fu_HeLa_inf_2_R2 **Scan** 5908
Peptide TAKKATGLL **Charge** 2 **Spectral Angle** 0.9

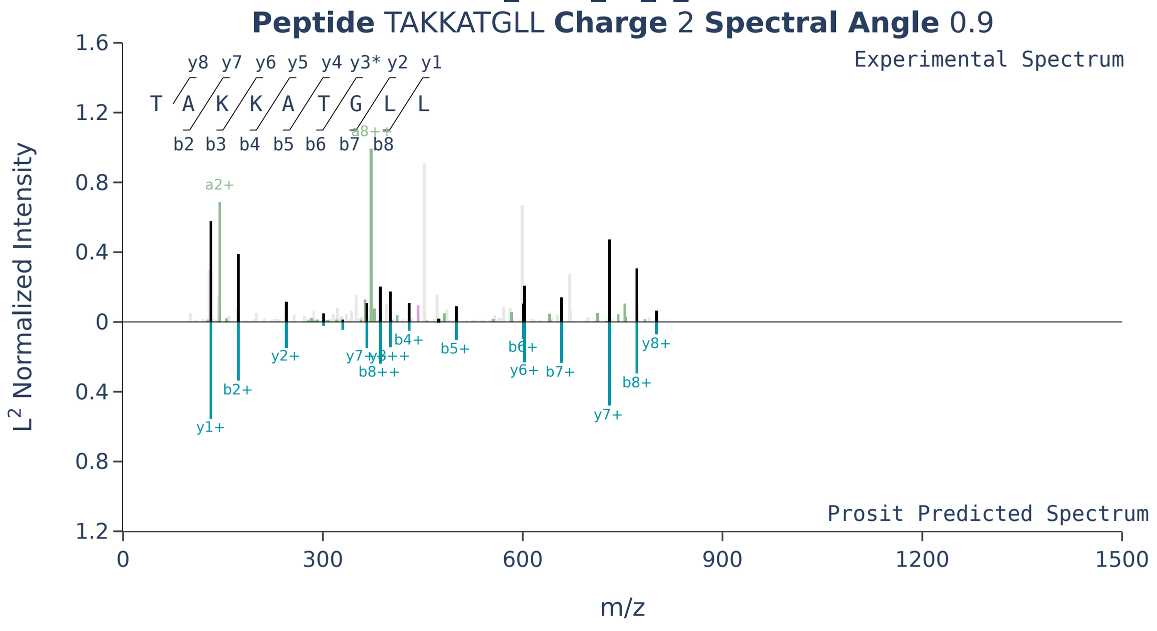

Source Fusion_Infected_HeLa_24h_PI_R2 **Scan** 5899
Peptide AITRALSGD **Charge** 2 **Spectral Angle** 0.93

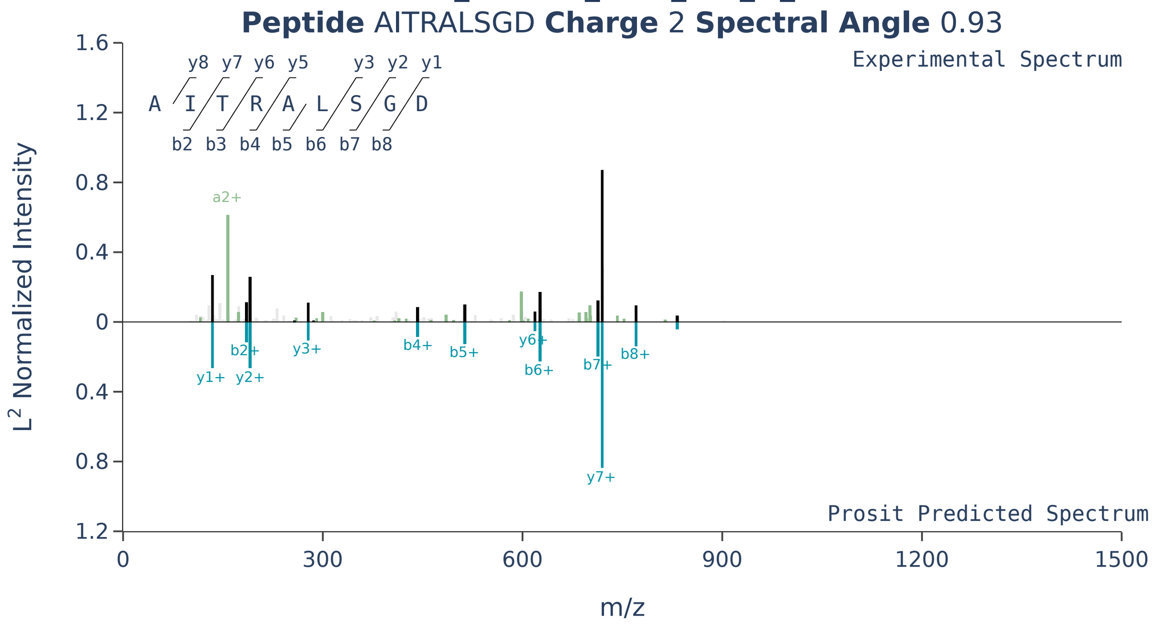

Source Fu_HeLa_inf_2_R1 **Scan** 11858
Peptide EMAGVKDIVA **Charge** 2 **Spectral Angle** 0.9

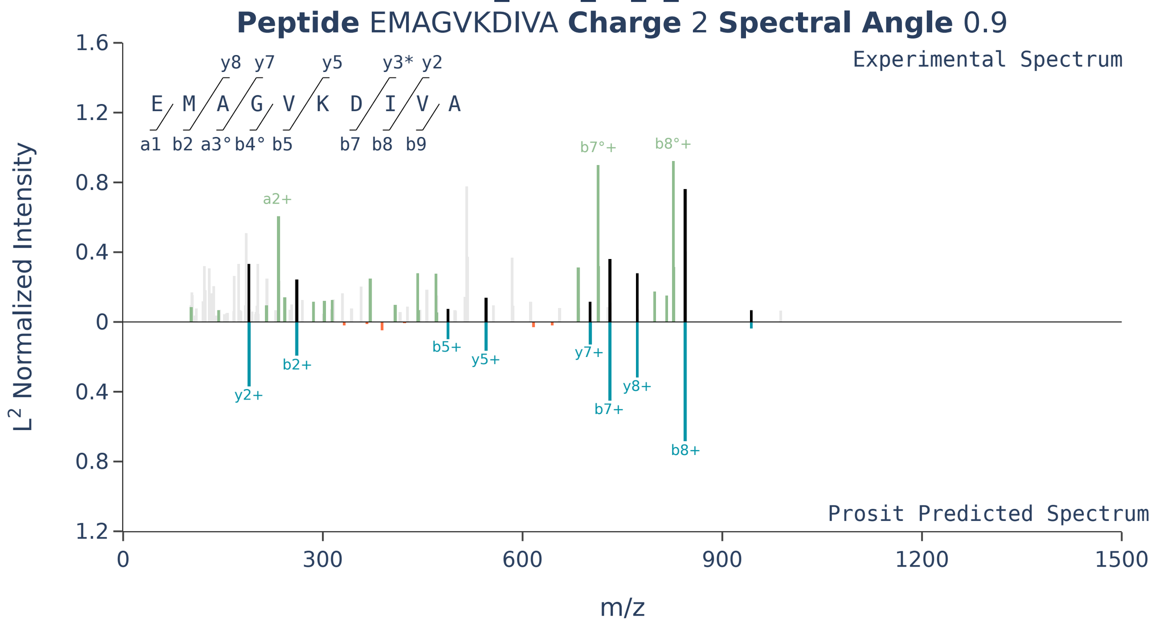

Source Fu_HeLa_inf_2_R2 **Scan** 13372
Peptide FASPKLNTL **Charge** 2 **Spectral Angle** 0.95

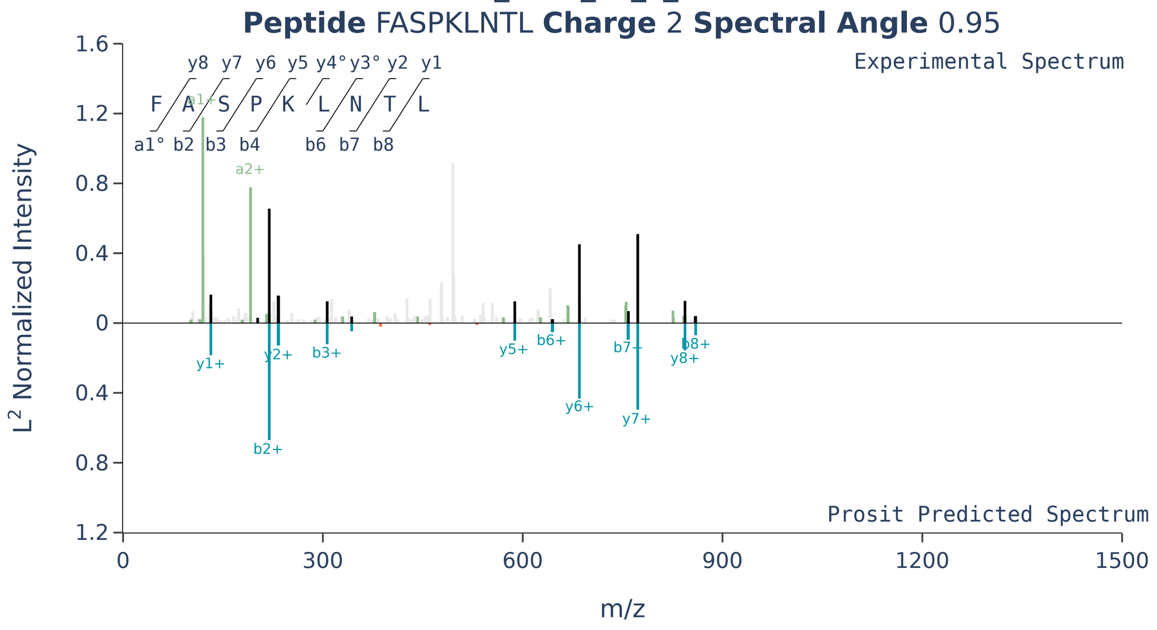

Source Fu_HeLa_inf_2_R1 **Scan** 8333
Peptide GVDIKIKAA **Charge** 2 **Spectral Angle** 0.88

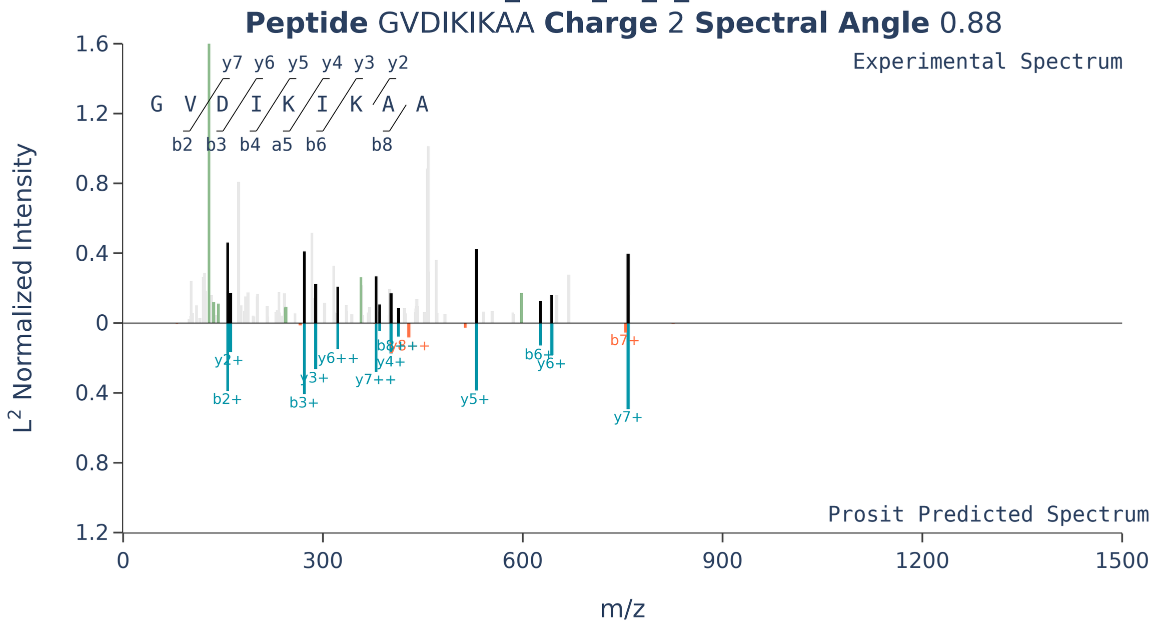

Source Fu_HeLa_inf_2_R2 **Scan** 4869
Peptide RLIETPTKSS **Charge** 2 **Spectral Angle** 0.89

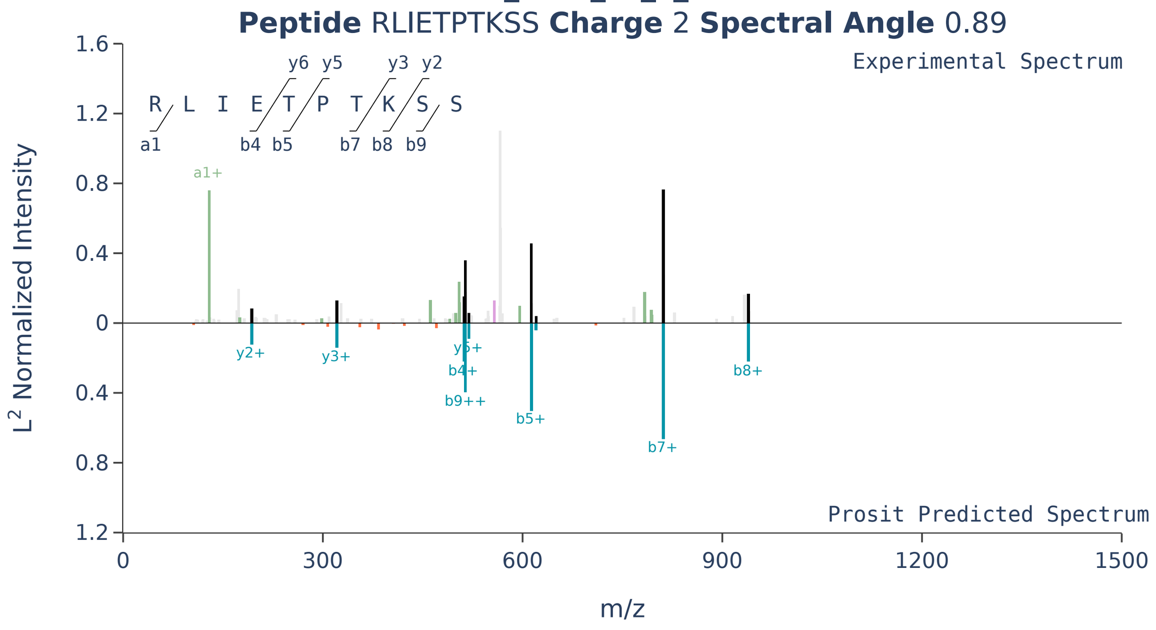

Source Fu_HeLa_inf_2_R1 **Scan** 7525
Peptide SVASRIHGL **Charge** 2 **Spectral Angle** 0.92

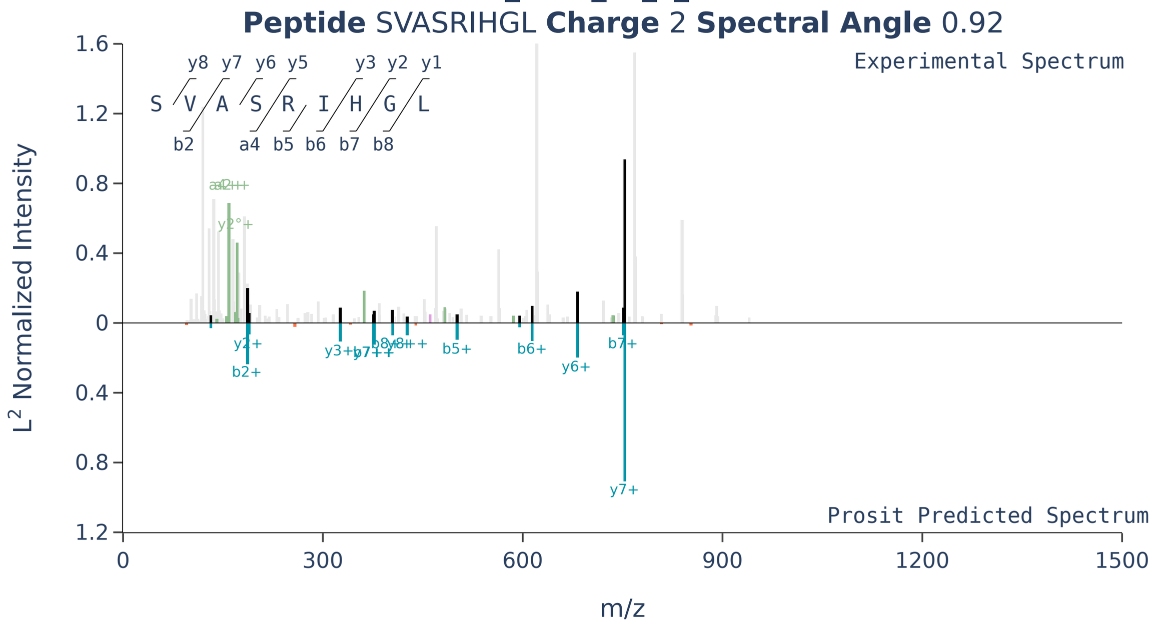

Source Fu_HeLa_inf_2_R1 **Scan** 8443
Peptide HTARPGLVIG **Charge** 2 **Spectral Angle** 0.9

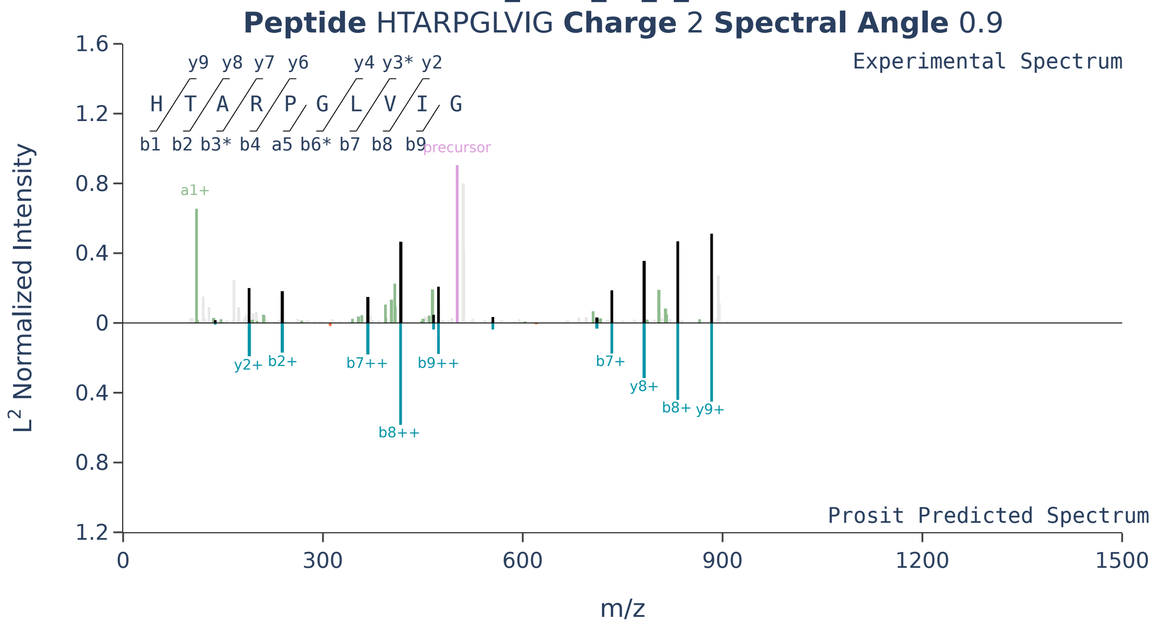

Source Fu_HeLa_inf_2_R1 **Scan** 5606
Peptide GANPMDLKRG **Charge** 3 **Spectral Angle** 0.91

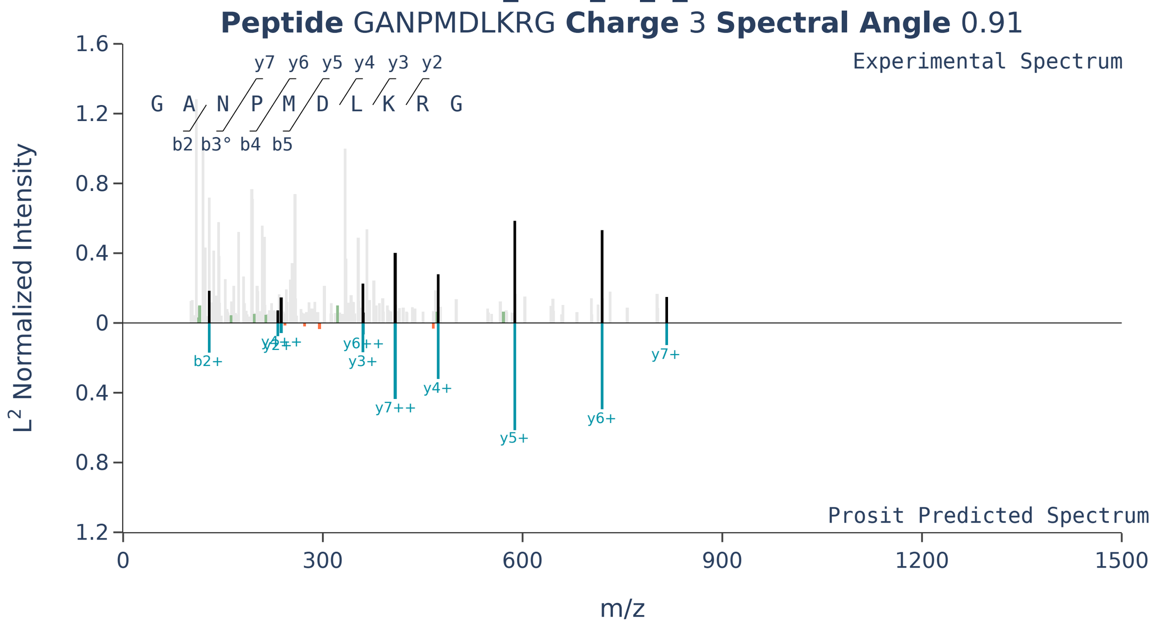

Source Fu_HeLa_inf_2_R1 **Scan** 11230
Peptide MAIHDPQGFA **Charge** 2 **Spectral Angle** 0.9

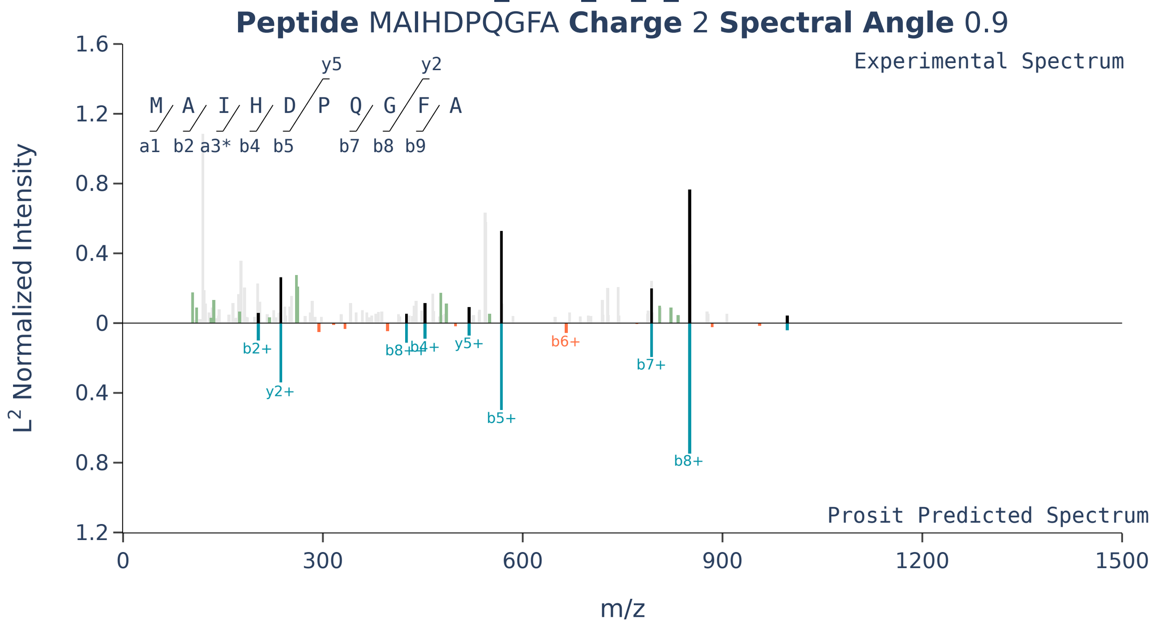

Source Fu_HeLa_inf_2_R1 **Scan** 1661
Peptide KYNEEARKKIQKG **Charge** 3 **Spectral Angle** 0.63

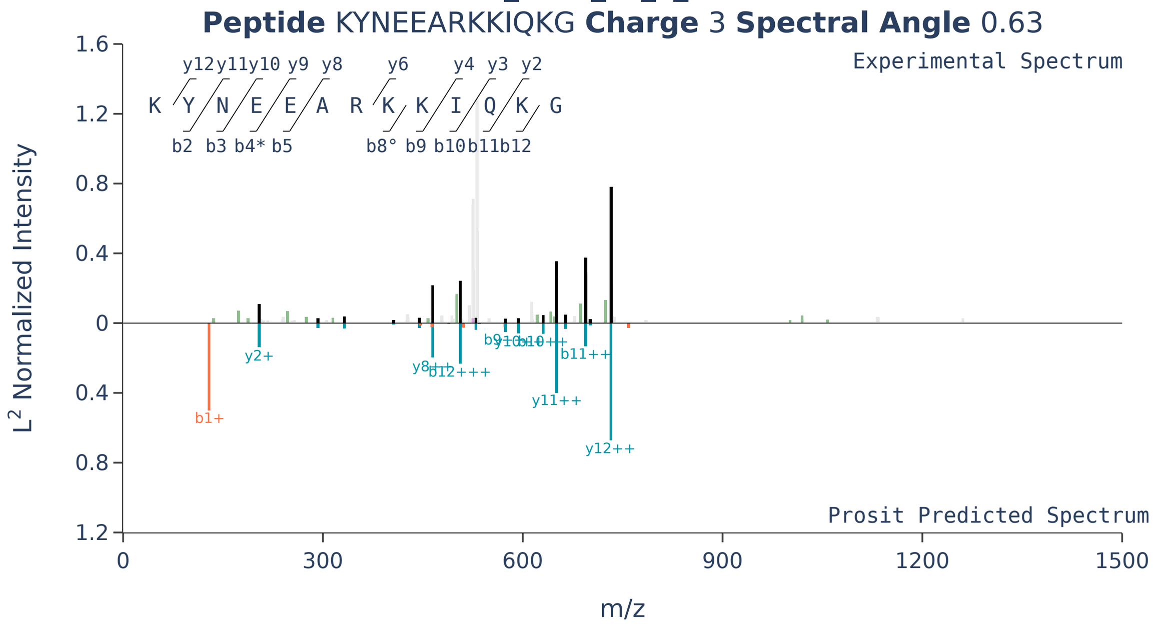

Source Fu_HeLa_inf_2_R2 **Scan** 8011
Peptide YTEGLRNVTA **Charge** 2 **Spectral Angle** 0.88

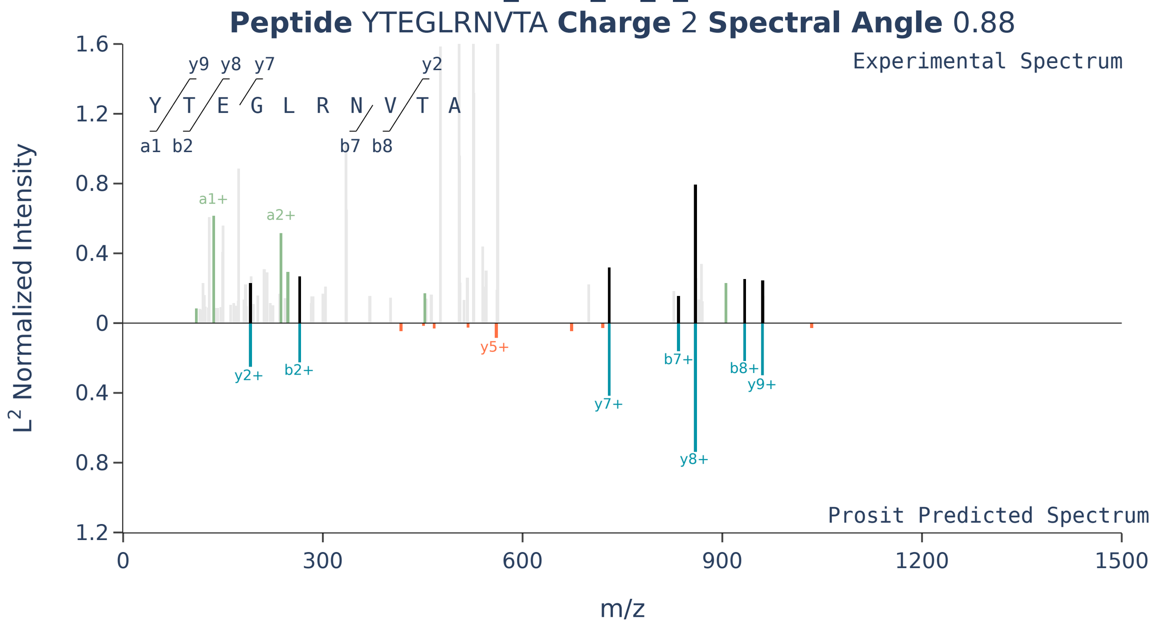

Source Fu_HeLa_inf_2_R2 **Scan** 8894
Peptide LPVGNPAE **Charge** 1 **Spectral Angle** 0.84

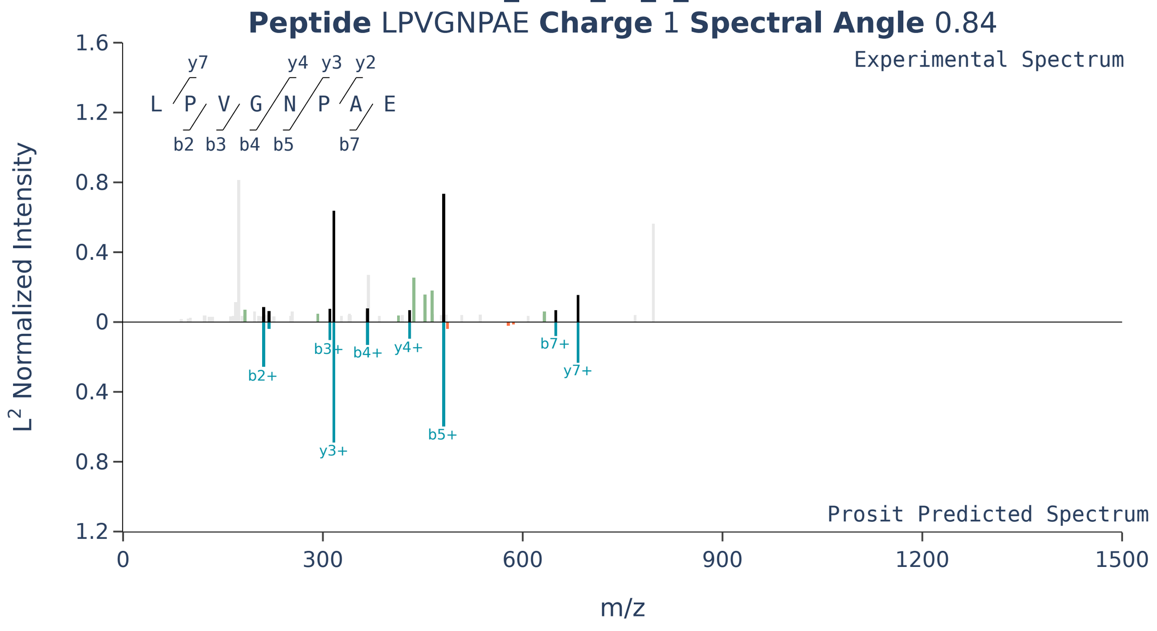

Source Fu_HeLa_inf_2_R2 **Scan** 8467
Peptide ADVEIVDKPE **Charge** 2 **Spectral Angle** 0.87

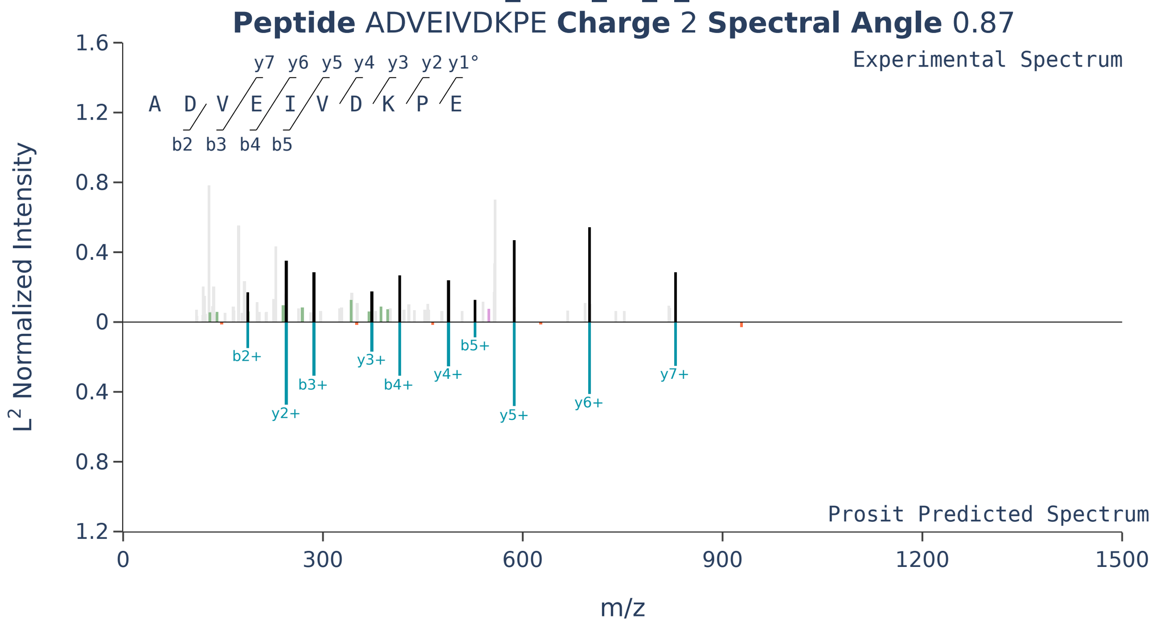

Source Fusion_Infected_HeLa_24h_PI_R1 **Scan** 2082
Peptide VIVRTRNDIHRKDG **Charge** 4 **Spectral Angle** 0.75

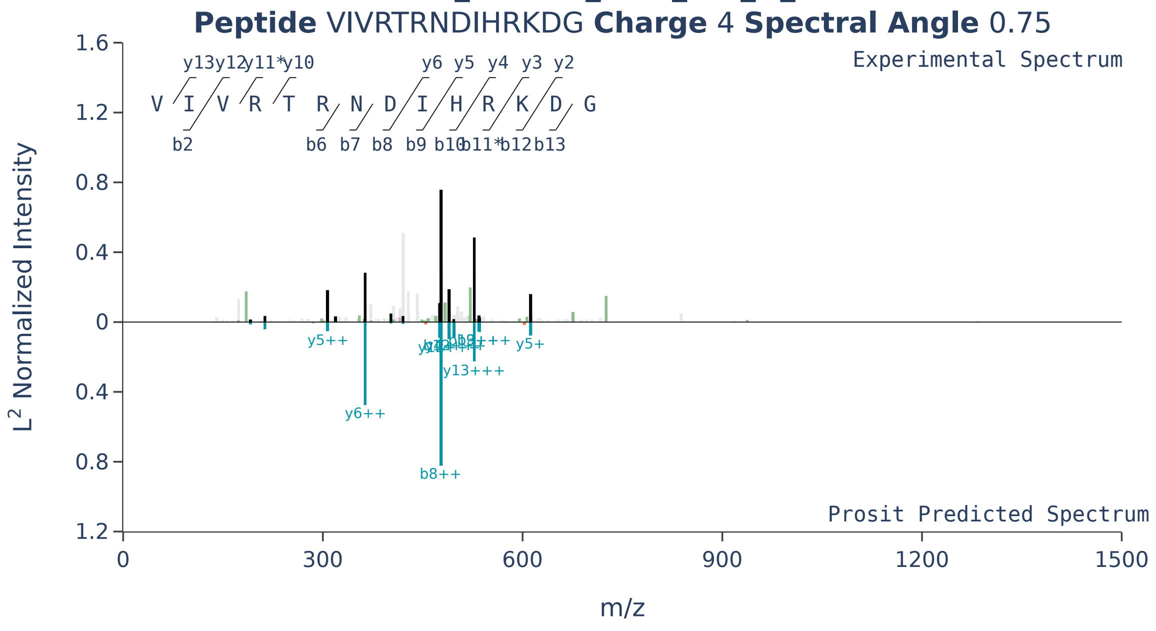

Source Fusion_Infected_HeLa_24h_PI_R2 **Scan** 3311
Peptide RAKNVGGEL **Charge** 2 **Spectral Angle** 0.91

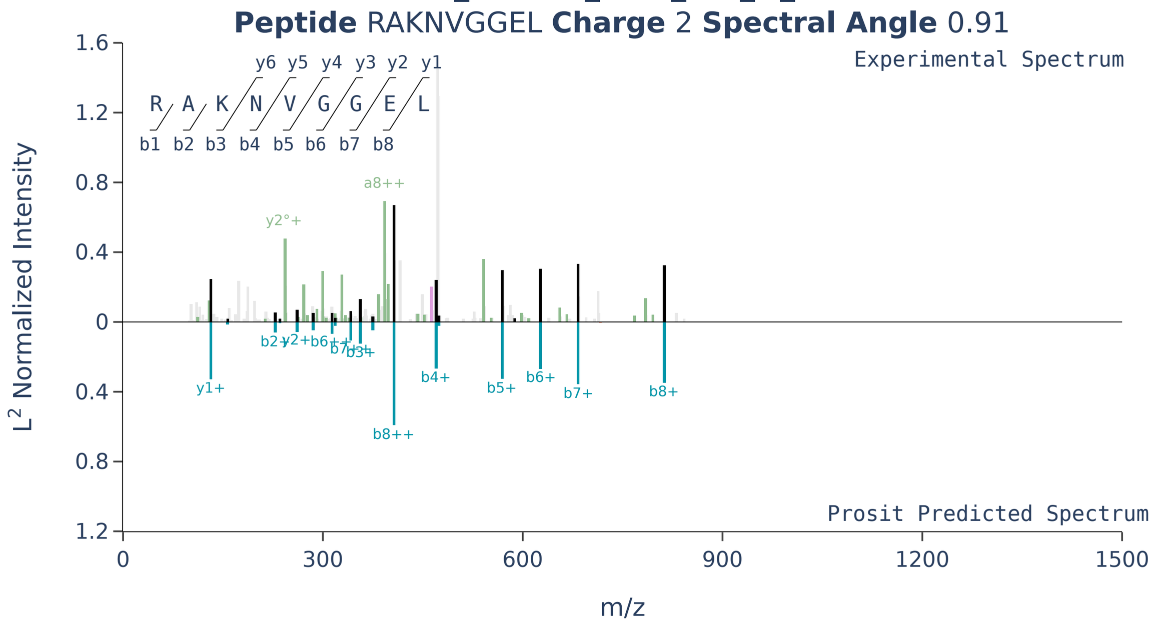

Source Fusion_Infected_HeLa_24h_PI_R2 **Scan** 7933
Peptide GLVAKSGGPLN **Charge** 2 **Spectral Angle** 0.75

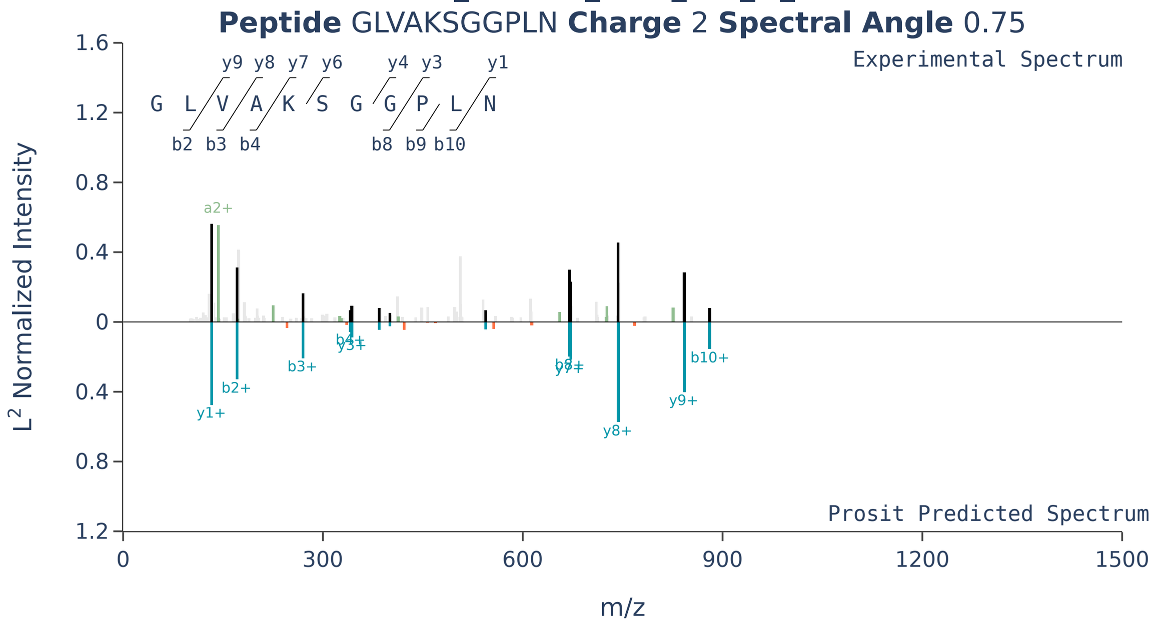

Source Fusion_Infected_HeLa_24h_PI_R2 **Scan** 6561
Peptide VDGIVVMKKTDRT **Charge** 3 **Spectral Angle** 0.73

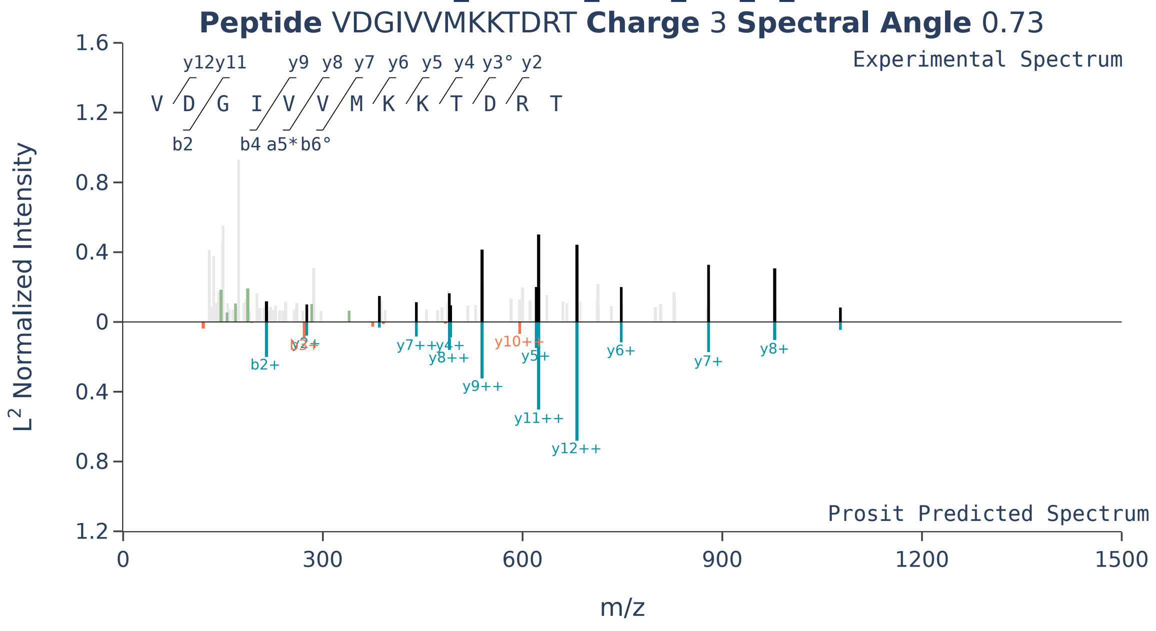

Source Fusion_Infected_HeLa_24h_PI_R2 **Scan** 10984
Peptide AITRALSGDGLAD **Charge** 2 **Spectral Angle** 0.8

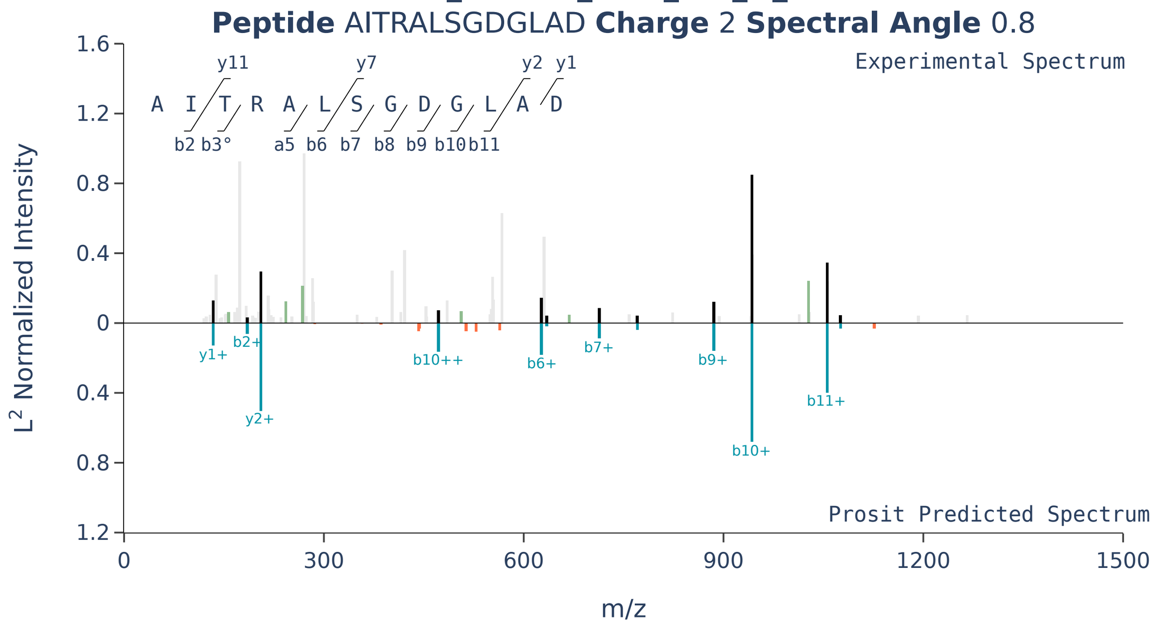

Source Fu_HeLa_inf_2_R2 **Scan** 2191
Peptide KNIKYNEEARKKIQ **Charge** 4 **Spectral Angle** 0.62

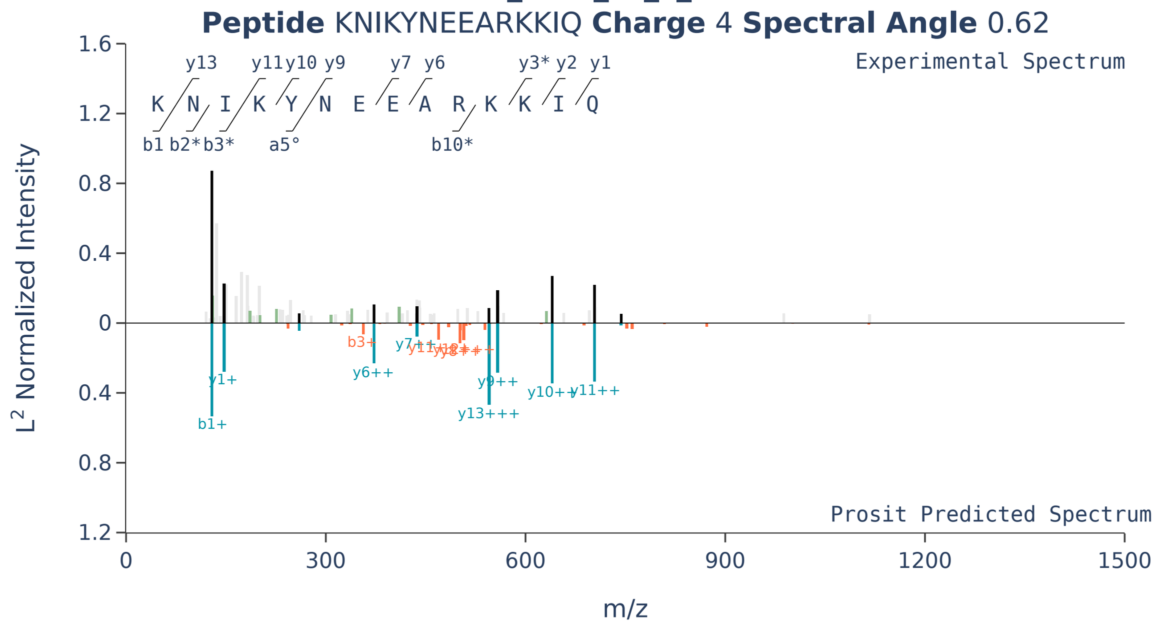

Source Fu_HeLa_inf_2_R1 **Scan** 8403
Peptide HATGIGLSA **Charge** 1 **Spectral Angle** 0.81

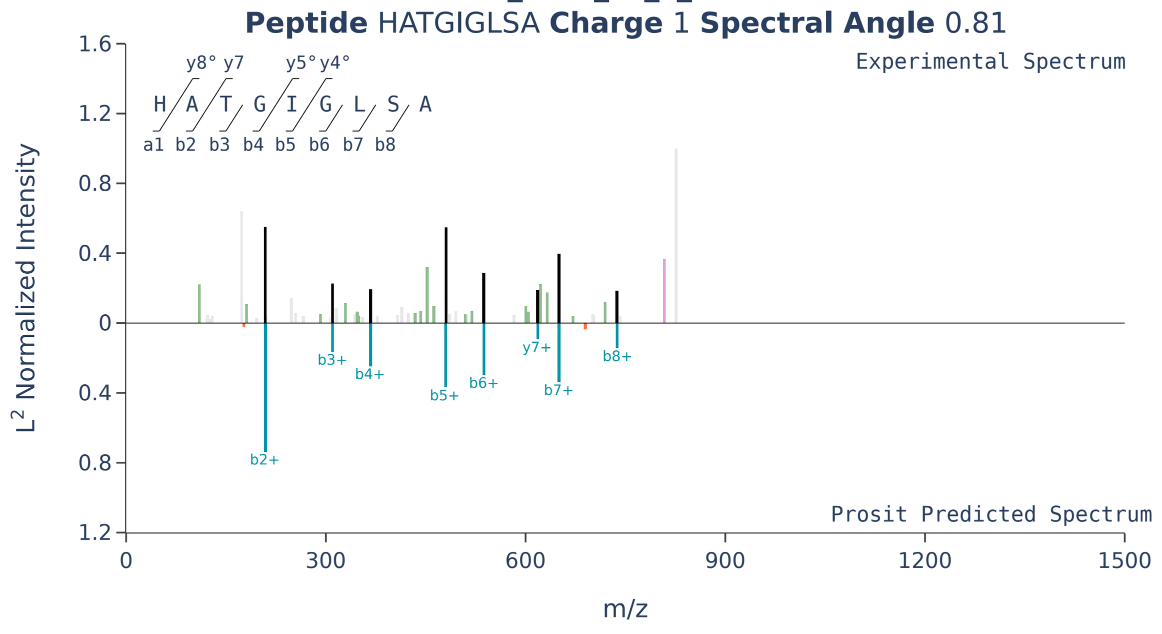

Source Fusion_Infected_HeLa_24h_PI_R2 **Scan** 12914
Peptide VVATQAKLALEA **Charge** 2 **Spectral Angle** 0.86

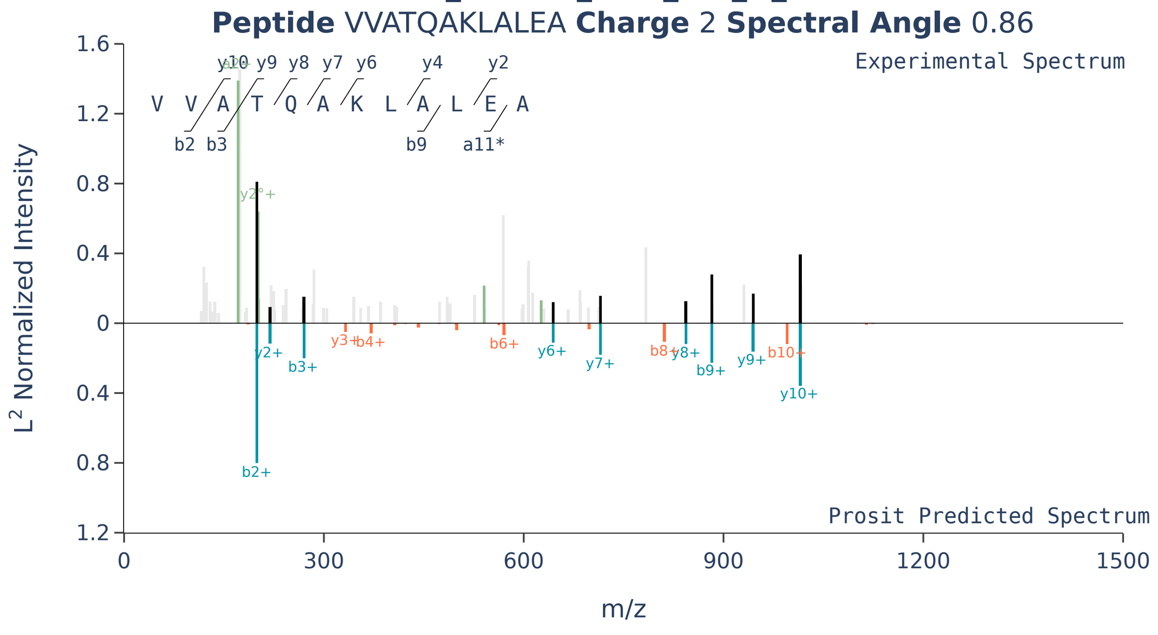

Source Fu_HeLa_inf_2_R1 **Scan** 3224
Peptide ARKGGGAGETVSLH **Charge** 3 **Spectral Angle** 0.85

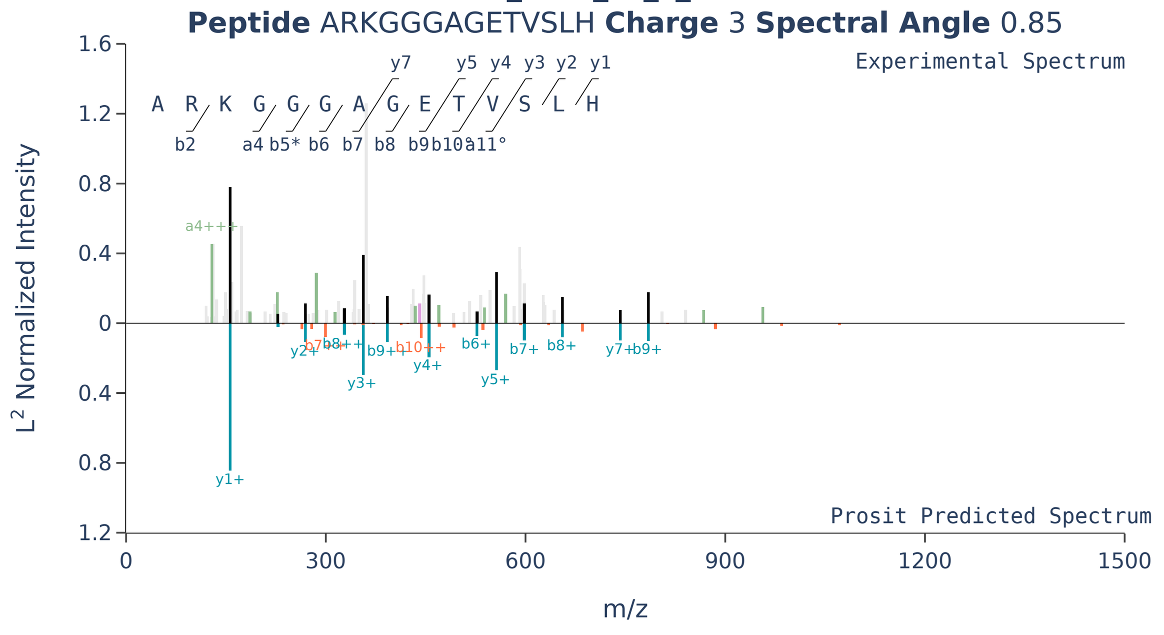

Source Fusion_Infected_HeLa_24h_PI_R2 **Scan** 4694
Peptide INVRVKNIK **Charge** 3 **Spectral Angle** 0.92

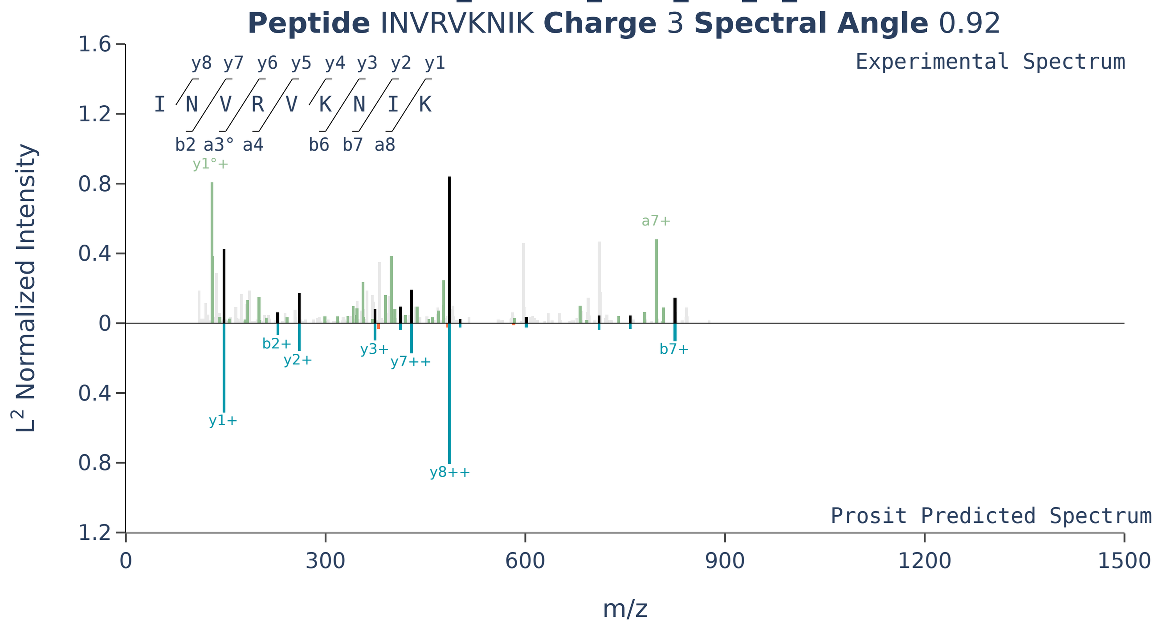

Source Fu_HeLa_inf_2_R1 **Scan** 8307
Peptide SSEGTRTTPSIVA **Charge** 2 **Spectral Angle** 0.79

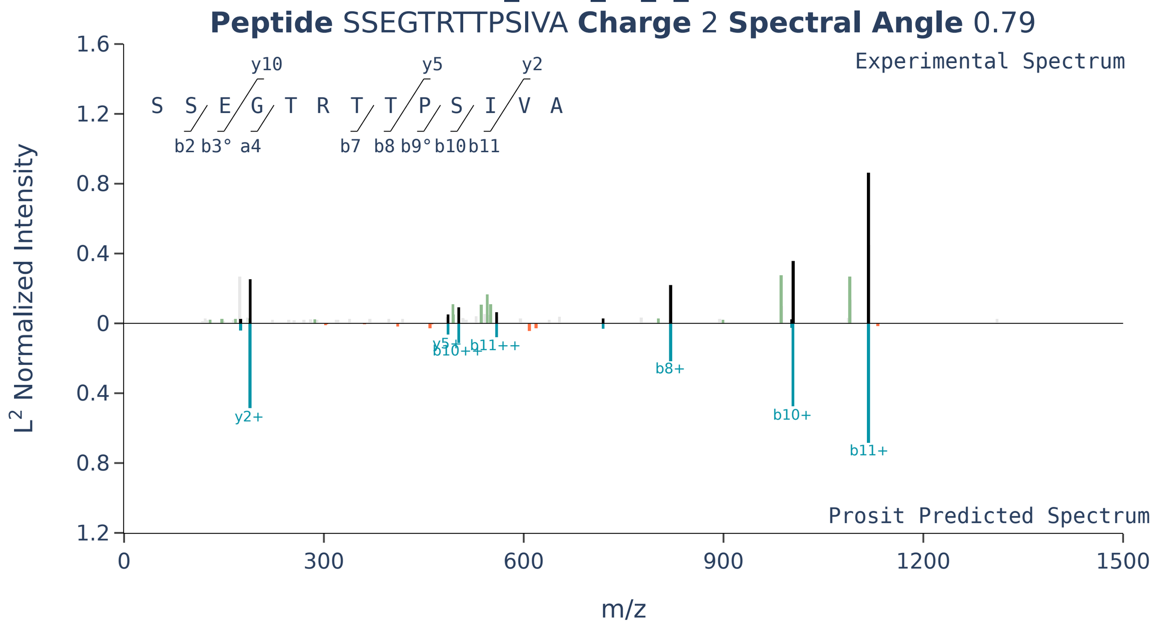

Source Fusion_Infected_HeLa_24h_PI_R1 **Scan** 3464
Peptide LTAGTDAATGTKD **Charge** 2 **Spectral Angle** 0.83

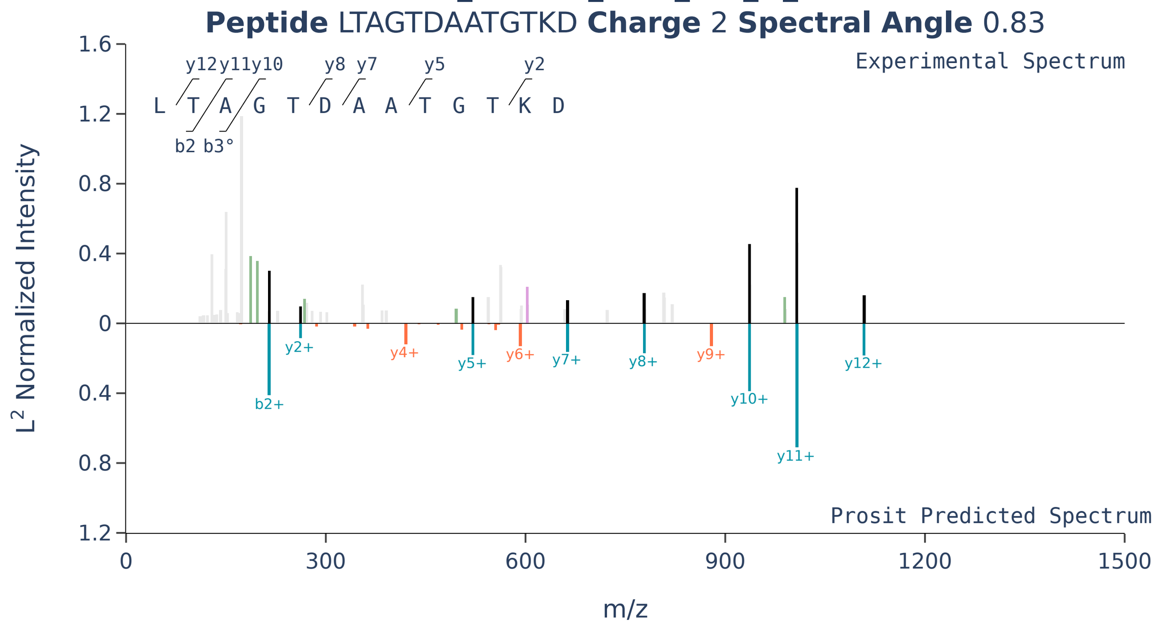

Source Fu_HeLa_inf_2_R2 **Scan** 5526
Peptide ITASPAAGA **Charge** 1 **Spectral Angle** 0.94

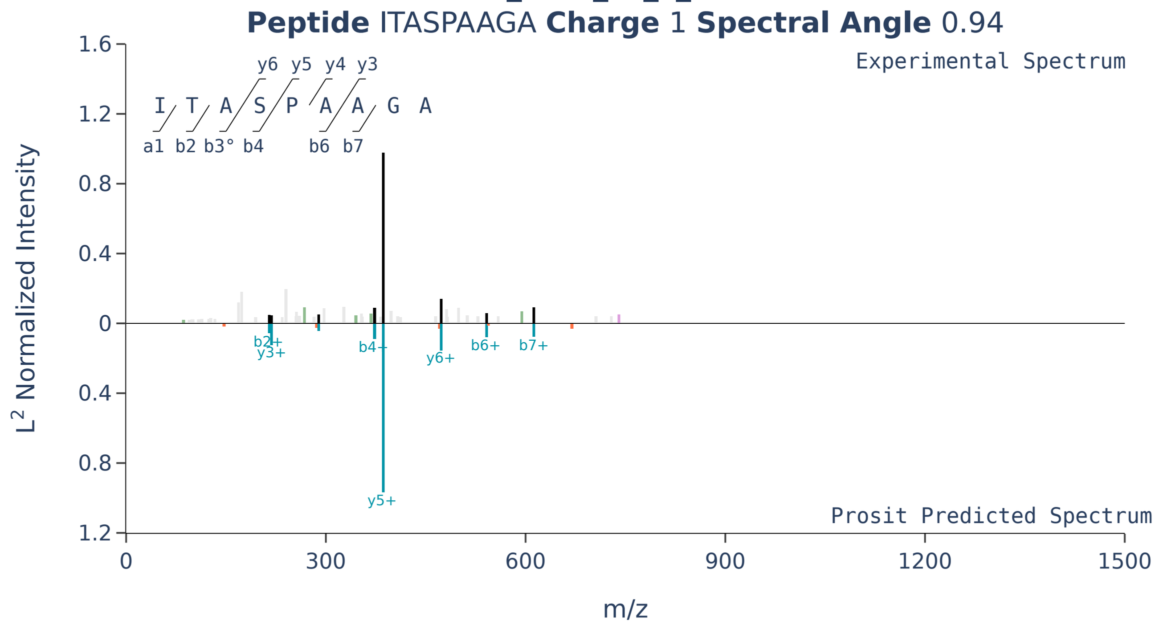

Source Fu_HeLa_inf_2_R2 **Scan** 8752
Peptide STANIVETA **Charge** 2 **Spectral Angle** 0.96

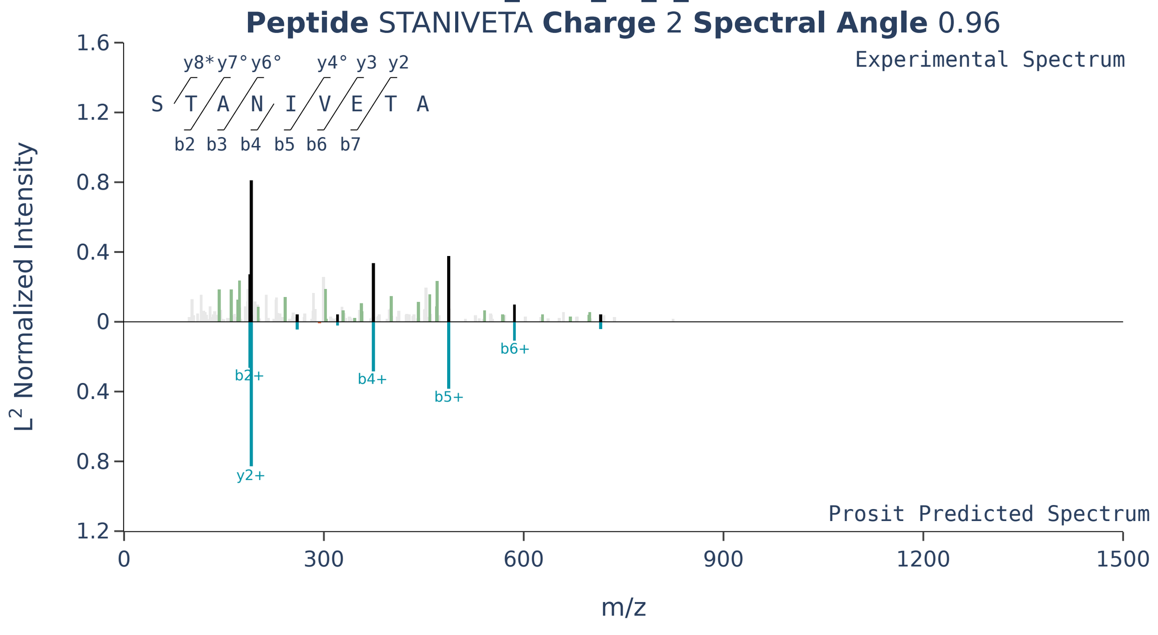

Source Fu_HeLa_inf_2_R1 **Scan** 10965
Peptide GSADKVILVTQ **Charge** 2 **Spectral Angle** 0.93

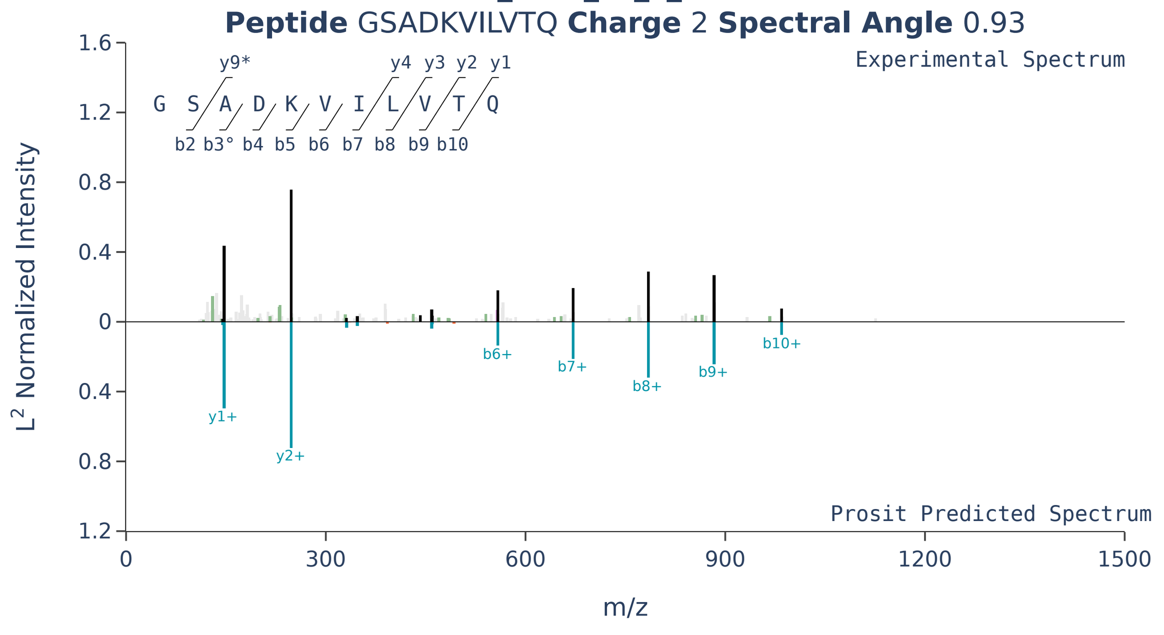

Source Fusion_Infected_HeLa_24h_PI_R2 **Scan** 10204
Peptide LTVIVGERGQ **Charge** 2 **Spectral Angle** 0.95

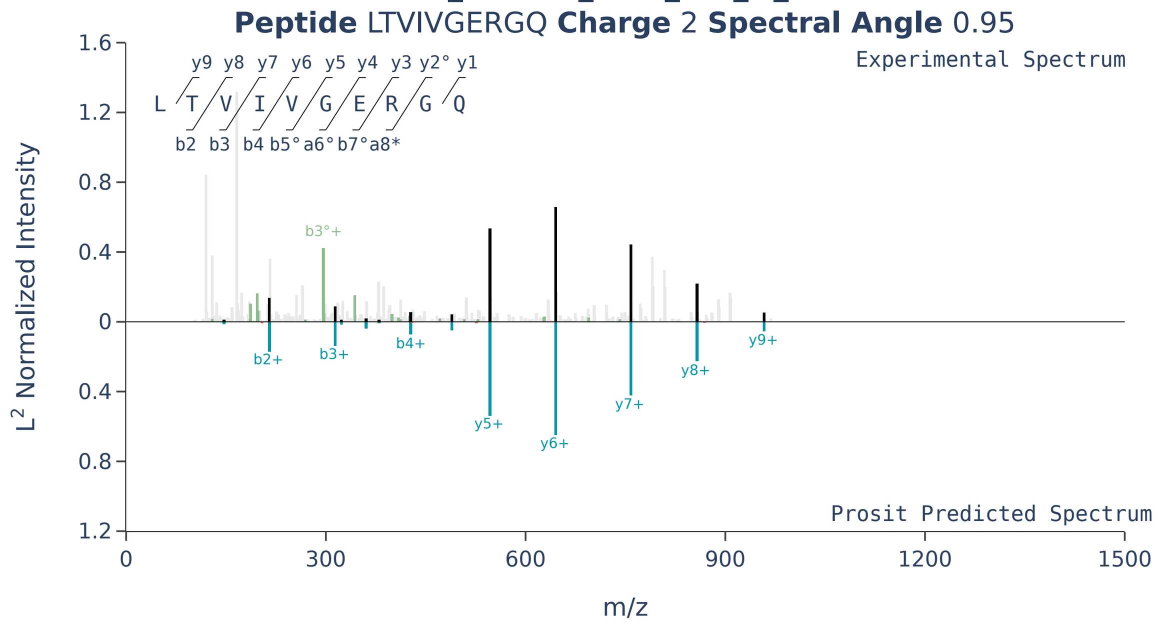

Source Fu_HeLa_inf_2_R1 **Scan** 11894
Peptide EVESLKAEL **Charge** 2 **Spectral Angle** 0.91

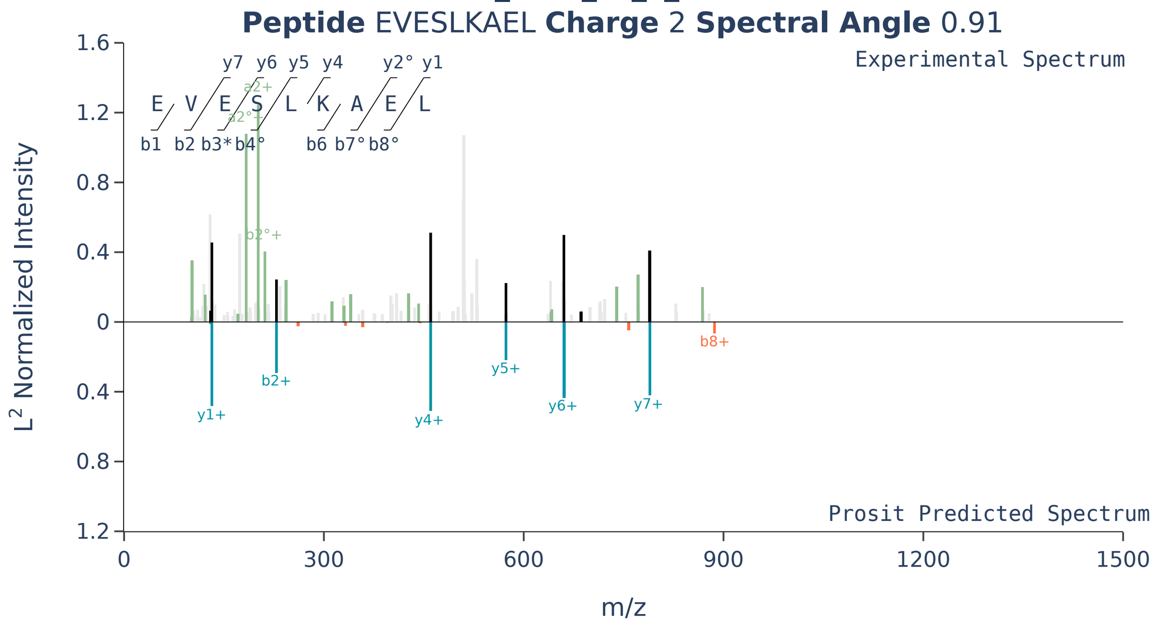

Source Fusion_Infected_HeLa_24h_PI_R1 **Scan** 9941
Peptide TNIHPTAIVED **Charge** 2 **Spectral Angle** 0.91

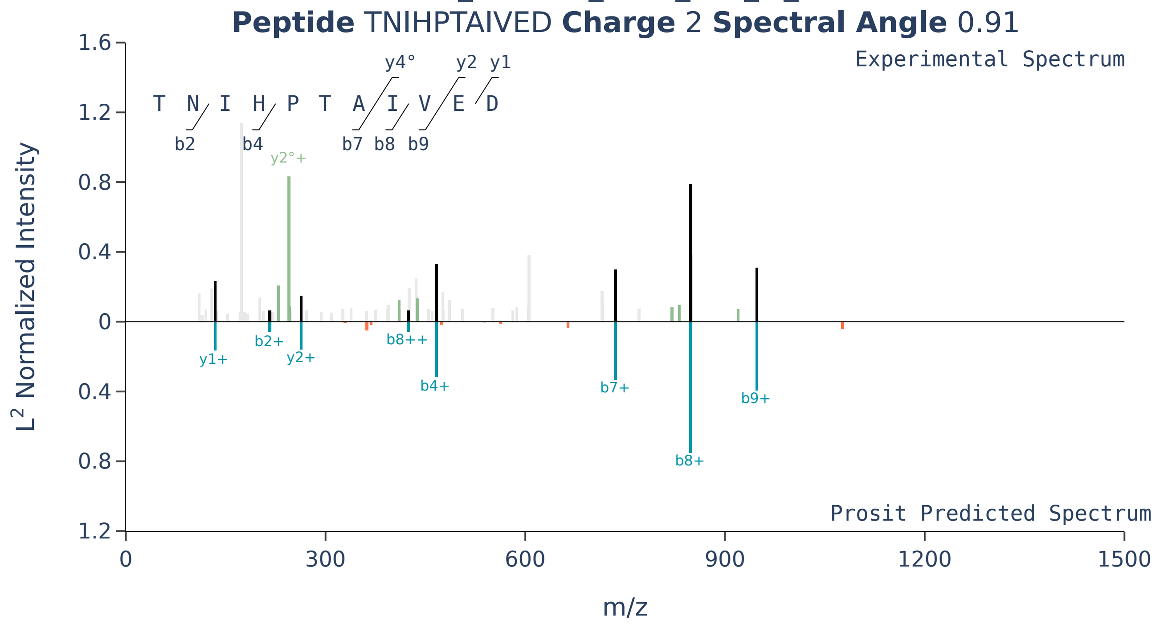

Source Fu_HeLa_inf_2_R1 **Scan** 9284
Peptide FAMGSTTRYSA **Charge** 2 **Spectral Angle** 0.94

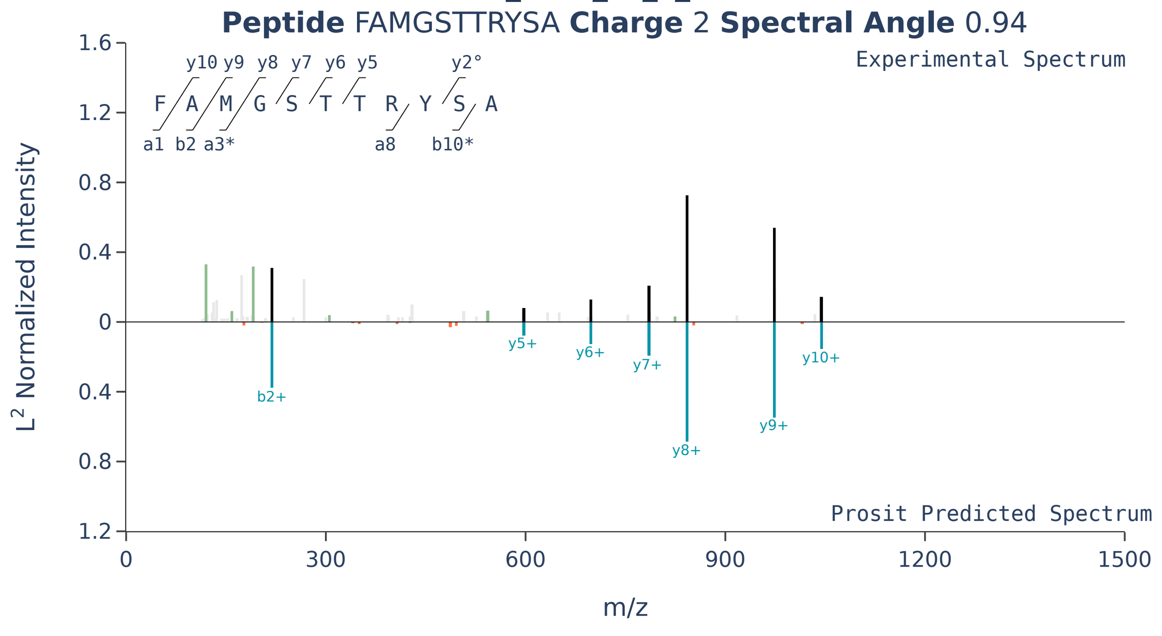

Source Fu_HeLa_inf_2_R2 **Scan** 11906
Peptide IAAPMSQVA **Charge** 1 **Spectral Angle** 0.88

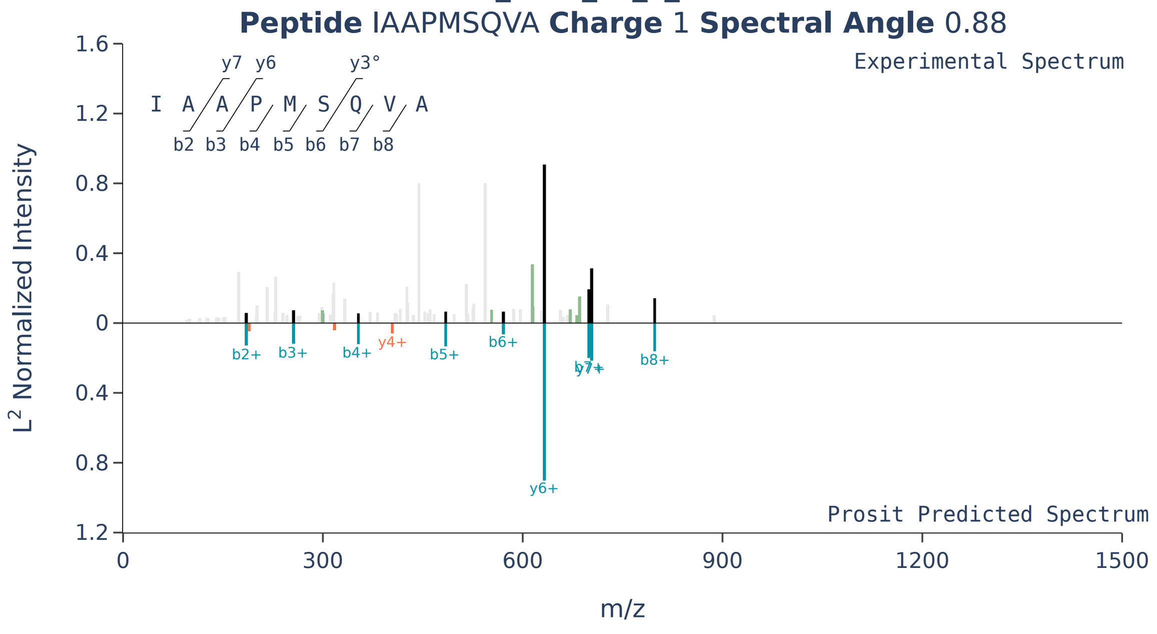

Source Fusion_Infected_HeLa_24h_PI_R2 **Scan** 3026
Peptide EVAKILRGKH **Charge** 3 **Spectral Angle** 0.82

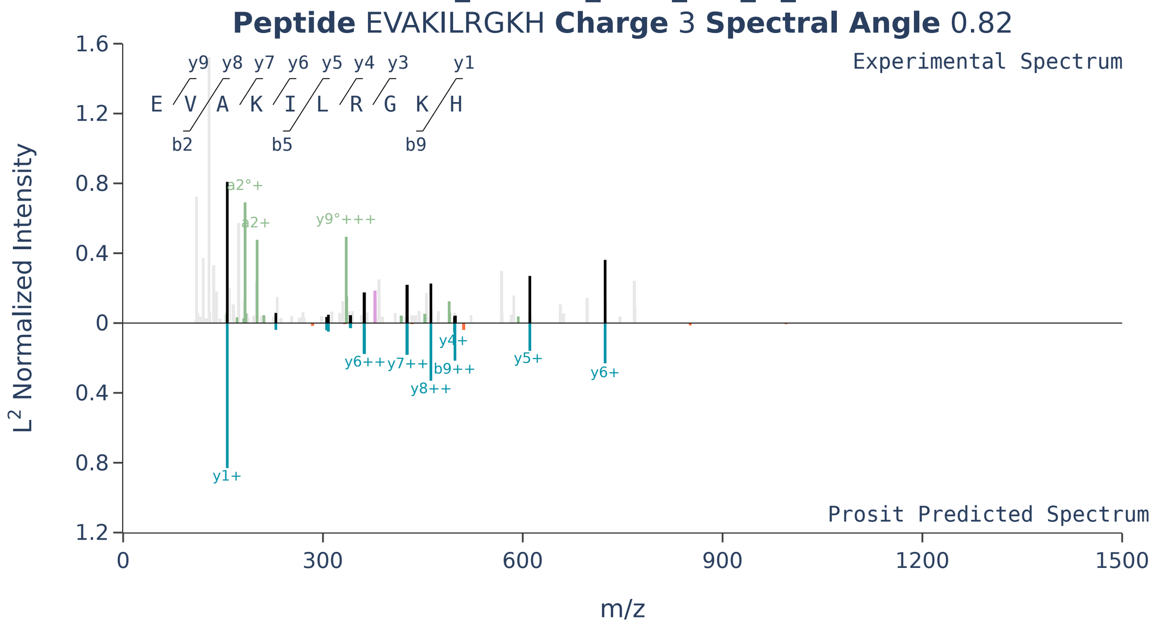

Source Fusion_Infected_HeLa_24h_PI_R2 **Scan** 10373
Peptide RPDNAIPTVATA **Charge** 2 **Spectral Angle** 0.85

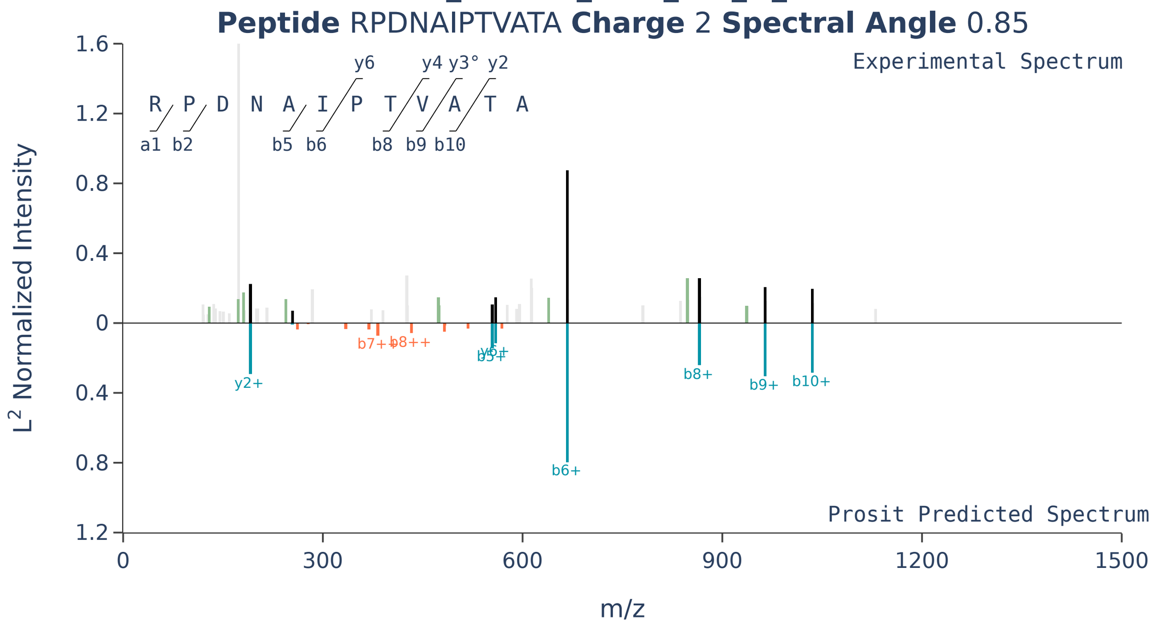

Source Fu_HeLa_inf_2_R1 **Scan** 5439
Peptide GSLVGRQAPD **Charge** 2 **Spectral Angle** 0.87

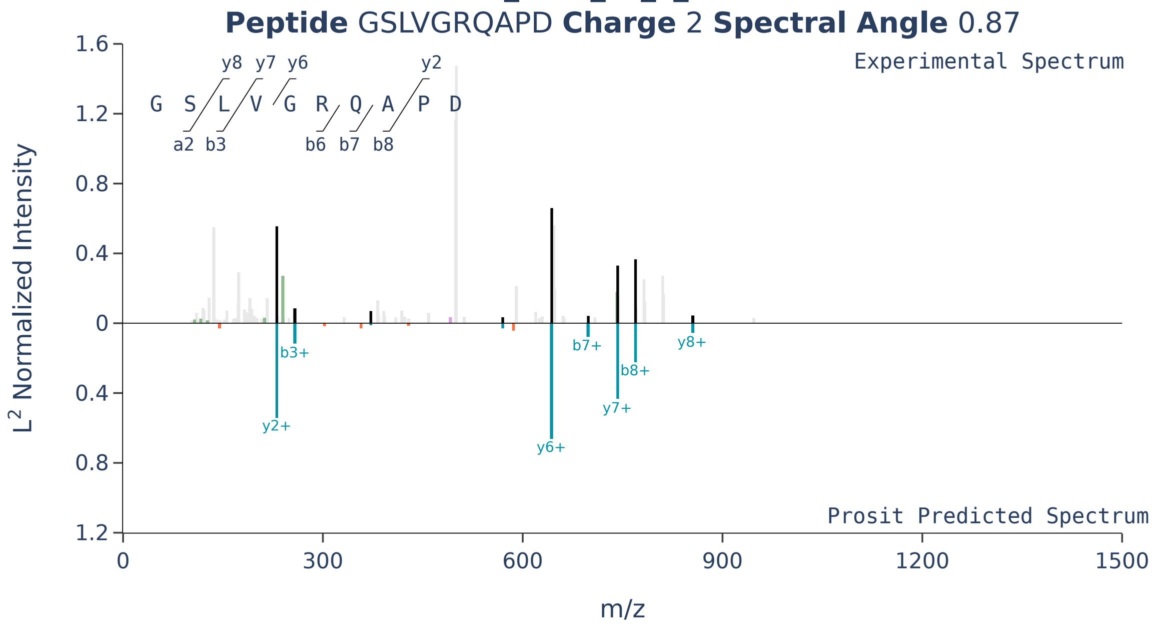

Source Fusion_Infected_HeLa_24h_PI_R1 **Scan** 13411
Peptide EAIYTEGLRNV **Charge** 2 **Spectral Angle** 0.91

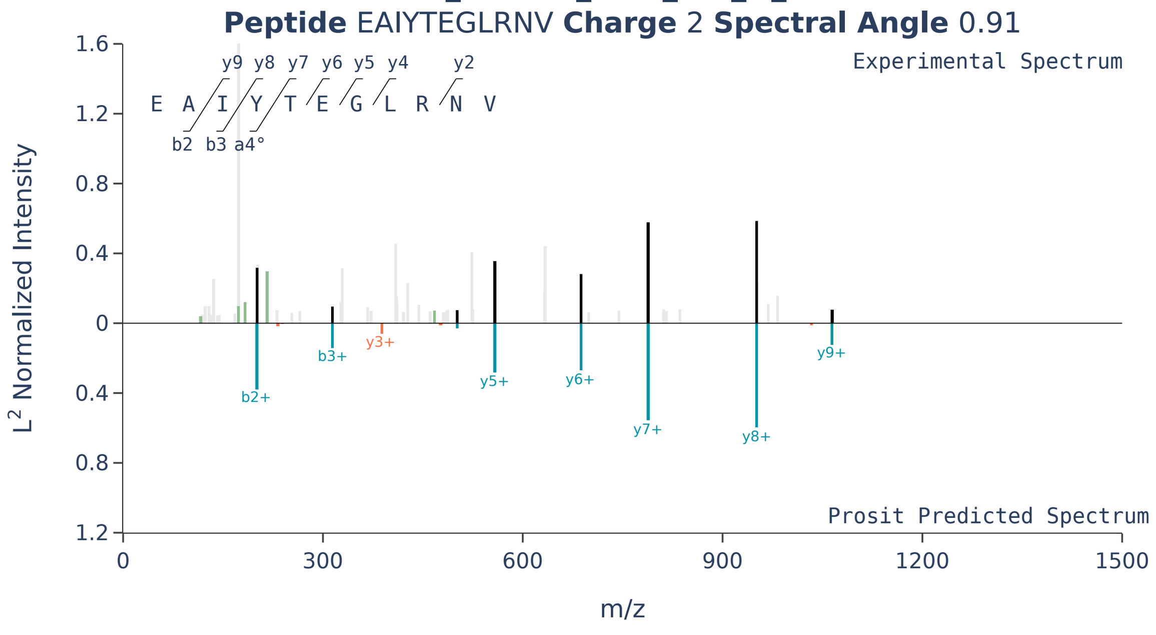

Source Fu_HeLa_inf_2_R1 **Scan** 4307
Peptide EVSNADHNL **Charge** 2 **Spectral Angle** 0.84

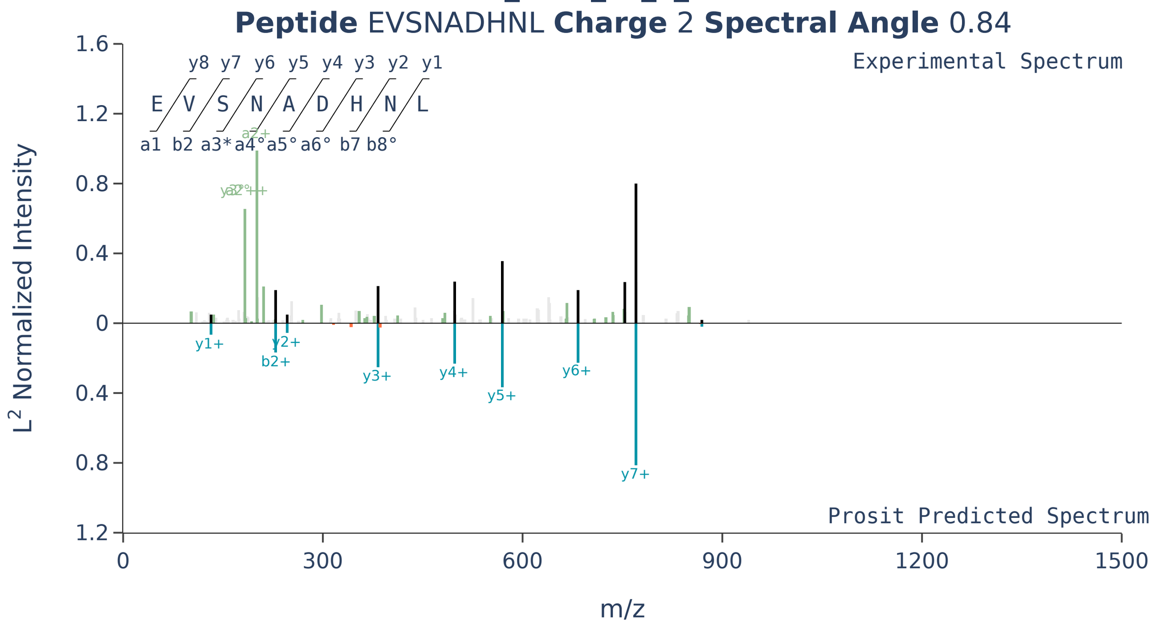

Source Fu_HeLa_inf_2_R1 **Scan** 5487
Peptide GANPMDLKRGIDKA **Charge** 3 **Spectral Angle** 0.85

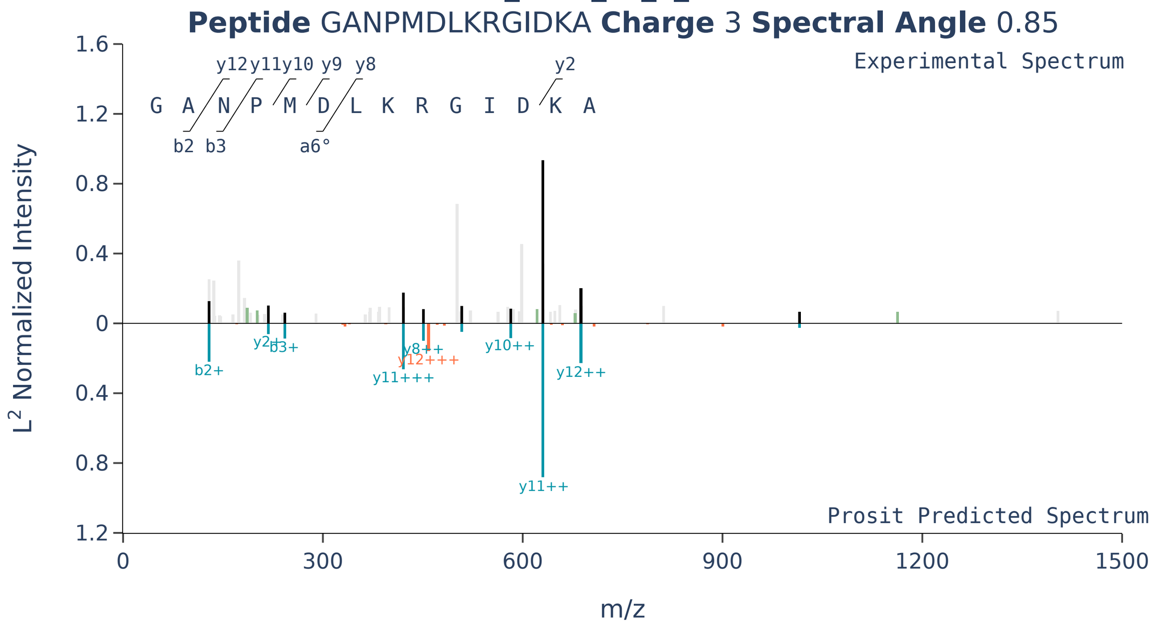

Source Fu_HeLa_inf_2_R2 **Scan** 11261
Peptide GVAVIRVGA **Charge** 2 **Spectral Angle** 0.98

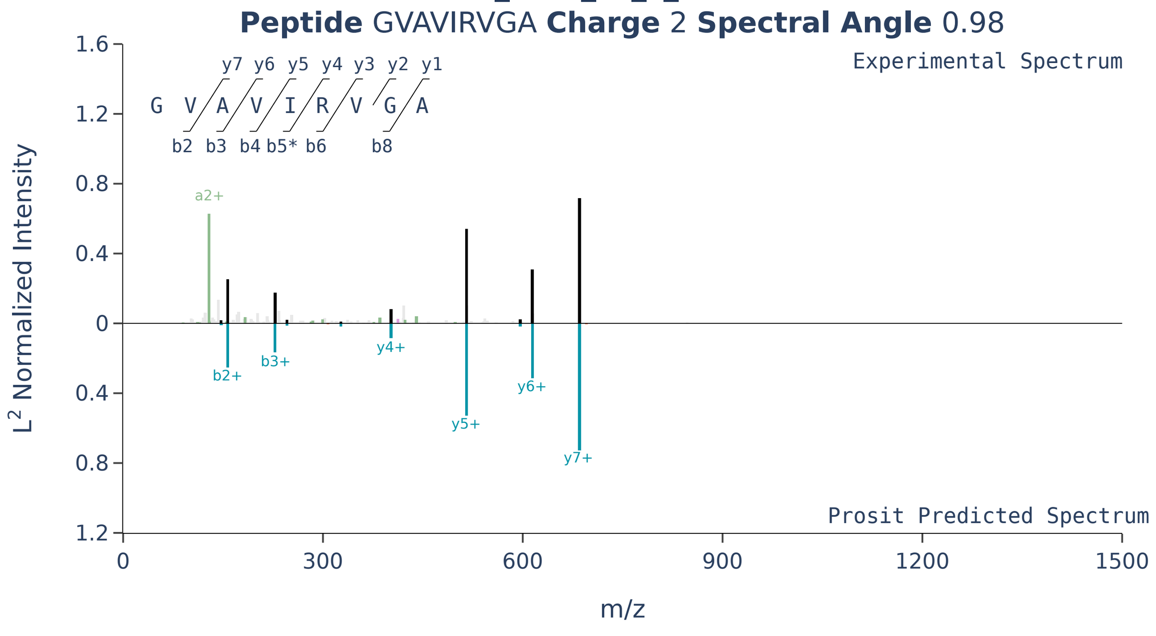

Source Fu_HeLa_inf_2_R2 **Scan** 2152
Peptide SRRHAAEKKVIPGD **Charge** 3 **Spectral Angle** 0.79

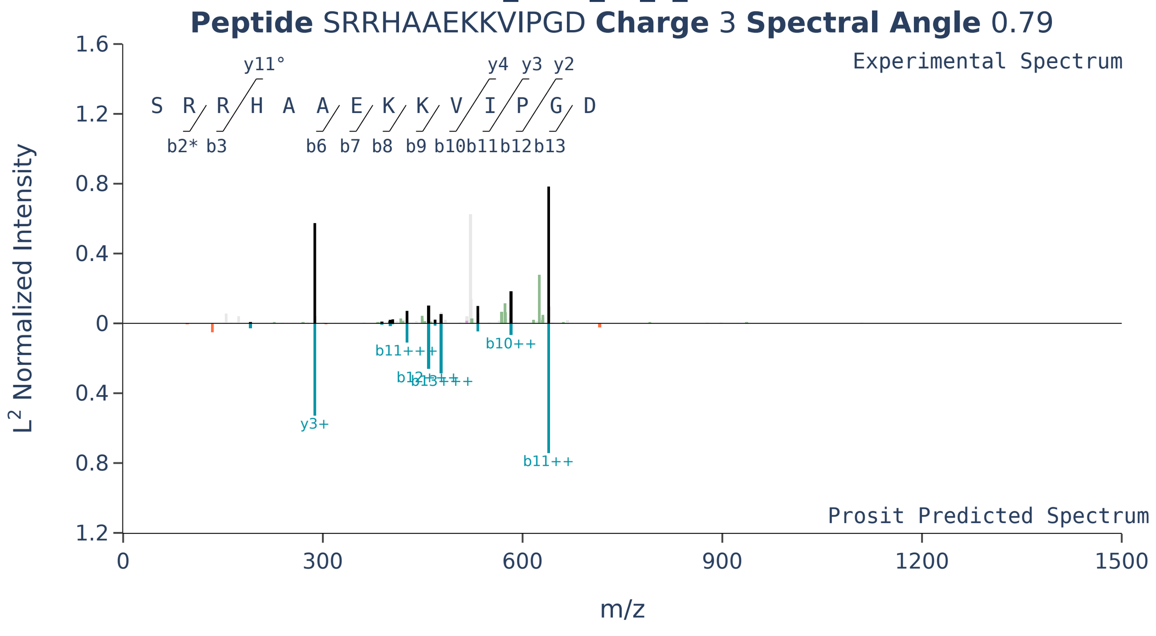

Source Fusion_Infected_HeLa_24h_PI_R2 **Scan** 5629
Peptide SIKADPSKGDKLV **Charge** 3 **Spectral Angle** 0.63

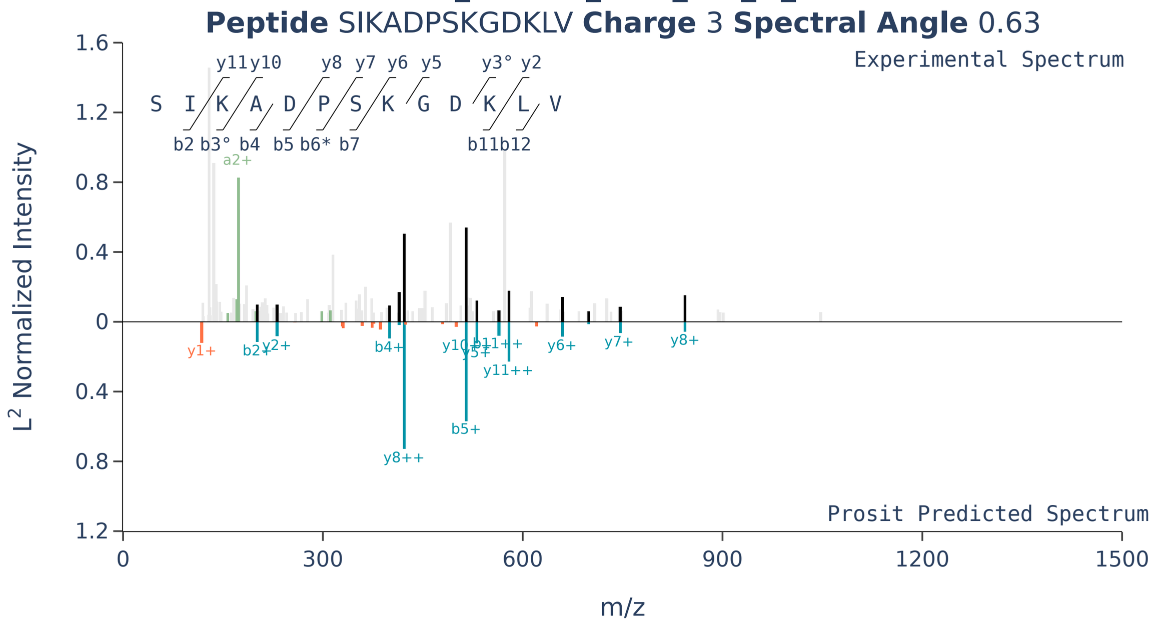

Source Fu_HeLa_inf_2_R1 **Scan** 8142
Peptide HLTVIVGERGQ **Charge** 2 **Spectral Angle** 0.88

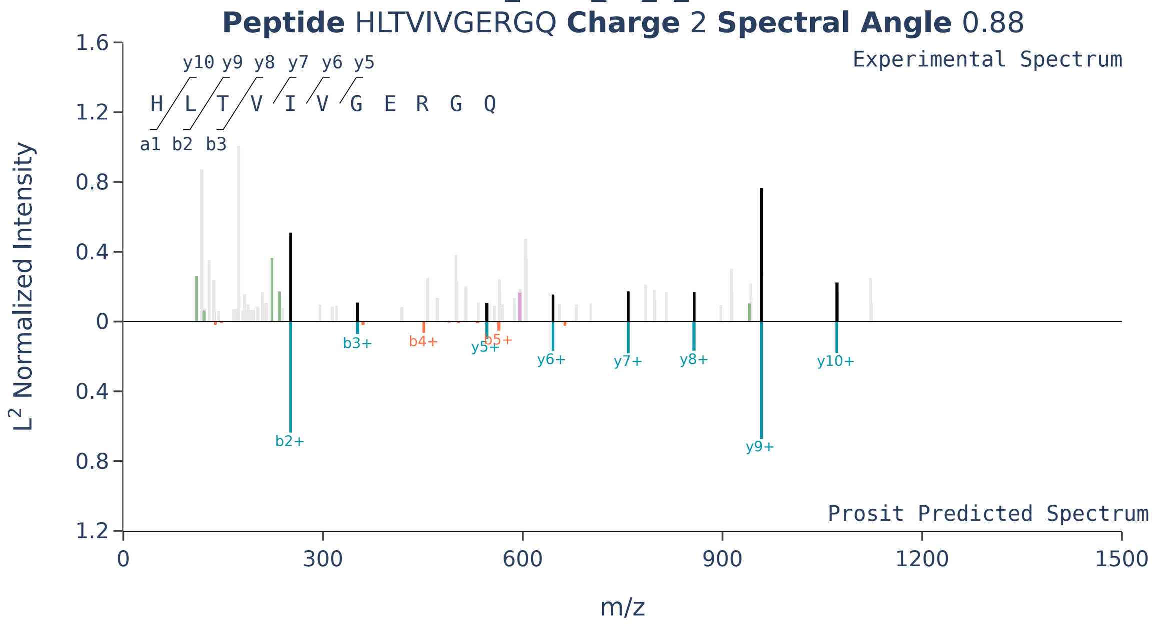

Source Fu_HeLa_inf_2_R1 **Scan** 12932
Peptide FAYGDPVSA **Charge** 1 **Spectral Angle** 0.88

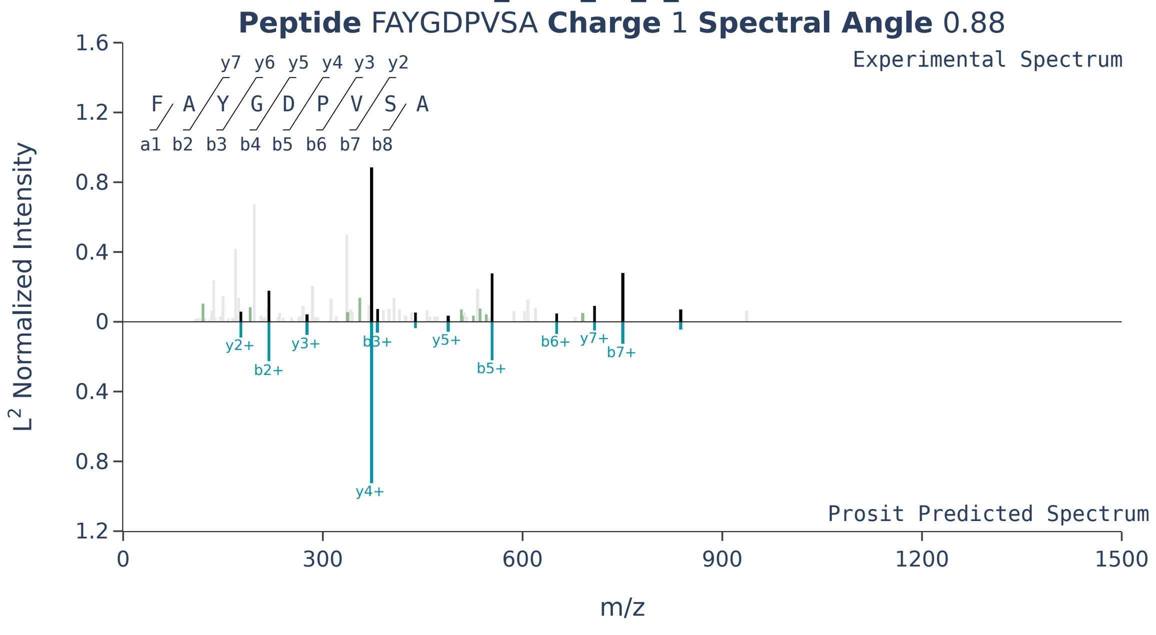

Source Fusion_Infected_HeLa_24h_PI_R1 **Scan** 5379
Peptide PTINQLIRKKRQS **Charge** 4 **Spectral Angle** 0.81

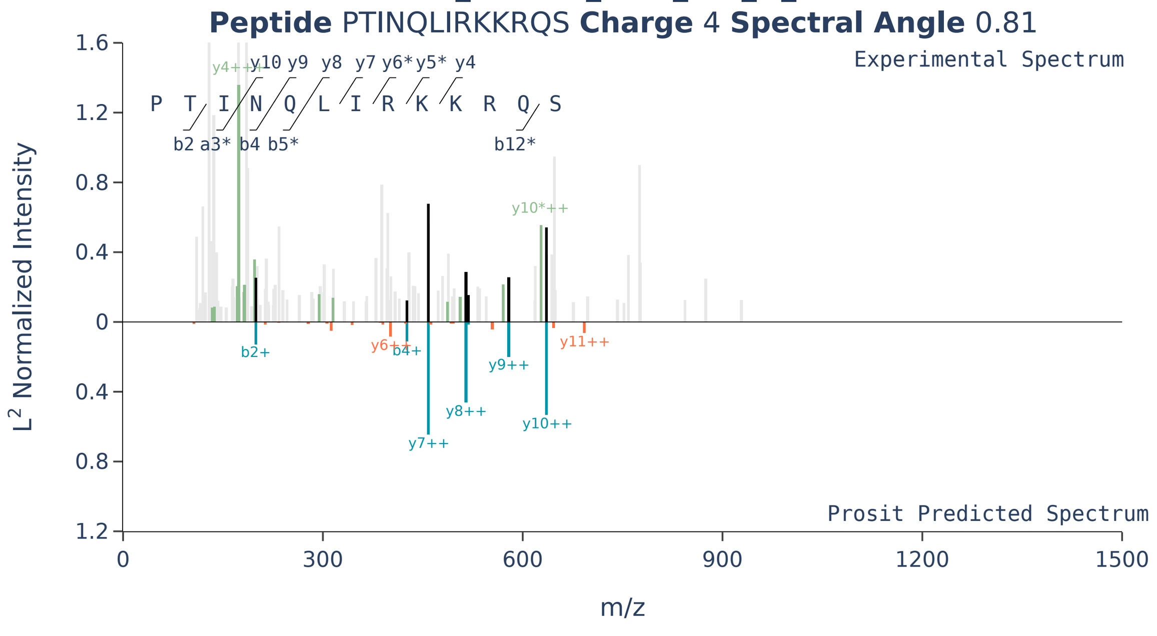

Source Fusion_Infected_HeLa_24h_PI_R1 **Scan** 5355
Peptide GAPARPYQEIHRQVA **Charge** 3 **Spectral Angle** 0.78

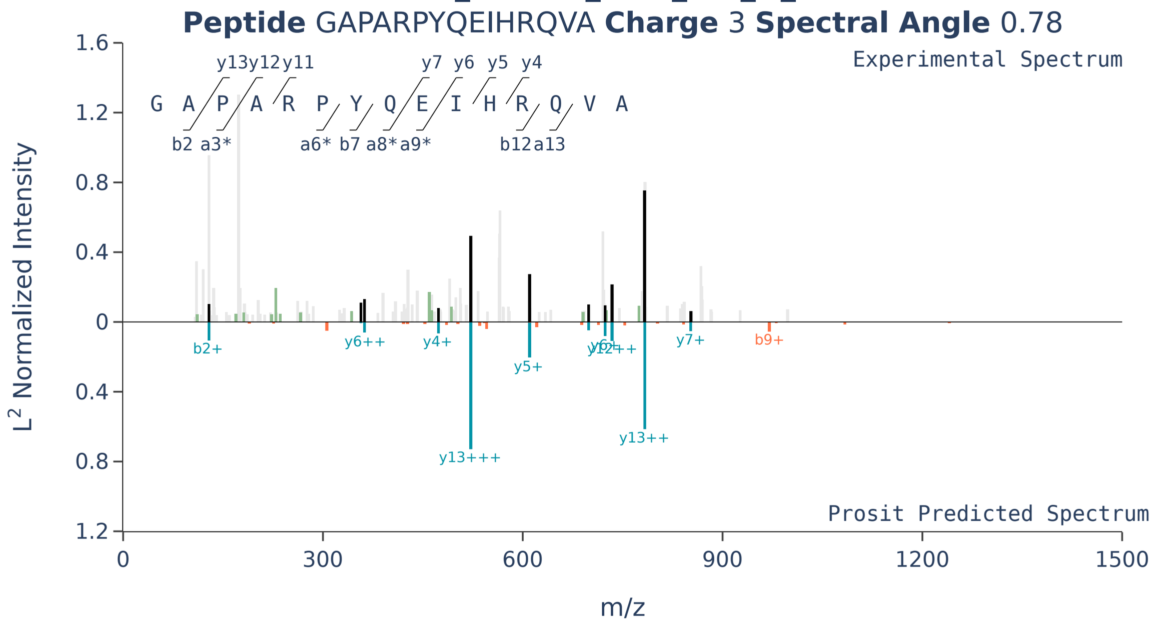

Source Fusion_Infected_HeLa_24h_PI_R1 **Scan** 2336
Peptide LQVKTKTPKKPNSA **Charge** 3 **Spectral Angle** 0.77

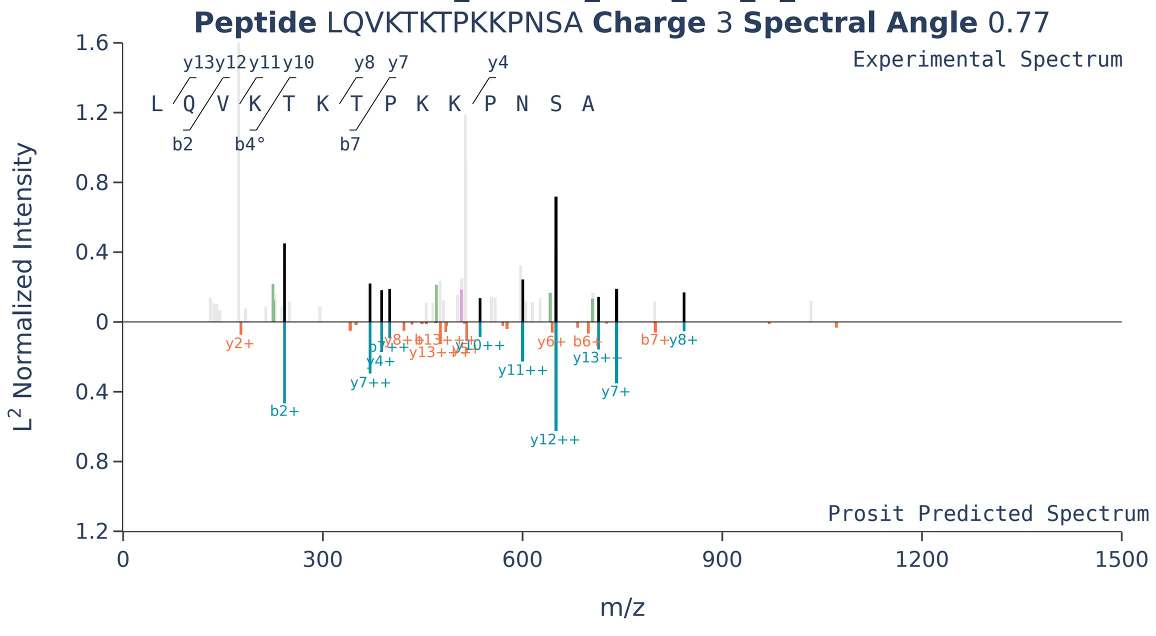

Source Fusion_Infected_HeLa_24h_PI_R2 **Scan** 5332
Peptide PTINQLIRKKRQSG **Charge** 3 **Spectral Angle** 0.93

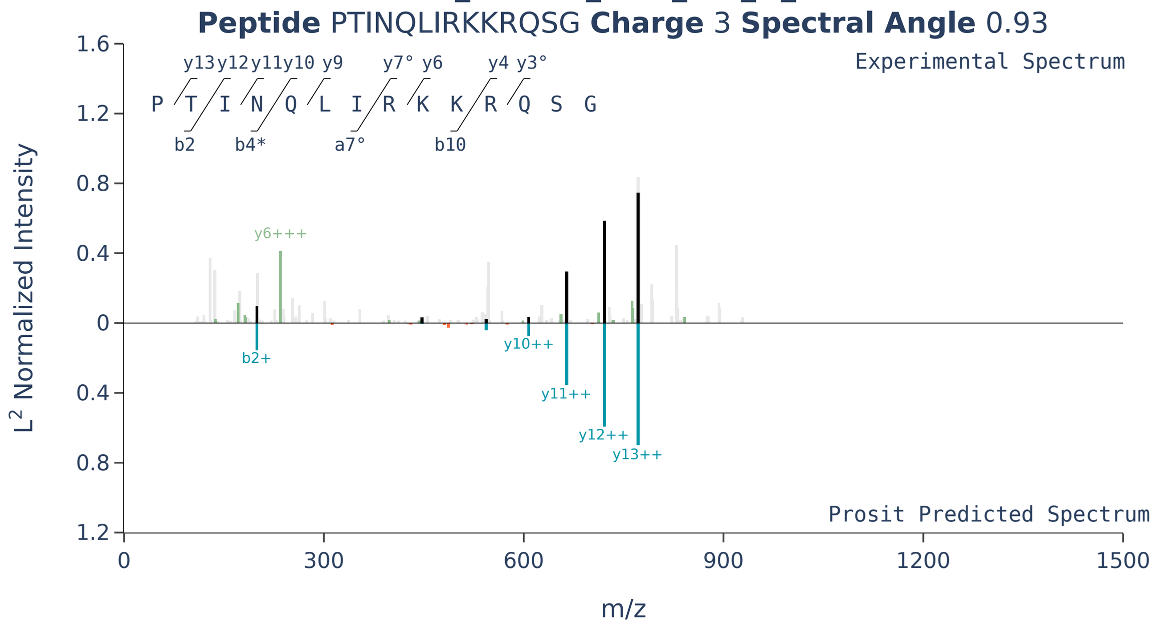

Source Fusion_Infected_HeLa_24h_PI_R1 **Scan** 7723
Peptide LATHIVANA **Charge** 2 **Spectral Angle** 0.85

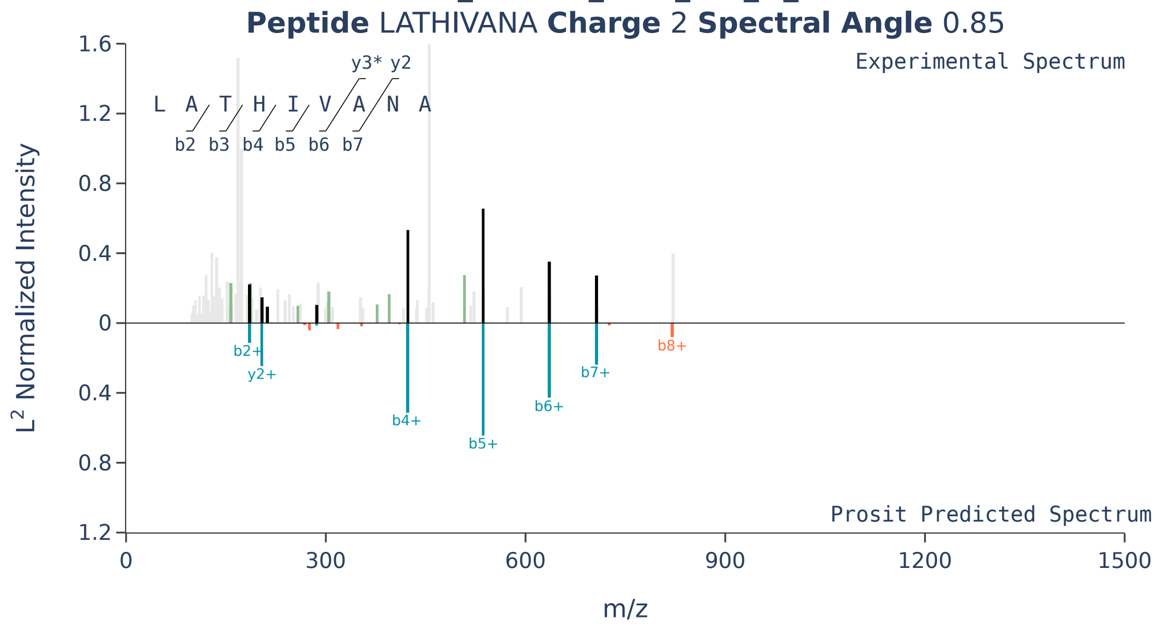

Source Fu_HeLa_inf_2_R2 **Scan** 11989
Peptide EMIGVGFRA **Charge** 2 **Spectral Angle** 0.96

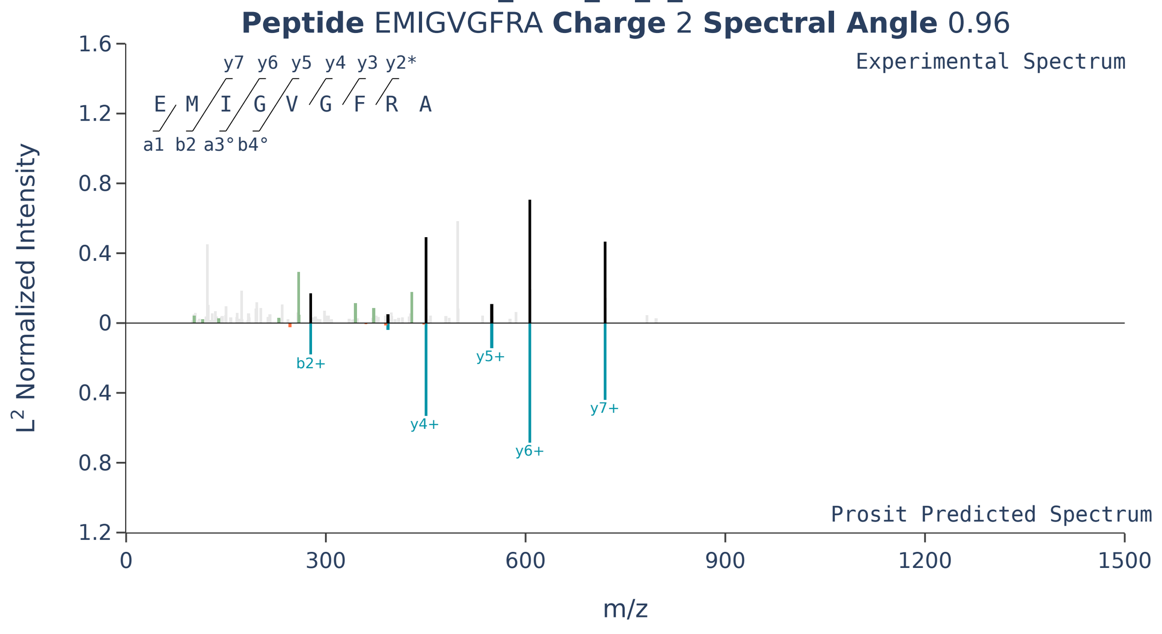

Source Fu_HeLa_inf_2_R2 **Scan** 1761
Peptide TAKKAPVRKAVA **Charge** 2 **Spectral Angle** 0.8

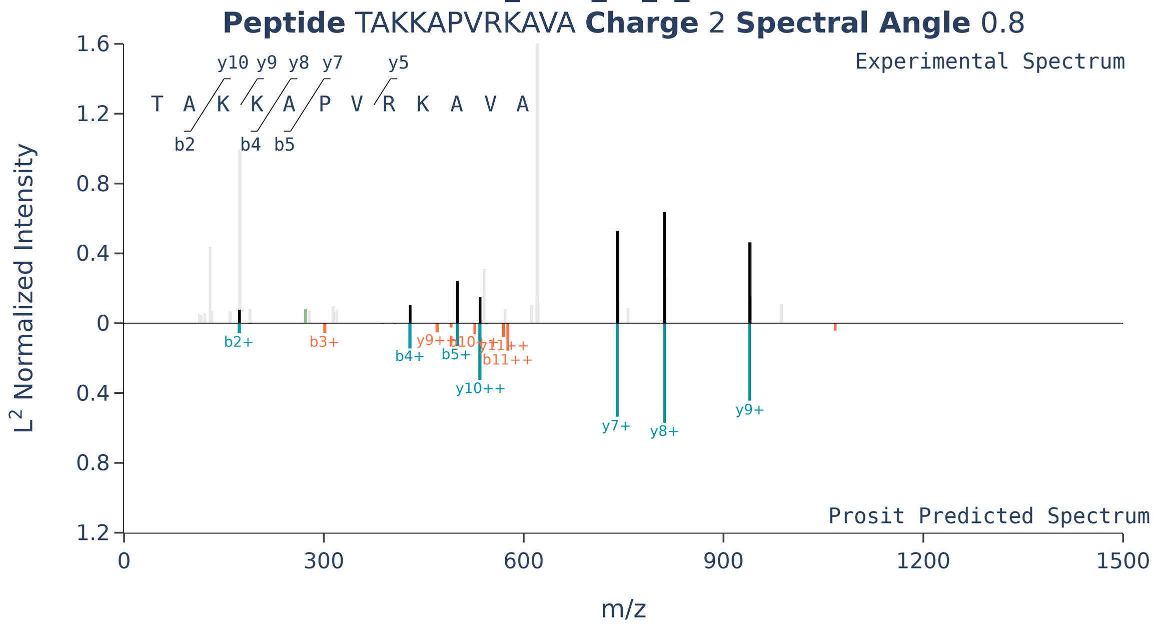

Source Fusion_Infected_HeLa_24h_PI_R1 **Scan** 5826
Peptide IVVMKKTDRTYVS **Charge** 3 **Spectral Angle** 0.28

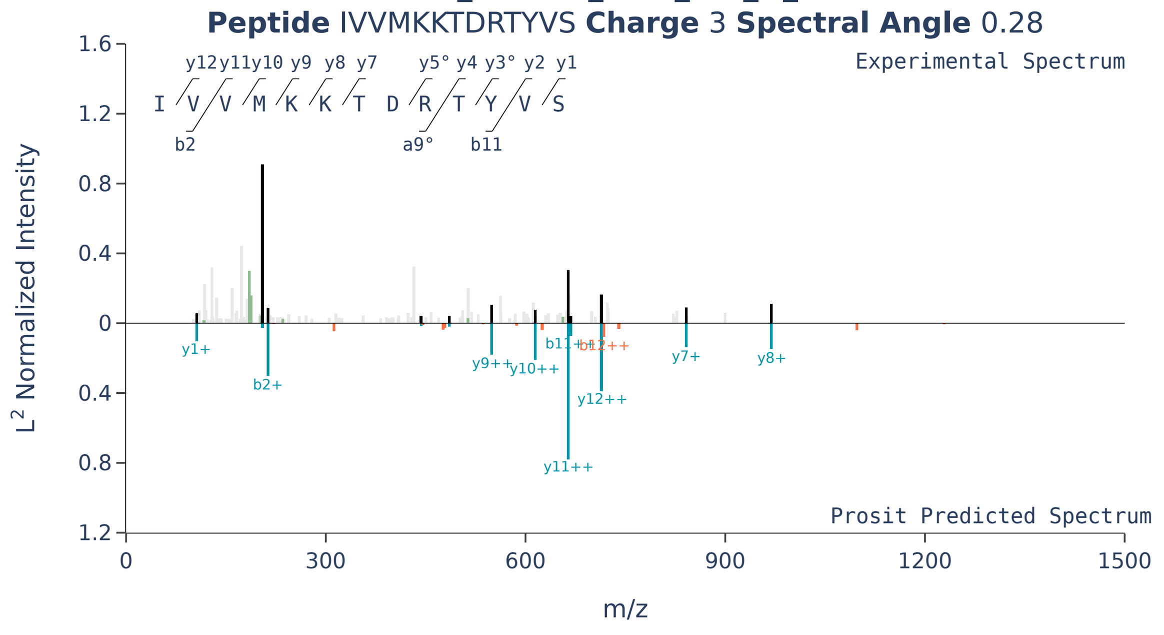

Source Fusion_Infected_HeLa_24h_PI_R1 **Scan** 7406
Peptide SQLSLIGKKEG **Charge** 3 **Spectral Angle** 0.81

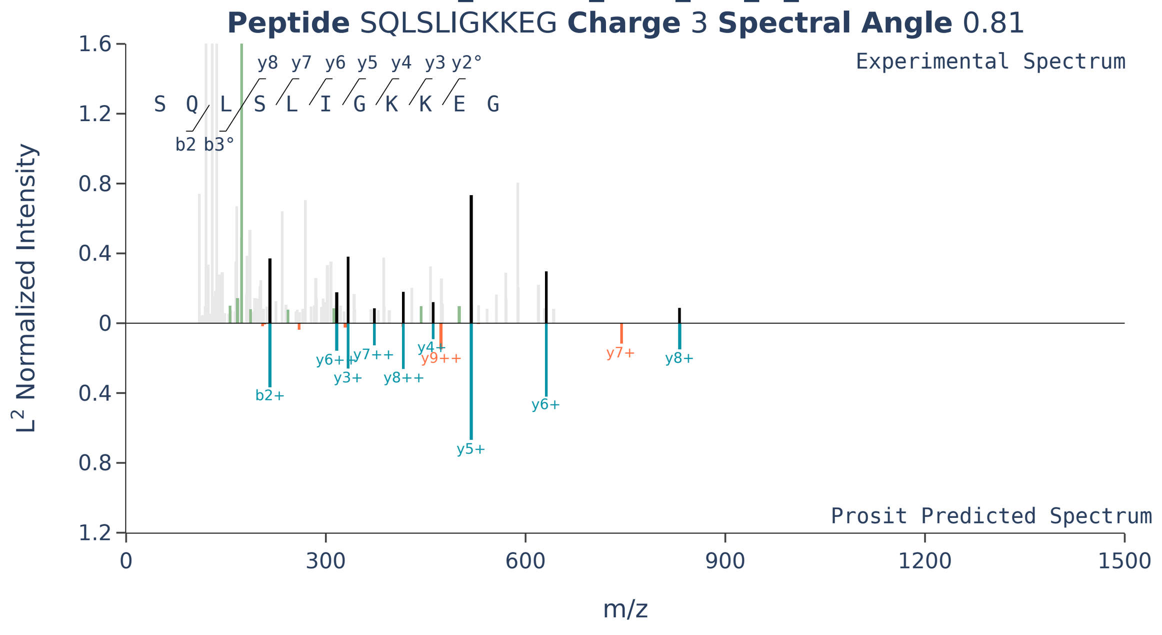

Source Fusion_Infected_HeLa_24h_PI_R2 **Scan** 6204
Peptide LVGIPAKRQA **Charge** 3 **Spectral Angle** 0.87

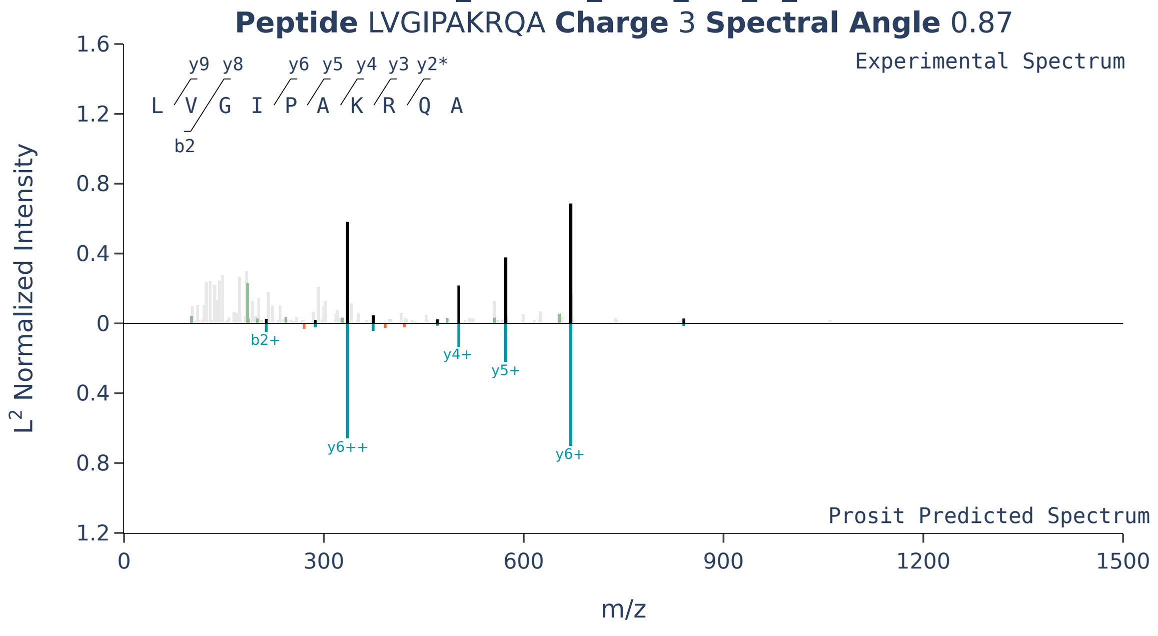

Source Fu_HeLa_inf_2_R1 **Scan** 16485
Peptide SVVGAGLAISA **Charge** 1 **Spectral Angle** 0.75

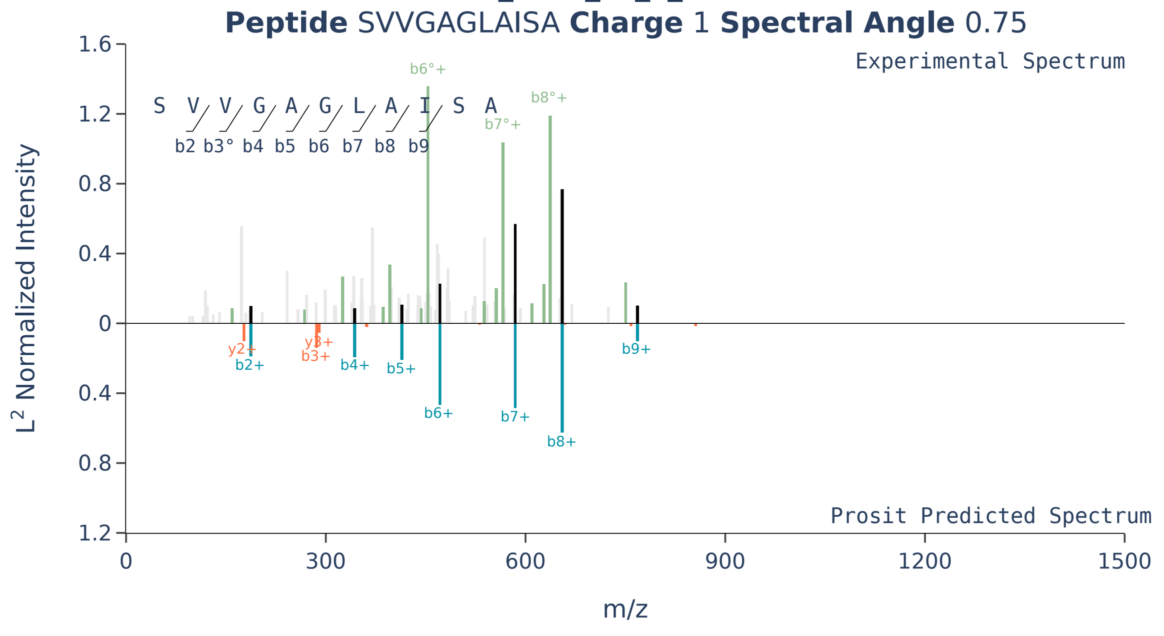

Source Fu_HeLa_inf_2_R1 **Scan** 6751
Peptide SVSLPHGTG **Charge** 2 **Spectral Angle** 0.93

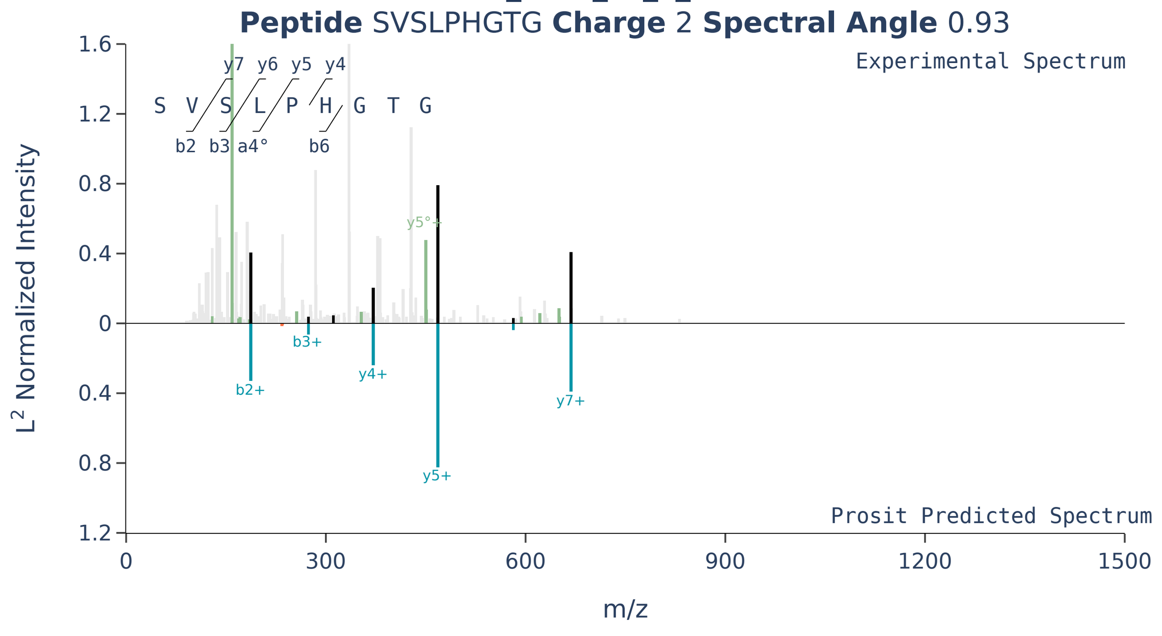

Source Fu_HeLa_inf_2_R2 **Scan** 20642
Peptide SVIIVGPEL **Charge** 1 **Spectral Angle** 0.86

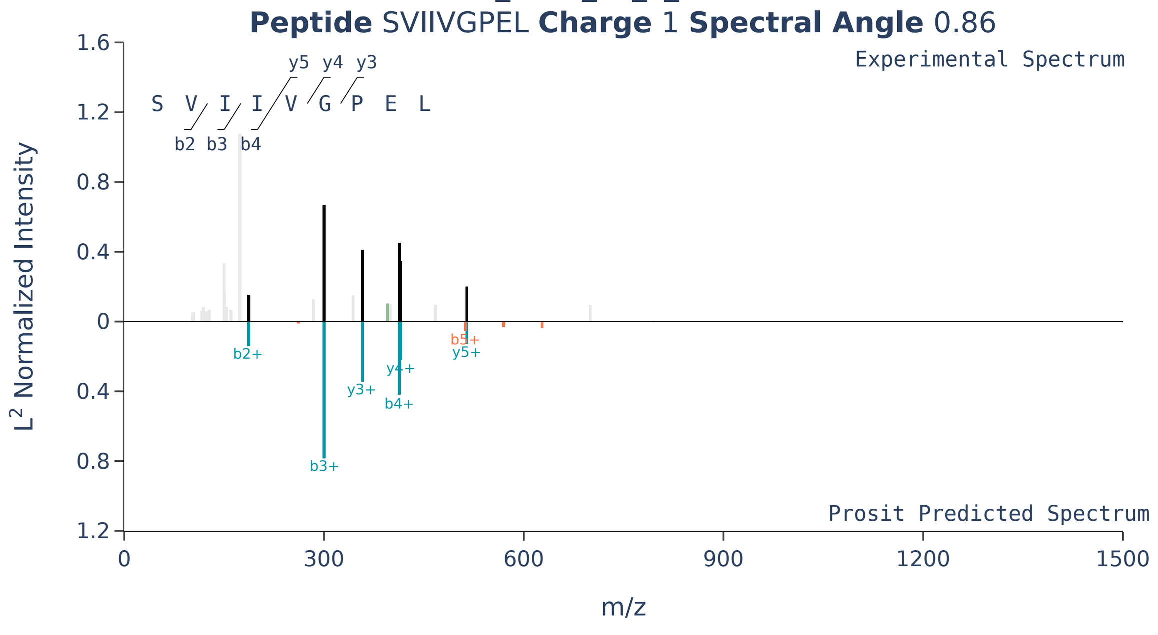

Source Fu_HeLa_inf_2_R2 **Scan** 15818
Peptide SVISVDANVVA **Charge** 2 **Spectral Angle** 0.93

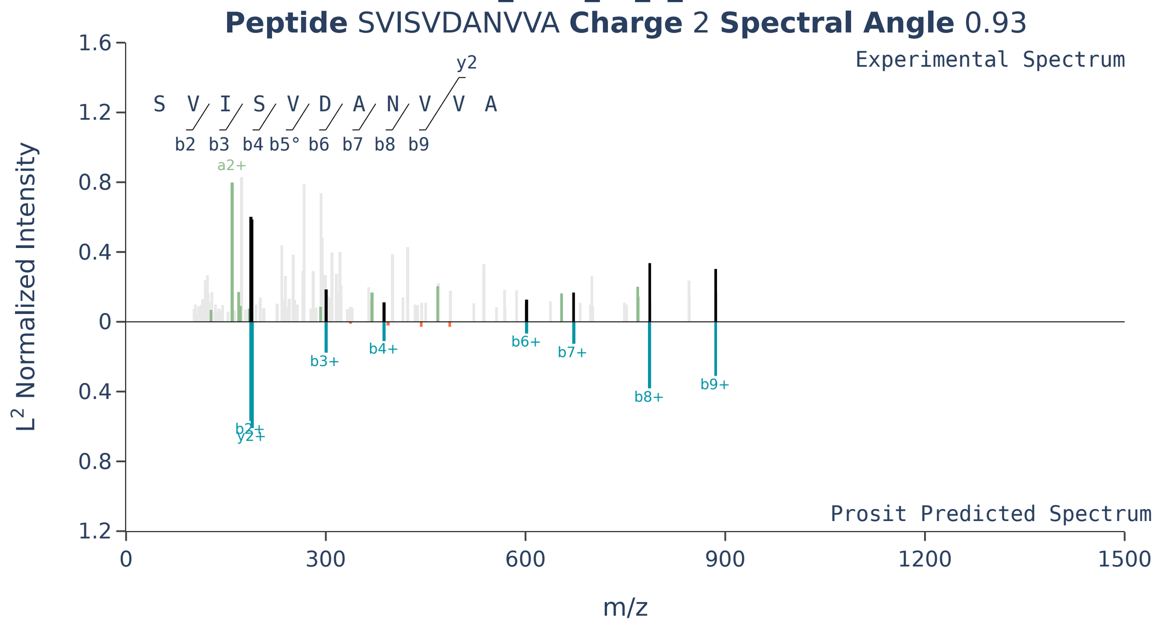

Source Fusion_Infected_HeLa_24h_PI_R1 **Scan** 3387
Peptide KRLIETPTKSS **Charge** 3 **Spectral Angle** 0.46

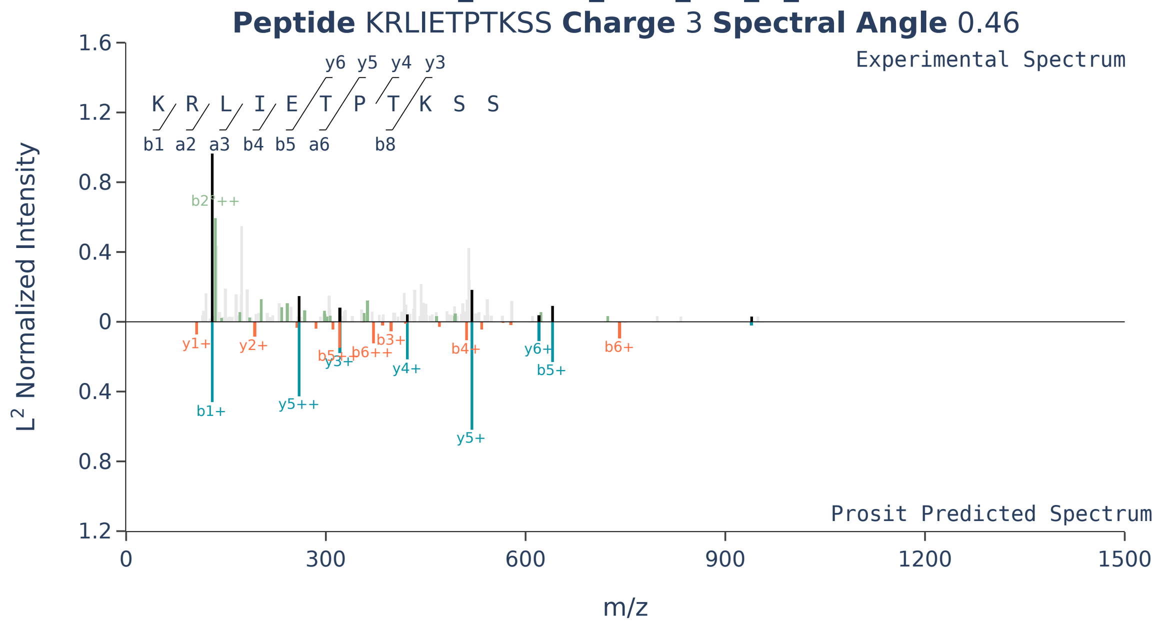

Source Fu_HeLa_inf_2_R2 **Scan** 2119
Peptide SKETFQRNKP **Charge** 3 **Spectral Angle** 0.85

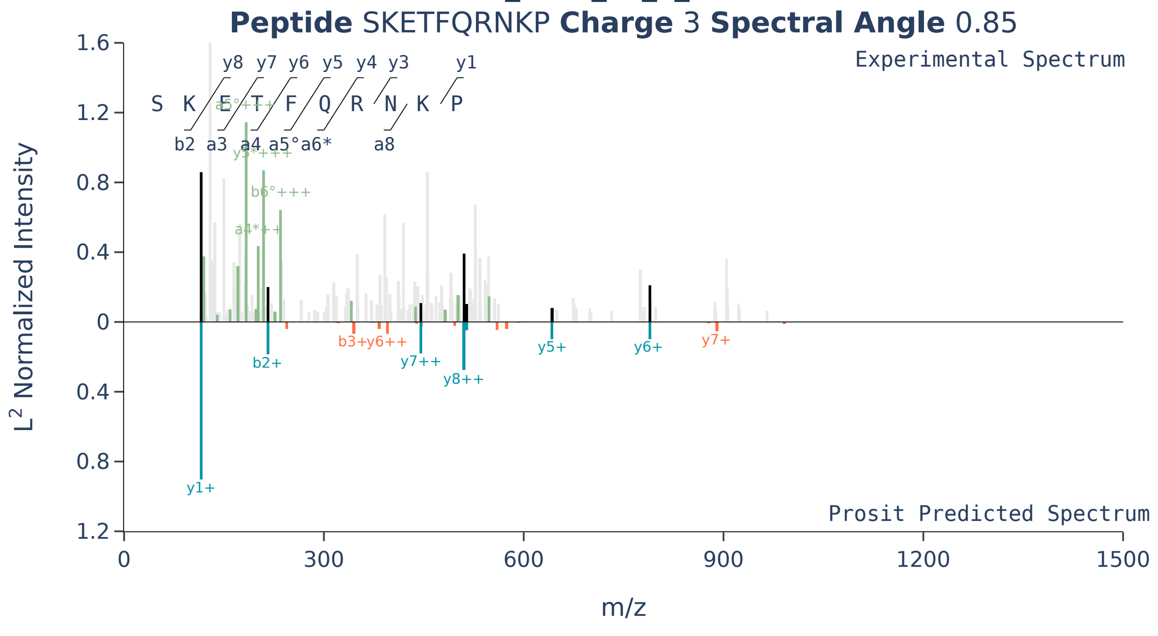

Source Fusion_Infected_HeLa_24h_PI_R1 **Scan** 5068
Peptide TVIVGERGQ **Charge** 2 **Spectral Angle** 0.95

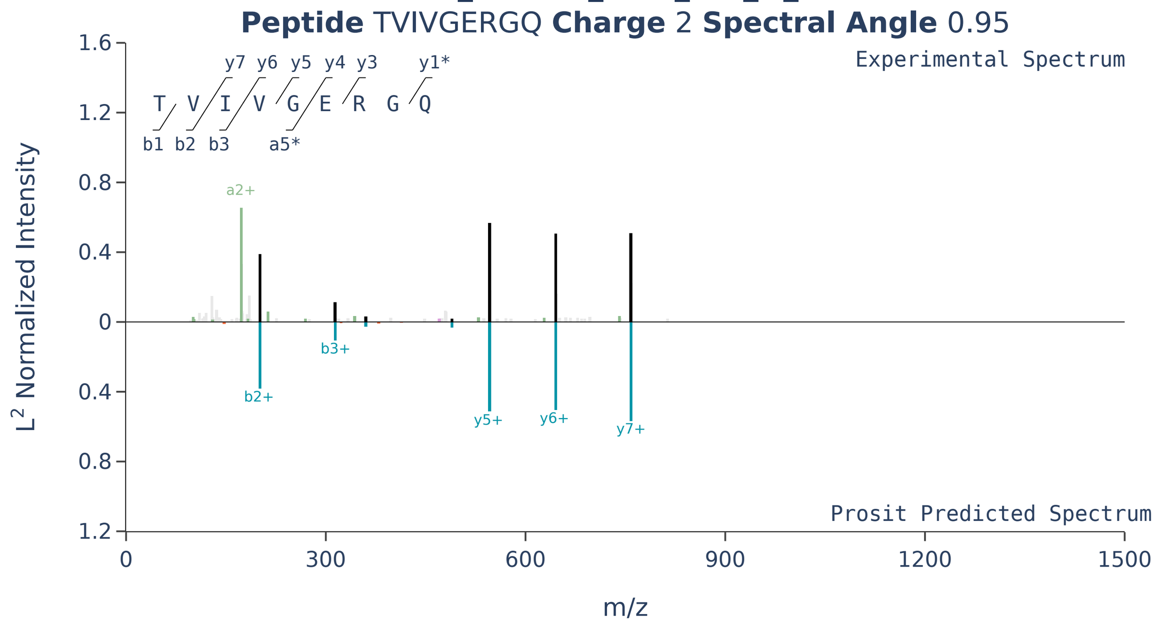

Source Fu_HeLa_inf_2_R1 **Scan** 15801
Peptide FSSPFSREL **Charge** 2 **Spectral Angle** 0.92

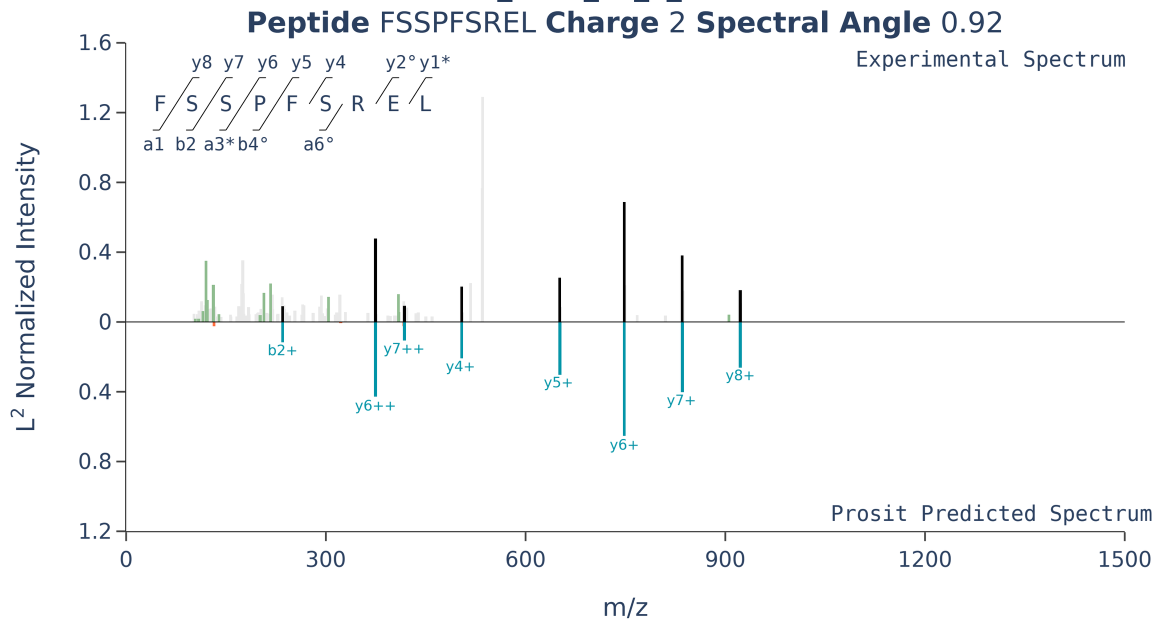

Source Fu_HeLa_inf_2_R2 **Scan** 6958
Peptide VEIVDKPE **Charge** 2 **Spectral Angle** 0.9

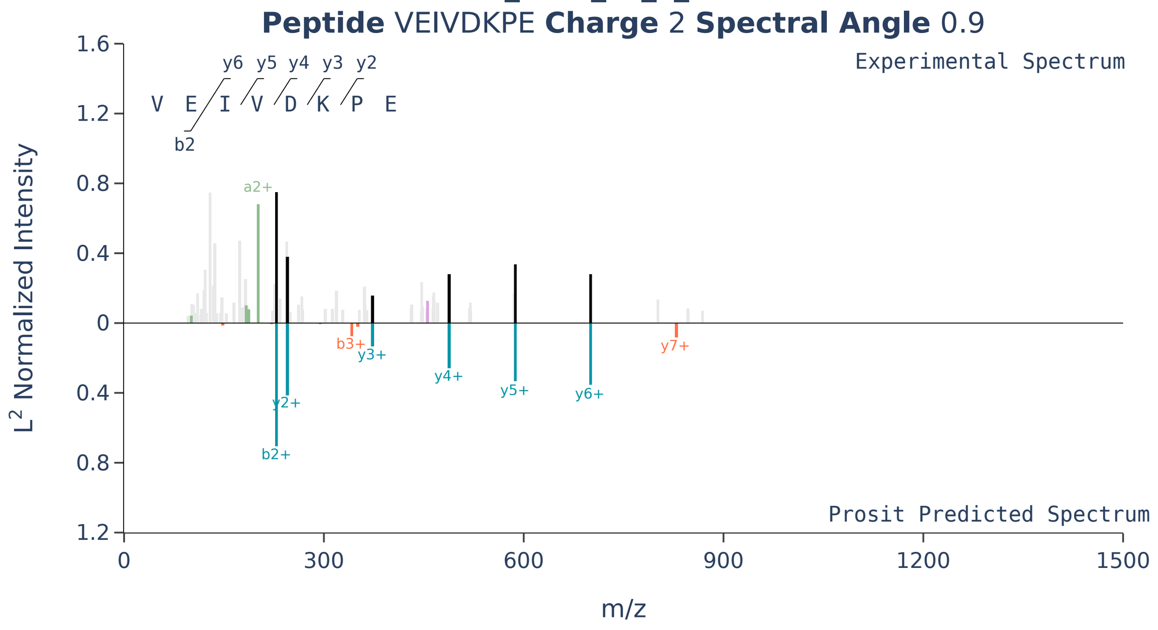

Source Fu_HeLa_inf_2_R2 **Scan** 3357
Peptide ETFQRNKPHIN **Charge** 3 **Spectral Angle** 0.74

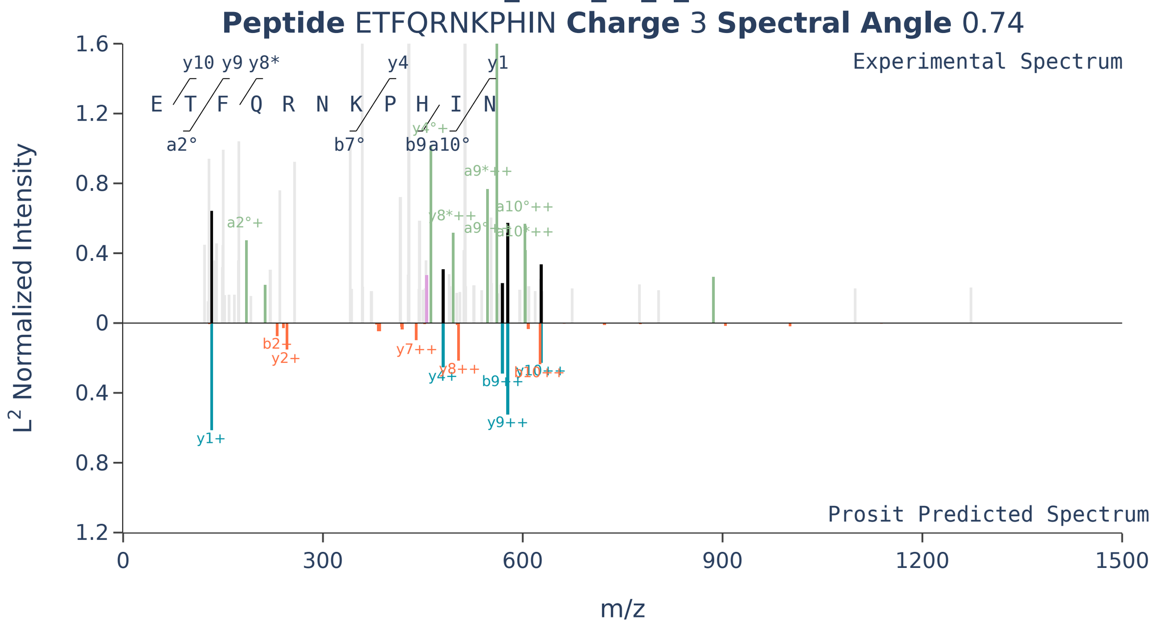

Source Fusion_Infected_HeLa_24h_PI_R1 **Scan** 18384
Peptide LPVGNPAEPSLMID **Charge** 2 **Spectral Angle** 0.64

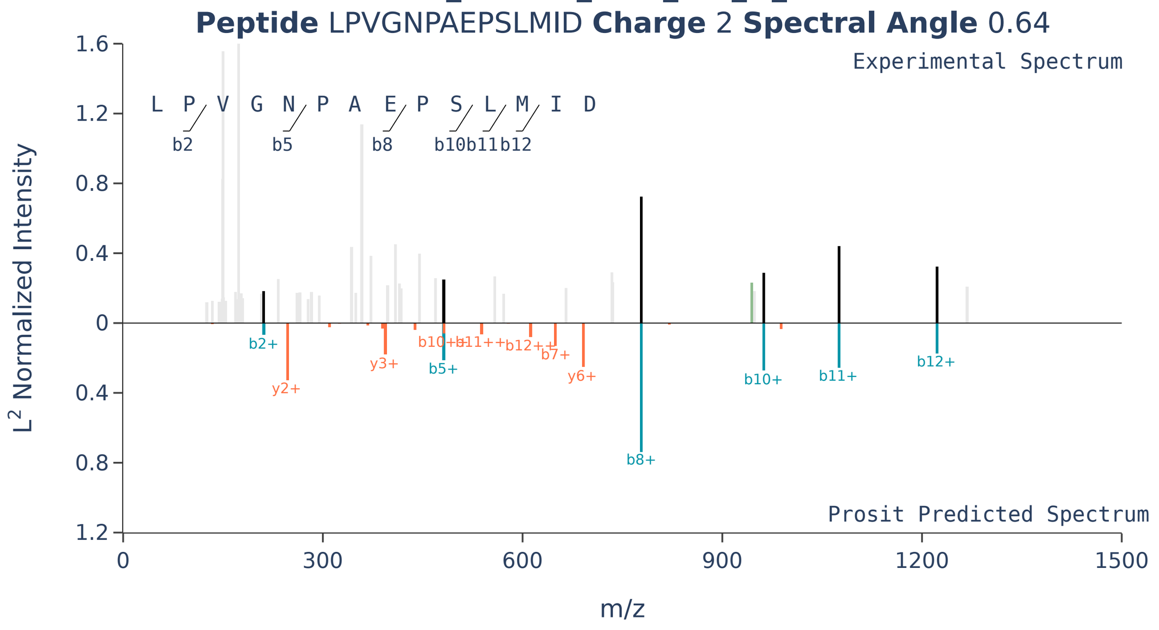

Source Fusion_Infected_HeLa_24h_PI_R1 **Scan** 3445
Peptide VAKNIKYNEEA **Charge** 3 **Spectral Angle** 0.8

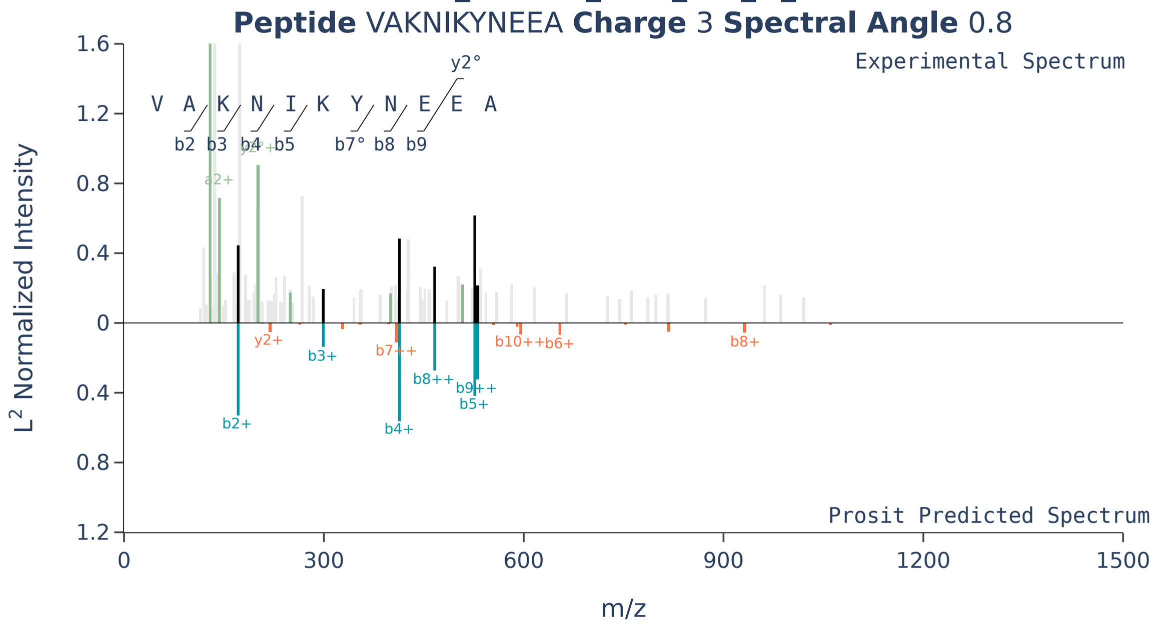

Source Fusion_Infected_HeLa_24h_PI_R2 **Scan** 6606
Peptide LSLIGKKEG **Charge** 2 **Spectral Angle** 0.82

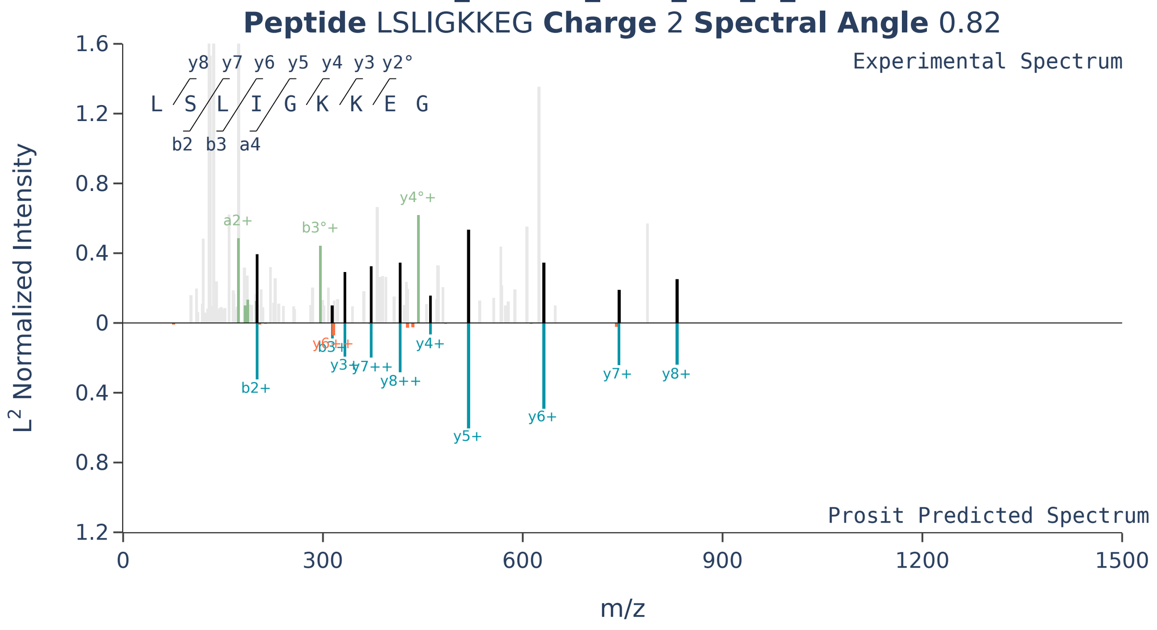

Source Fu_HeLa_inf_2_R2 **Scan** 7743
Peptide KALETSGVEMD **Charge** 2 **Spectral Angle** 0.84

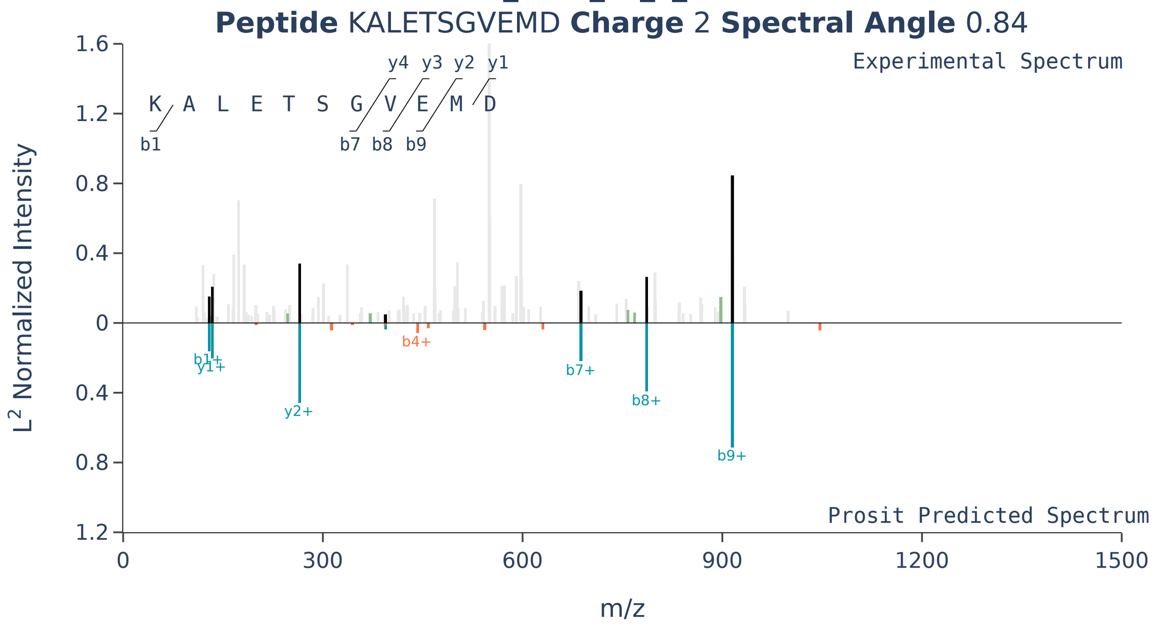

Source Fu_HeLa_inf_2_R2 **Scan** 1764
Peptide KYNEEARKKIQ **Charge** 3 **Spectral Angle** 0.85

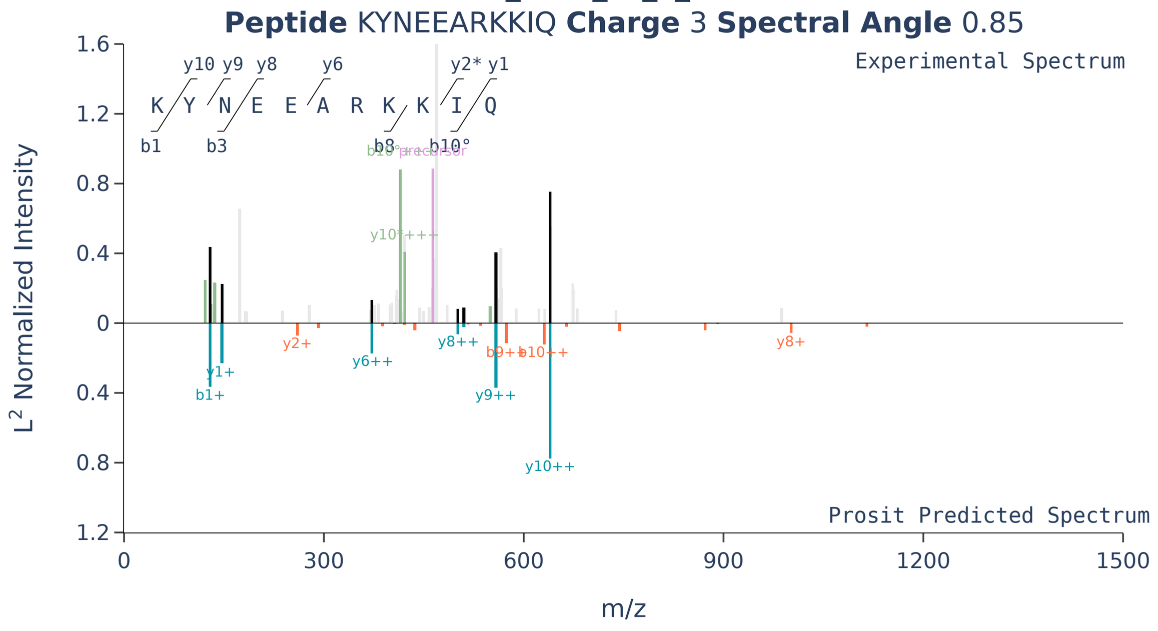

Source Fu_HeLa_inf_2_R2 **Scan** 1661
Peptide KKAPVRKVAA **Charge** 3 **Spectral Angle** 0.77

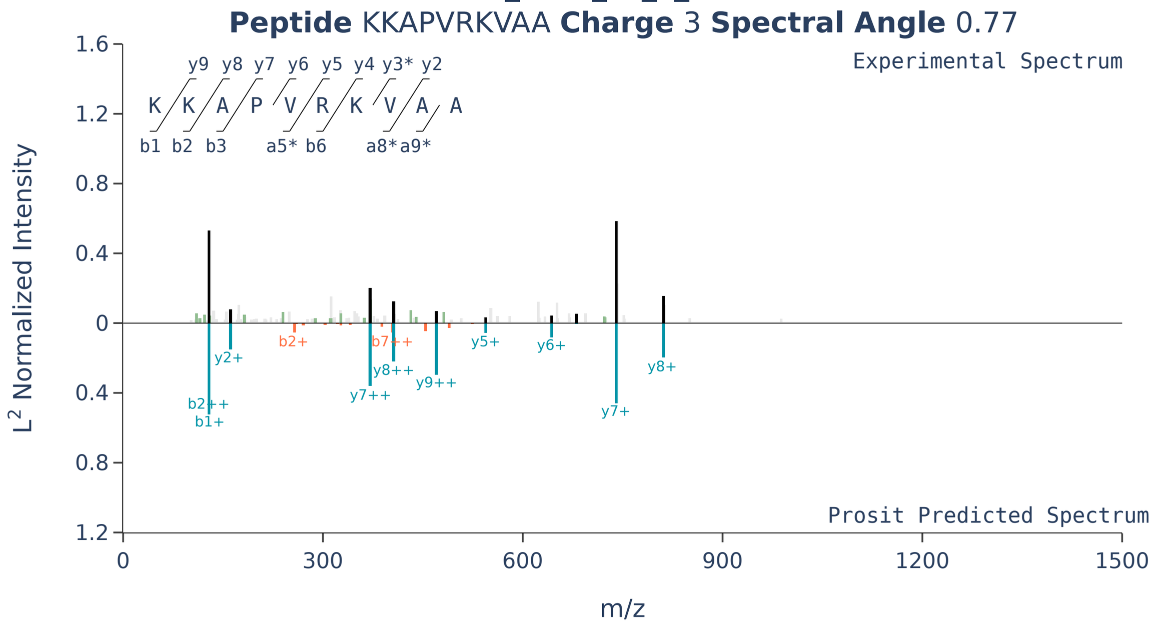

Source Fu_HeLa_inf_2_R2 **Scan** 12708
Peptide IVGGVIGA **Charge** 1 **Spectral Angle** 0.91

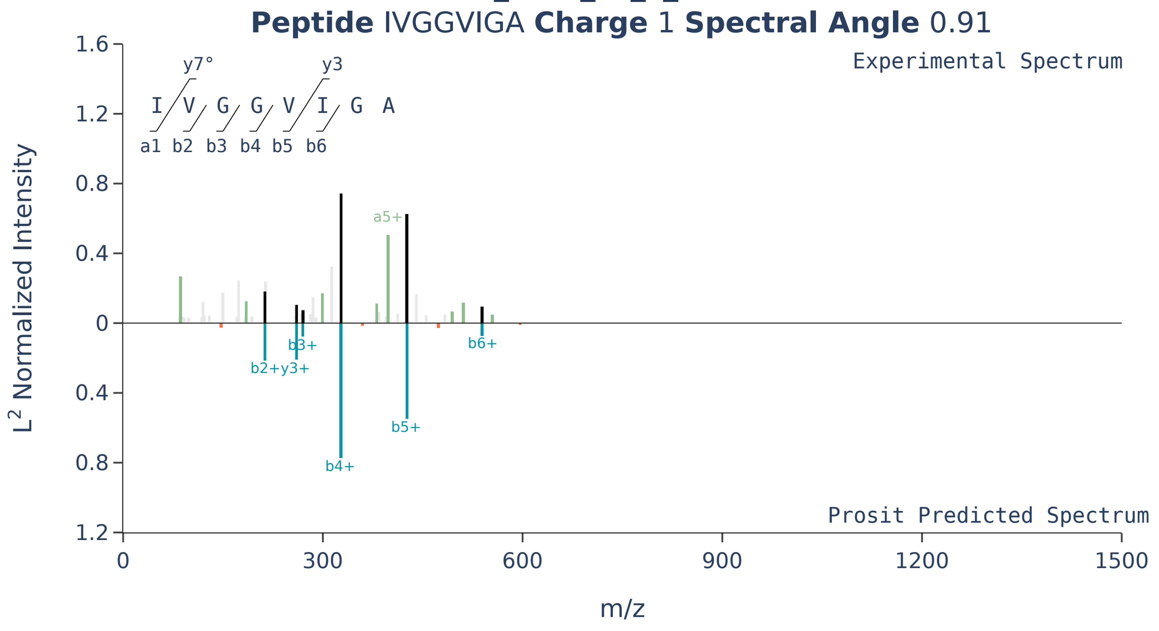

Source Fu_HeLa_inf_2_R2 **Scan** 8690
Peptide AIVTESLENKD **Charge** 2 **Spectral Angle** 0.78

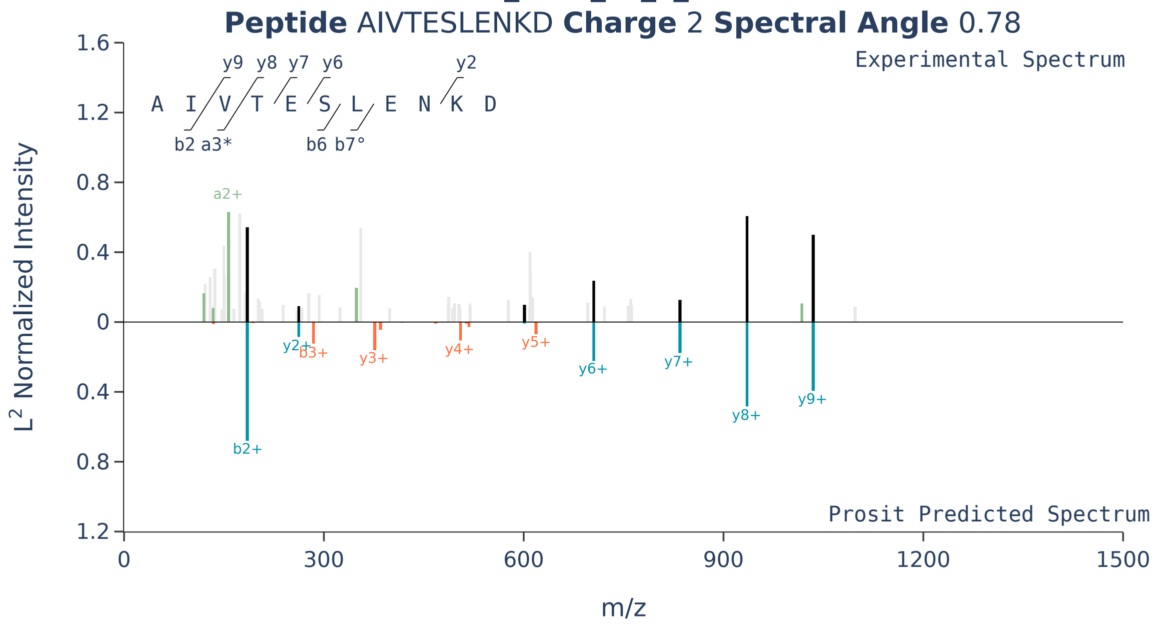

Source Fusion_Infected_HeLa_24h_PI_R2 **Scan** 4571
Peptide VQGGRVKDLPG **Charge** 2 **Spectral Angle** 0.8

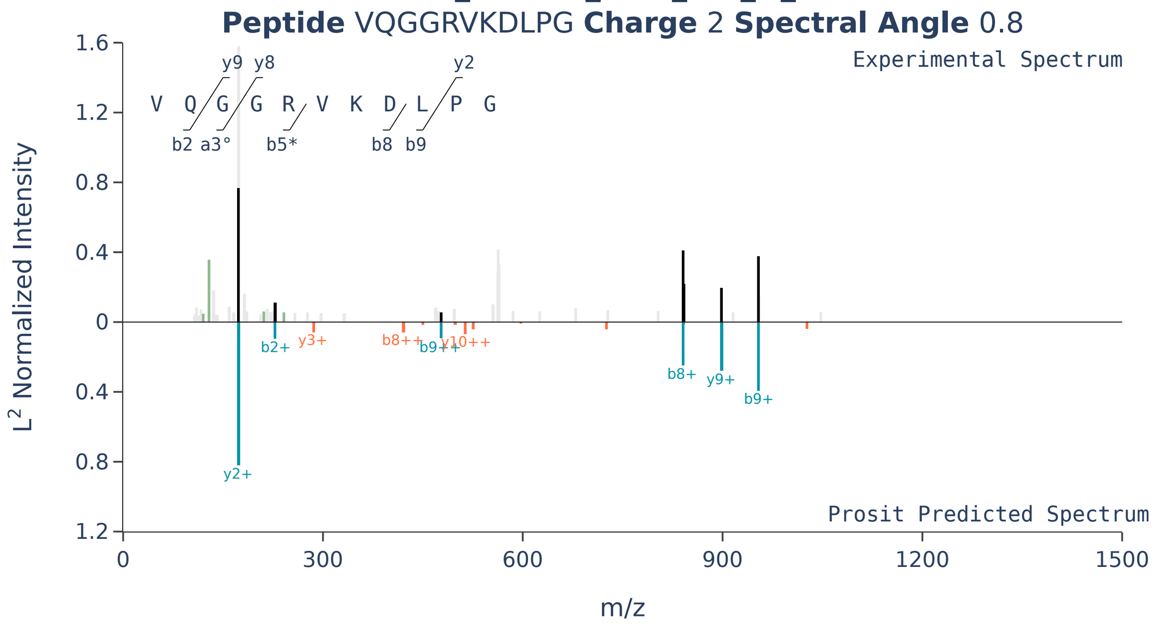

Source Fu_HeLa_inf_2_R2 **Scan** 5391
Peptide LIETPTKSS **Charge** 2 **Spectral Angle** 0.85

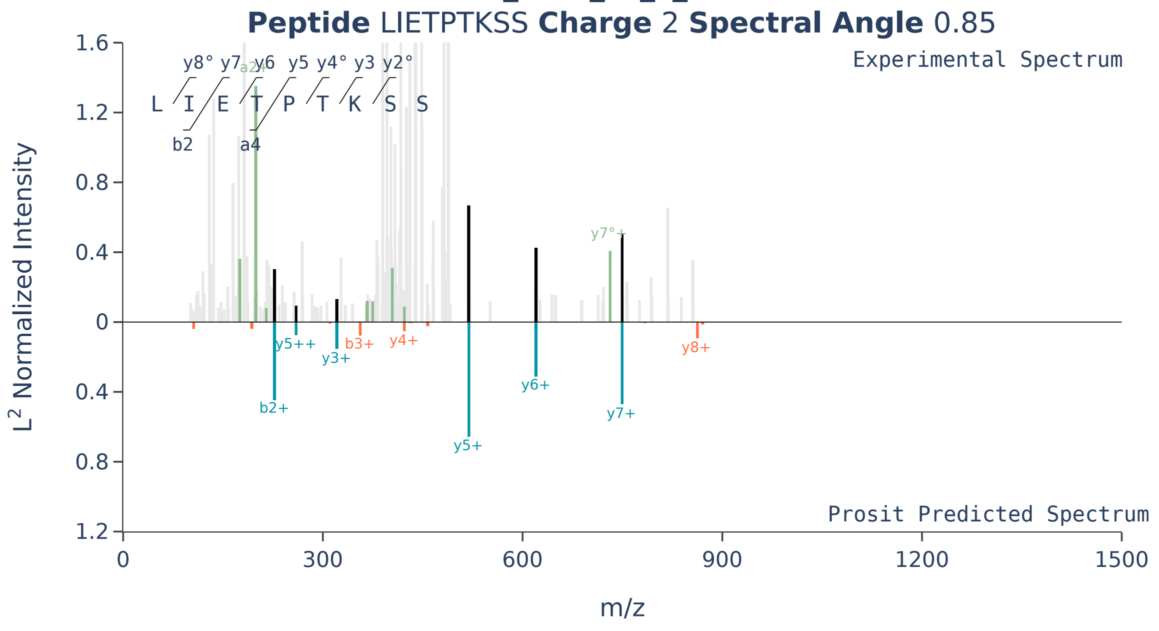

Source Fusion_Infected_HeLa_24h_PI_R2 **Scan** 6485
Peptide LVAIQANK **Charge** 2 **Spectral Angle** 0.89

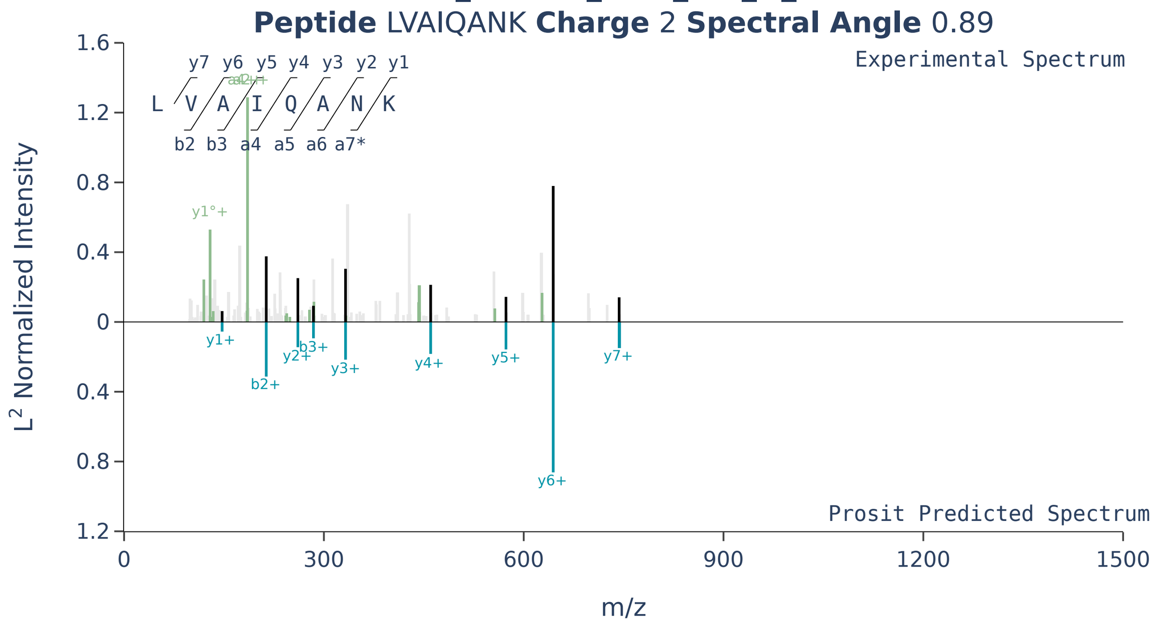

Source Fusion_Infected_HeLa_24h_PI_R1 **Scan** 3981
Peptide RVERVYSHPQYA **Charge** 3 **Spectral Angle** 0.81

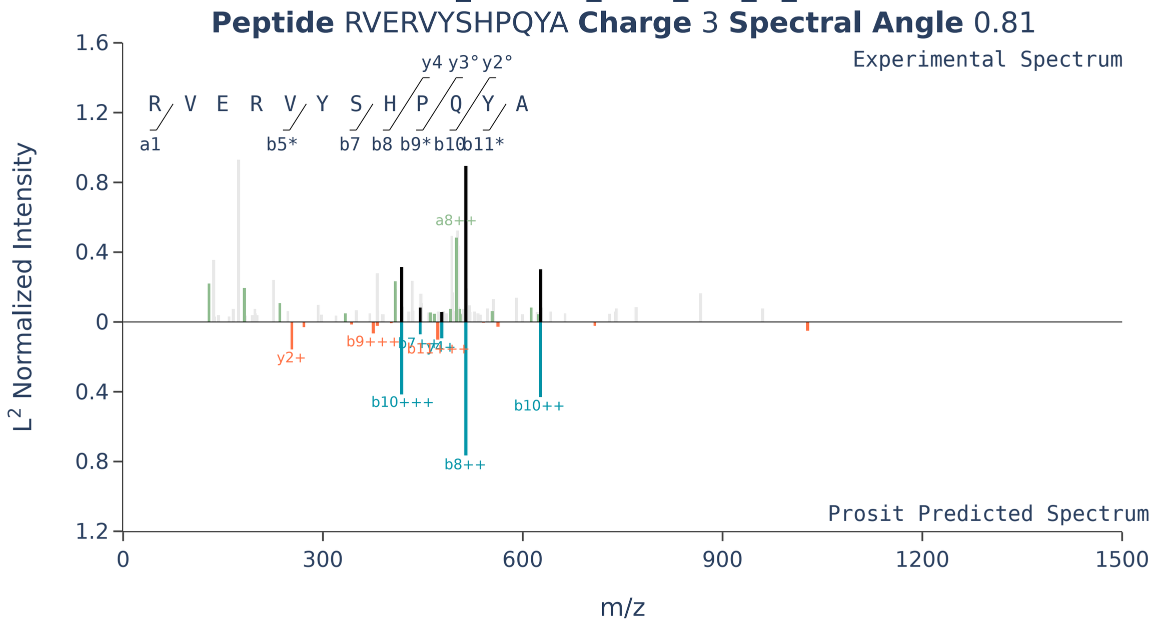

Source Fu_HeLa_inf_2_R1 **Scan** 13264
Peptide AVGPVSQL **Charge** 1 **Spectral Angle** 0.82

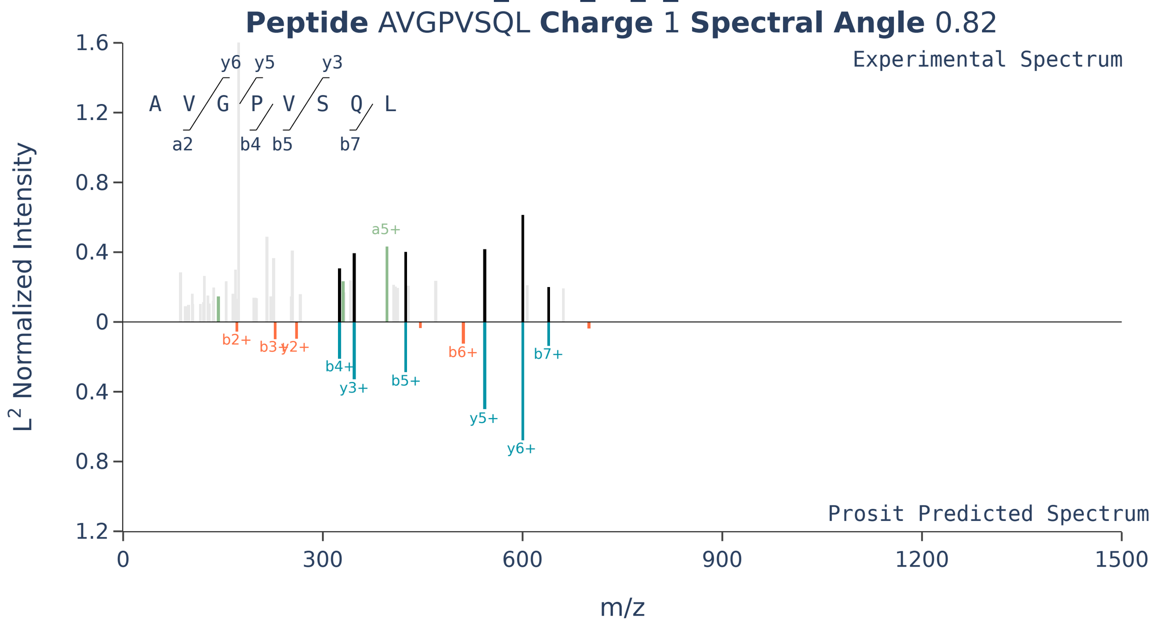

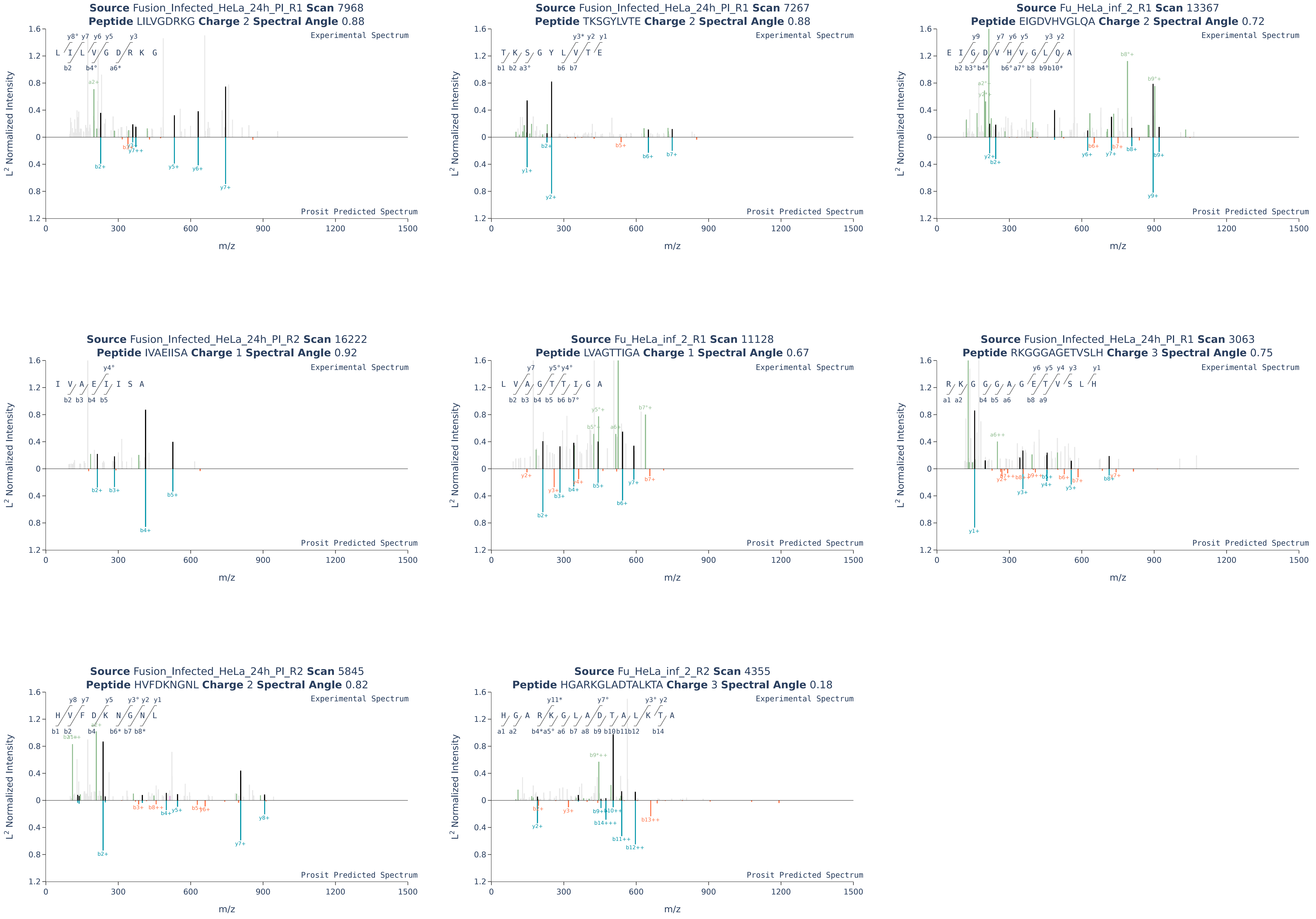
Source Fusion_Infected_HeLa_24h_PI_R1 Scan 7968
Peptide LILVGDRKG Charge 2 Spectral Angle 0.88
Source Fusion_Infected_HeLa_24h_PI_R1 Scan 7267
Peptide TKSGYLVTE Charge 2 Spectral Angle 0.88
Source Fu_HeLa_inf_2_R1 Scan 13367
Peptide EIGDVHVGLQA Charge 2 Spectral Angle 0.72
Source Fusion_Infected_HeLa_24h_PI_R2 Scan 16222
Peptide IVAEIISA Charge 1 Spectral Angle 0.92
Source Fu_HeLa_inf_2_R1 Scan 11128
Peptide LVAGTTIGA Charge 1 Spectral Angle 0.67
Source Fusion_Infected_HeLa_24h_PI_R1 Scan 3063
Peptide RKGGGAGETVSLH Charge 3 Spectral Angle 0.75
Source Fusion_Infected_HeLa_24h_PI_R2 Scan 5845
Peptide HVFDKNGNL Charge 2 Spectral Angle 0.82
Source Fu_HeLa_inf_2_R2 Scan 4355
Peptide HGARKGLADTALKTA Charge 3 Spectral Angle 0.18
Experimental Spectrum
Prosit Predicted Spectrum
L² Normalized Intensity
m/z

inSPIRE Spectral Plotting for cov-a549-240117

Experimental Spectrum Colour Code:

- Experimental peak matched to a Prosit predicted peak.
- Possible ion unknown to Prosit.
- Precursor matched peak.
- Experimental peak not matched to any potential ion.

Prosit Spectrum Colour Code:

- Prosit predicted peak matched to experimental spectrum.
- Prosit predicted peak not matched to experimental spectrum.

Additional Notes:

° indicates an ion with loss of H_2O.

* indicates an ion with loss of NH_3.

Source HLA_A549_SarsCoV2infected_fxn03 **Scan** 27267
Peptide KNIDGYFKIY **Charge** 2 **Spectral Angle** 0.9

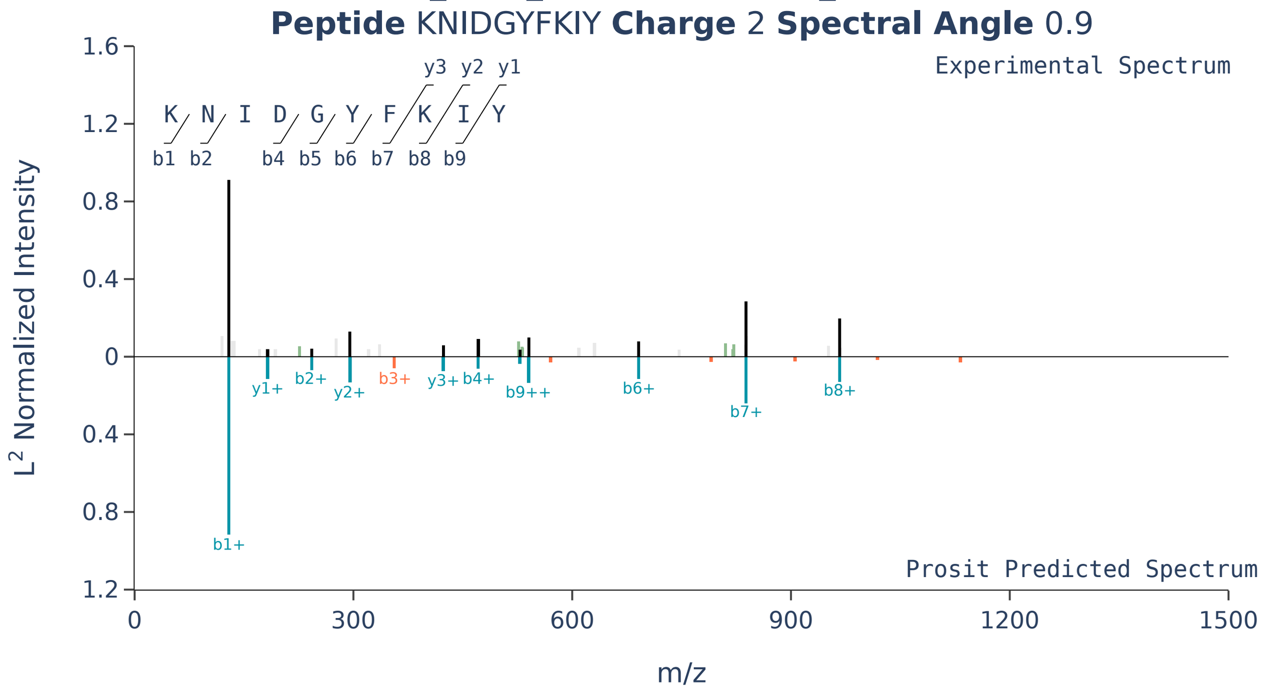

Source HLA_A549_SarsCoV2infected_fxn01 **Scan** 28679
Peptide EEFEPSTQYEY **Charge** 2 **Spectral Angle** 0.87

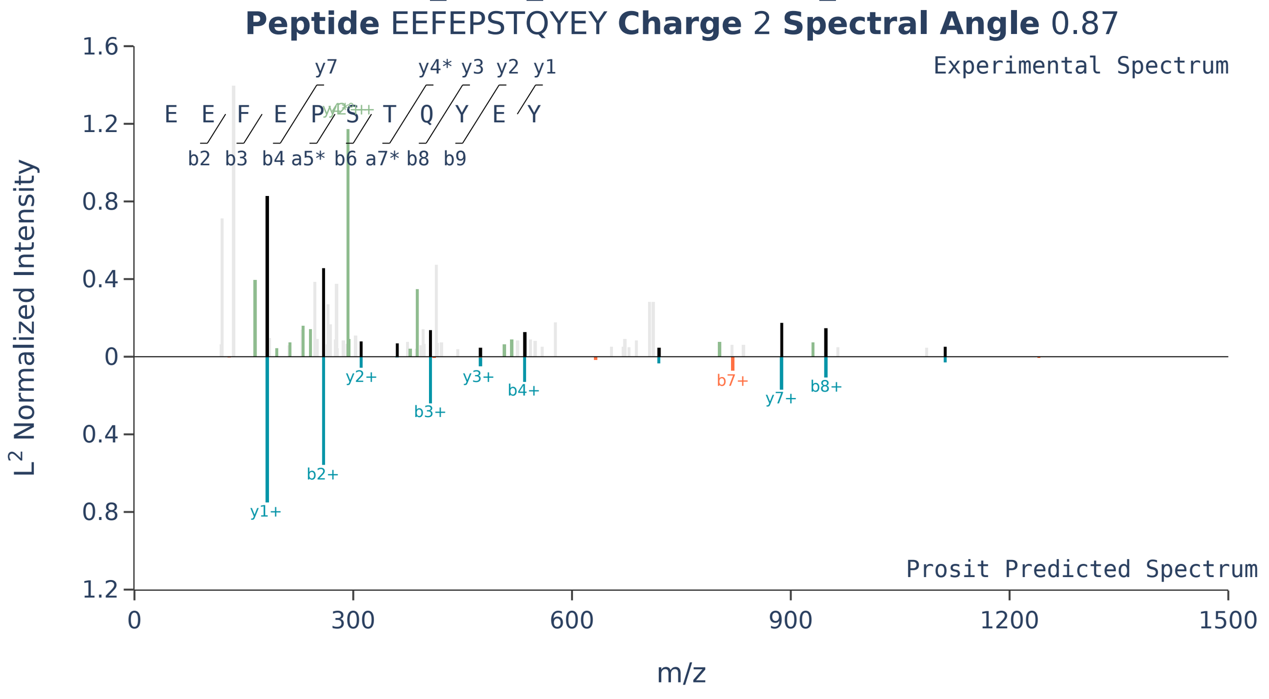

Source HLA_A549_SarsCoV2infected_fxn01 **Scan** 21359
Peptide FAVDAAKAY **Charge** 2 **Spectral Angle** 0.91

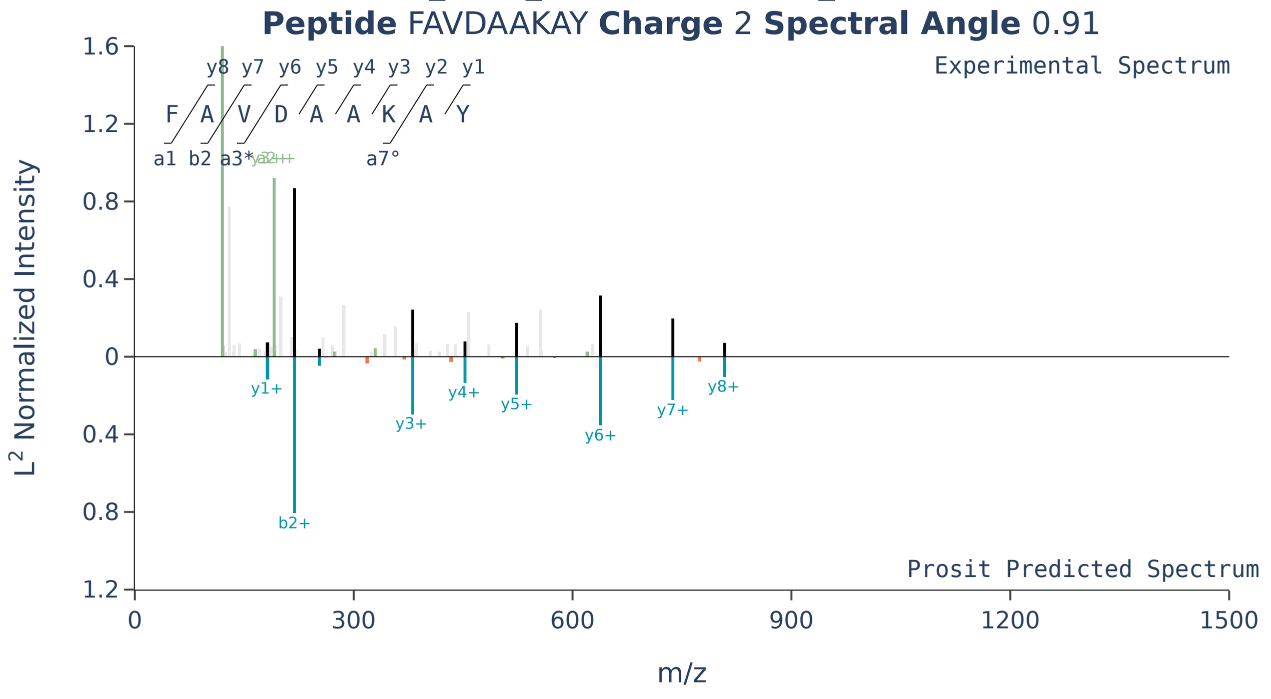

Source HLA_A549_SarsCoV2infected_fxn03 **Scan** 29284
Peptide QLTPTWRVY **Charge** 2 **Spectral Angle** 0.93

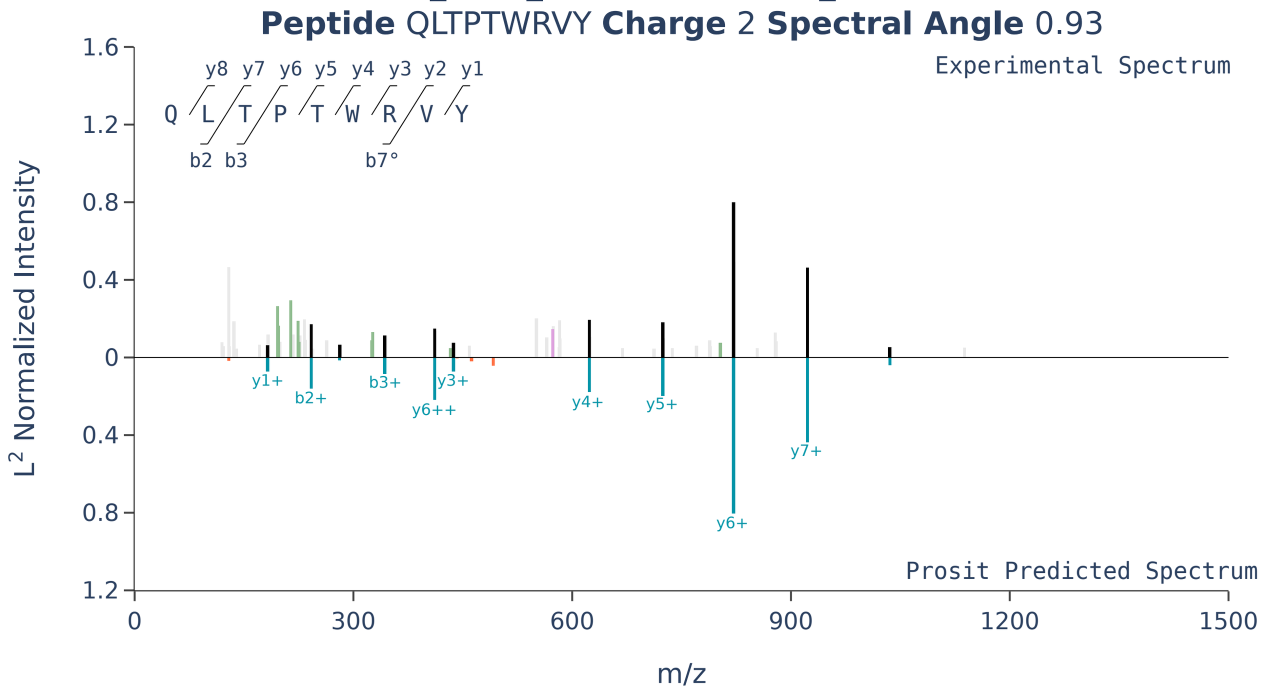

Source HLA_A549_SarsCoV2infected_fxn03 **Scan** 24247
Peptide VGYLQPRTF **Charge** 2 **Spectral Angle** 0.93

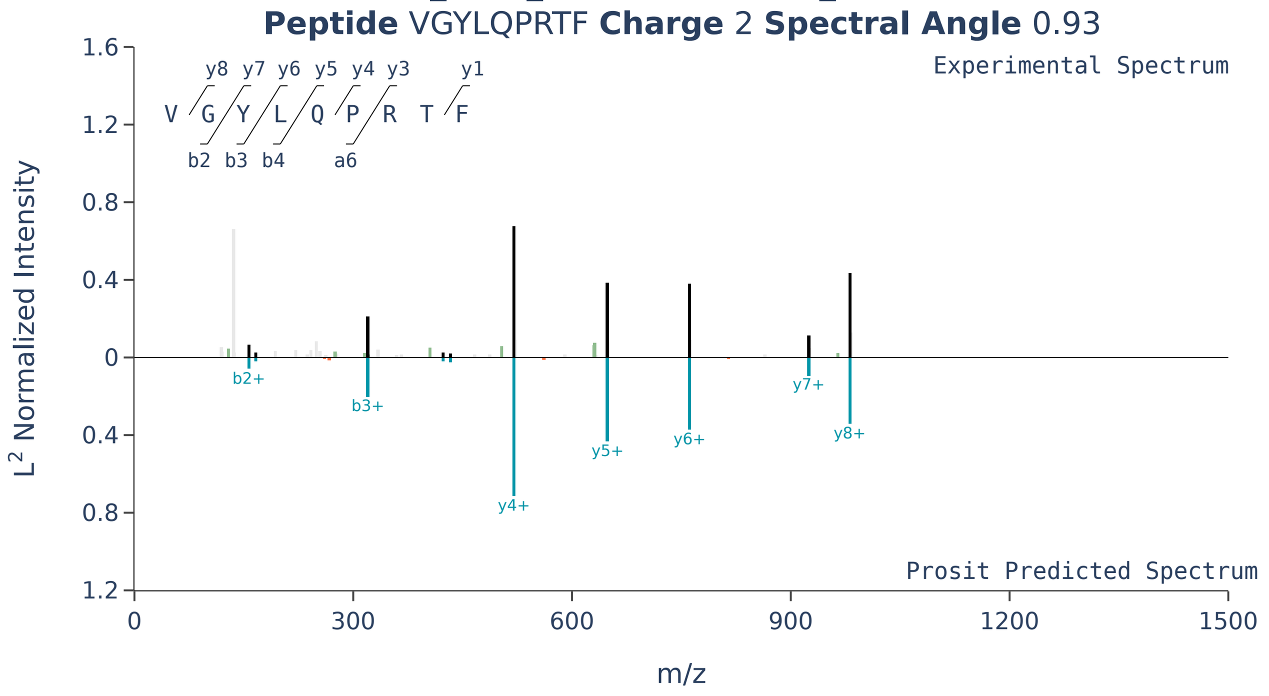

Source HLA_A549_SarsCoV2infected_fxn01 **Scan** 17801
Peptide FASEAARVV **Charge** 2 **Spectral Angle** 0.95

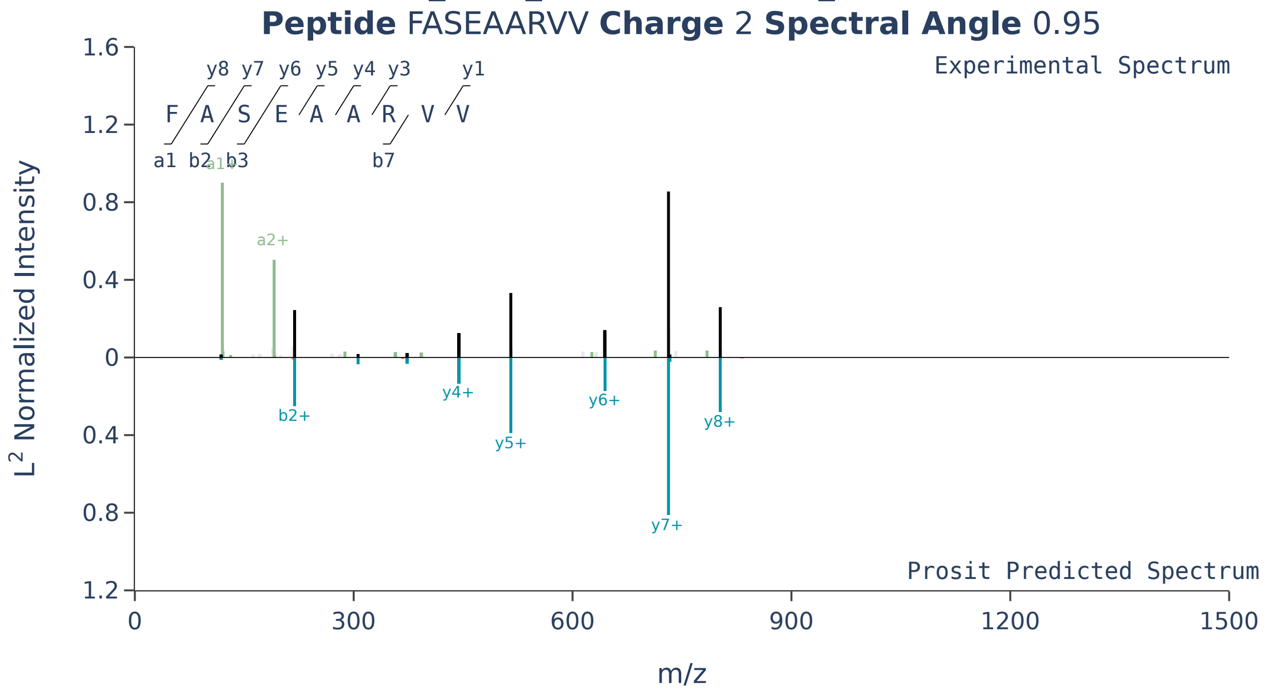

Source HLA_A549_SarsCoV2infected_fxn01 **Scan** 21905
Peptide MESEFRVY **Charge** 2 **Spectral Angle** 0.9

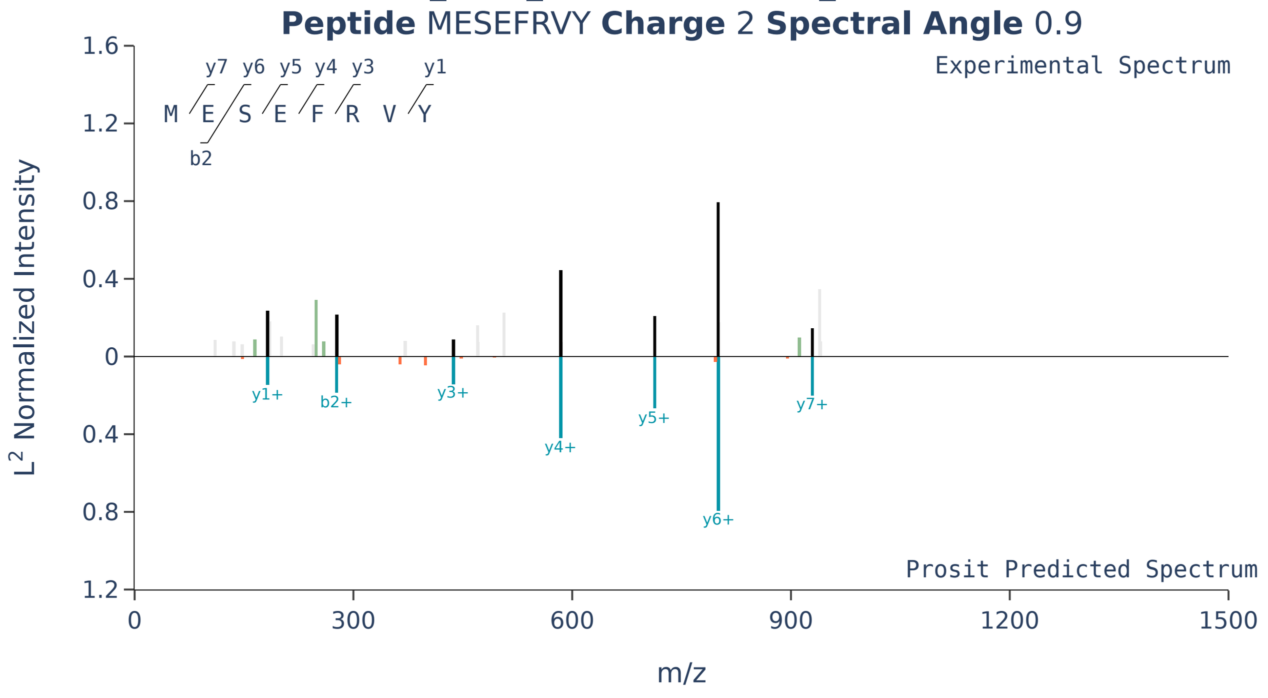

Source HLA_A549_SarsCoV2infected_fxn01 **Scan** 27681
Peptide LEDKAFQL **Charge** 2 **Spectral Angle** 0.83

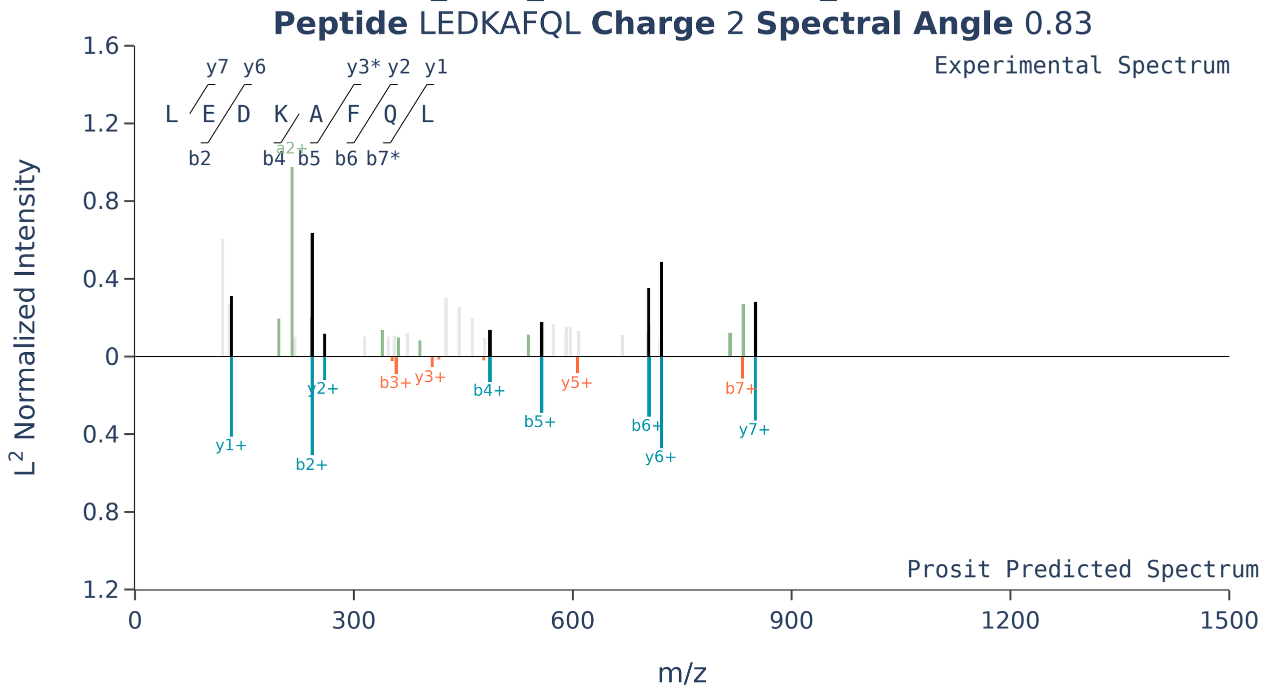

Source HLA_A549_SarsCoV2infected_fxn02 **Scan** 20722
Peptide NATNVVIKV **Charge** 2 **Spectral Angle** 0.89

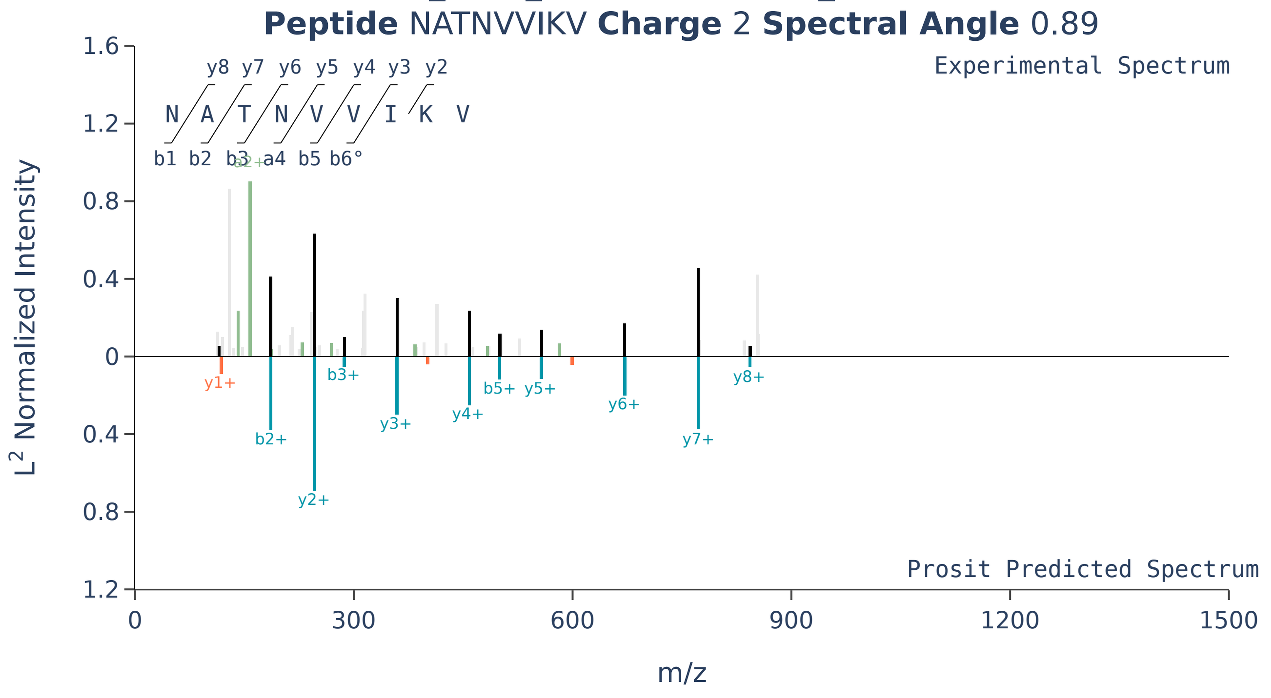

Source HLA_A549_SarsCoV2infected_fxn01 **Scan** 25488
Peptide LATNNLVVM **Charge** 2 **Spectral Angle** 0.96

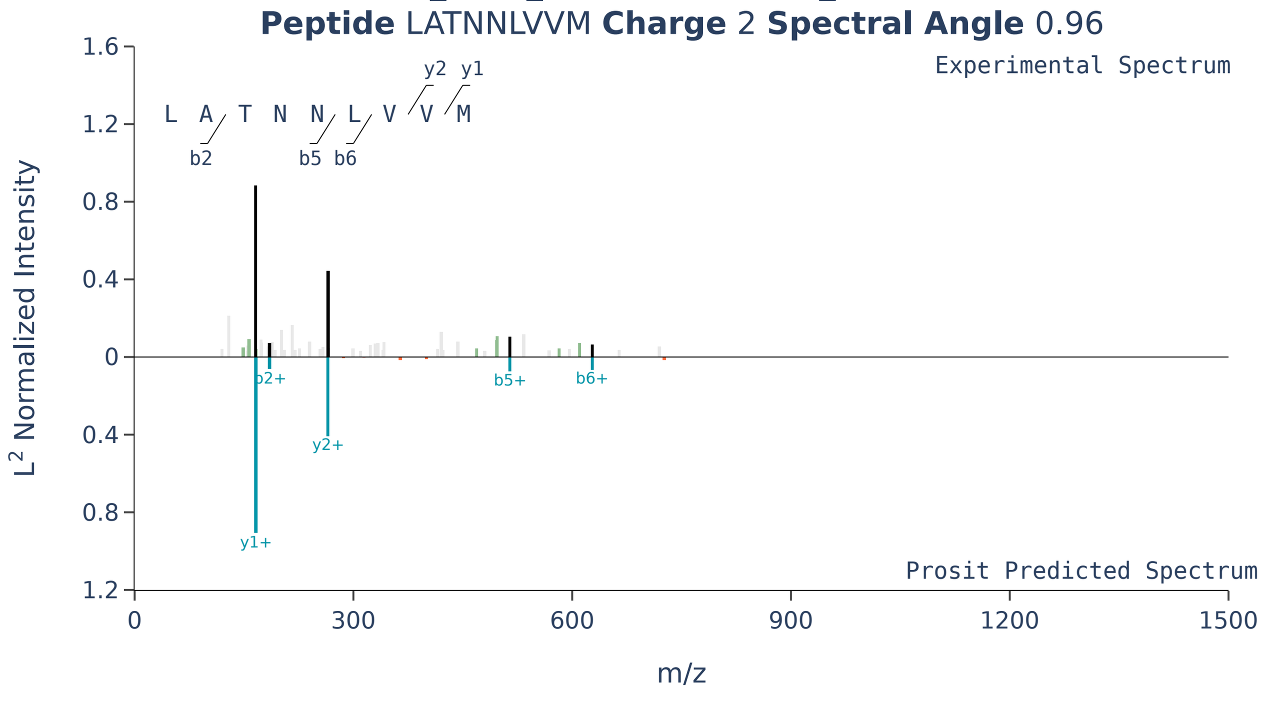

Source HLA_A549_SarsCoV2infected_fxn01 **Scan** 41094
Peptide QEILGTVSW **Charge** 2 **Spectral Angle** 0.85

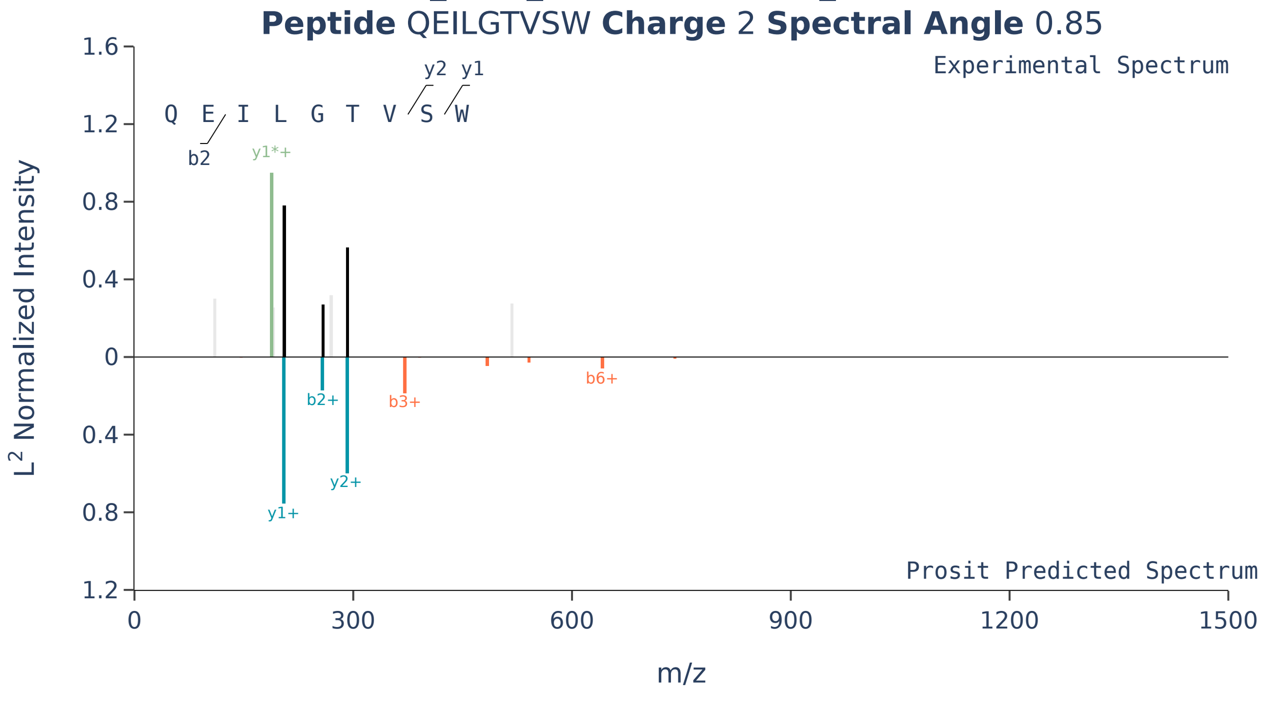

Source HLA_A549_SarsCoV2infected_fxn02 **Scan** 12959
Peptide VATSRTLSY **Charge** 2 **Spectral Angle** 0.85

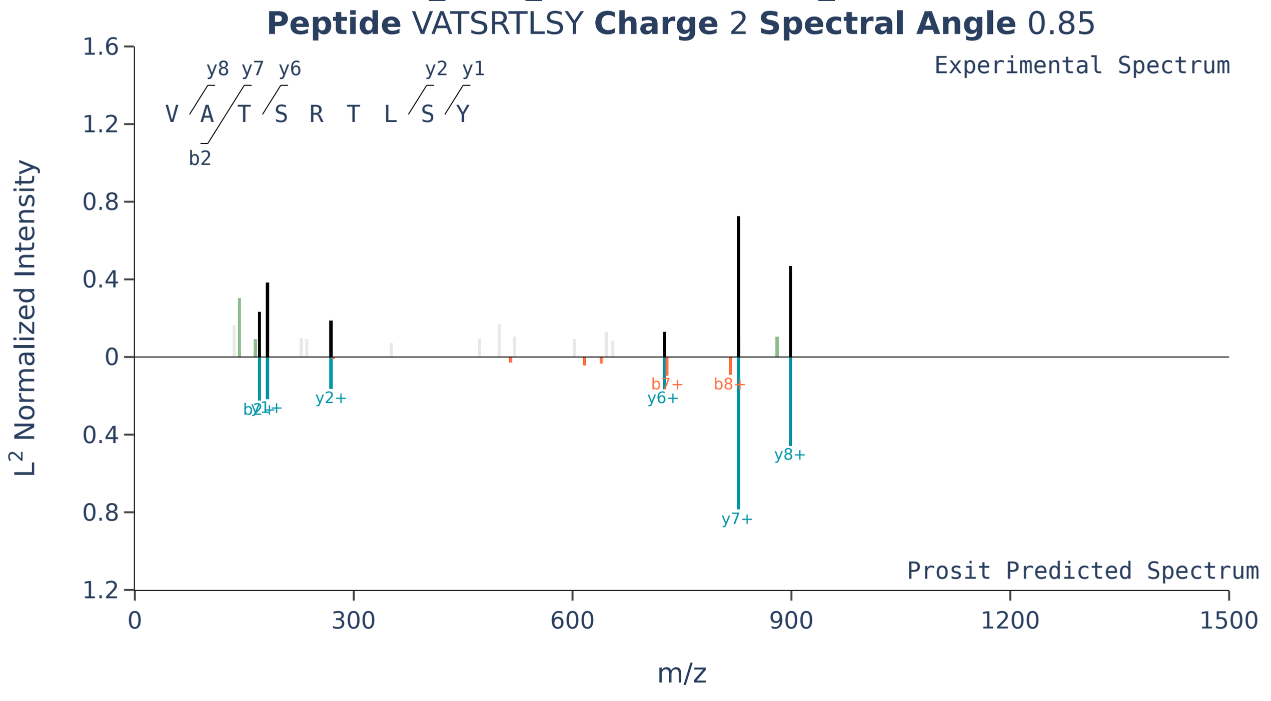

Source HLA_A549_SarsCoV2infected_fxn01 **Scan** 19666
Peptide EIKDATPSDF **Charge** 2 **Spectral Angle** 0.82

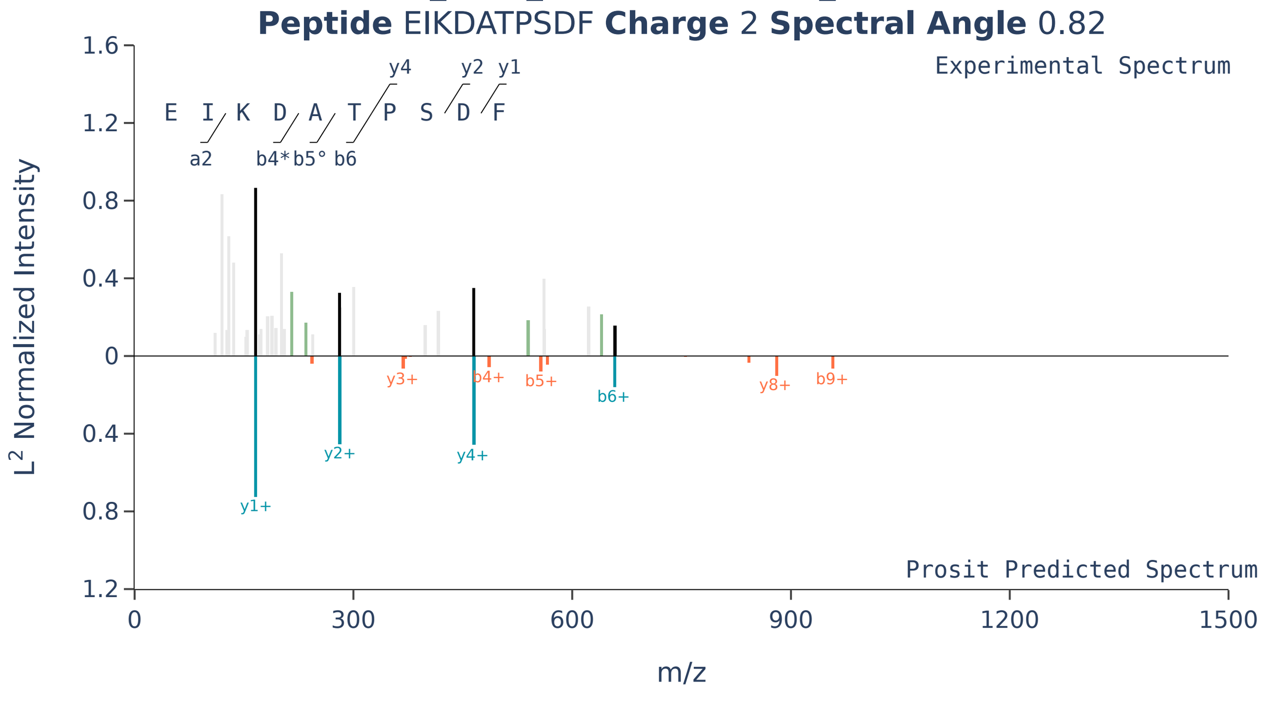

Source HLA_A549_SarsCoV2infected_fxn03 **Scan** 25589
Peptide FAFACPDGVKHVY **Charge** 3 **Spectral Angle** 0.53

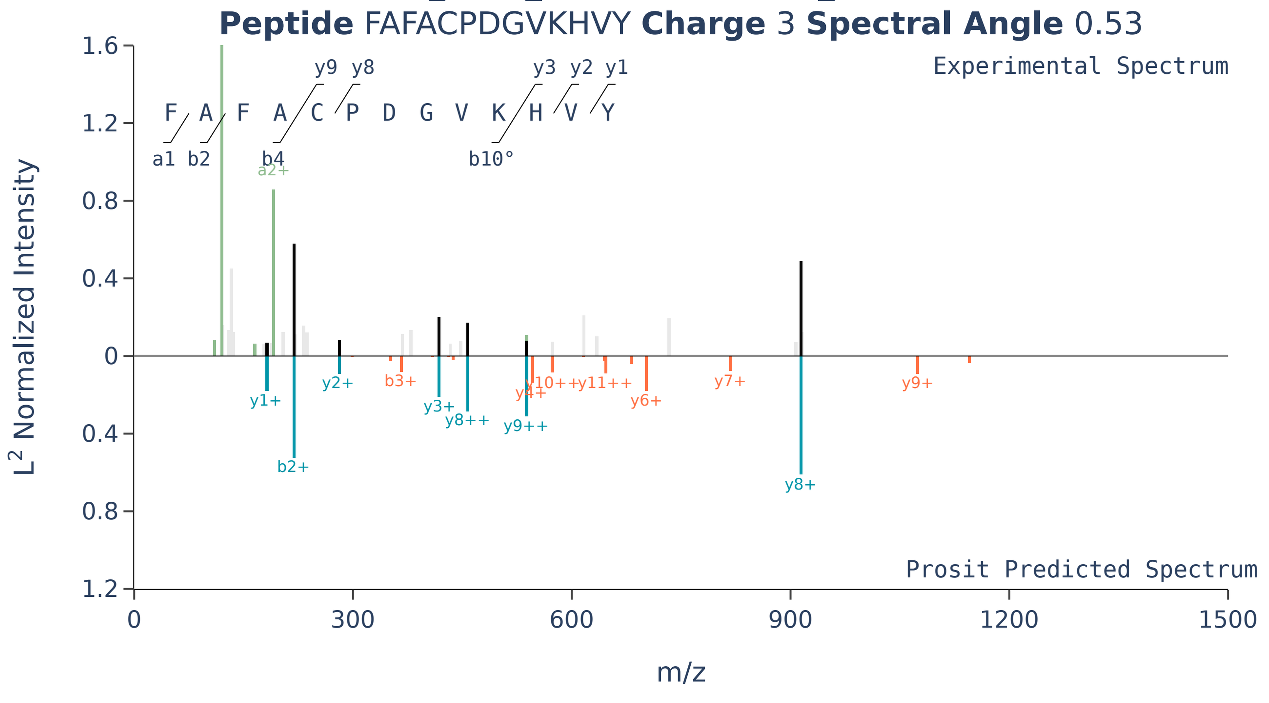

inSPIRE Spectral Plotting for cov-calu-240117

Experimental Spectrum Colour Code:

- Experimental peak matched to a Prosit predicted peak.
- Possible ion unknown to Prosit.
- Precursor matched peak.
- Experimental peak not matched to any potential ion.

Prosit Spectrum Colour Code:

- Prosit predicted peak matched to experimental spectrum.
- Prosit predicted peak not matched to experimental spectrum.

Additional Notes:

° indicates an ion with loss of H_2O.

* indicates an ion with loss of NH_3.

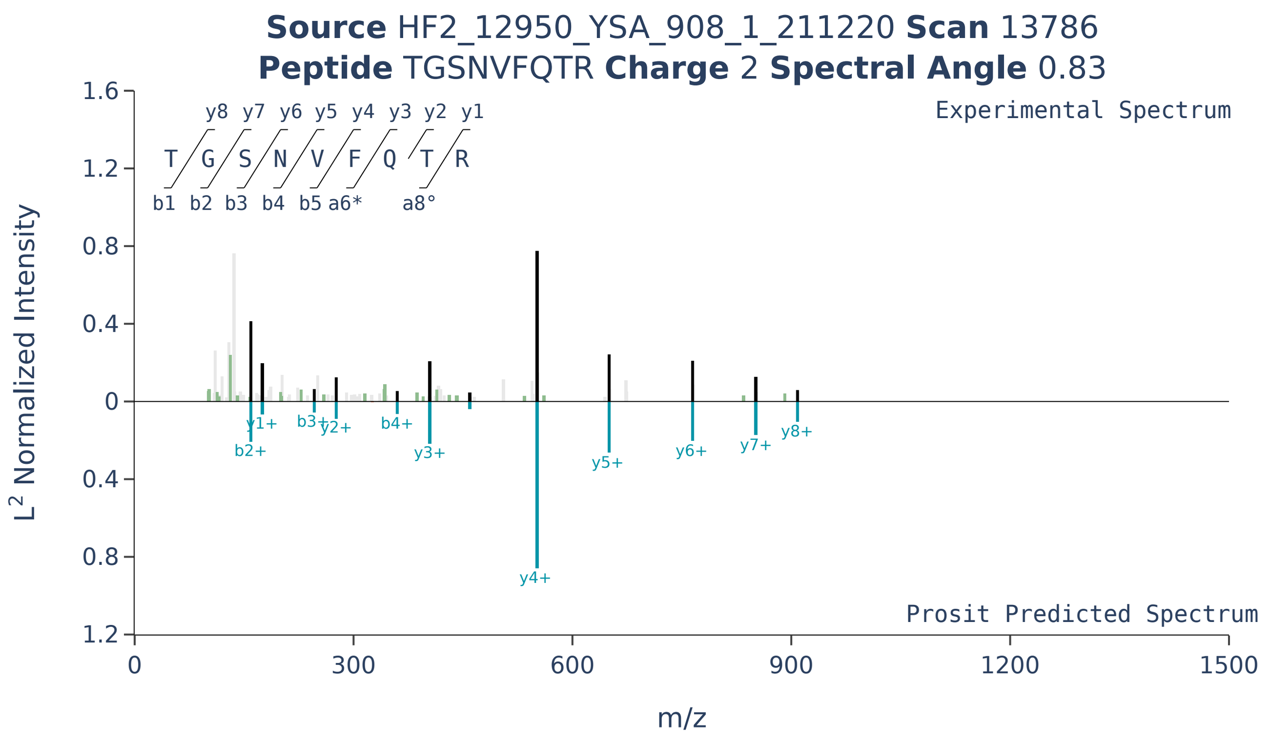
Source HF2_12950_YSA_908_1_211220 Scan 13786
Peptide TGSNVFQTR Charge 2 Spectral Angle 0.83
Experimental Spectrum
Prosit Predicted Spectrum
L² Normalized Intensity
m/z

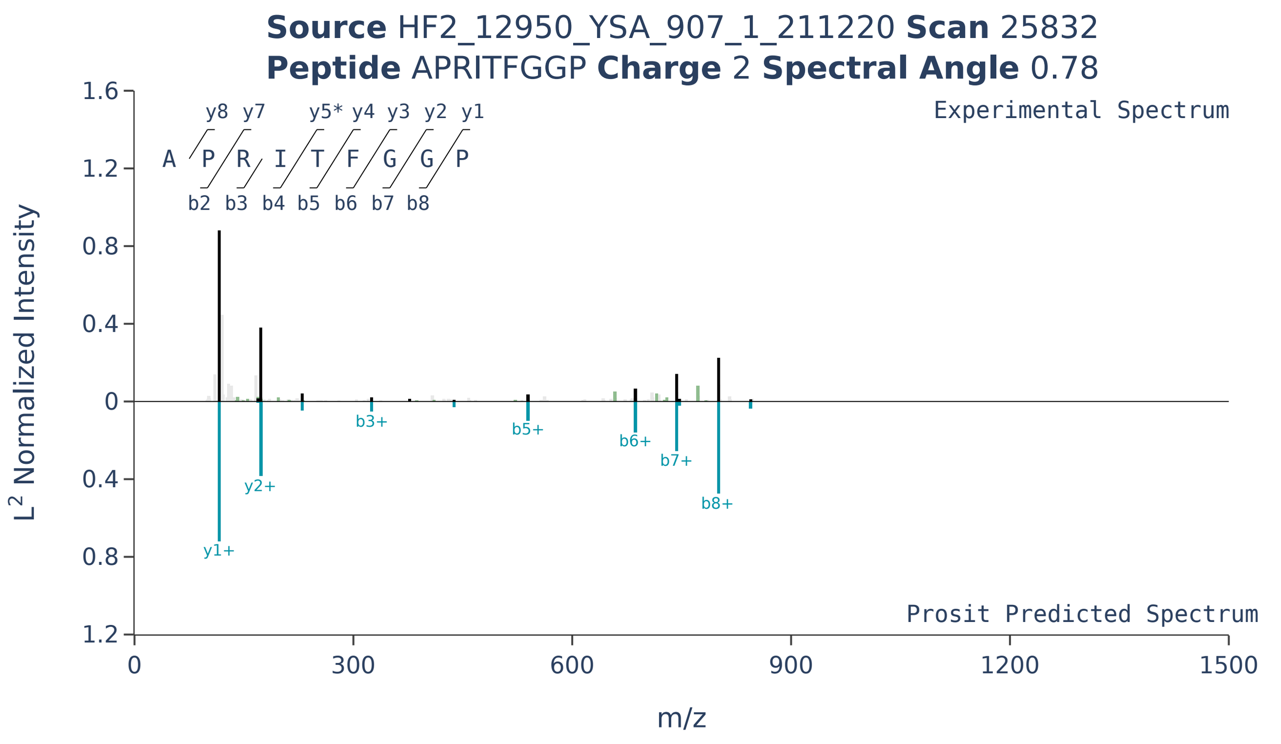
Source HF2_12950_YSA_907_1_211220 Scan 25832
Peptide APRITFGGP Charge 2 Spectral Angle 0.78
Experimental Spectrum
Prosit Predicted Spectrum
L² Normalized Intensity
m/z

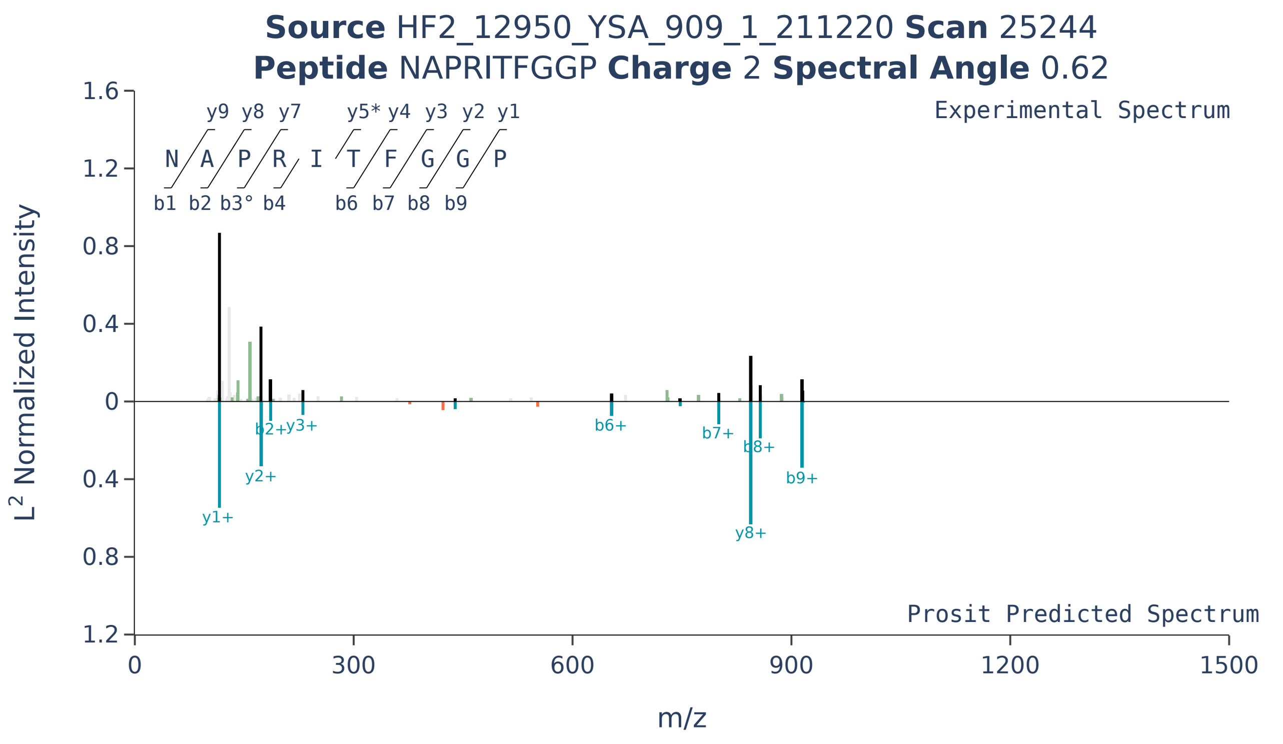
Source HF2_12950_YSA_909_1_211220 Scan 25244
Peptide NAPRITFGGP Charge 2 Spectral Angle 0.62
Experimental Spectrum
Prosit Predicted Spectrum
L² Normalized Intensity
m/z

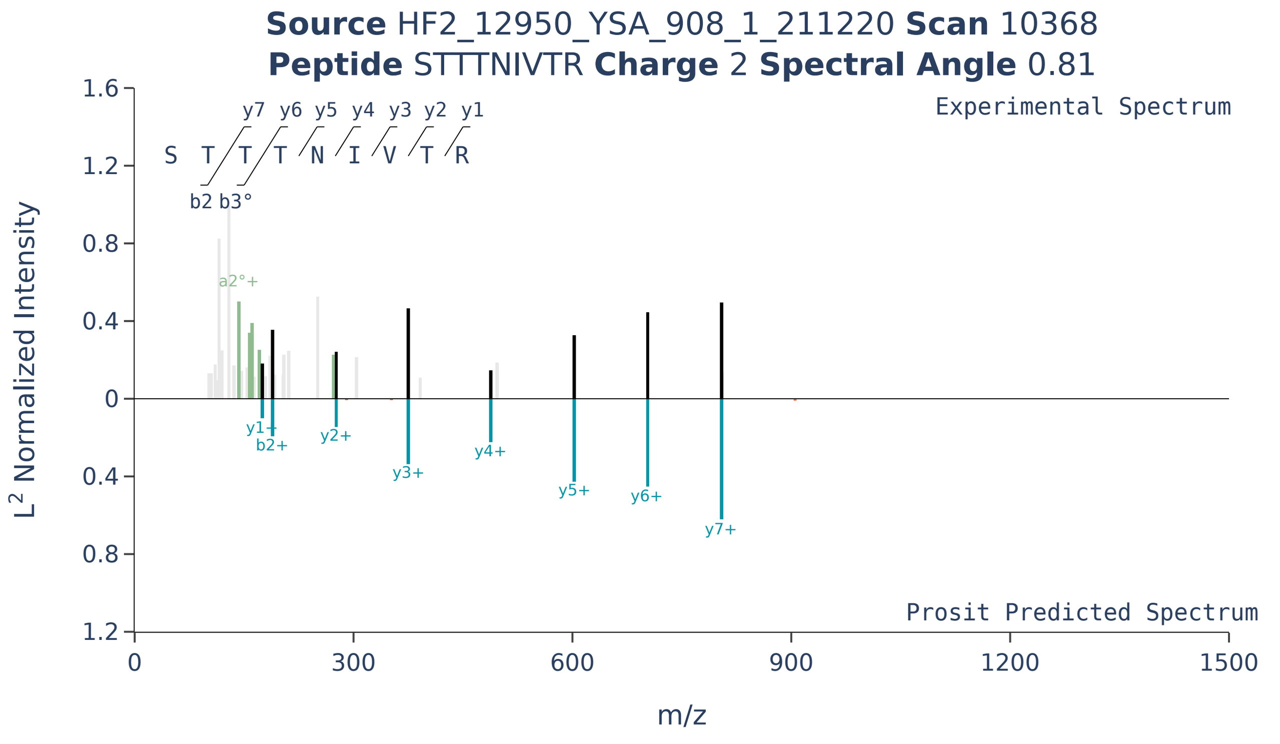
Source HF2_12950_YSA_908_1_211220 Scan 10368
Peptide STTTNIVTR Charge 2 Spectral Angle 0.81
Experimental Spectrum
Prosit Predicted Spectrum
L² Normalized Intensity
m/z

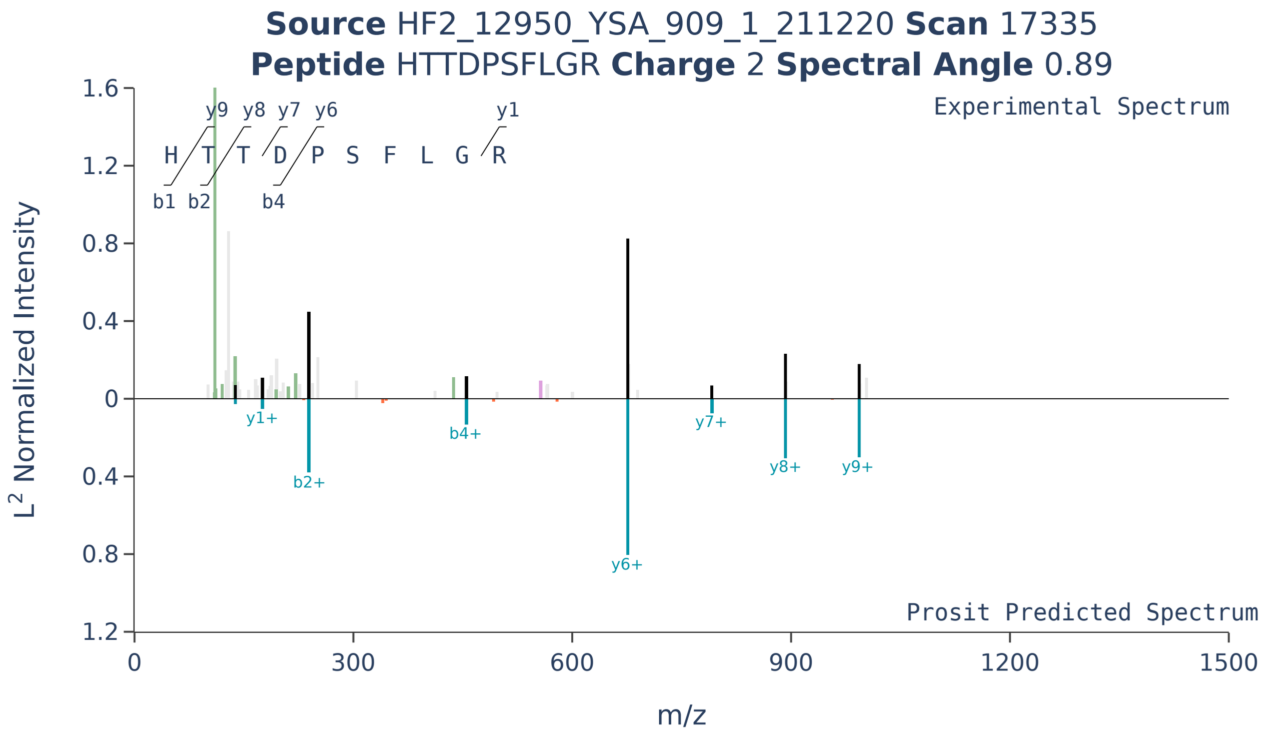
Source HF2_12950_YSA_909_1_211220 Scan 17335
Peptide HTTDPSFLGR Charge 2 Spectral Angle 0.89
Experimental Spectrum
Prosit Predicted Spectrum
L² Normalized Intensity
m/z

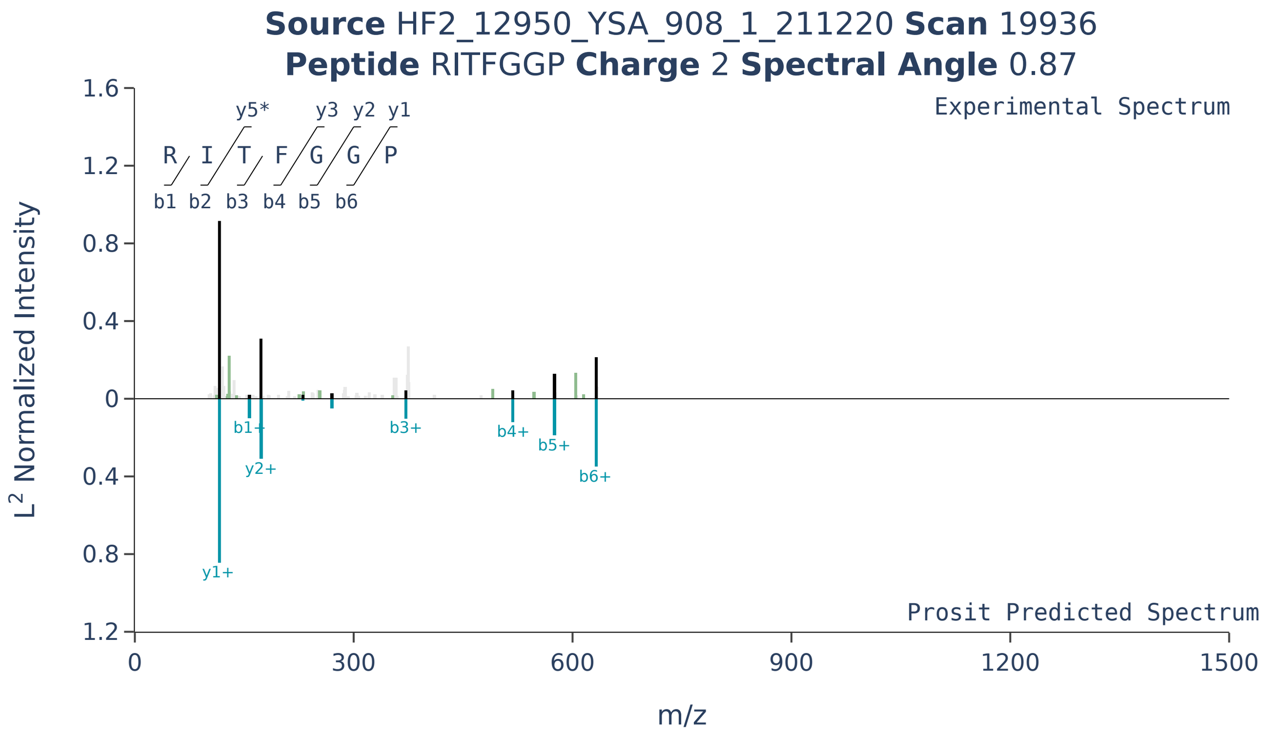
Source HF2_12950_YSA_908_1_211220 Scan 19936
Peptide RITFGGP Charge 2 Spectral Angle 0.87
Experimental Spectrum
Prosit Predicted Spectrum
L² Normalized Intensity
m/z

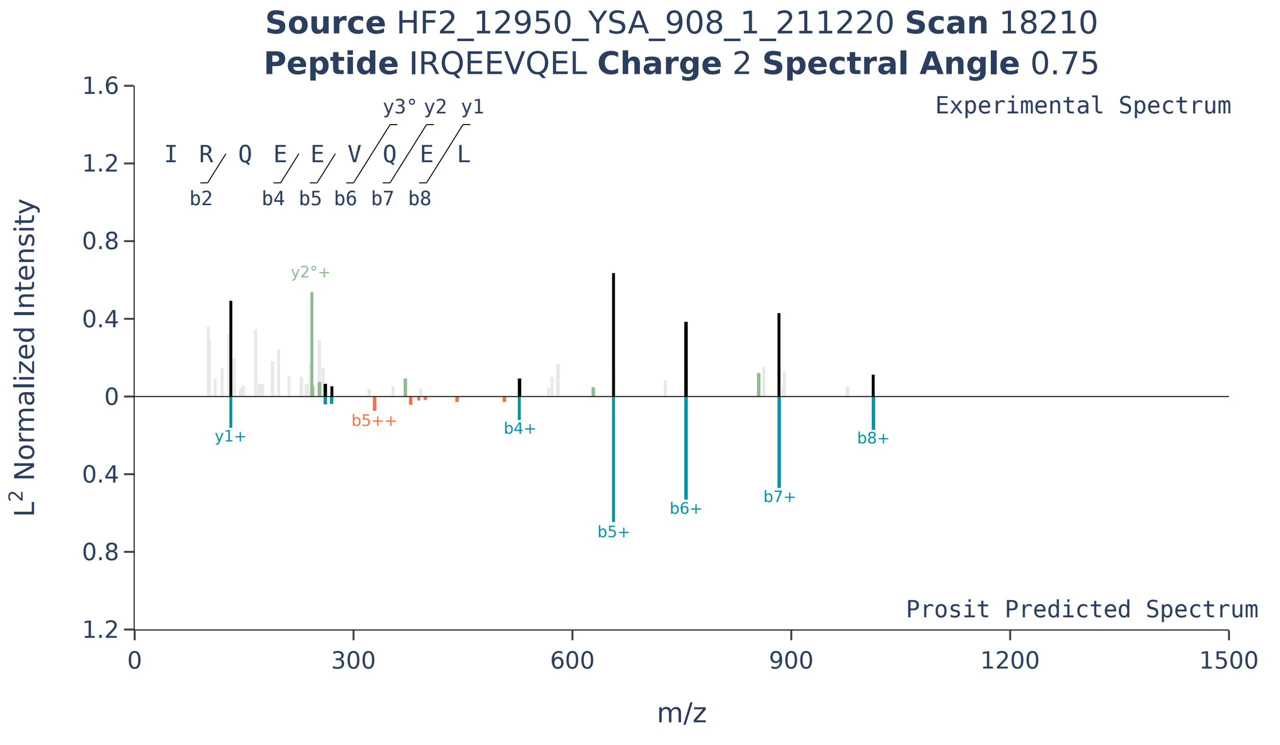
Source HF2_12950_YSA_908_1_211220 Scan 18210
Peptide IRQEEVQEL Charge 2 Spectral Angle 0.75
Experimental Spectrum
Prosit Predicted Spectrum
L² Normalized Intensity
m/z

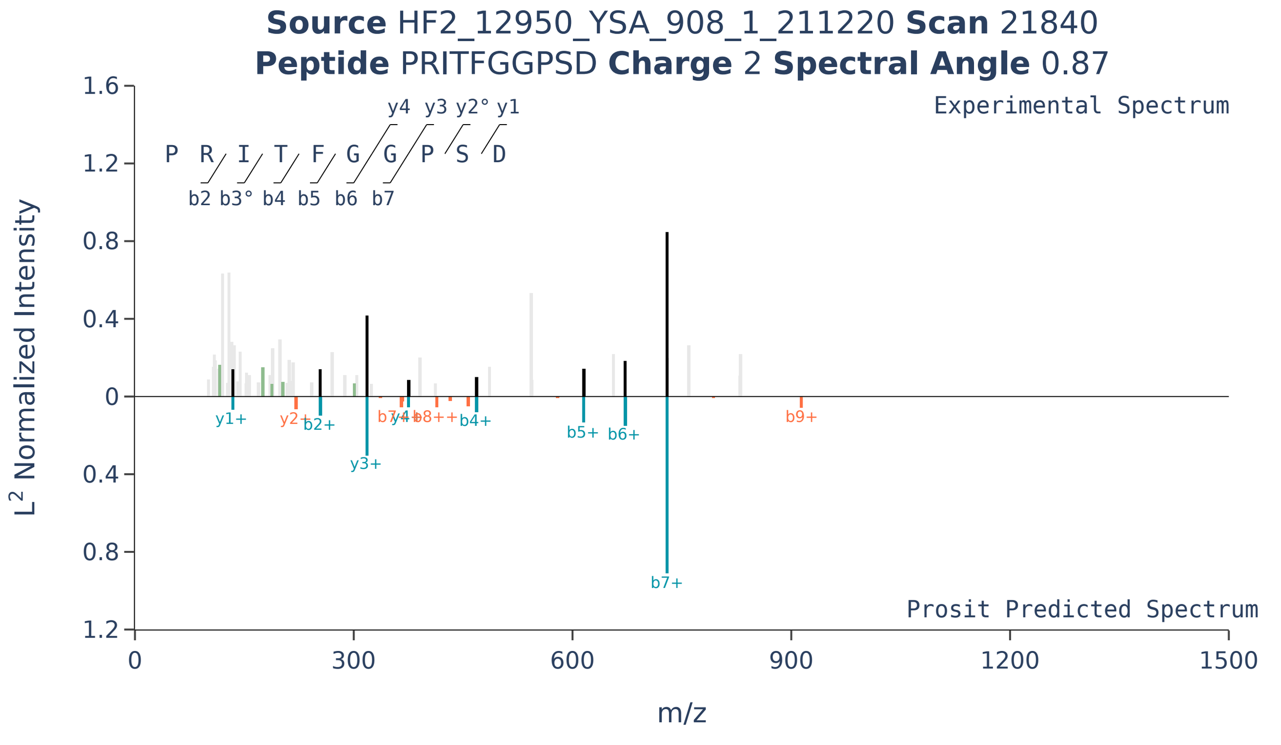
Source HF2_12950_YSA_908_1_211220 Scan 21840
Peptide PRITFGGPSD Charge 2 Spectral Angle 0.87
Experimental Spectrum
Prosit Predicted Spectrum
L² Normalized Intensity
m/z

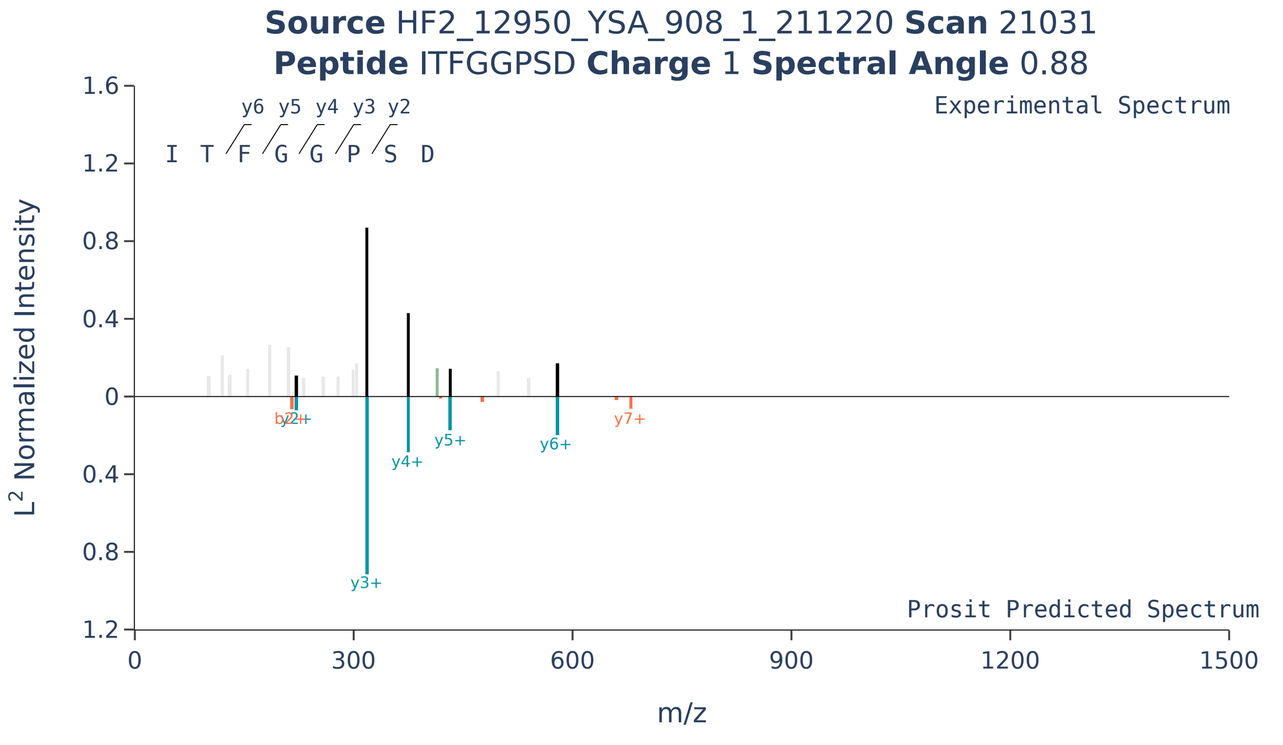
Source HF2_12950_YSA_908_1_211220 Scan 21031
Peptide ITFGGPSD Charge 1 Spectral Angle 0.88
Experimental Spectrum
Prosit Predicted Spectrum
L² Normalized Intensity
m/z

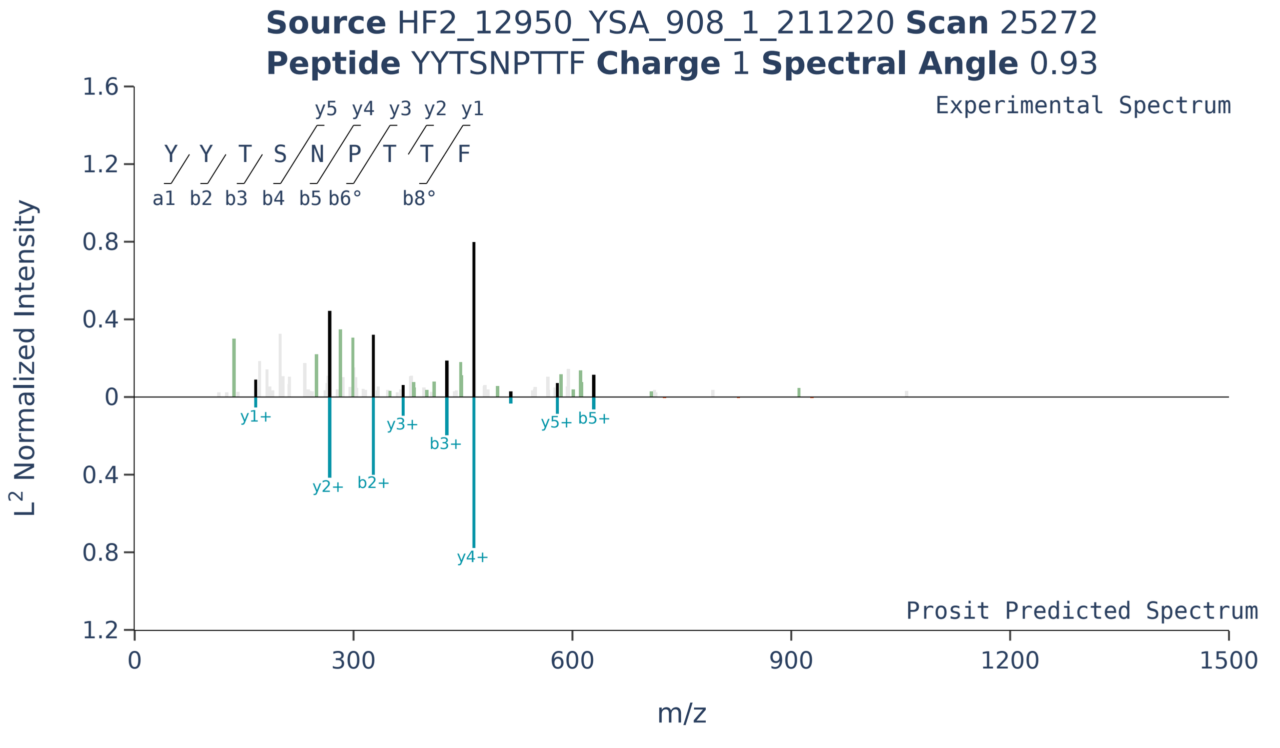
Source HF2_12950_YSA_908_1_211220 Scan 25272
Peptide YYTSNPTTF Charge 1 Spectral Angle 0.93
Experimental Spectrum
Prosit Predicted Spectrum
L² Normalized Intensity
m/z

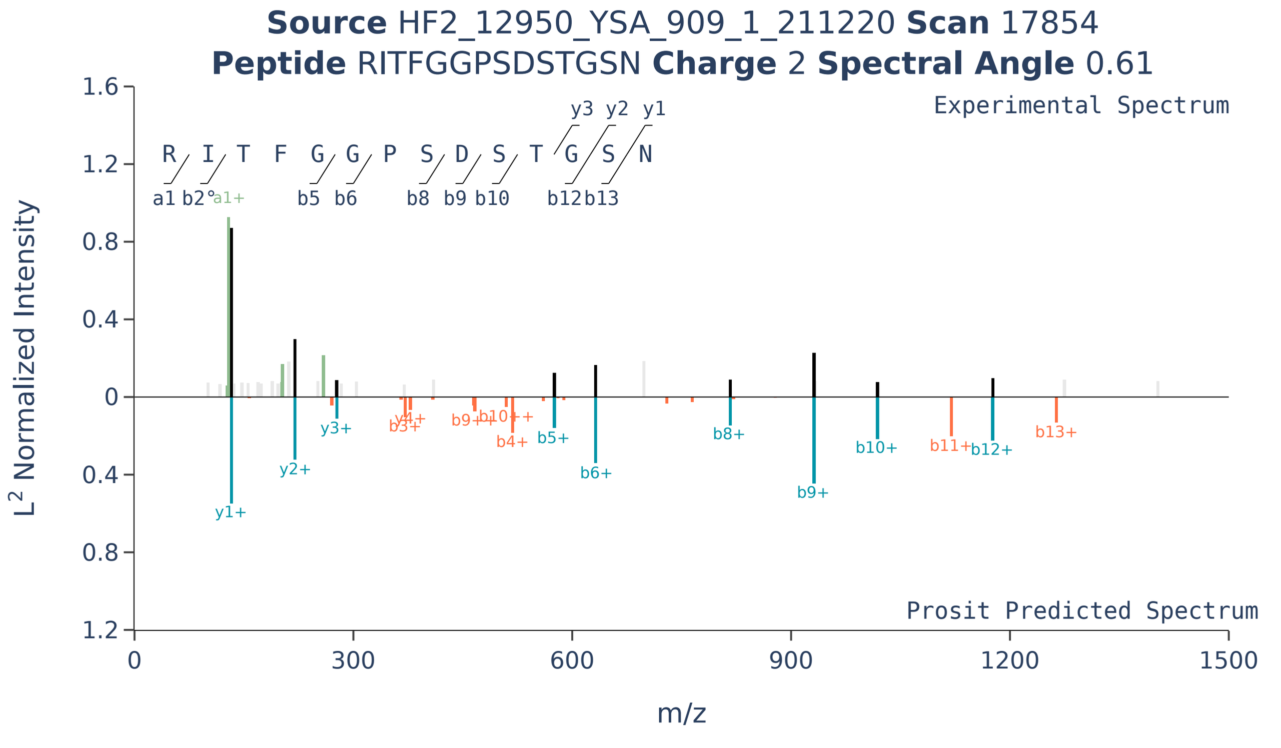
Source HF2_12950_YSA_909_1_211220 Scan 17854
Peptide RITFGGPSDSTGSN Charge 2 Spectral Angle 0.61
Experimental Spectrum
Prosit Predicted Spectrum
L² Normalized Intensity
m/z

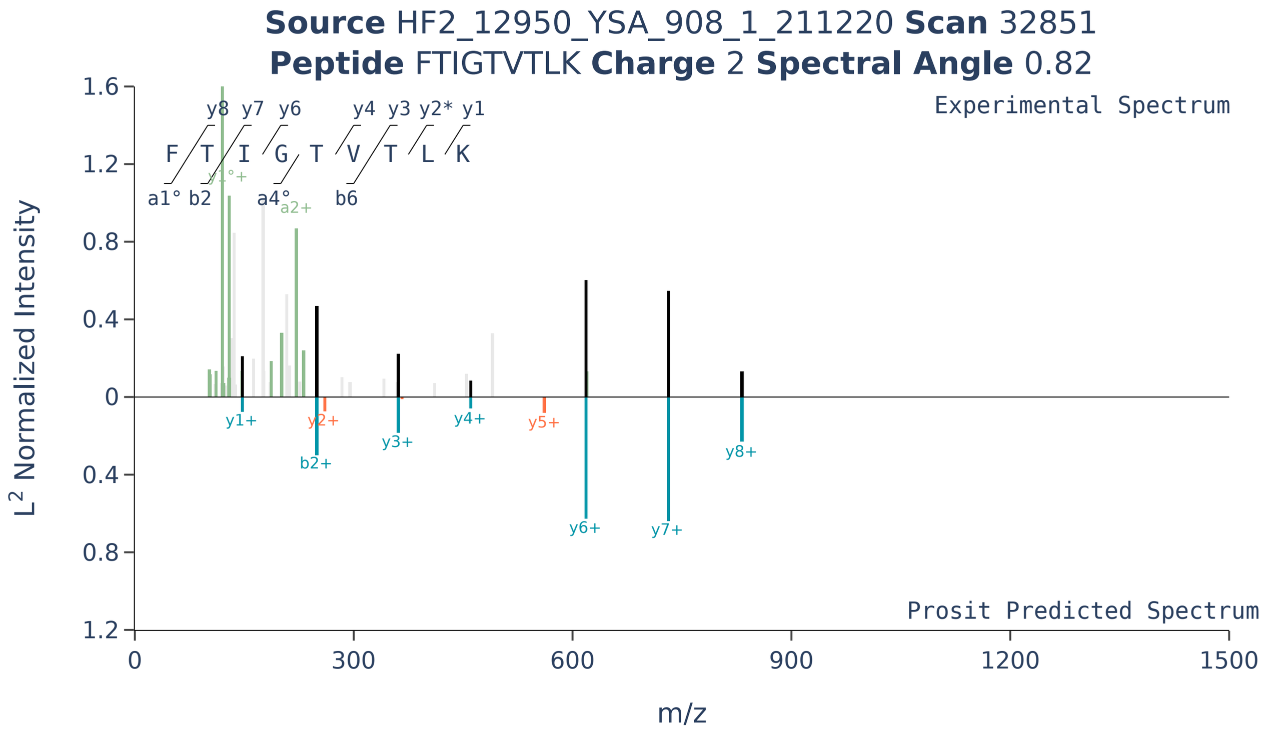
Source HF2_12950_YSA_908_1_211220 Scan 32851
Peptide FTIGTVTLK Charge 2 Spectral Angle 0.82
Experimental Spectrum
Prosit Predicted Spectrum
L² Normalized Intensity
m/z

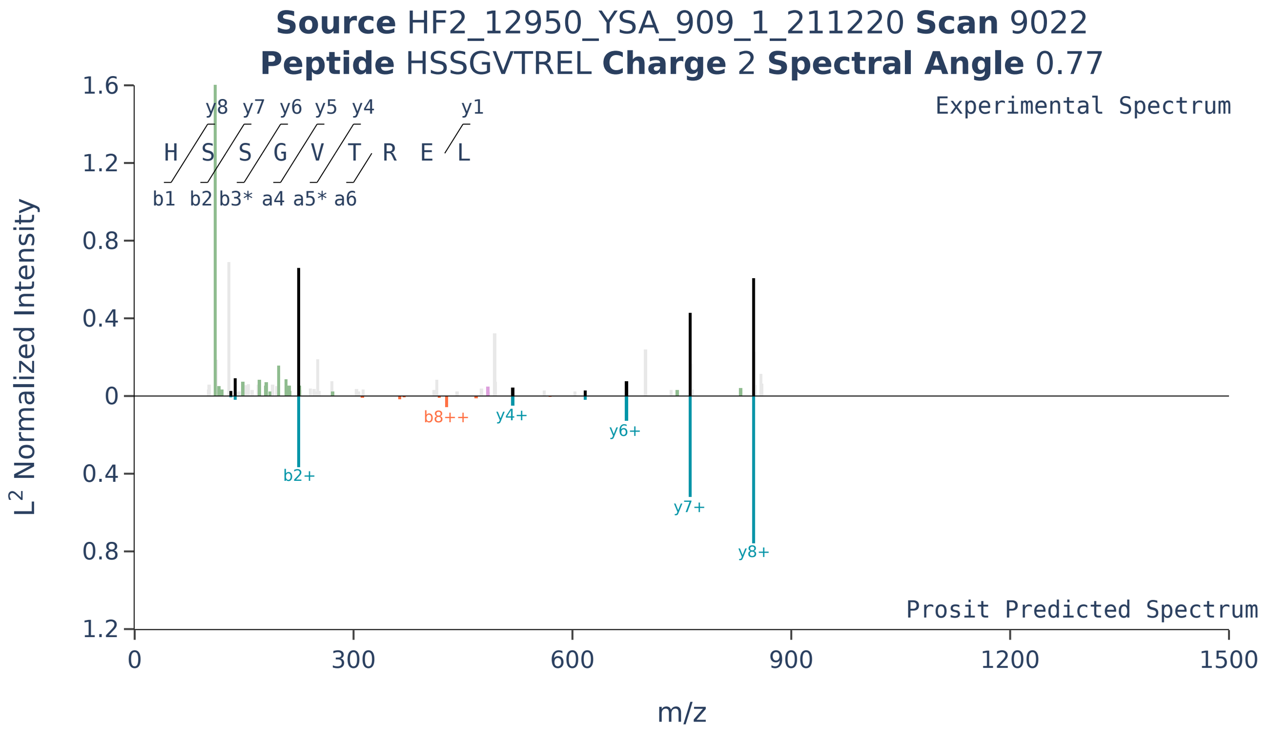
Source HF2_12950_YSA_909_1_211220 Scan 9022
Peptide HSSGVTREL Charge 2 Spectral Angle 0.77
Experimental Spectrum
Prosit Predicted Spectrum
L² Normalized Intensity
m/z

inSPIRE Spectral Plotting for cov-hek-1-240117

Experimental Spectrum Colour Code:

- Experimental peak matched to a Prosit predicted peak.
- Possible ion unknown to Prosit.
- Precursor matched peak.
- Experimental peak not matched to any potential ion.

Prosit Spectrum Colour Code:

- Prosit predicted peak matched to experimental spectrum.
- Prosit predicted peak not matched to experimental spectrum.

Additional Notes:

° indicates an ion with loss of H_2O.

* indicates an ion with loss of NH_3.

Source HF2_12950_YSA_904_1_211220 **Scan** 17169
Peptide RITFGGPSDSTGSN **Charge** 2 **Spectral Angle** 0.68

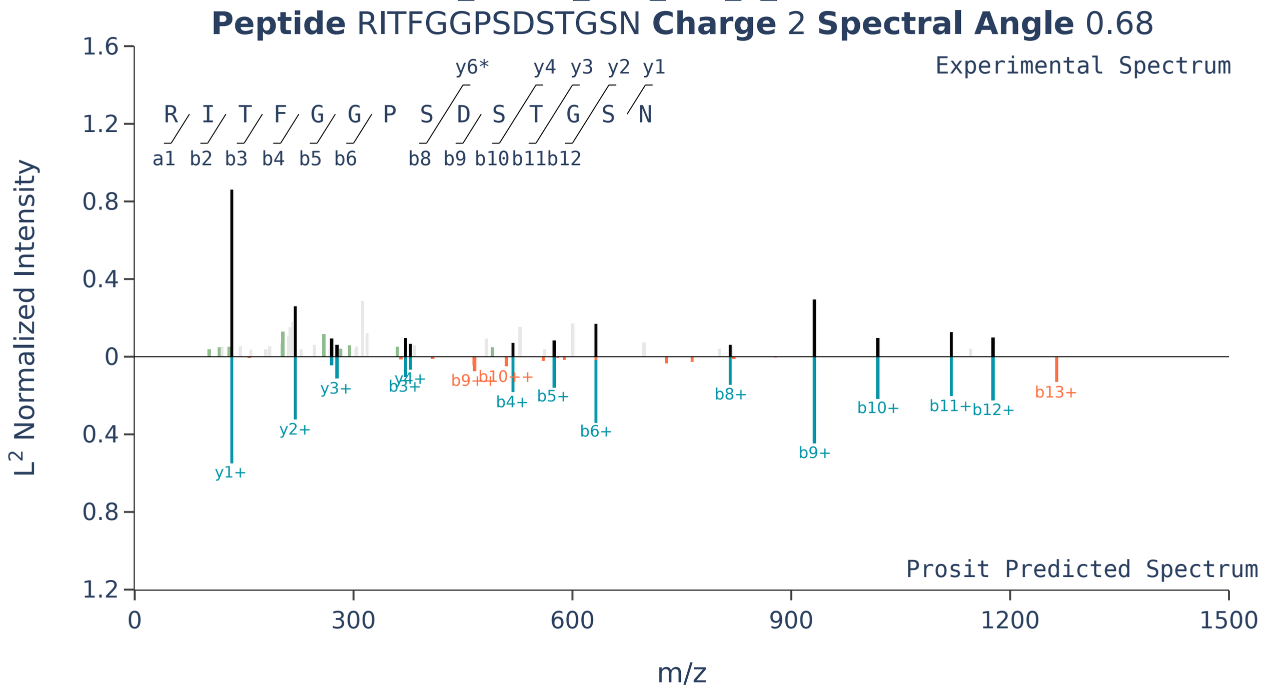

Source HF2_12950_YSA_904_1_211220 **Scan** 23387
Peptide APRITFGGP **Charge** 2 **Spectral Angle** 0.78

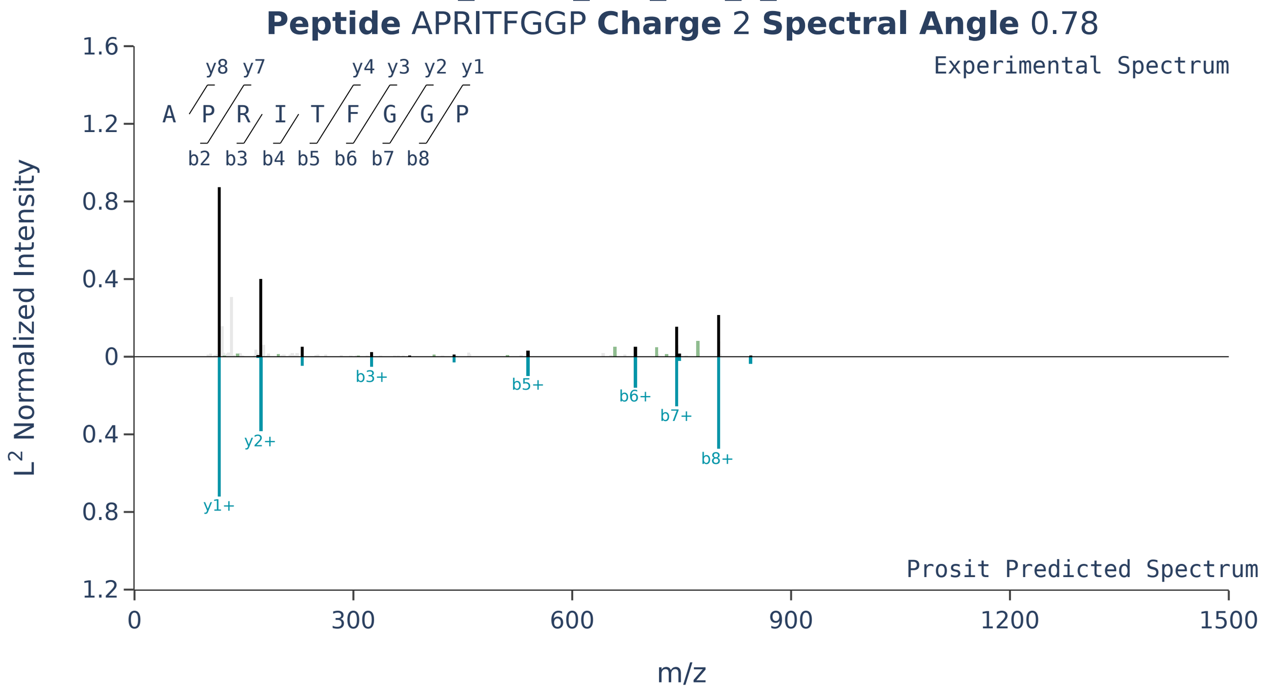

Source HF2_12950_YSA_904_1_211220 **Scan** 27798
Peptide AADLDDFSKQLQ **Charge** 2 **Spectral Angle** 0.87

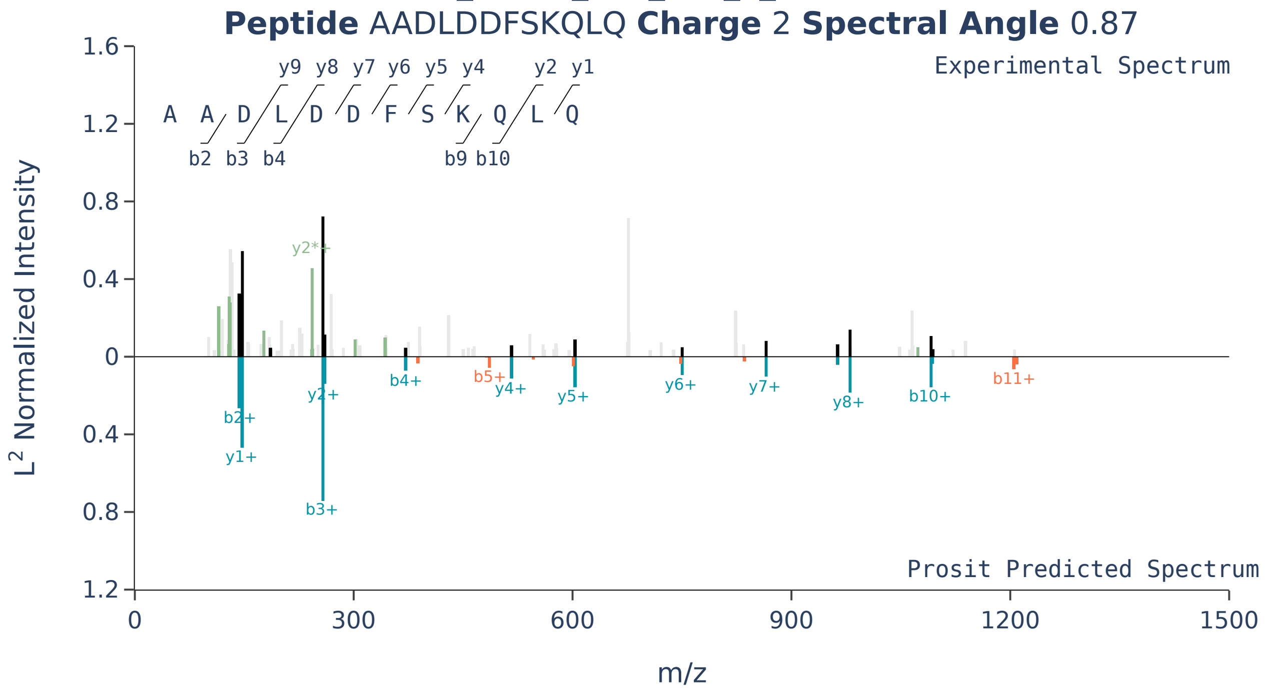

Source HF2_12950_YSA_904_1_211220 **Scan** 17638
Peptide RITFGGPSD **Charge** 2 **Spectral Angle** 0.95

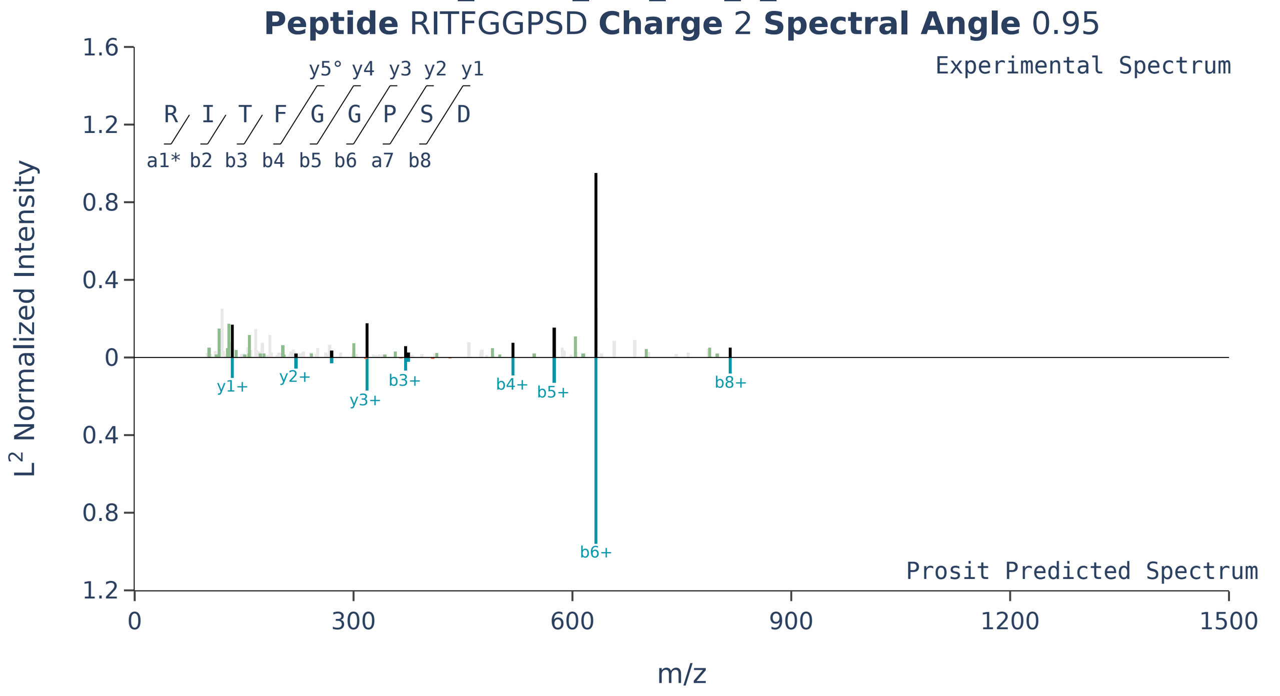

Source HF2_12950_YSA_904_1_211220 **Scan** 30775
Peptide GPMVLRGLIT **Charge** 2 **Spectral Angle** 0.72

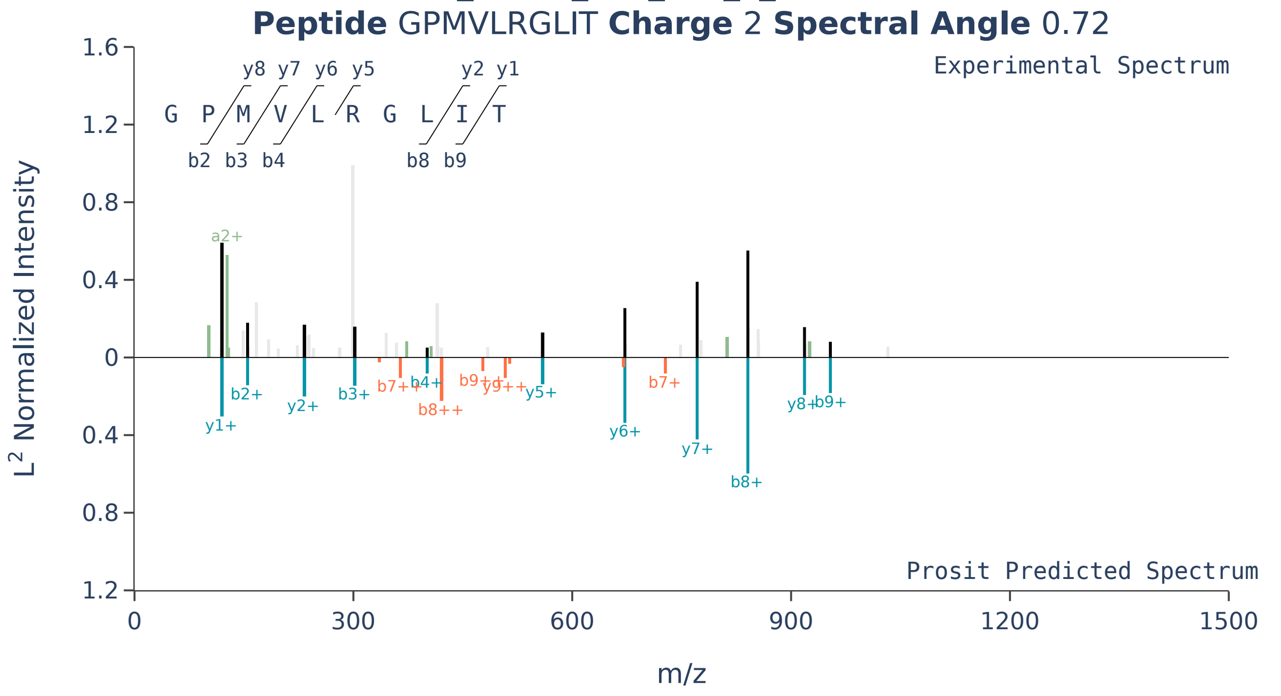

Source HF2_12950_YSA_904_1_211220 **Scan** 23034
Peptide NAPRITFGGPSD **Charge** 2 **Spectral Angle** 0.86

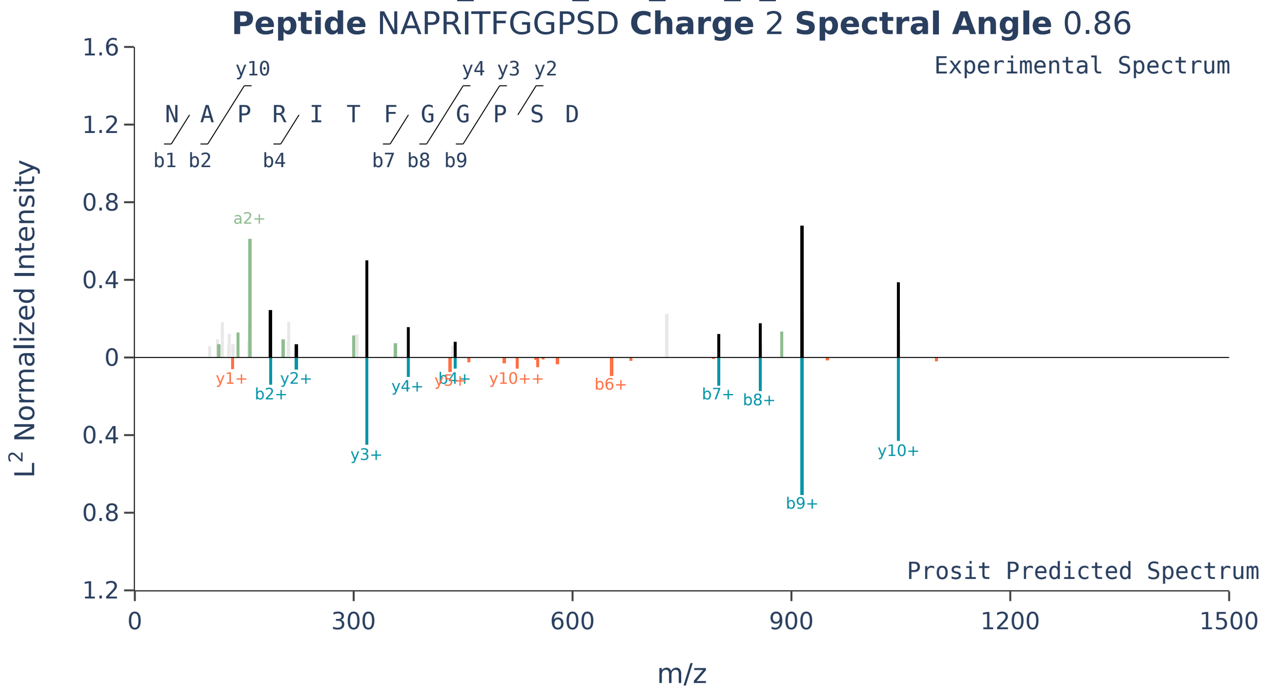

Source HF2_12950_YSA_904_1_211220 **Scan** 21044
Peptide PRITFGGPSD **Charge** 2 **Spectral Angle** 0.9

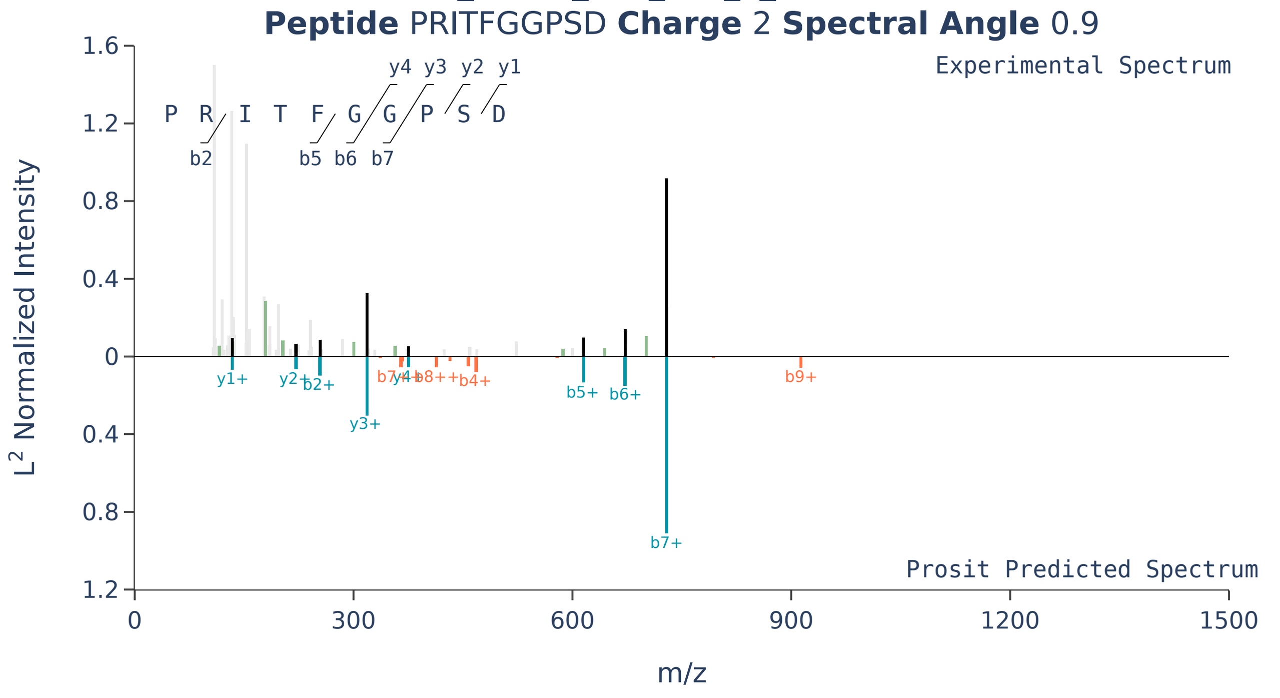

Source HF2_12950_YSA_904_1_211220 **Scan** 19236
Peptide RITFGGP **Charge** 2 **Spectral Angle** 0.85

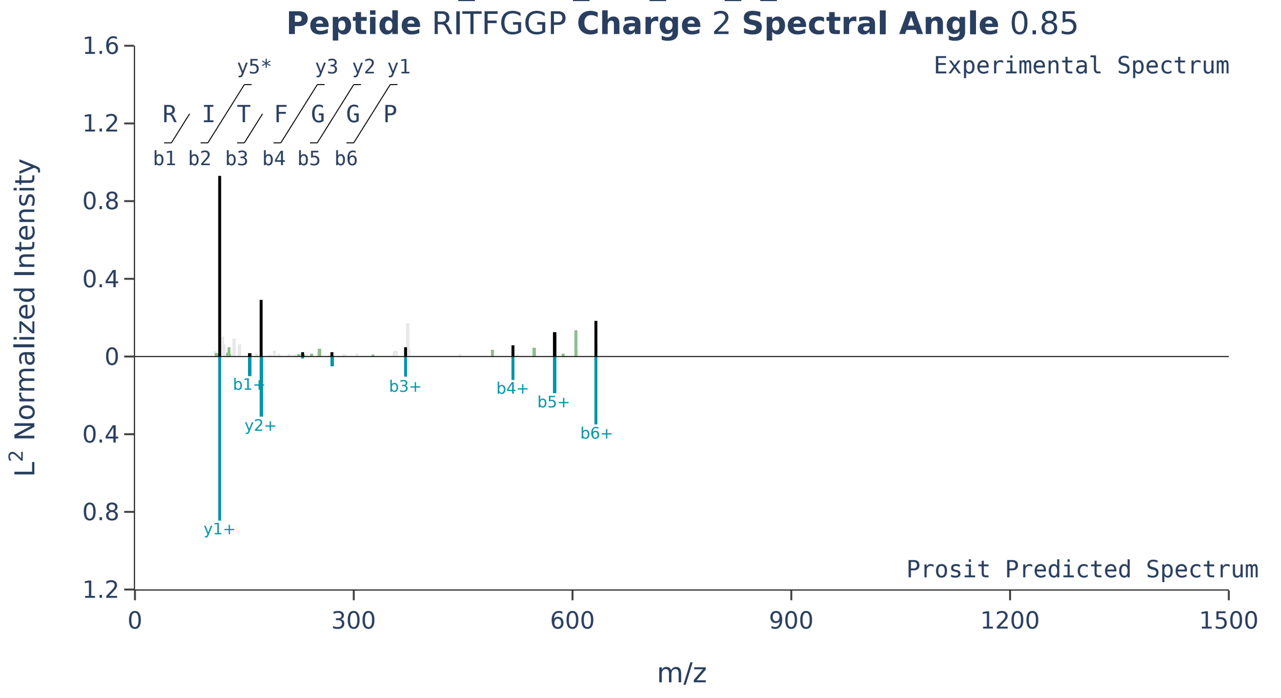

Source HF2_12950_YSA_904_1_211220 **Scan** 20233
Peptide ITFGGPSD **Charge** 1 **Spectral Angle** 0.92

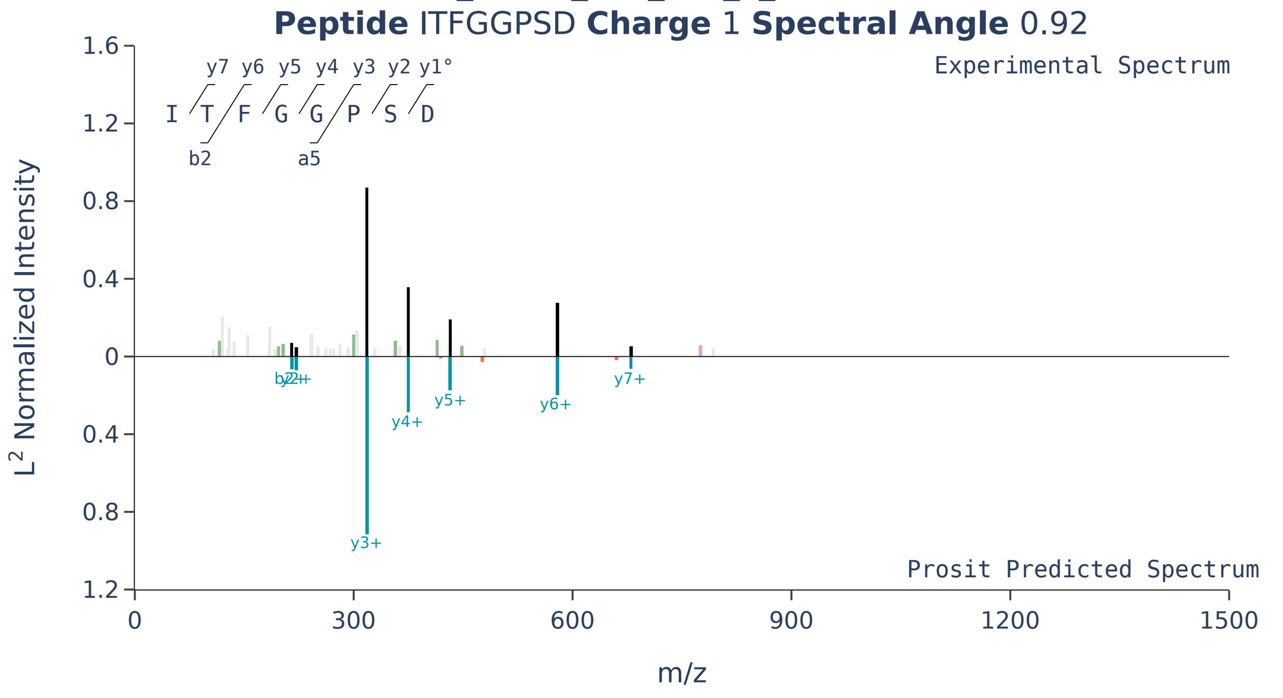

Source HF2_12950_YSA_904_1_211220 **Scan** 22299
Peptide APRITFGGPSD **Charge** 2 **Spectral Angle** 0.89

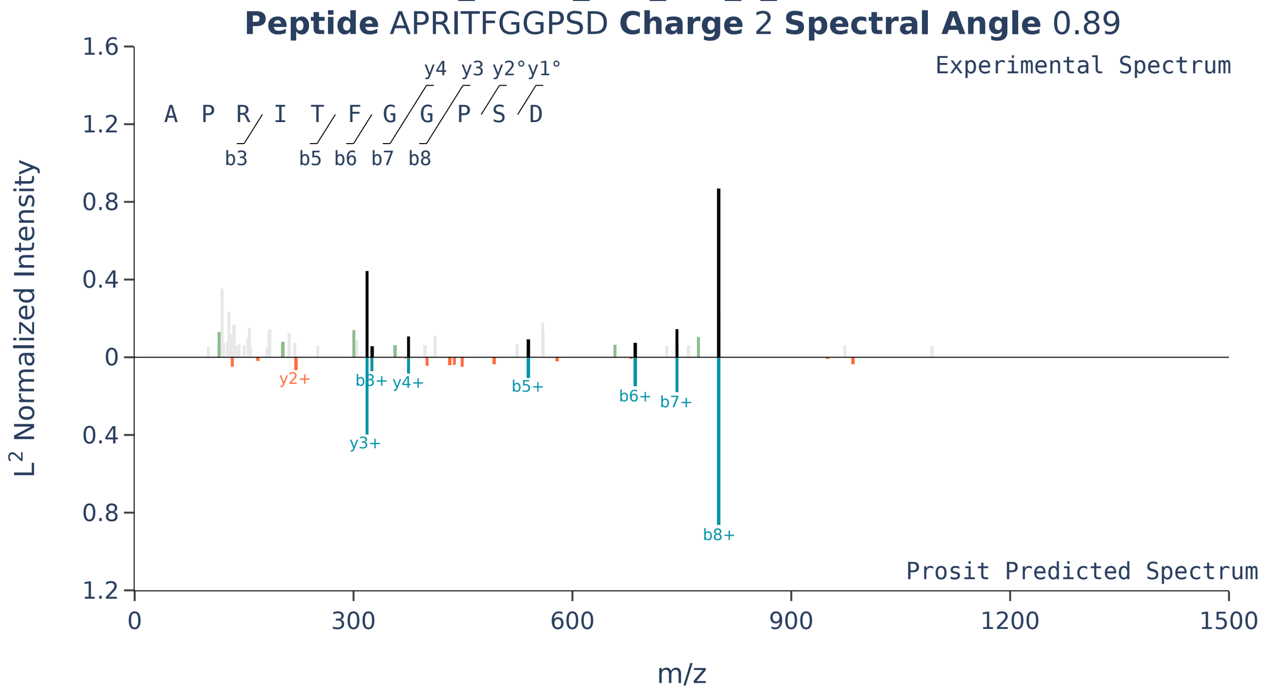

Source HF2_12950_YSA_904_1_211220 **Scan** 22936
Peptide NNELSPVALR **Charge** 2 **Spectral Angle** 0.82

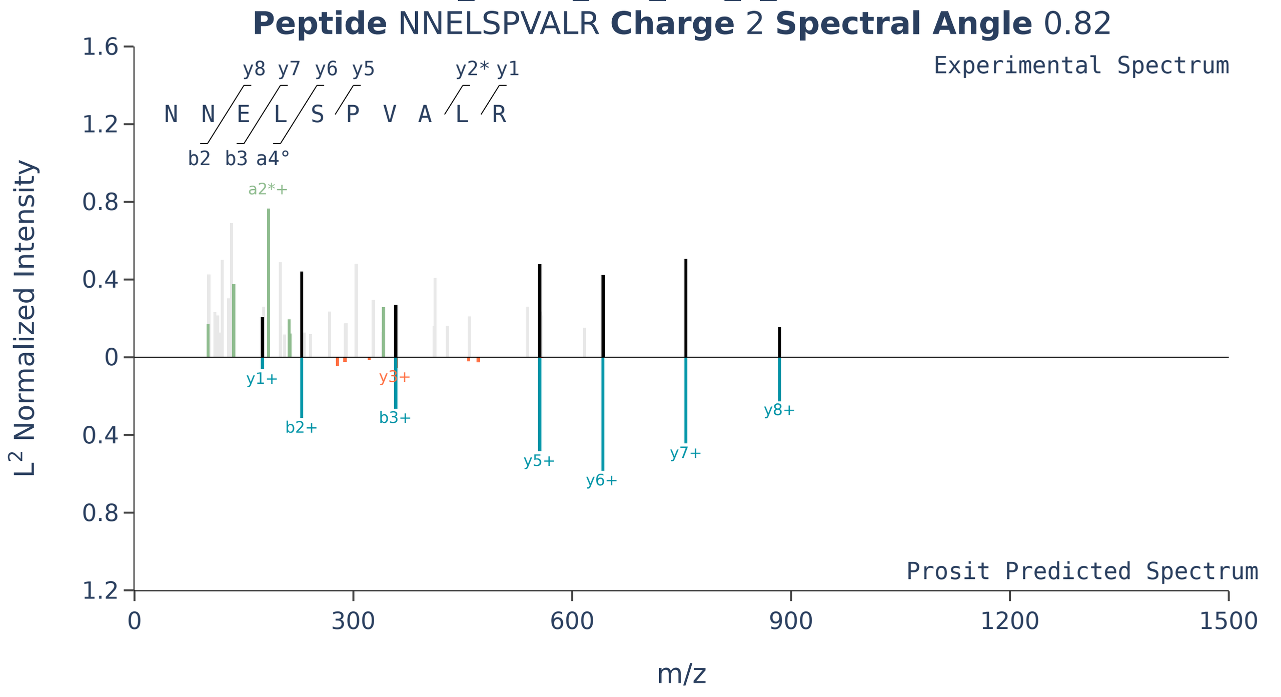

Source HF2_12950_YSA_904_1_211220 **Scan** 24377
Peptide NAPRITFGGP **Charge** 2 **Spectral Angle** 0.59

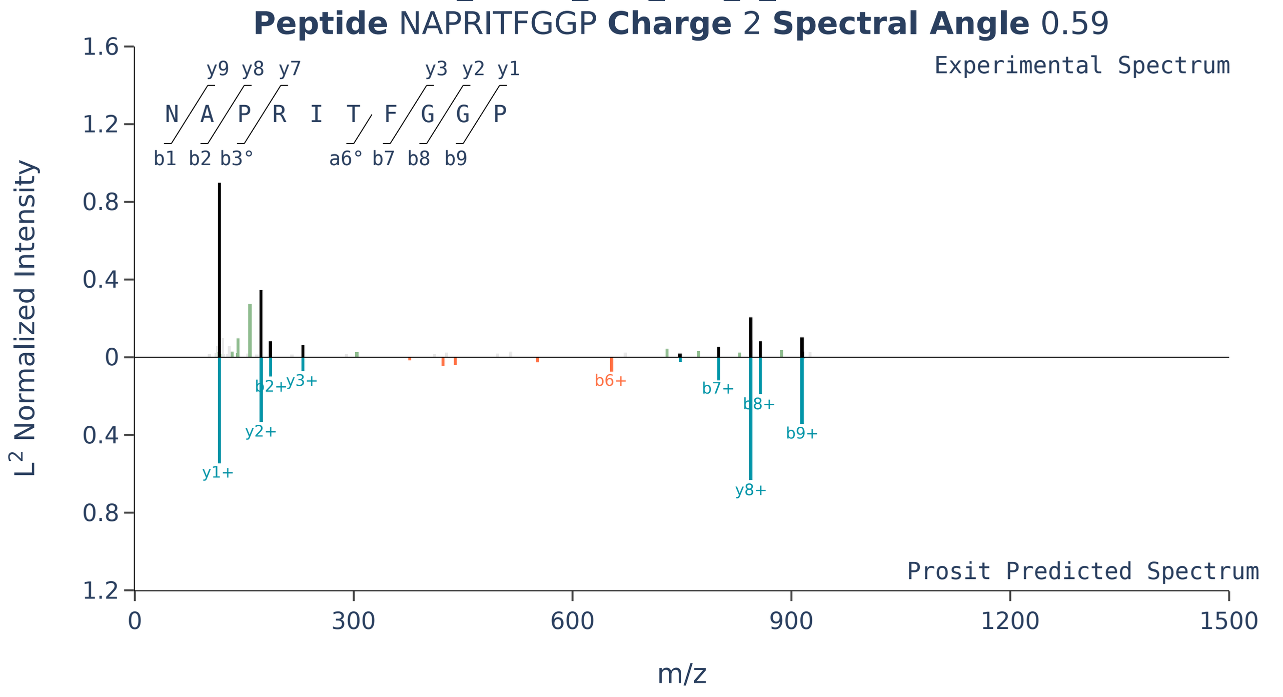

Source HF2_12950_YSA_904_1_211220 **Scan** 22325
Peptide PRITFGGP **Charge** 2 **Spectral Angle** 0.8

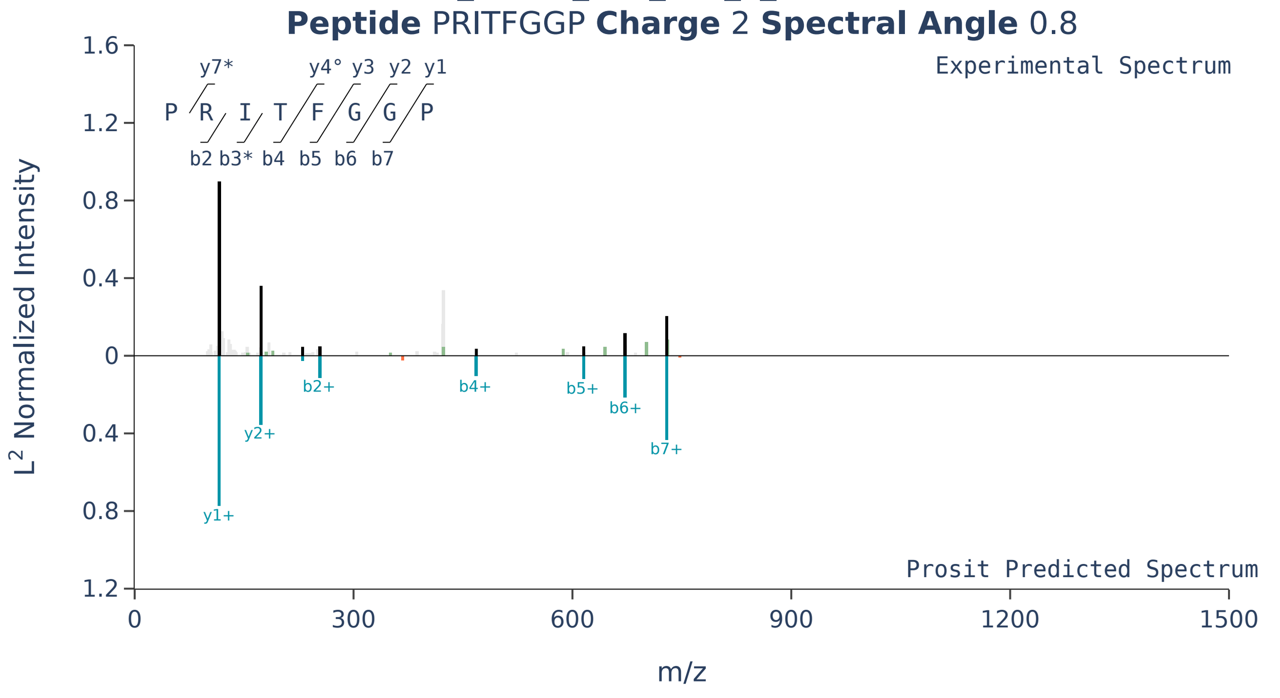

Source HF2_12950_YSA_904_1_211220 **Scan** 19506
Peptide ITFGGPSDSTGSN **Charge** 2 **Spectral Angle** 0.88

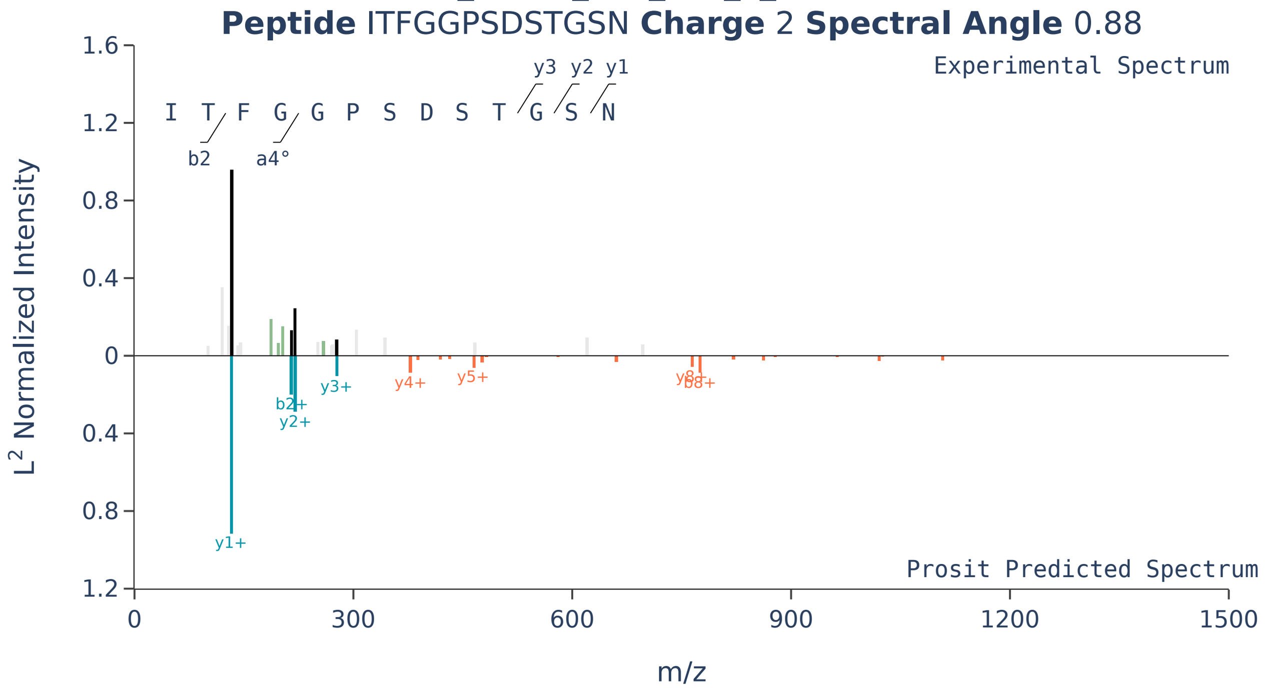

inSPIRE Spectral Plotting for cov-hek-2-240117

Experimental Spectrum Colour Code:

- Experimental peak matched to a Prosit predicted peak.
- Possible ion unknown to Prosit.
- Precursor matched peak.
- Experimental peak not matched to any potential ion.

Prosit Spectrum Colour Code:

- Prosit predicted peak matched to experimental spectrum.
- Prosit predicted peak not matched to experimental spectrum.

Additional Notes:

° indicates an ion with loss of H_2O.

* indicates an ion with loss of NH_3.

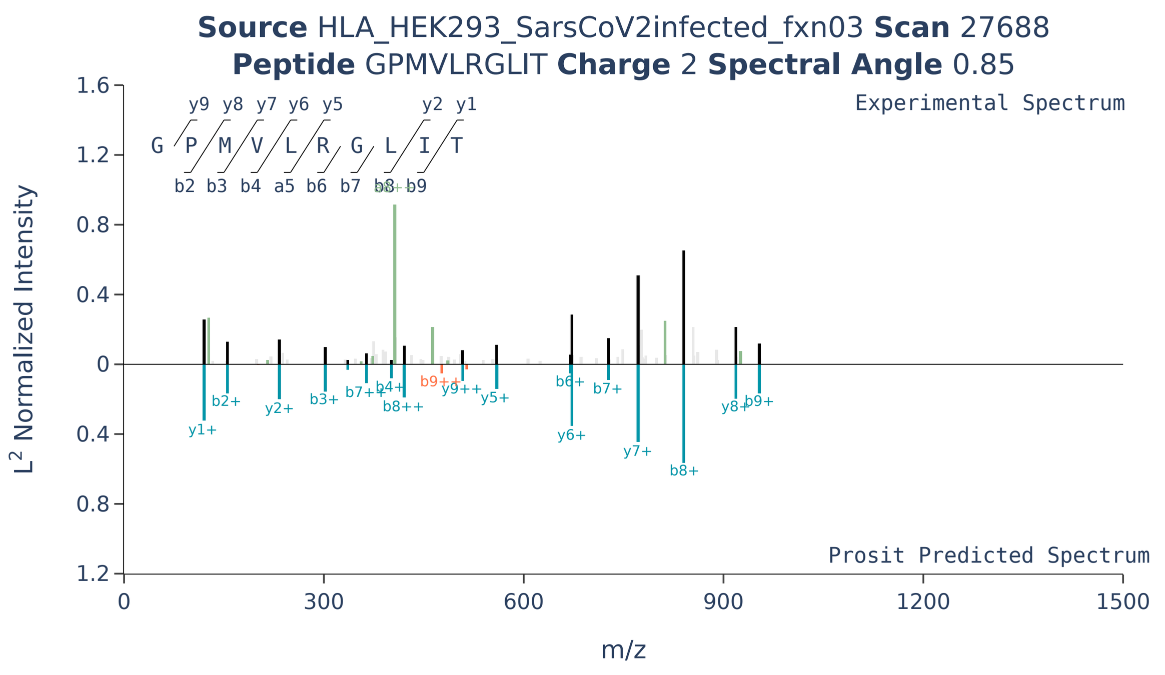
Source HLA_HEK293_SarsCoV2infected_fxn03 Scan 27688
Peptide GPMVLRGLIT Charge 2 Spectral Angle 0.85
Experimental Spectrum
Prosit Predicted Spectrum
L² Normalized Intensity
m/z

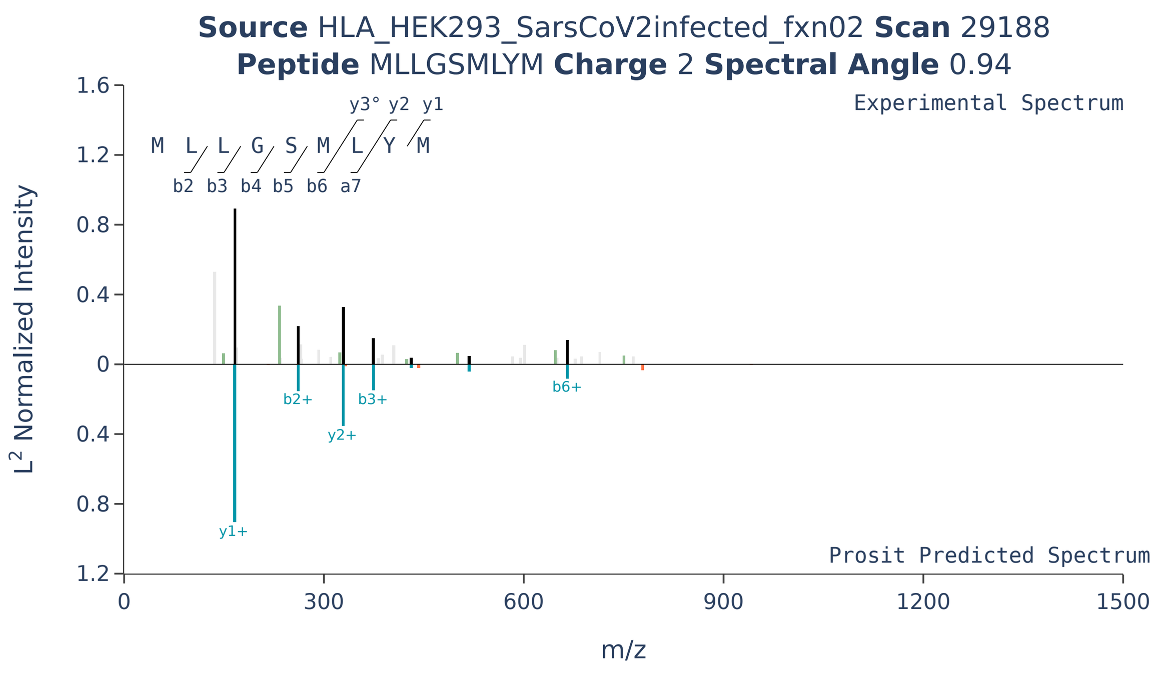
Source HLA_HEK293_SarsCoV2infected_fxn02 Scan 29188
Peptide MLLGSMLYM Charge 2 Spectral Angle 0.94
Experimental Spectrum
Prosit Predicted Spectrum
L² Normalized Intensity
m/z

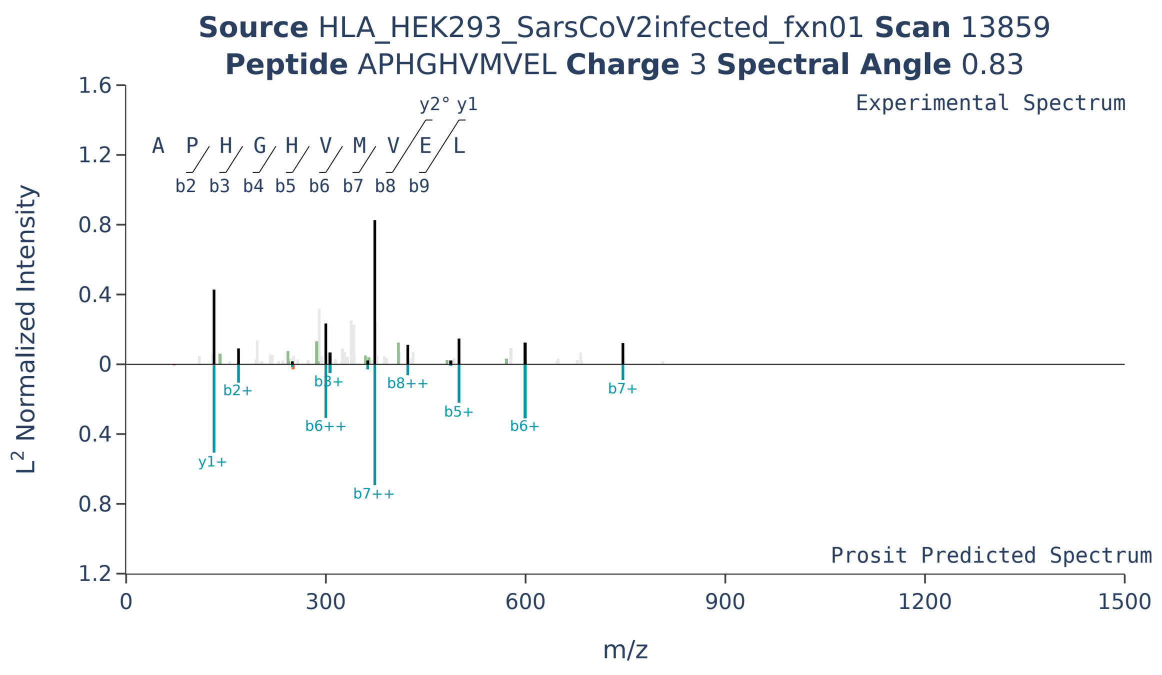
Source HLA_HEK293_SarsCoV2infected_fxn01 Scan 13859
Peptide APHGHVMVEL Charge 3 Spectral Angle 0.83
Experimental Spectrum
Prosit Predicted Spectrum
L² Normalized Intensity
m/z

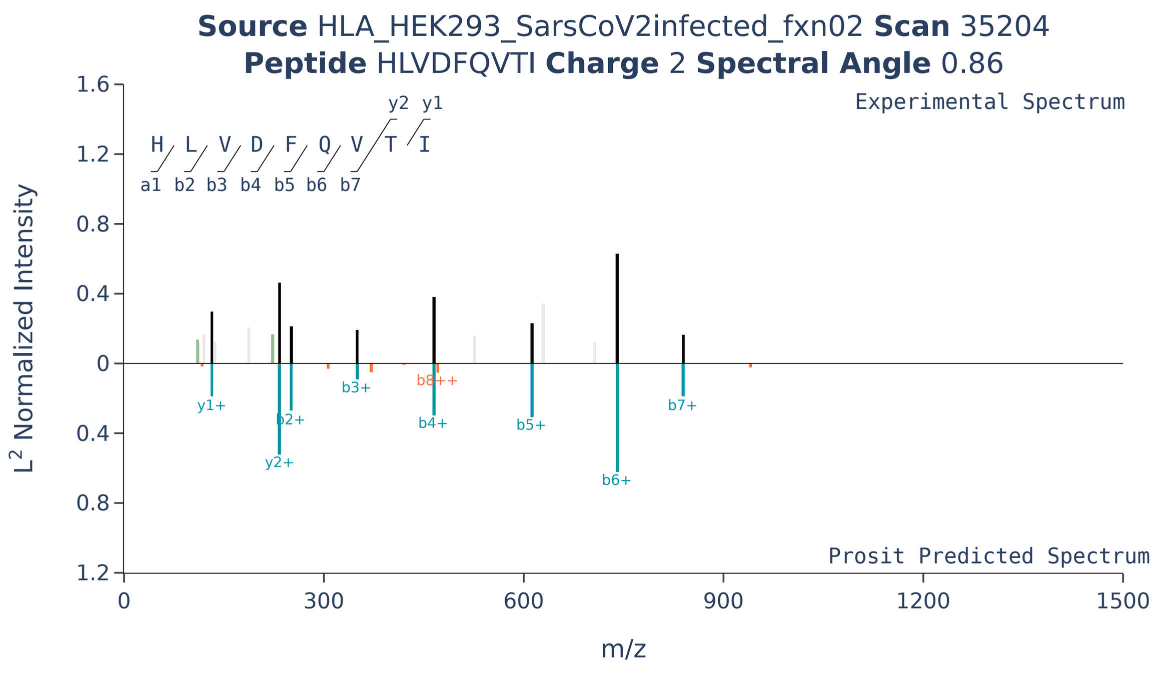
Source HLA_HEK293_SarsCoV2infected_fxn02 Scan 35204
Peptide HLVDFQVTI Charge 2 Spectral Angle 0.86
Experimental Spectrum
Prosit Predicted Spectrum
L² Normalized Intensity
m/z

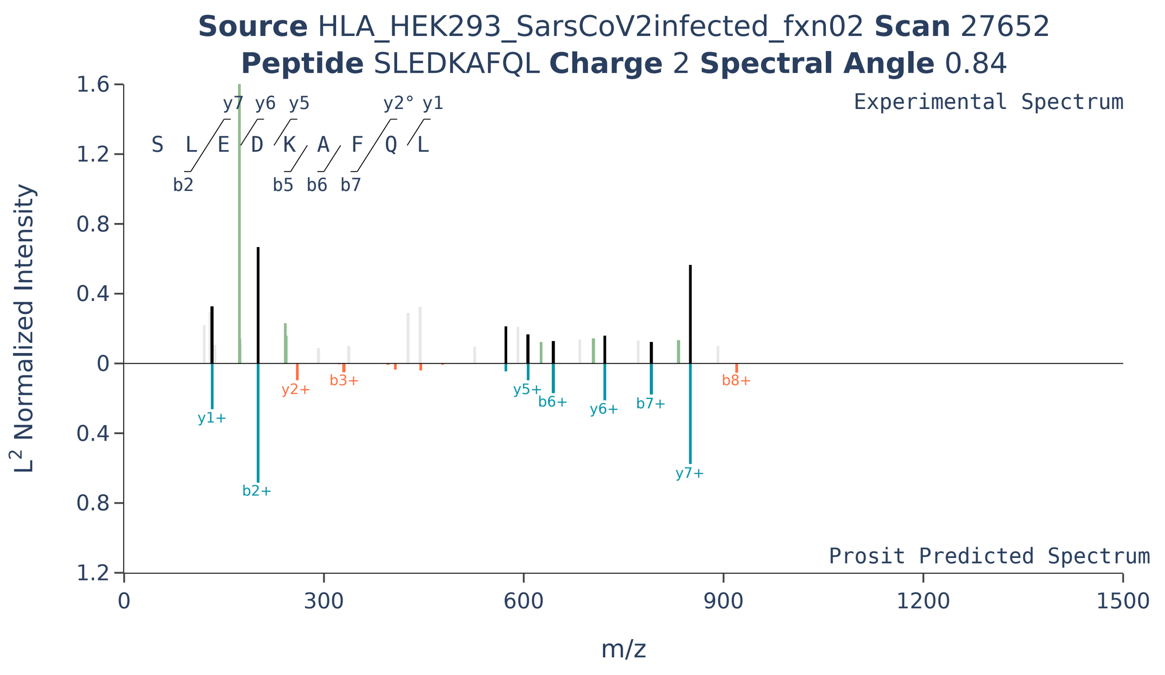
Source HLA_HEK293_SarsCoV2infected_fxn02 Scan 27652
Peptide SLEDKAFQL Charge 2 Spectral Angle 0.84
Experimental Spectrum
Prosit Predicted Spectrum
L² Normalized Intensity
m/z

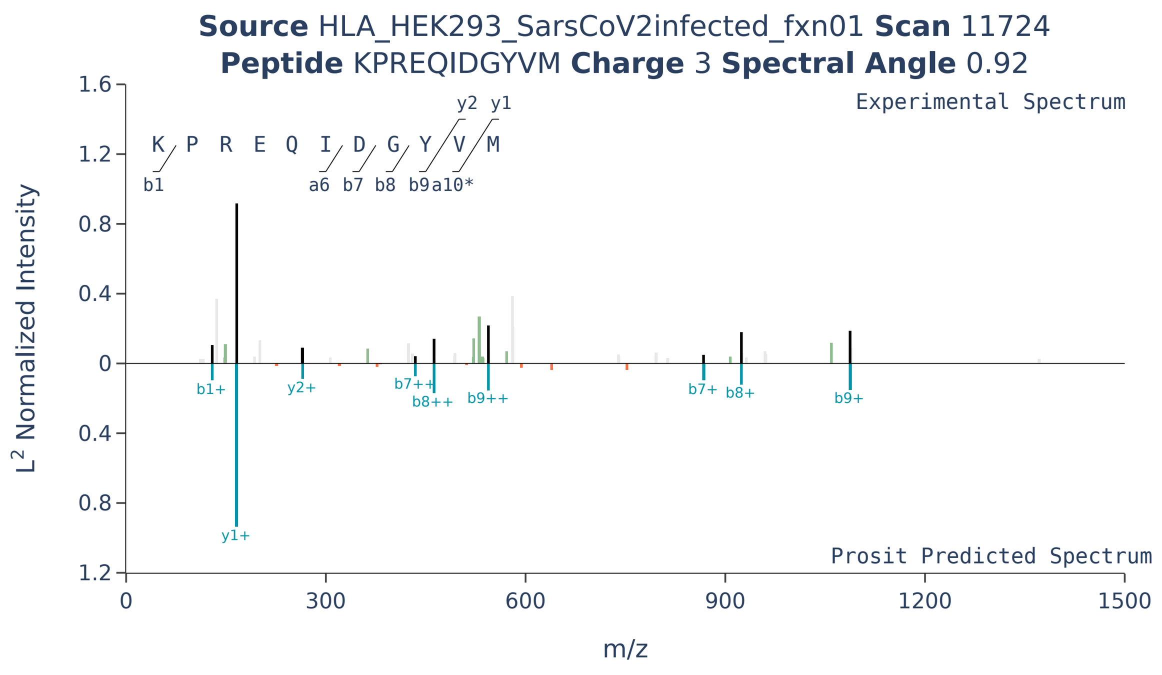
Source HLA_HEK293_SarsCoV2infected_fxn01 Scan 11724
Peptide KPREQIDGYVM Charge 3 Spectral Angle 0.92
Experimental Spectrum
Prosit Predicted Spectrum
L² Normalized Intensity
m/z

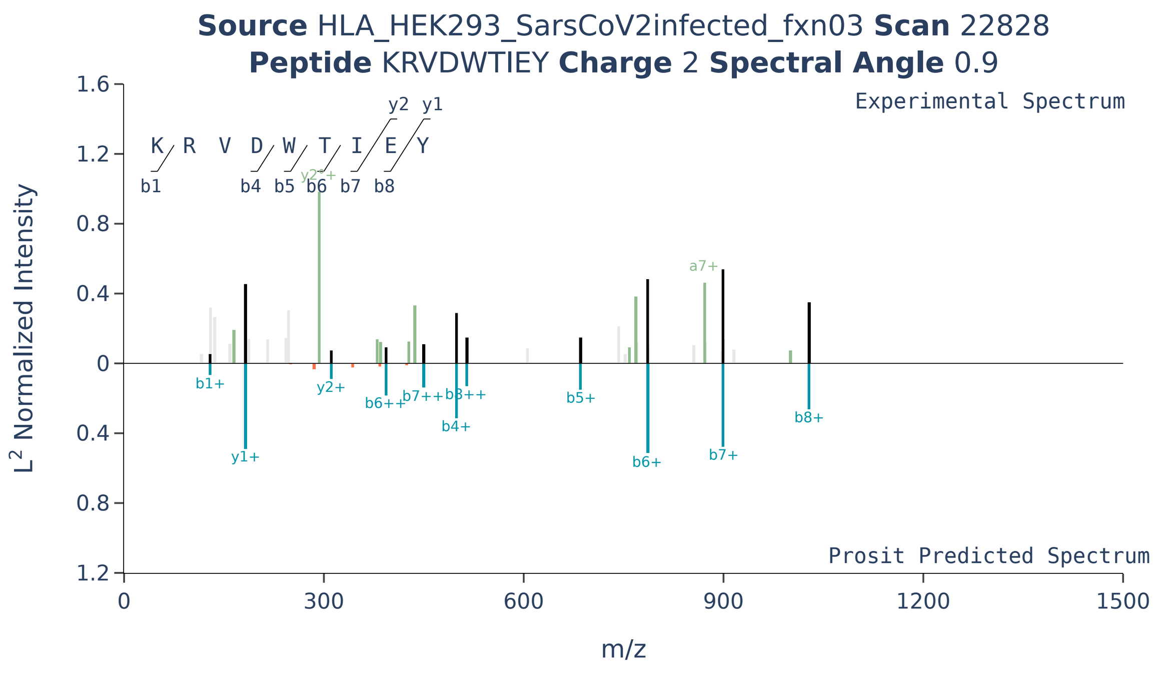
Source HLA_HEK293_SarsCoV2infected_fxn03 Scan 22828
Peptide KRVDWTIEY Charge 2 Spectral Angle 0.9
Experimental Spectrum
Prosit Predicted Spectrum
L² Normalized Intensity
m/z

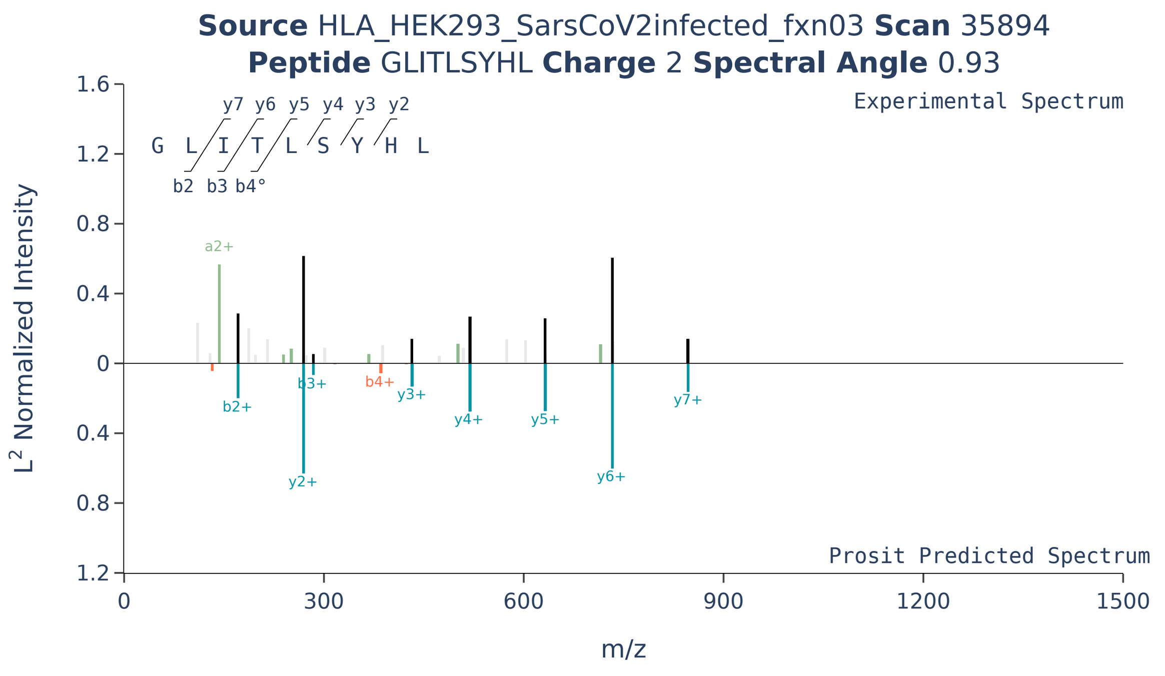
Source HLA_HEK293_SarsCoV2infected_fxn03 Scan 35894
Peptide GLITLSYHL Charge 2 Spectral Angle 0.93
Experimental Spectrum
Prosit Predicted Spectrum
L² Normalized Intensity
m/z

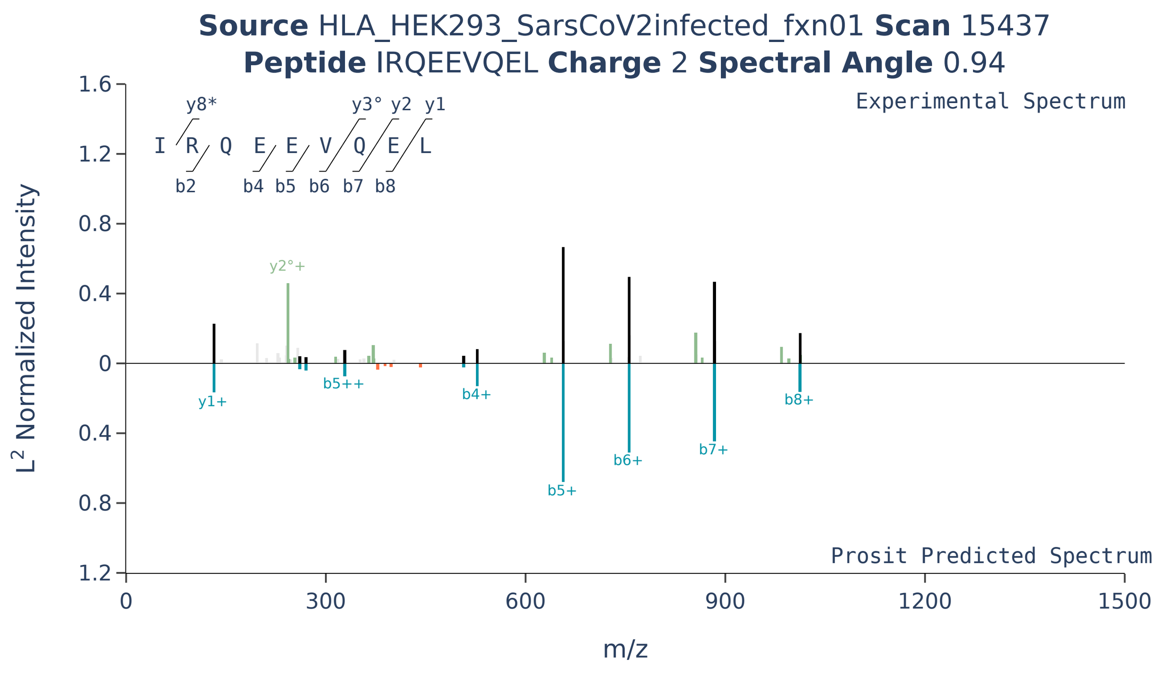
Source HLA_HEK293_SarsCoV2infected_fxn01 Scan 15437
Peptide IRQEEVQEL Charge 2 Spectral Angle 0.94
Experimental Spectrum
Prosit Predicted Spectrum
L² Normalized Intensity
m/z

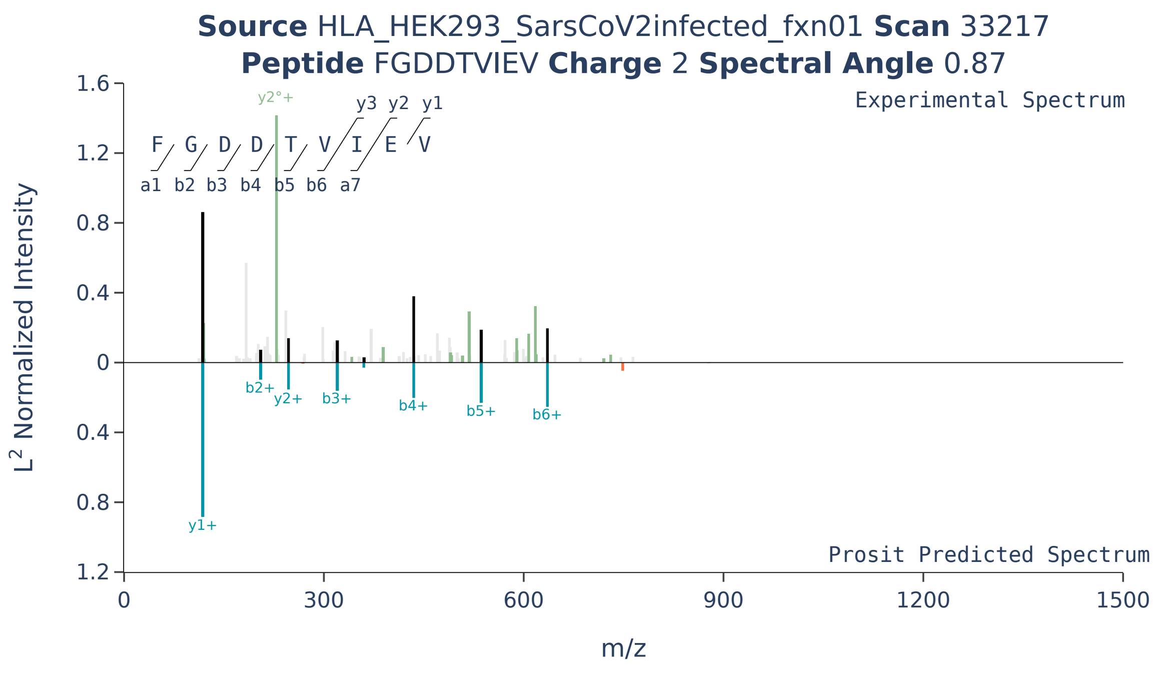
Source HLA_HEK293_SarsCoV2infected_fxn01 Scan 33217
Peptide FGDDTVIEV Charge 2 Spectral Angle 0.87
Experimental Spectrum
Prosit Predicted Spectrum
L² Normalized Intensity
m/z

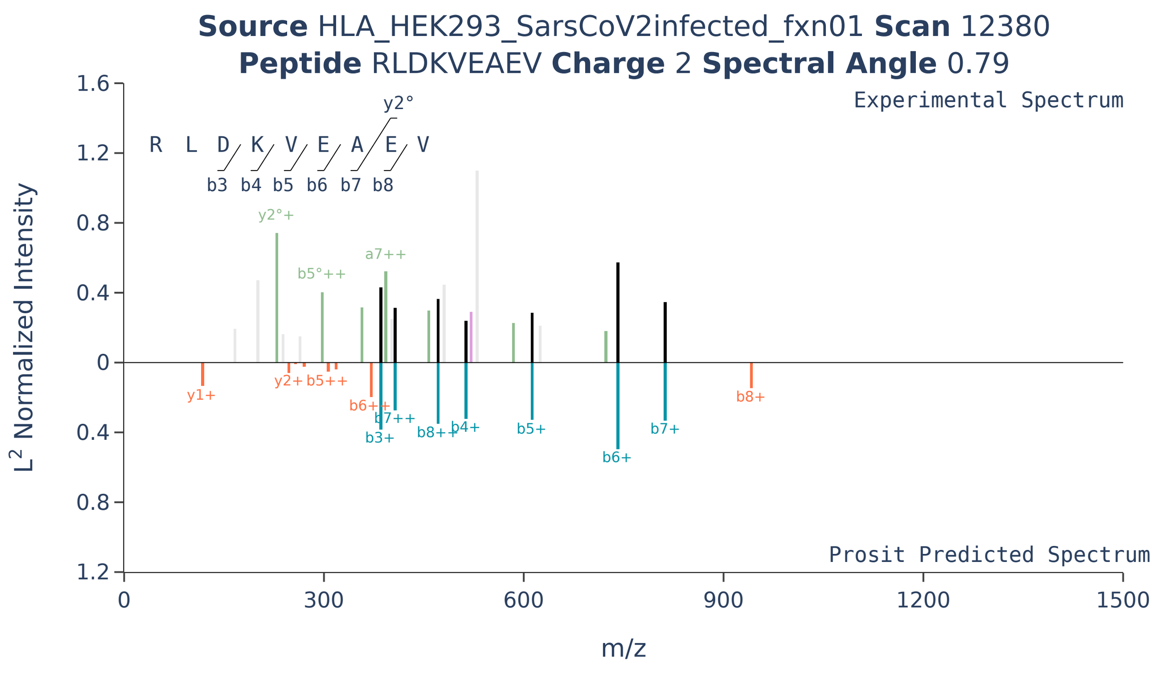
Source HLA_HEK293_SarsCoV2infected_fxn01 Scan 12380
Peptide RLDKVEAEV Charge 2 Spectral Angle 0.79
Experimental Spectrum
Prosit Predicted Spectrum
L² Normalized Intensity
m/z

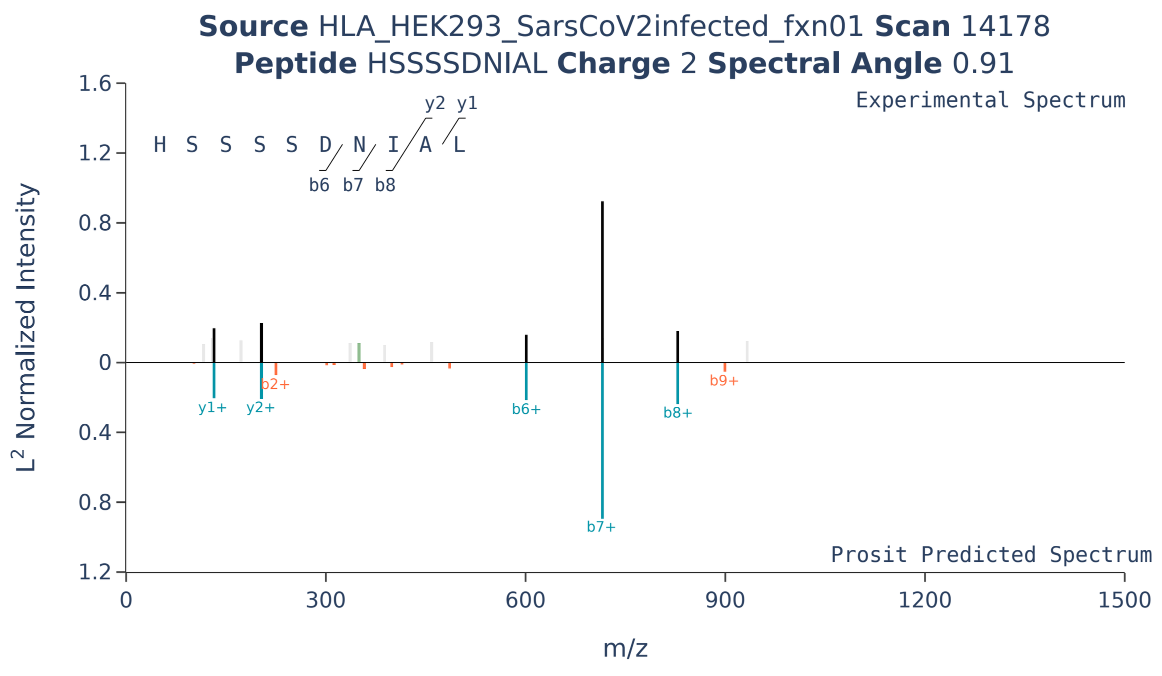
Source HLA_HEK293_SarsCoV2infected_fxn01 Scan 14178
Peptide HSSSSDNIAL Charge 2 Spectral Angle 0.91
Experimental Spectrum
Prosit Predicted Spectrum
L² Normalized Intensity
m/z

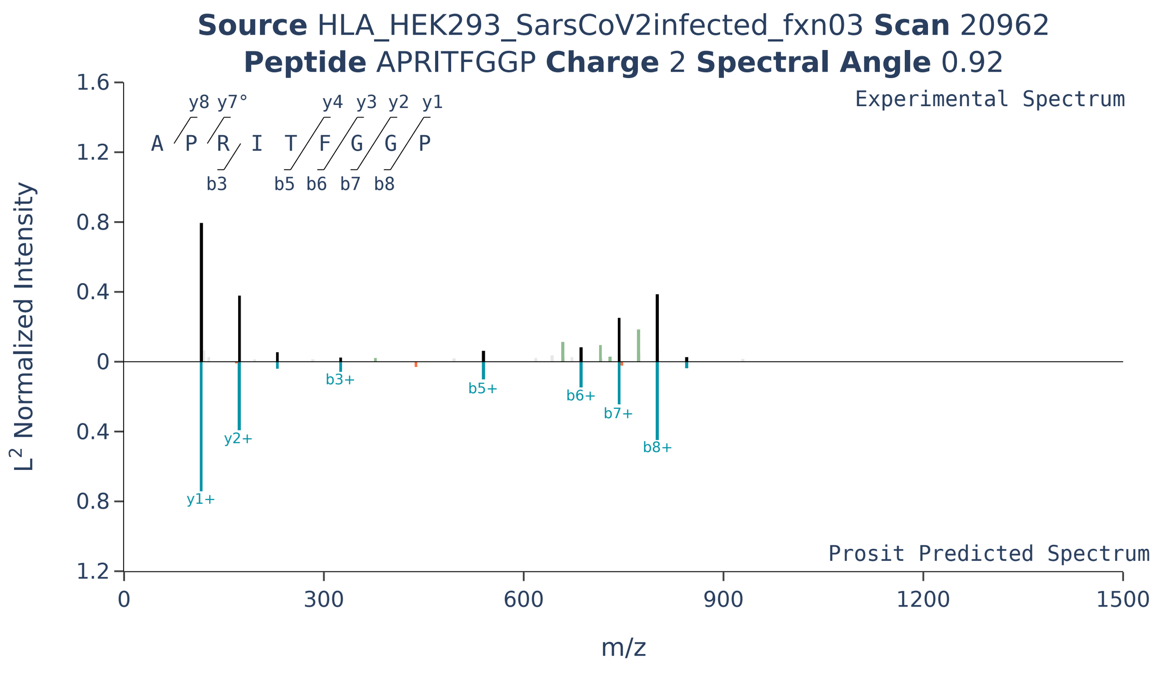
Source HLA_HEK293_SarsCoV2infected_fxn03 Scan 20962
Peptide APRITFGGP Charge 2 Spectral Angle 0.92
Experimental Spectrum
Prosit Predicted Spectrum
L² Normalized Intensity
m/z

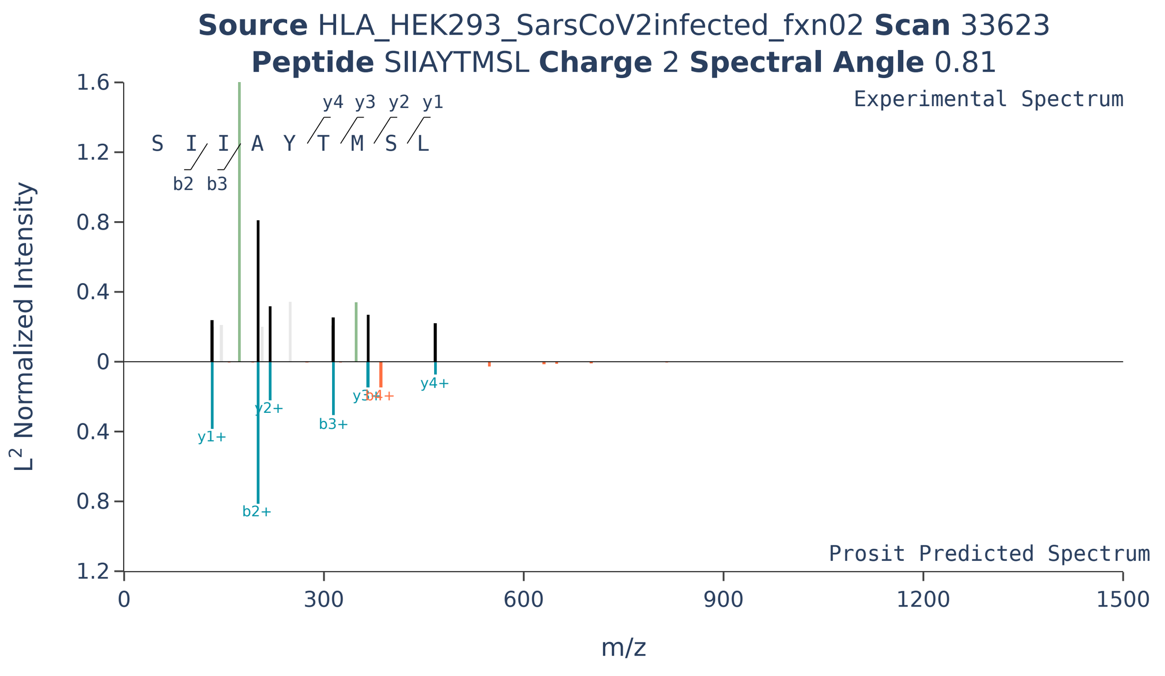
Source HLA_HEK293_SarsCoV2infected_fxn02 Scan 33623
Peptide SIIAYTMSL Charge 2 Spectral Angle 0.81
Experimental Spectrum
Prosit Predicted Spectrum
L² Normalized Intensity
m/z

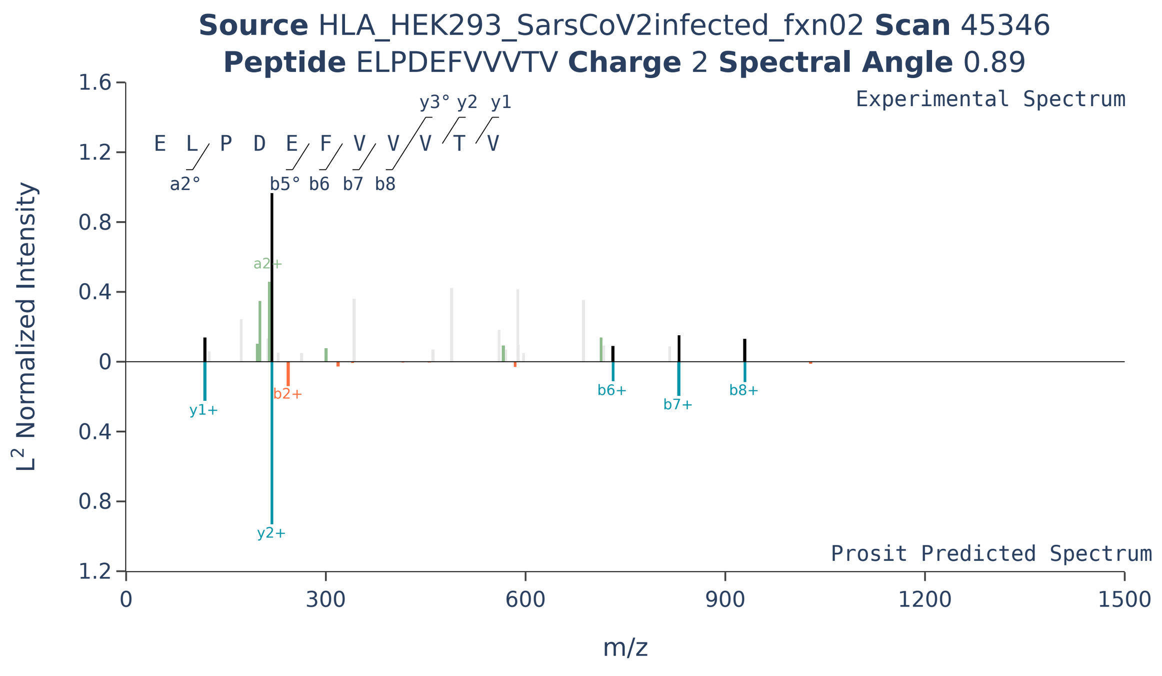
Source HLA_HEK293_SarsCoV2infected_fxn02 Scan 45346
Peptide ELPDEFVVVTV Charge 2 Spectral Angle 0.89
Experimental Spectrum
Prosit Predicted Spectrum
L² Normalized Intensity
m/z

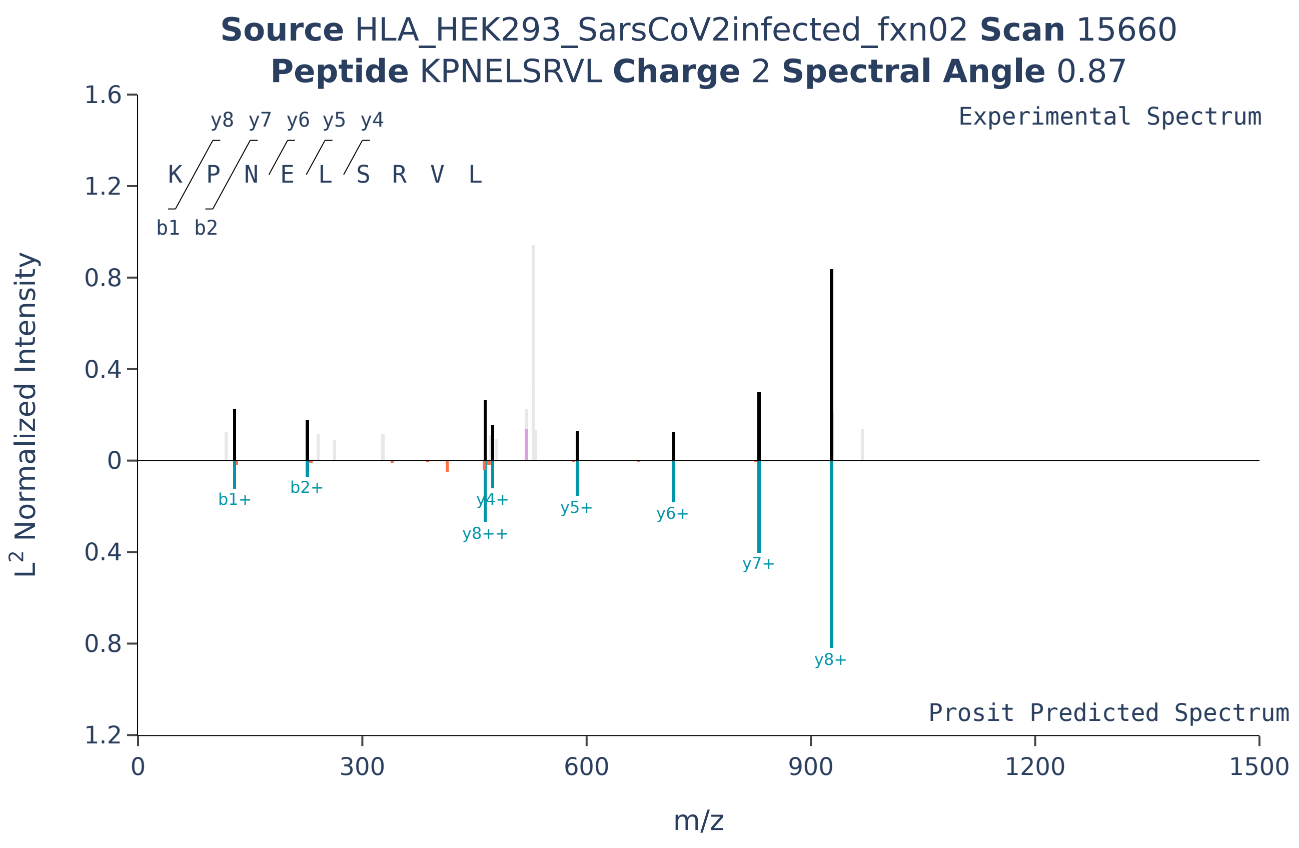
Source HLA_HEK293_SarsCoV2infected_fxn02 Scan 15660
Peptide KPNELSRVL Charge 2 Spectral Angle 0.87
Experimental Spectrum
Prosit Predicted Spectrum
y8 y7 y6 y5 y4
K P N E L S R V L
b1 b2
b1+
b2+
y4+
y8++
y5+
y6+
y7+
y8+
L2 Normalized Intensity
m/z
0
300
600
900
1200
1500
1.6
1.2
0.8
0.4
0
0.4
0.8
1.2

inSPIRE Spectral Plotting for listeria-hct-240117

Experimental Spectrum Colour Code:

- Experimental peak matched to a Prosit predicted peak.
- Possible ion unknown to Prosit.
- Precursor matched peak.
- Experimental peak not matched to any potential ion.

Prosit Spectrum Colour Code:

- Prosit predicted peak matched to experimental spectrum.
- Prosit predicted peak not matched to experimental spectrum.

Additional Notes:

° indicates an ion with loss of H_2O.

* indicates an ion with loss of NH_3.

Source LFQ_HCT116-Listeria-3 **Scan** 44078
Peptide NTDYMSGNDFTLY **Charge** 2 **Spectral Angle** 0.94

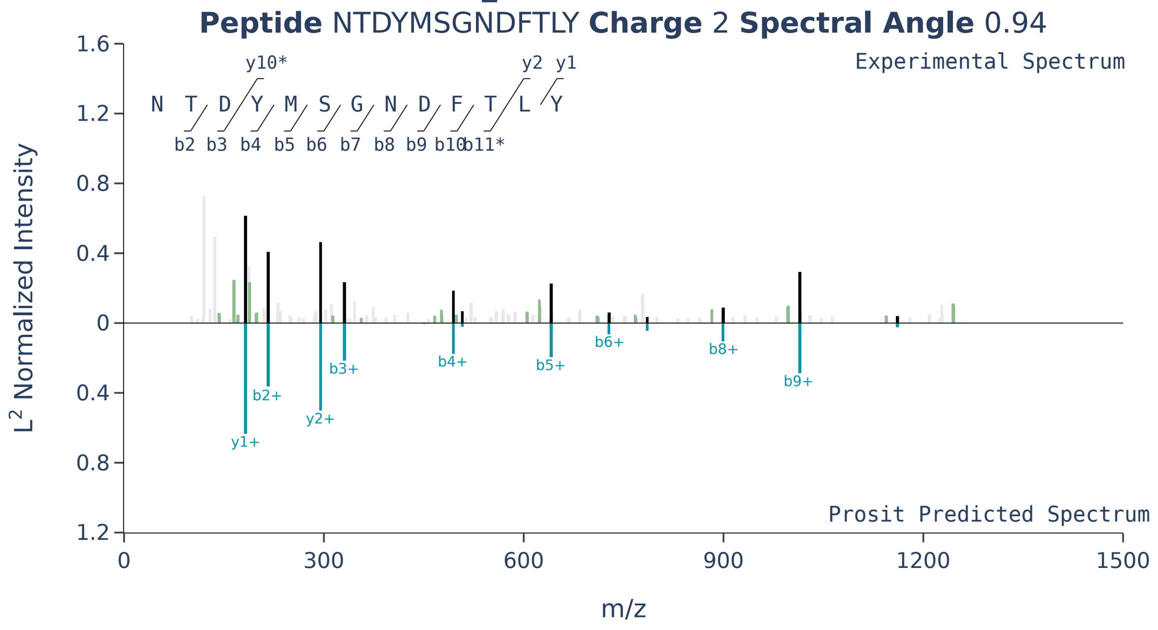

Source LFQ_HCT116-Listeria-1 **Scan** 35293
Peptide LSDYFNNHMSY **Charge** 2 **Spectral Angle** 0.8

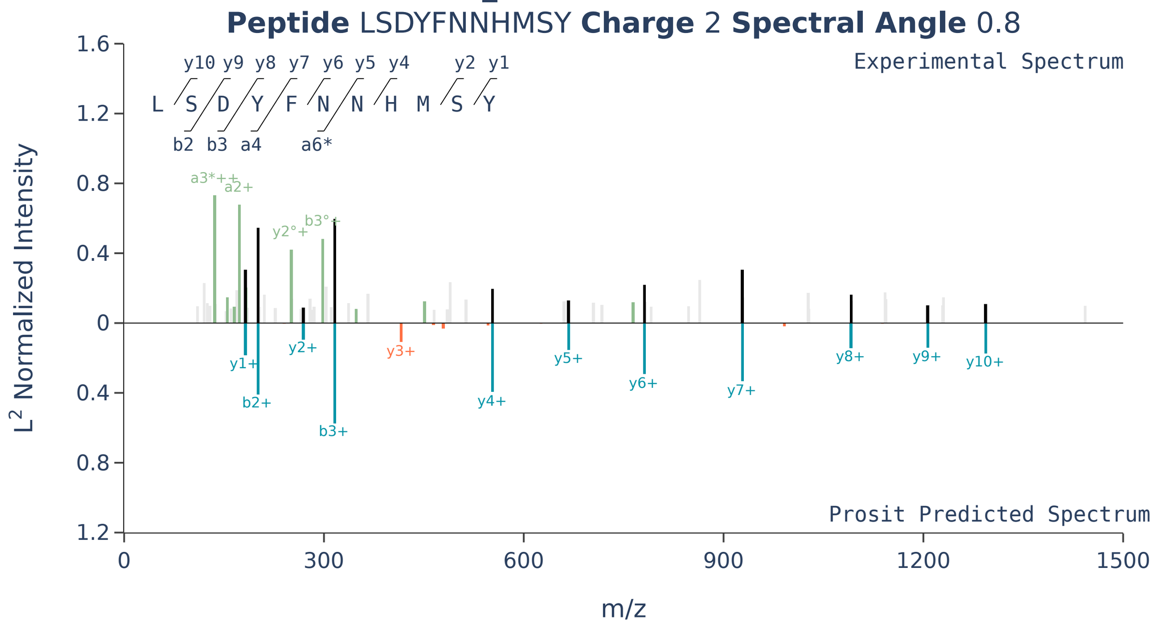

Source LFQ_HCT116-Listeria-3 **Scan** 41175
Peptide SESQLIAKFGTA **Charge** 2 **Spectral Angle** 0.91

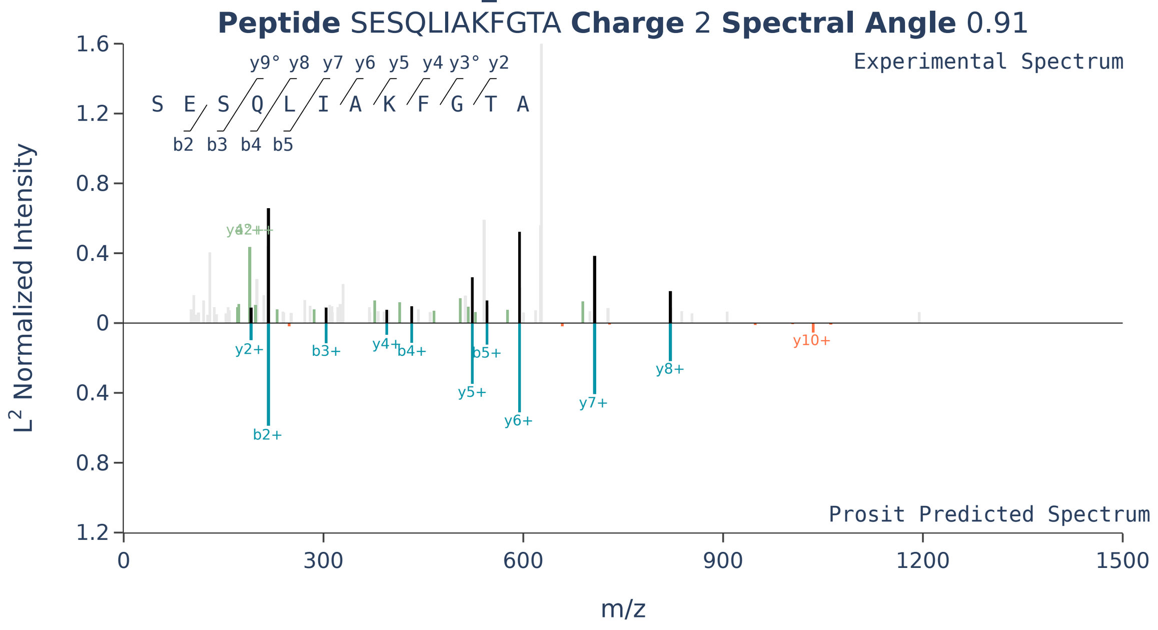

Source LFQ_HCT116-Listeria-1 **Scan** 31401
Peptide VSDEDVDKKFNEY **Charge** 3 **Spectral Angle** 0.87

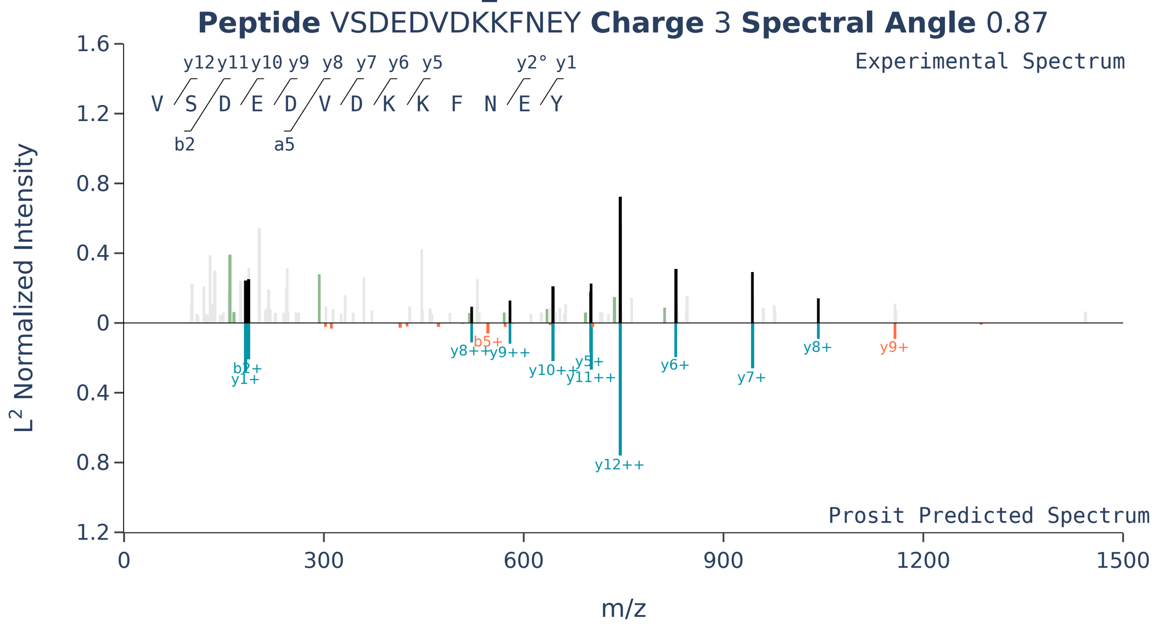

Source LFQ_HCT116-Listeria-1 **Scan** 40525
Peptide LTYPGALVKA **Charge** 2 **Spectral Angle** 0.93

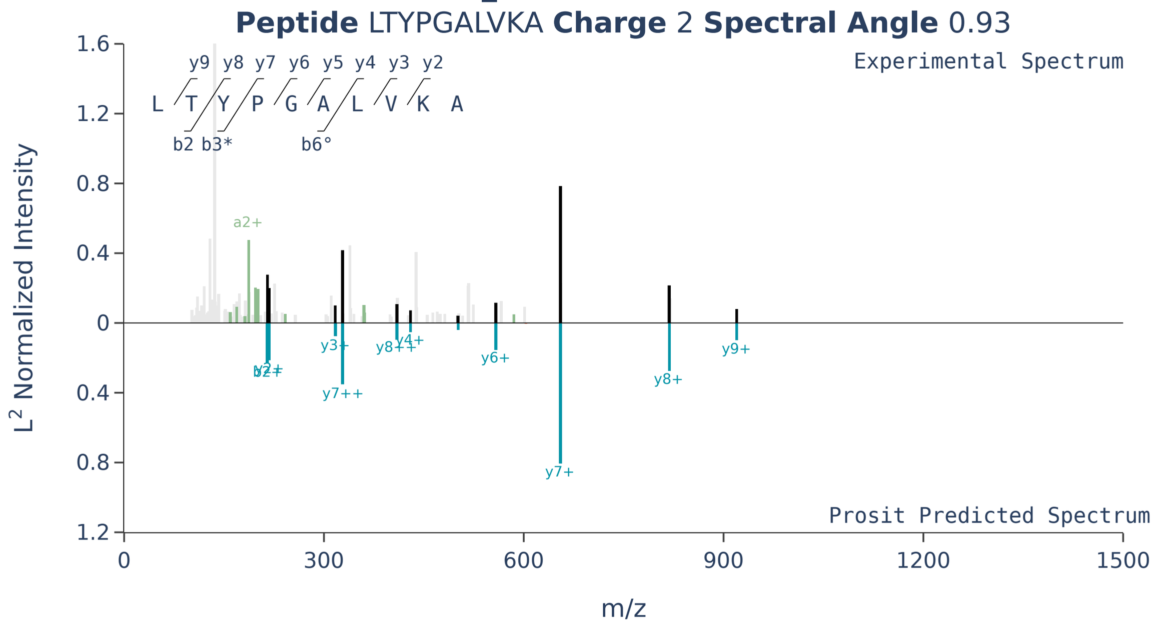

Source LFQ_HCT116-Listeria-4 **Scan** 49188
Peptide LTDDVAIQPLY **Charge** 2 **Spectral Angle** 0.8

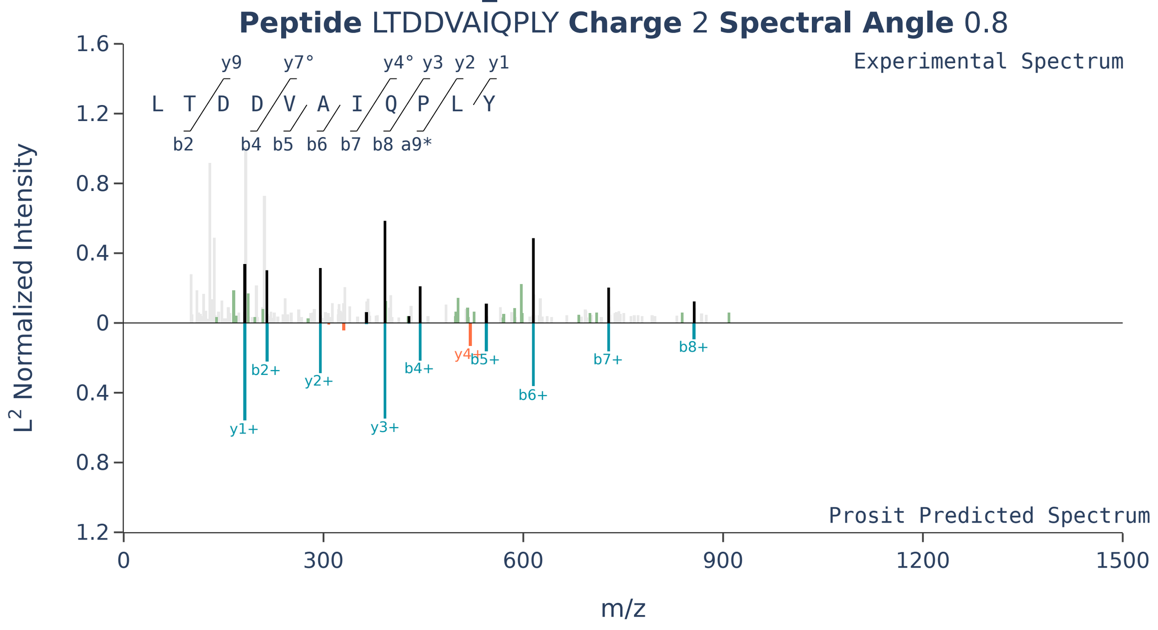

Source LFQ_HCT116-Listeria-1 **Scan** 45926
Peptide YLDESVKSFVDY **Charge** 2 **Spectral Angle** 0.89

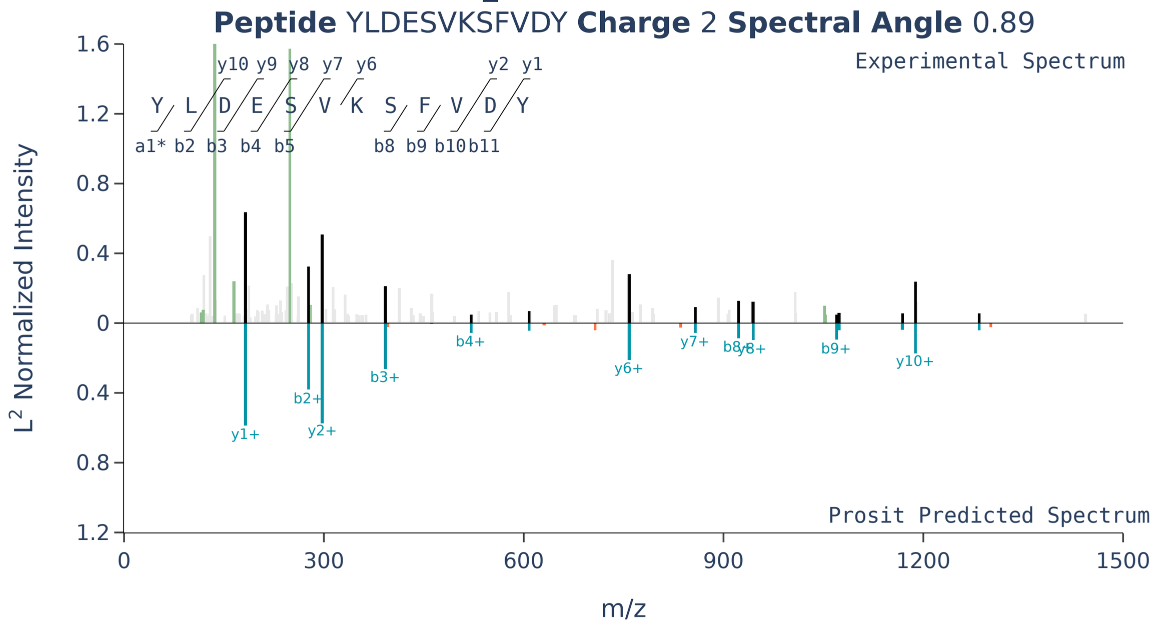

Source LFQ_HCT116-Listeria-1 **Scan** 35781
Peptide VTDLLKNMY **Charge** 2 **Spectral Angle** 0.79

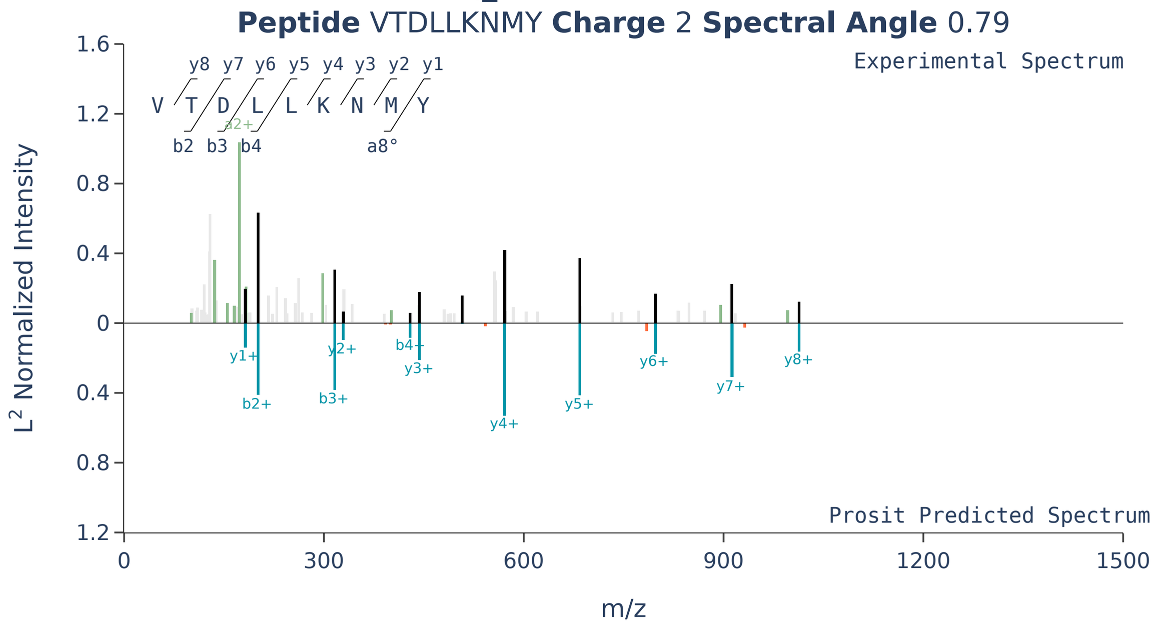

Source LFQ_HCT116-Listeria-4 **Scan** 42338
Peptide ATDPTTLEI **Charge** 1 **Spectral Angle** 0.82

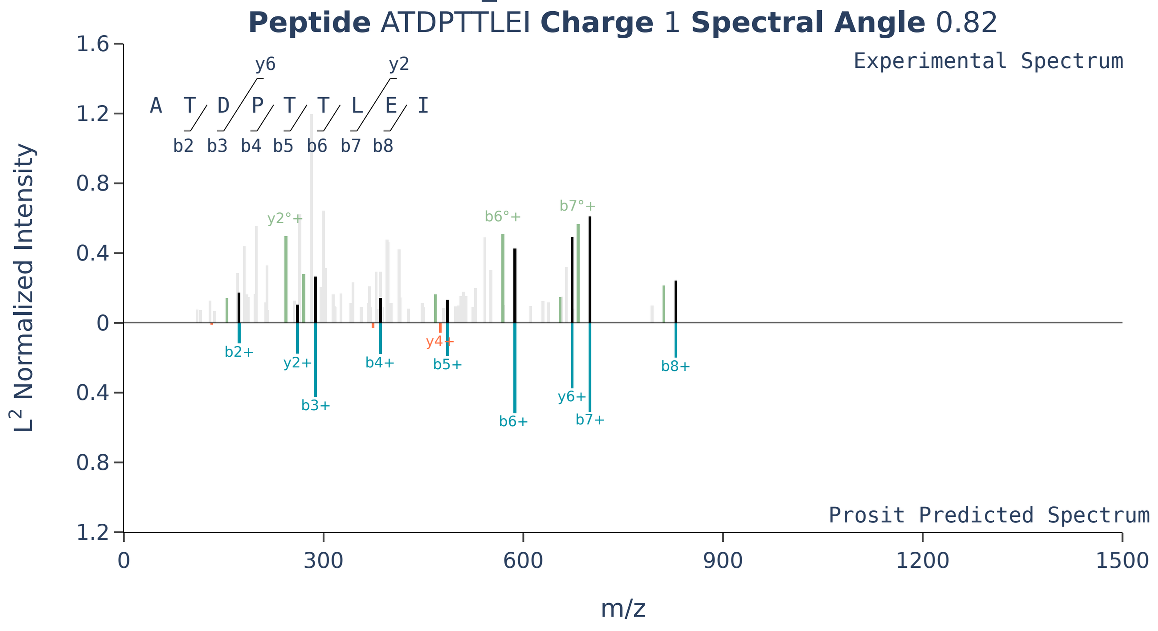

Source LFQ_HCT116-Listeria-1 **Scan** 50868
Peptide YLFDAIKNGGDIVA **Charge** 2 **Spectral Angle** 0.76

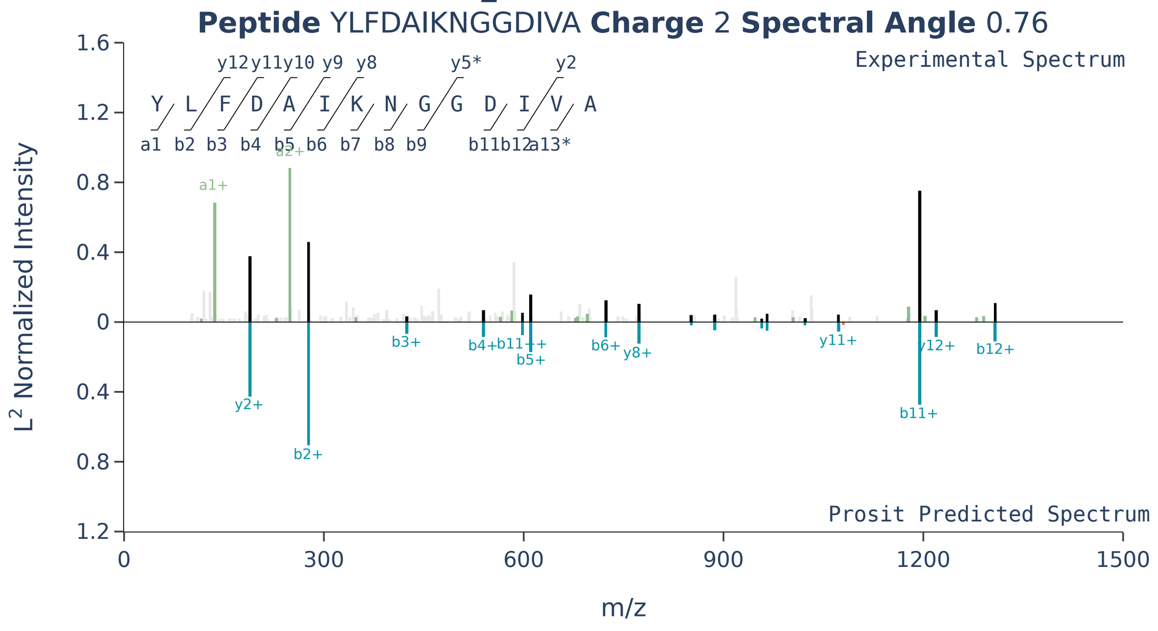

Source LFQ_HCT116-Listeria-2 **Scan** 39429
Peptide HLPEFTNEV **Charge** 2 **Spectral Angle** 0.93

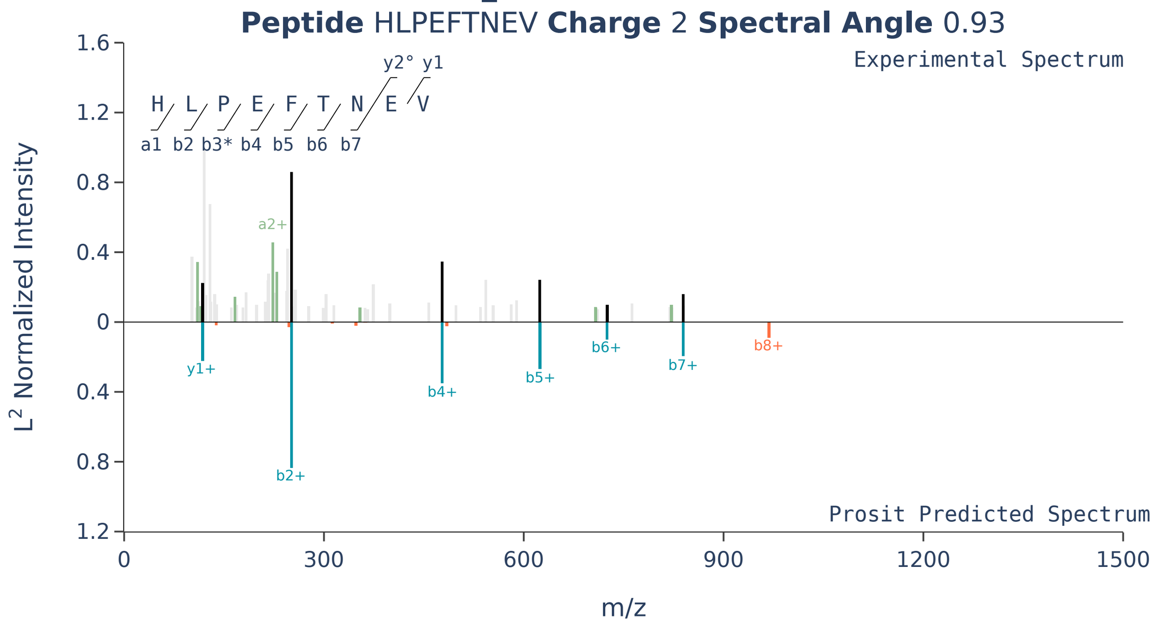

Source LFQ_HCT116-Listeria-1 **Scan** 29465
Peptide KIDYDDEMAY **Charge** 2 **Spectral Angle** 0.8

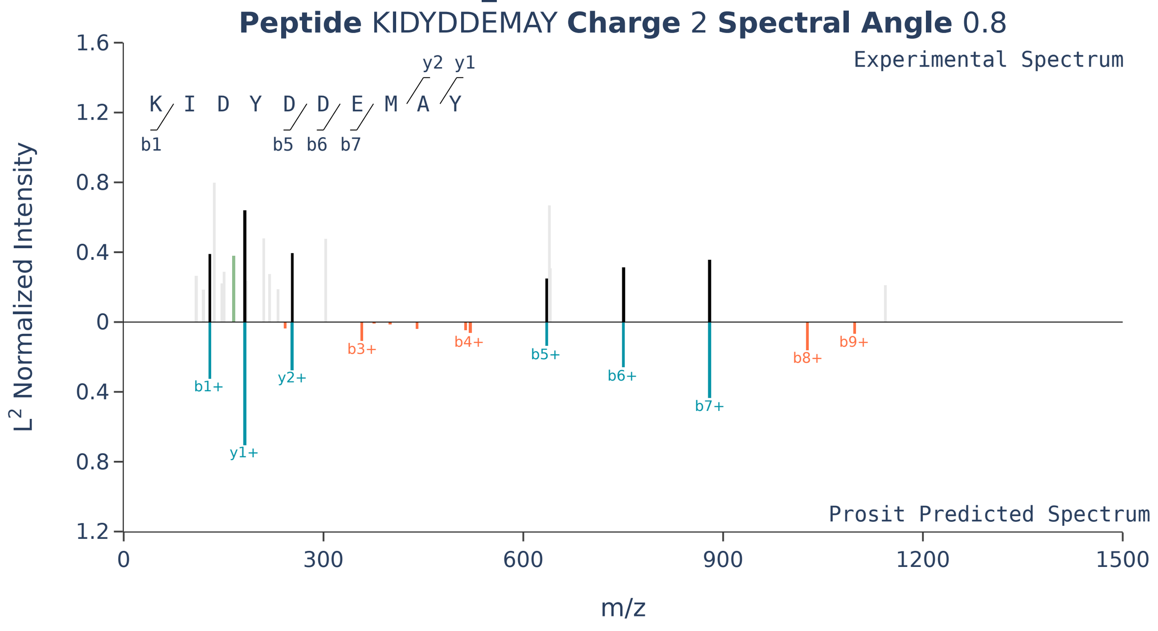

Source LFQ_HCT116-Listeria-1 **Scan** 44737
Peptide EEIFDVKVA **Charge** 2 **Spectral Angle** 0.91

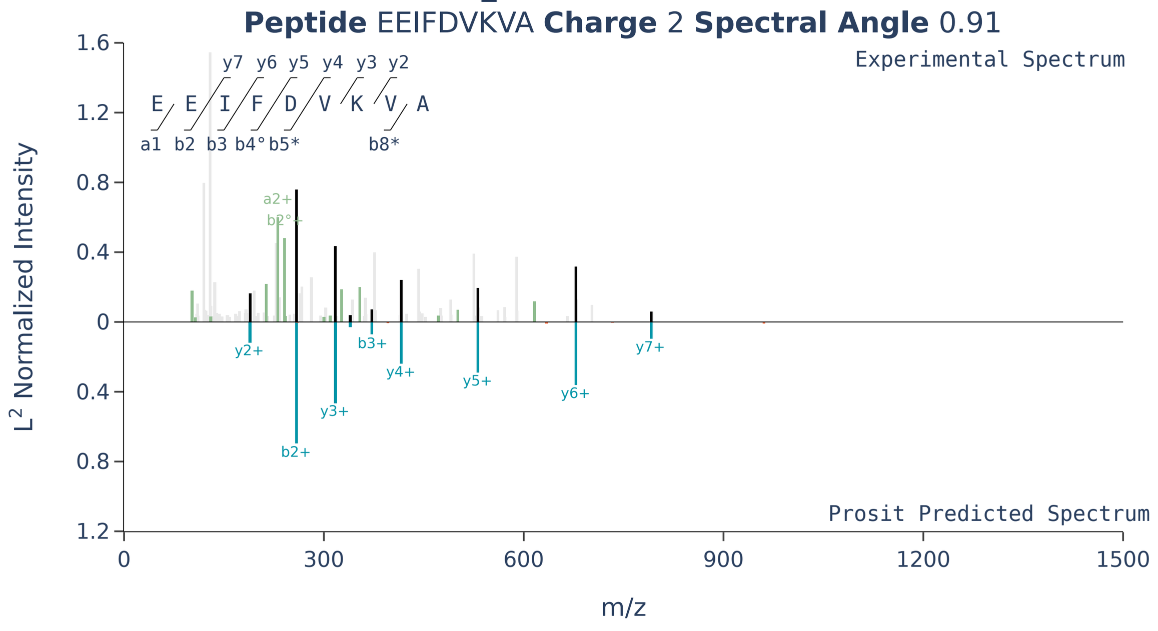

Source LFQ_HCT116-Listeria-4 **Scan** 45661
Peptide ILADIVKNV **Charge** 2 **Spectral Angle** 0.9

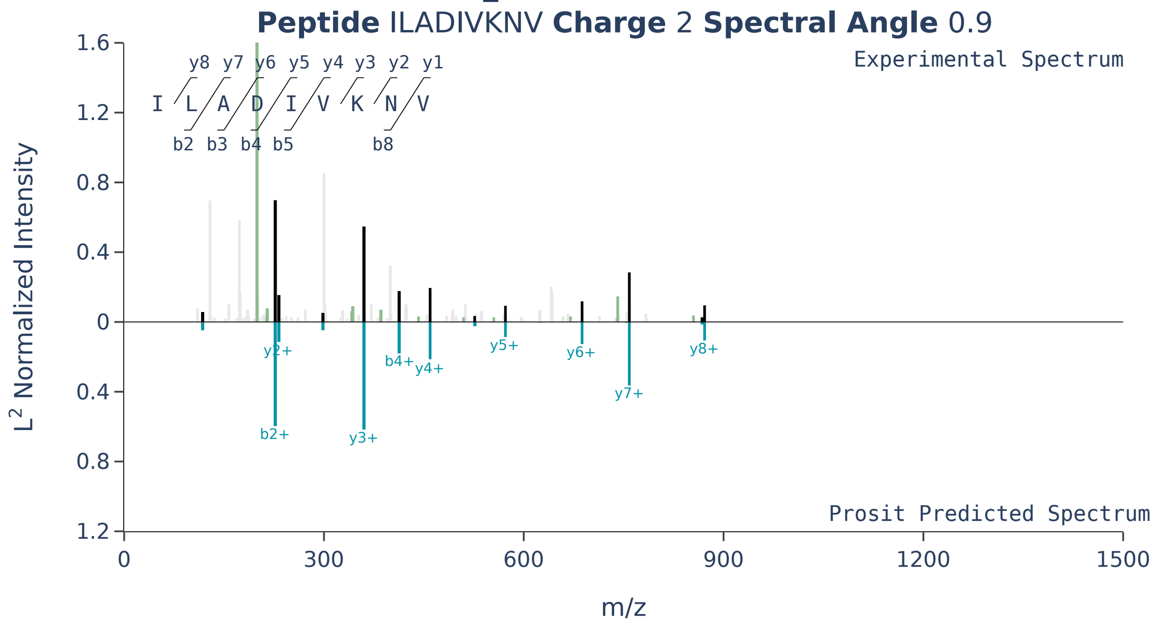

Source LFQ_HCT116-Listeria-1 **Scan** 42966
Peptide VLQQIIHDV **Charge** 2 **Spectral Angle** 0.9

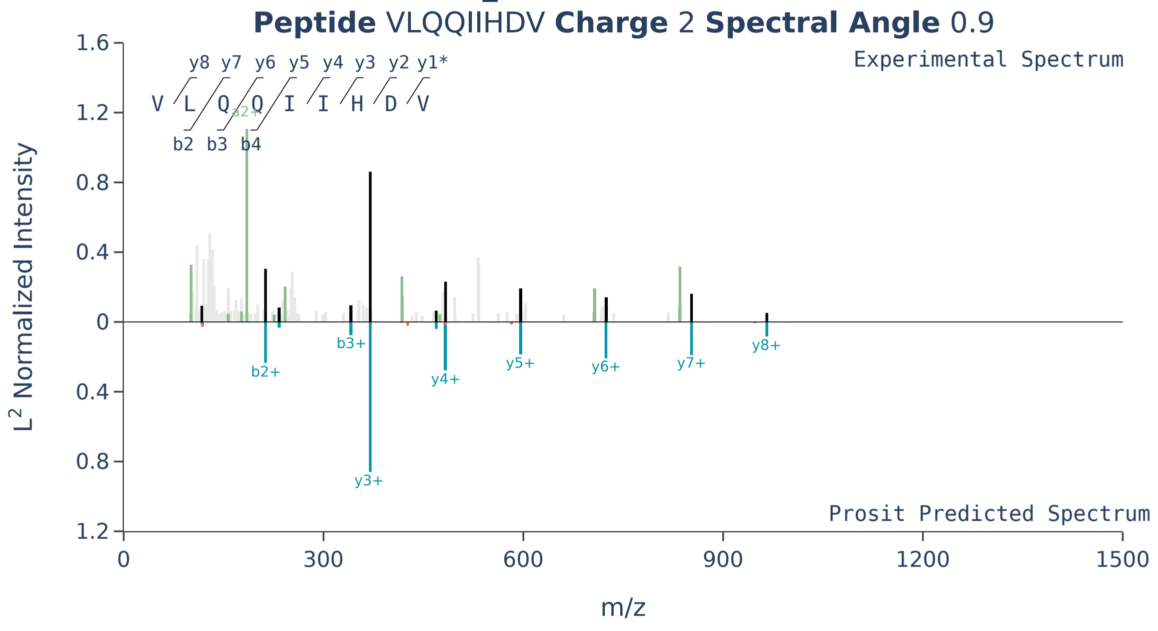

Source LFQ_HCT116-Listeria-1 **Scan** 40642
Peptide SESQLIAKF **Charge** 2 **Spectral Angle** 0.9

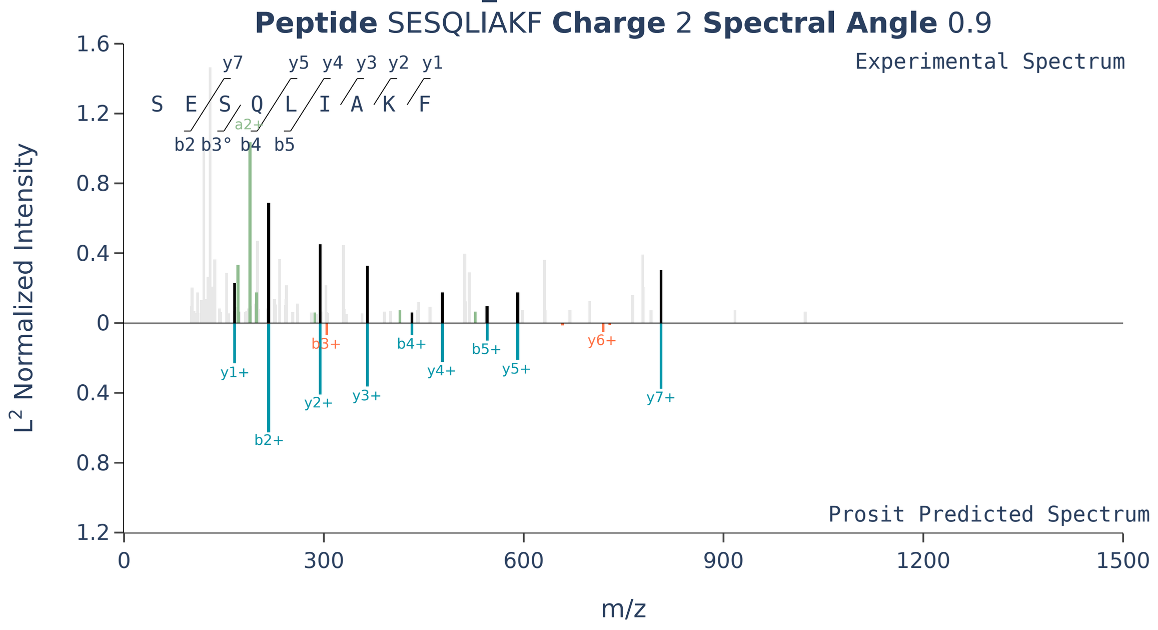

Source LFQ_HCT116-Listeria-4 **Scan** 44226
Peptide YMSGNDFTLY **Charge** 2 **Spectral Angle** 0.88

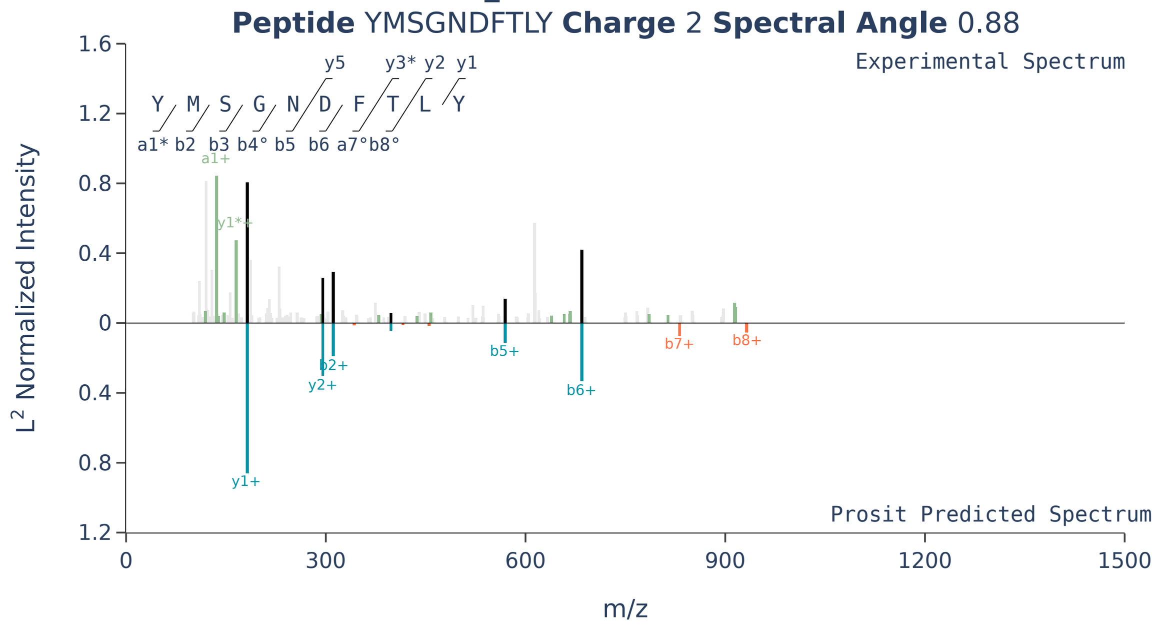

Source LFQ_HCT116-Listeria-4 **Scan** 45132
Peptide HTDLSGSLFY **Charge** 2 **Spectral Angle** 0.89

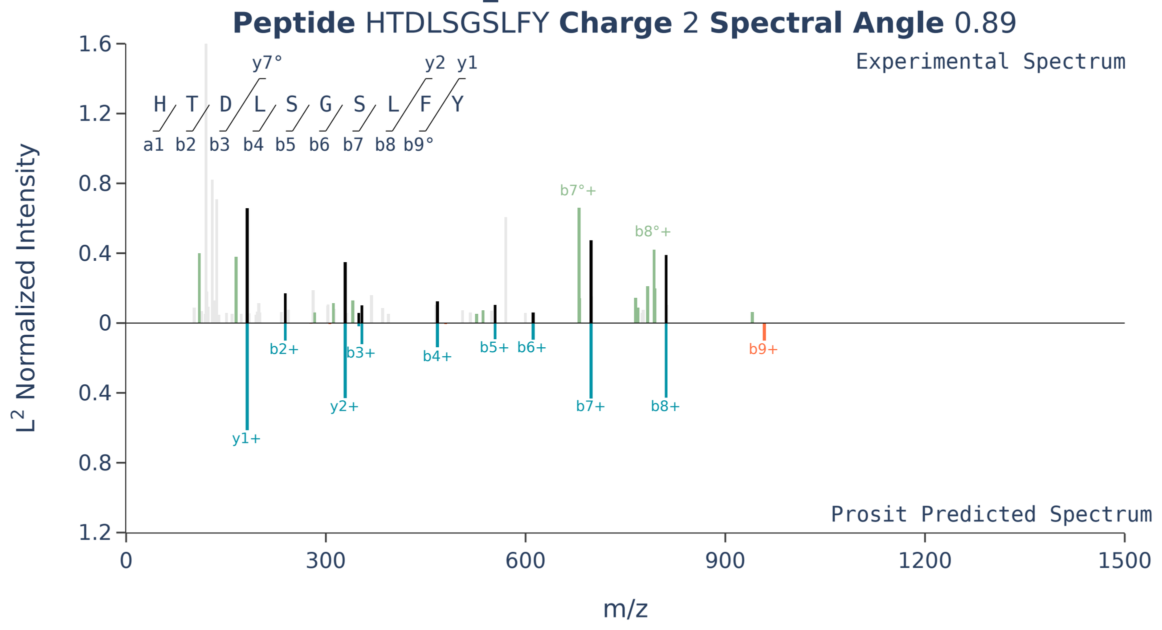

Source LFQ_HCT116-Listeria-1 **Scan** 40435
Peptide KIYHGPIYL **Charge** 2 **Spectral Angle** 0.76

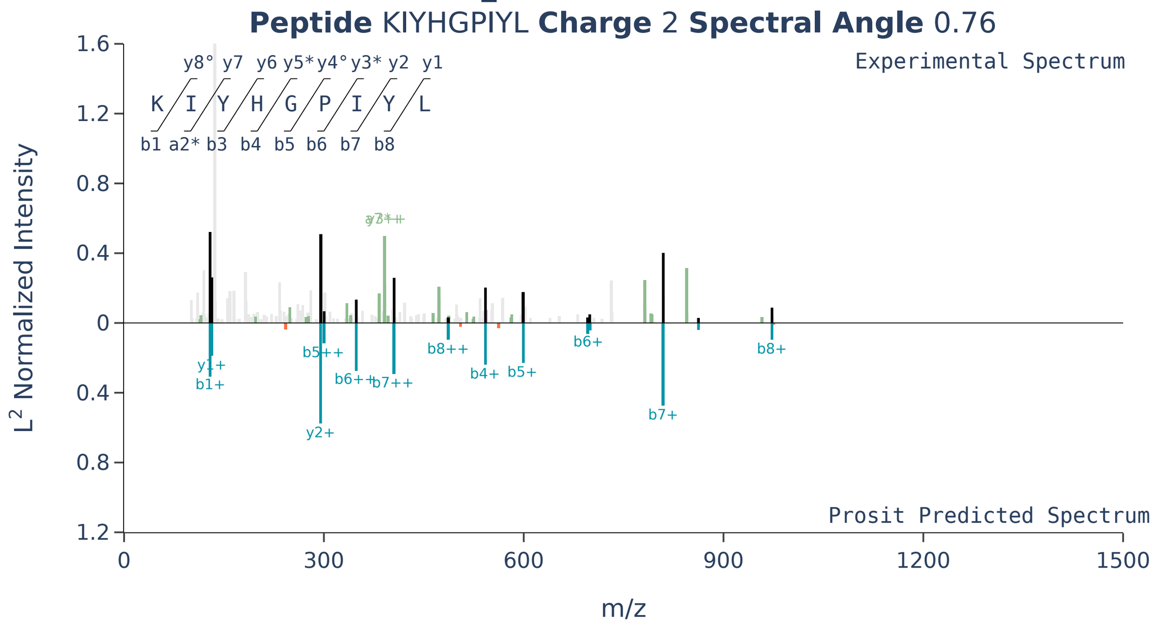

Source LFQ_HCT116-Listeria-1 **Scan** 48976
Peptide LGDRVVIEVLE **Charge** 2 **Spectral Angle** 0.89

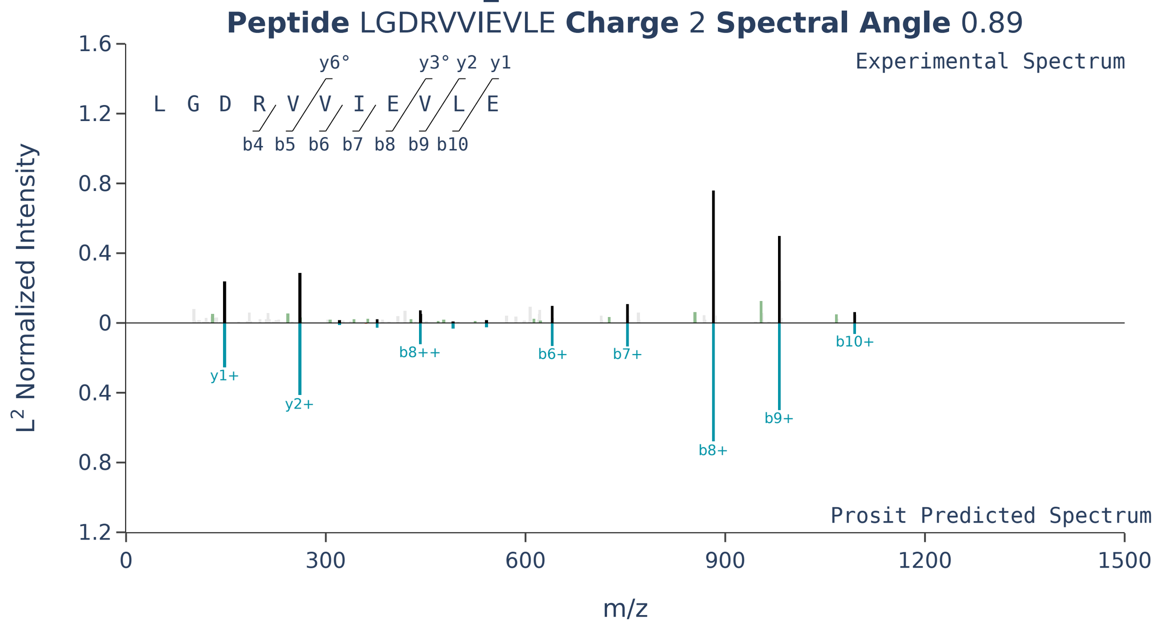

Source LFQ_HCT116-Listeria-3 **Scan** 41593
Peptide DSELESLTY **Charge** 1 **Spectral Angle** 0.93

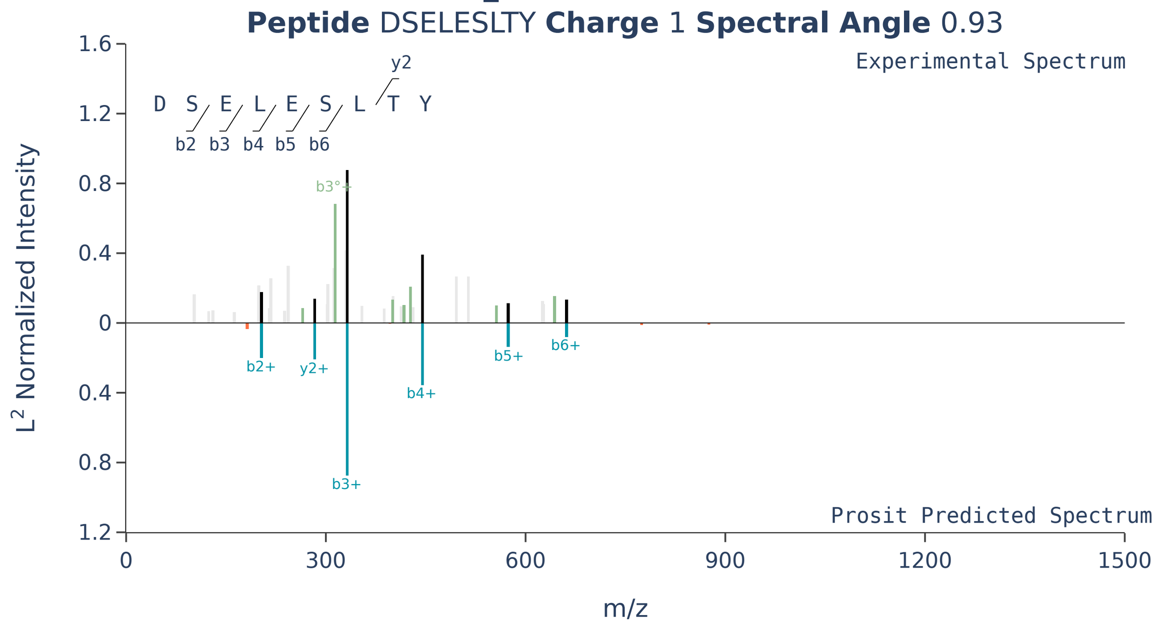

Source LFQ_HCT116-Listeria-1 **Scan** 27689
Peptide NMLSEVERA **Charge** 2 **Spectral Angle** 0.89

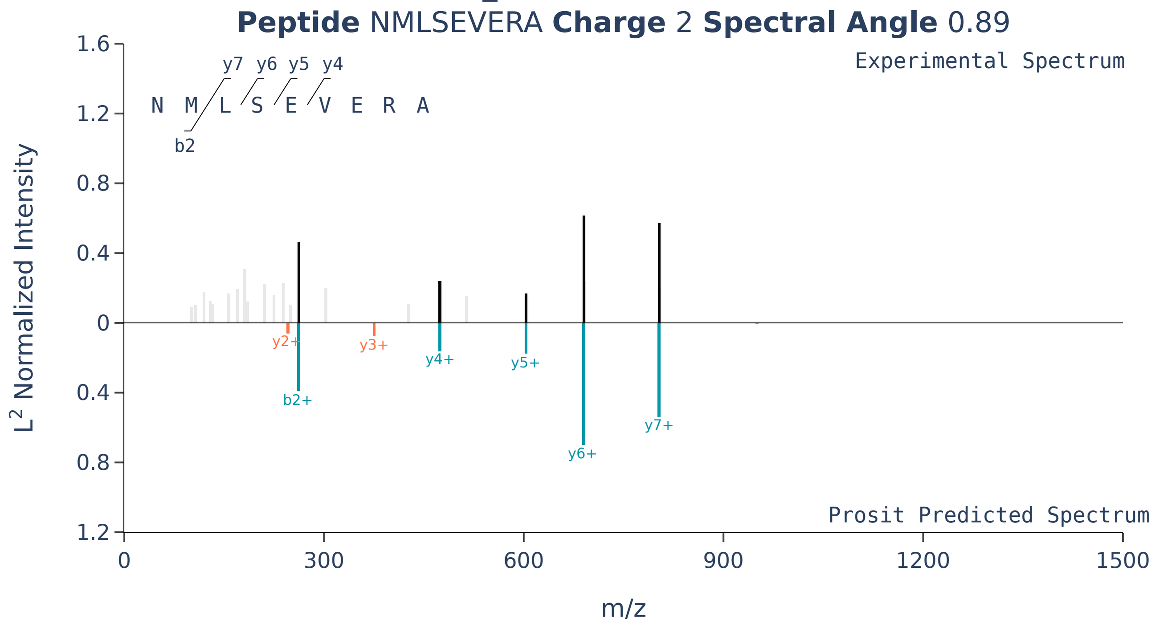

Source LFQ_HCT116-Listeria-4 **Scan** 27770
Peptide RTDSGDHLVY **Charge** 2 **Spectral Angle** 0.9

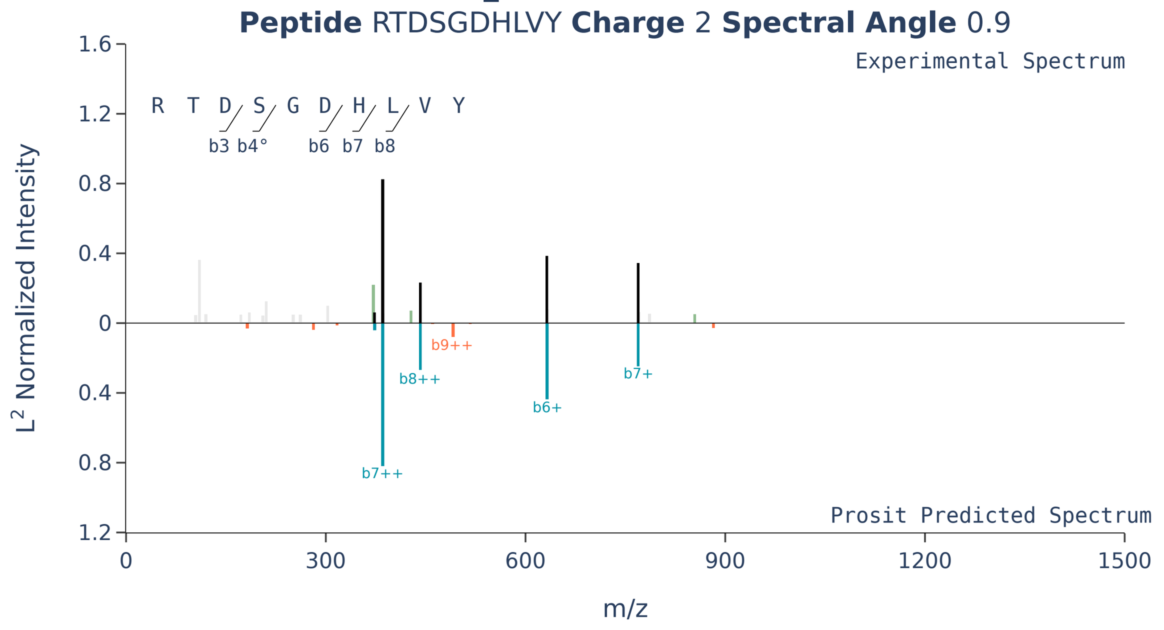

Source LFQ_HCT116-Listeria-3 **Scan** 49506
Peptide TTELQTLIY **Charge** 2 **Spectral Angle** 0.88

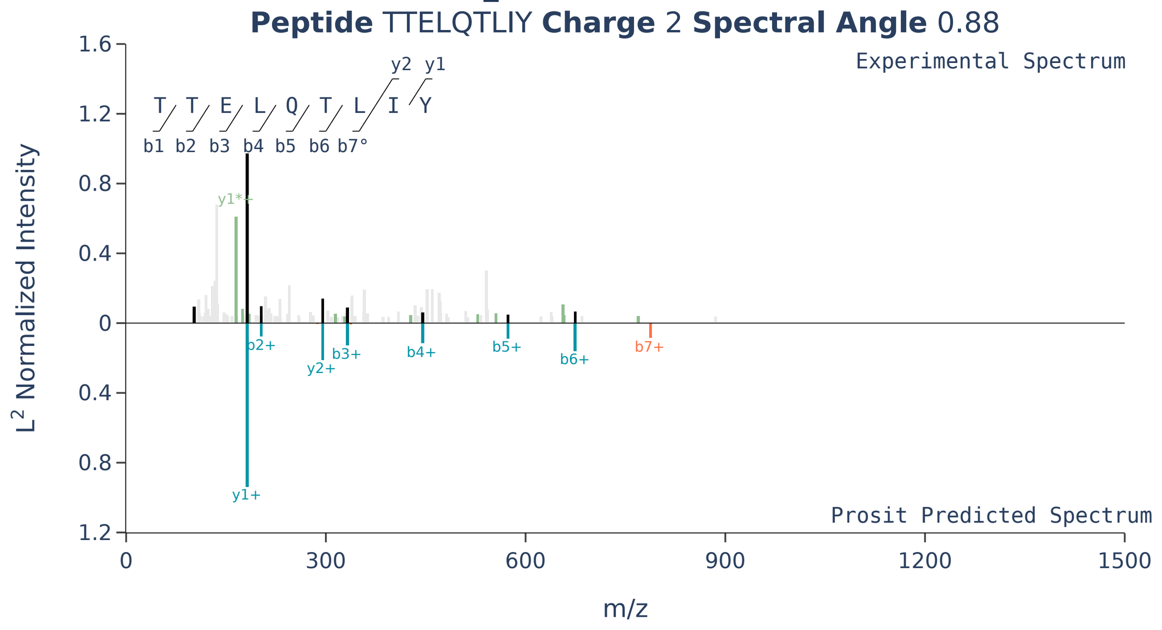

Source LFQ_HCT116-Listeria-4 **Scan** 40849
Peptide LSEIPKEYDVY **Charge** 2 **Spectral Angle** 0.77

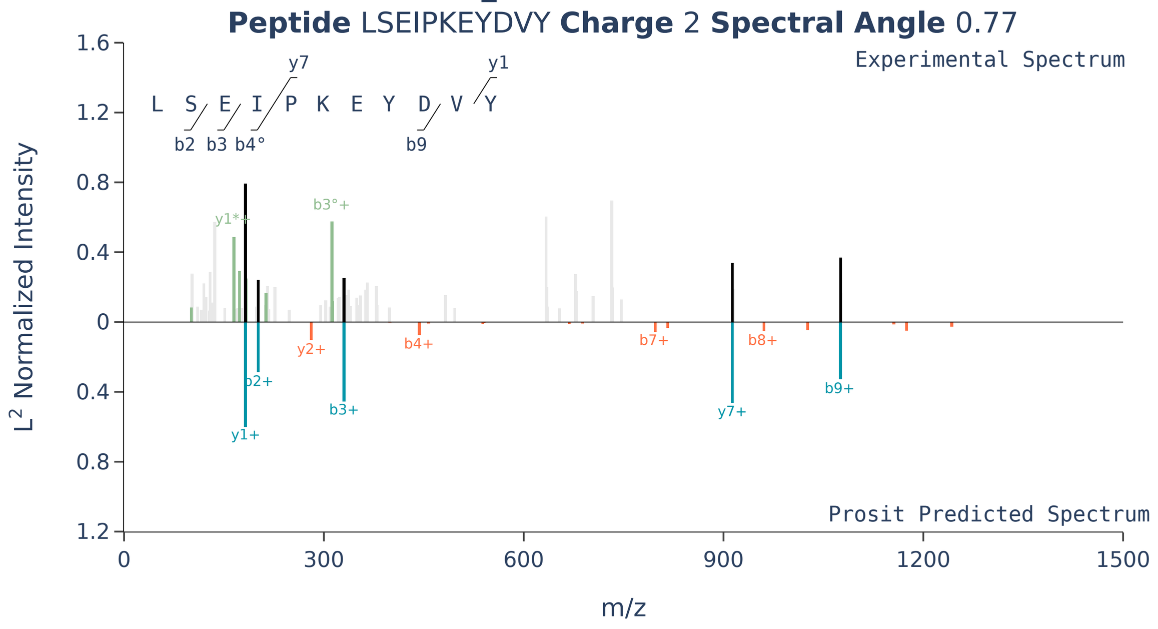

Source LFQ_HCT116-Listeria-1 **Scan** 52202
Peptide LGDRVVIEVLEA **Charge** 2 **Spectral Angle** 0.94

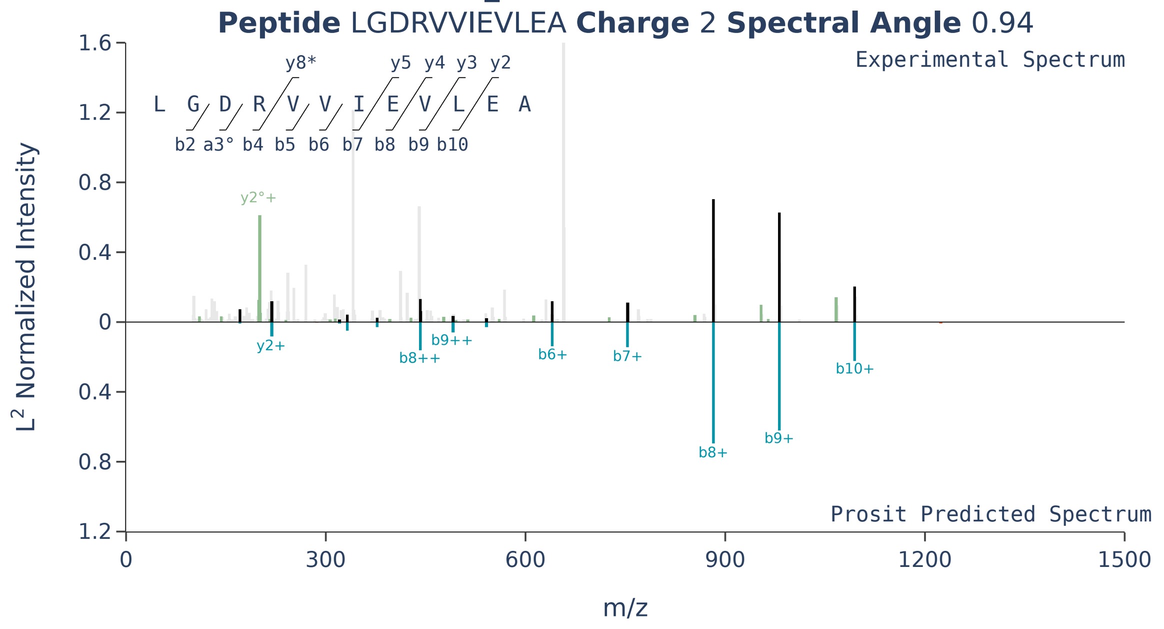

Source LFQ_HCT116-Listeria-3 **Scan** 57510
Peptide KINDLFYLV **Charge** 2 **Spectral Angle** 0.72

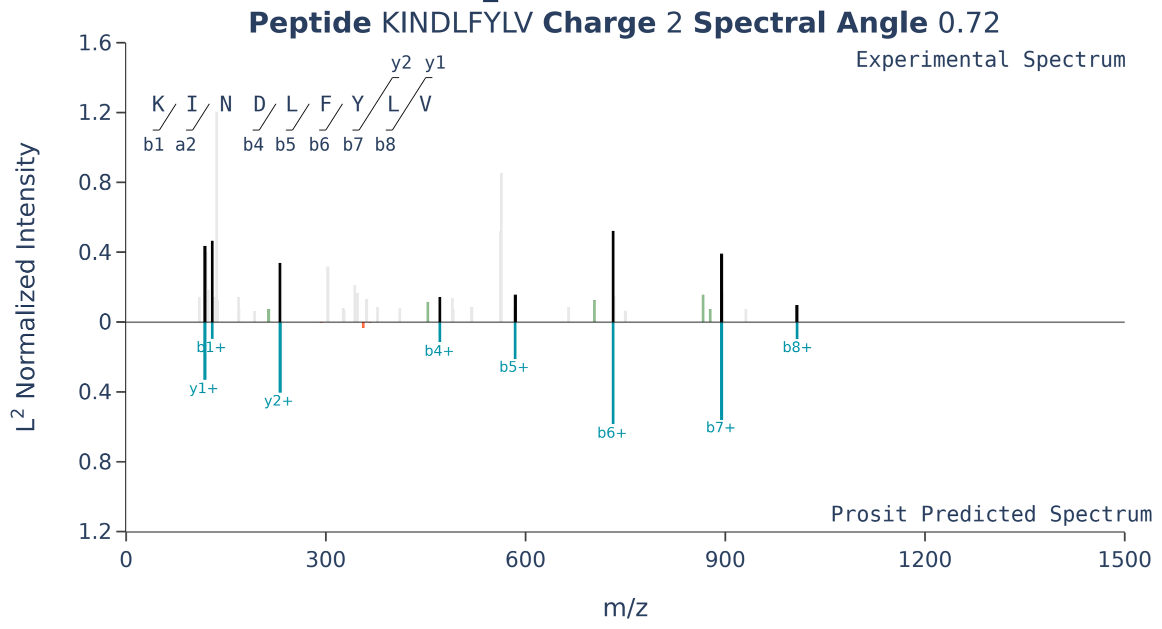

Source LFQ_HCT116-Listeria-1 **Scan** 42809
Peptide EEIRTIFTA **Charge** 2 **Spectral Angle** 0.79

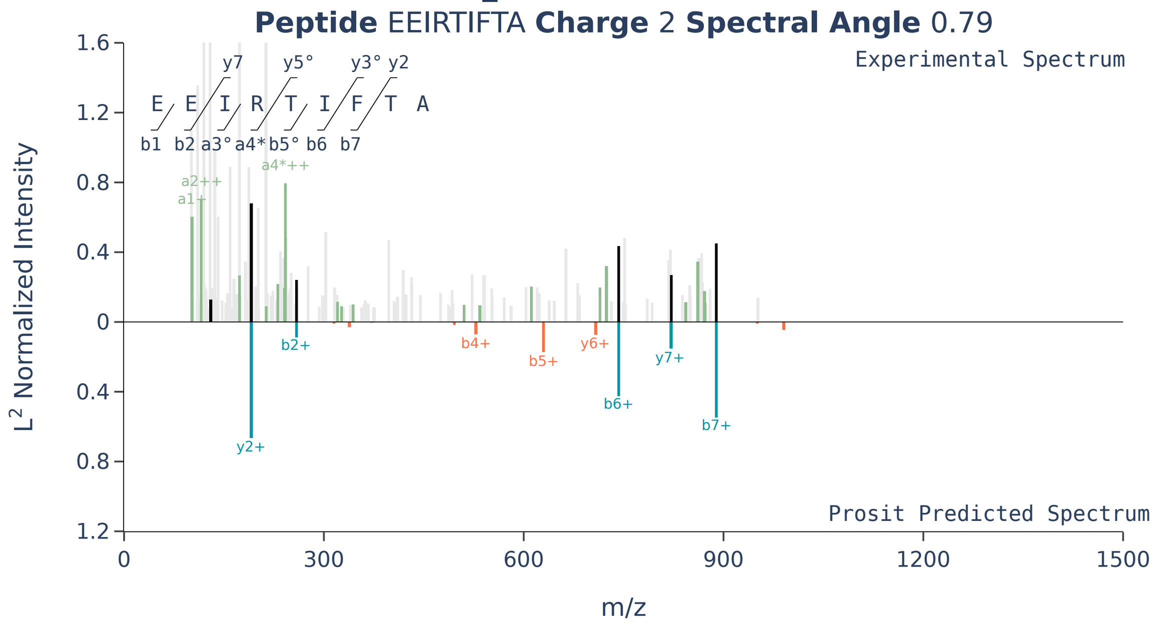

Source LFQ_HCT116-Listeria-1 **Scan** 40733
Peptide RRNPYSIVL **Charge** 2 **Spectral Angle** 0.89

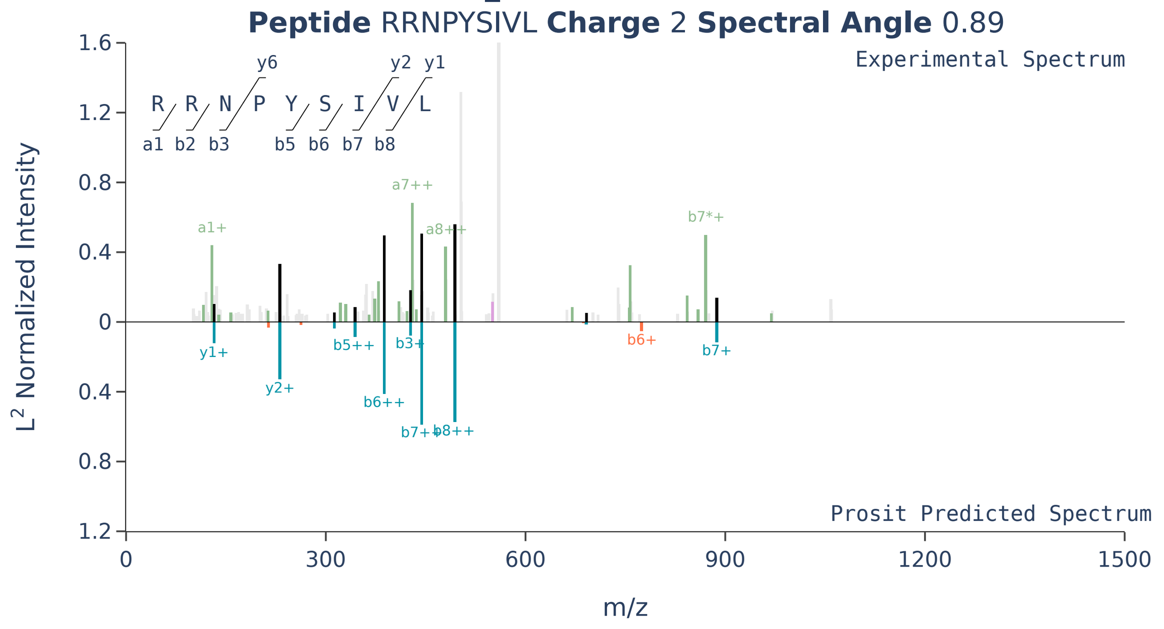

Source LFQ_HCT116-Listeria-1 **Scan** 44536
Peptide NILGQSSFNY **Charge** 2 **Spectral Angle** 0.84

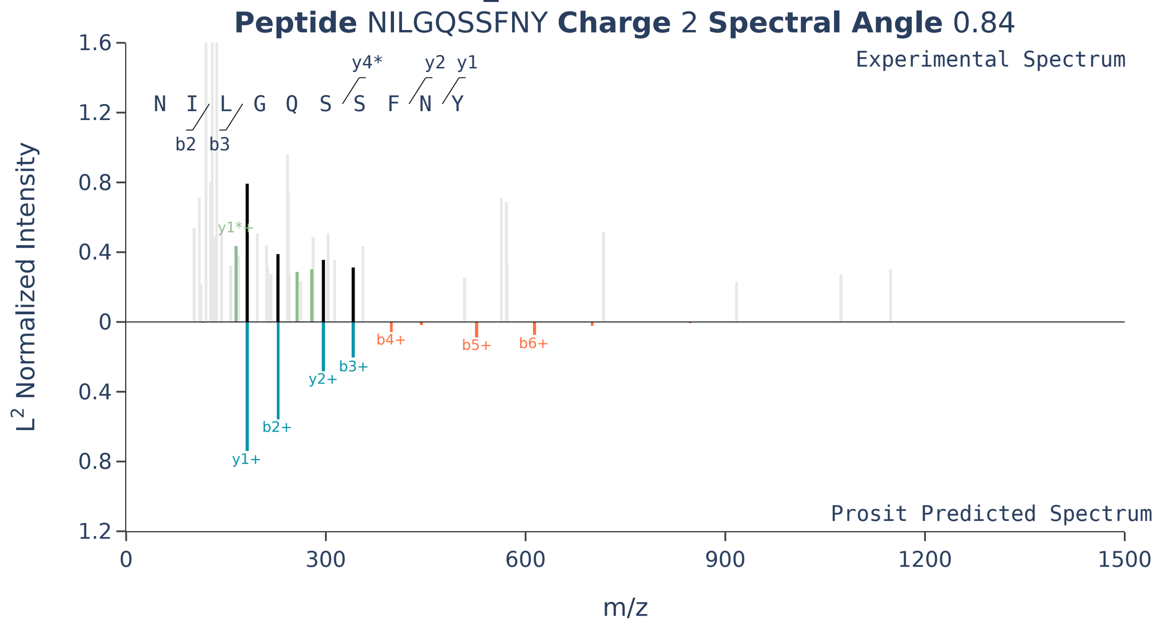

inSPIRE Spectral Plotting for listeria-hela-240117

Experimental Spectrum Colour Code:

- Experimental peak matched to a Prosit predicted peak.
- Possible ion unknown to Prosit.
- Precursor matched peak.
- Experimental peak not matched to any potential ion.

Prosit Spectrum Colour Code:

- Prosit predicted peak matched to experimental spectrum.
- Prosit predicted peak not matched to experimental spectrum.

Additional Notes:

° indicates an ion with loss of H_2O.

* indicates an ion with loss of NH_3.

Source UnLabeled_HeLa_w_Listeria_rep2 **Scan** 40354
Peptide ETFFPQKESYV **Charge** 2 **Spectral Angle** 0.92

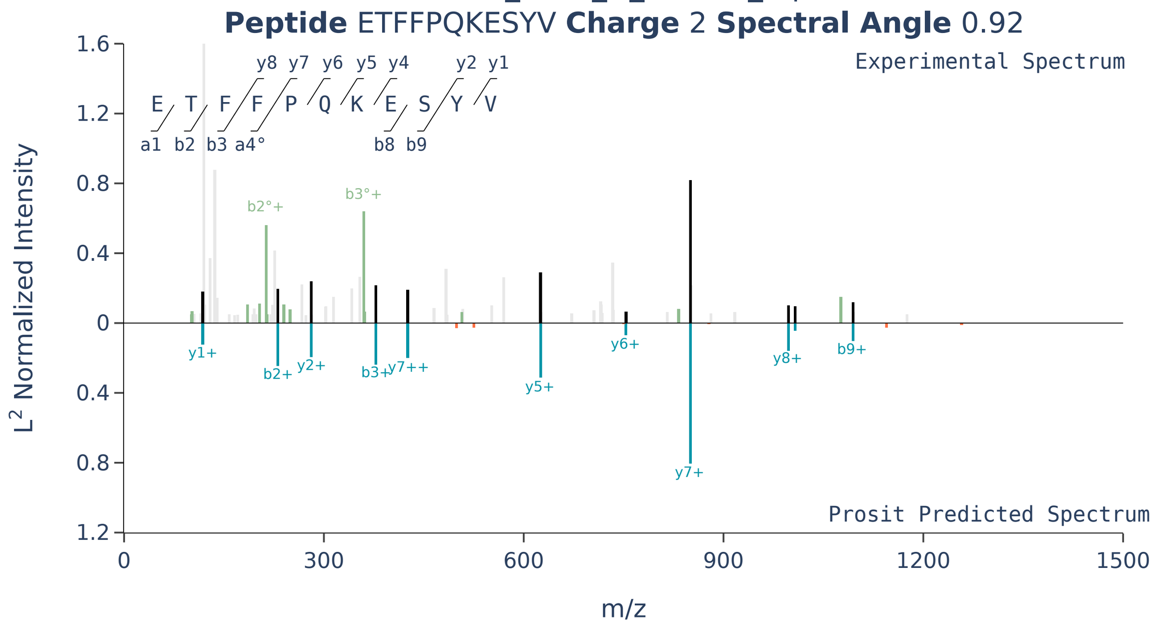

Source UnLabeled_HeLa_w_Listeria_rep1 **Scan** 48948
Peptide EVISFKQIYYNV **Charge** 2 **Spectral Angle** 0.9

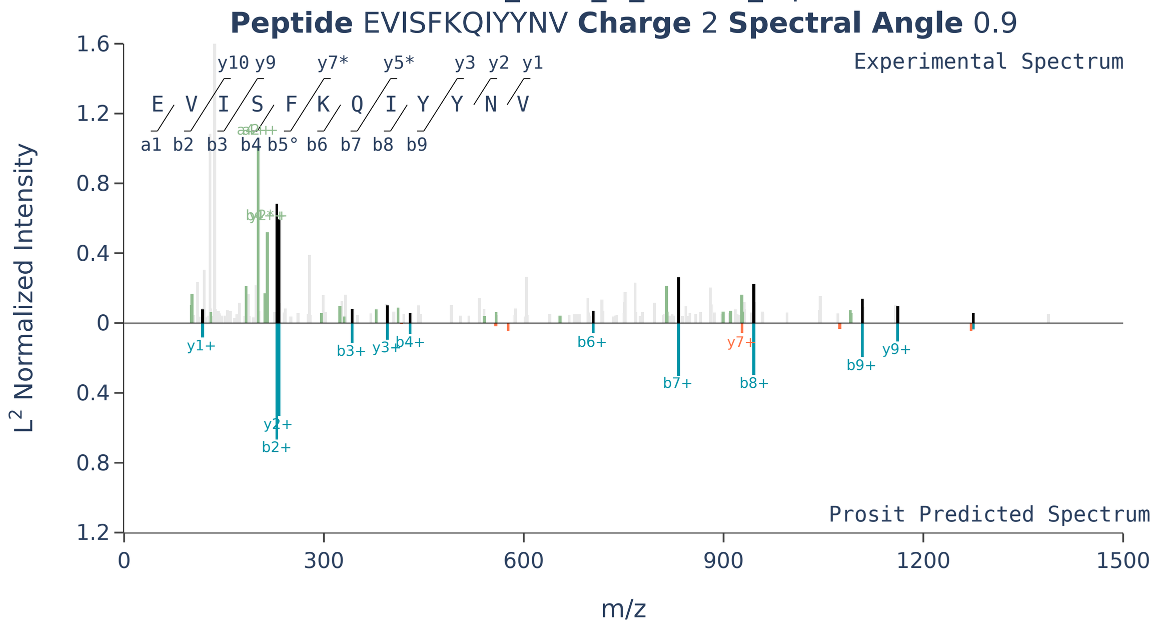

Source UnLabeled_HeLa_w_Listeria_rep1 **Scan** 43469
Peptide EVFDKYFELK **Charge** 3 **Spectral Angle** 0.92

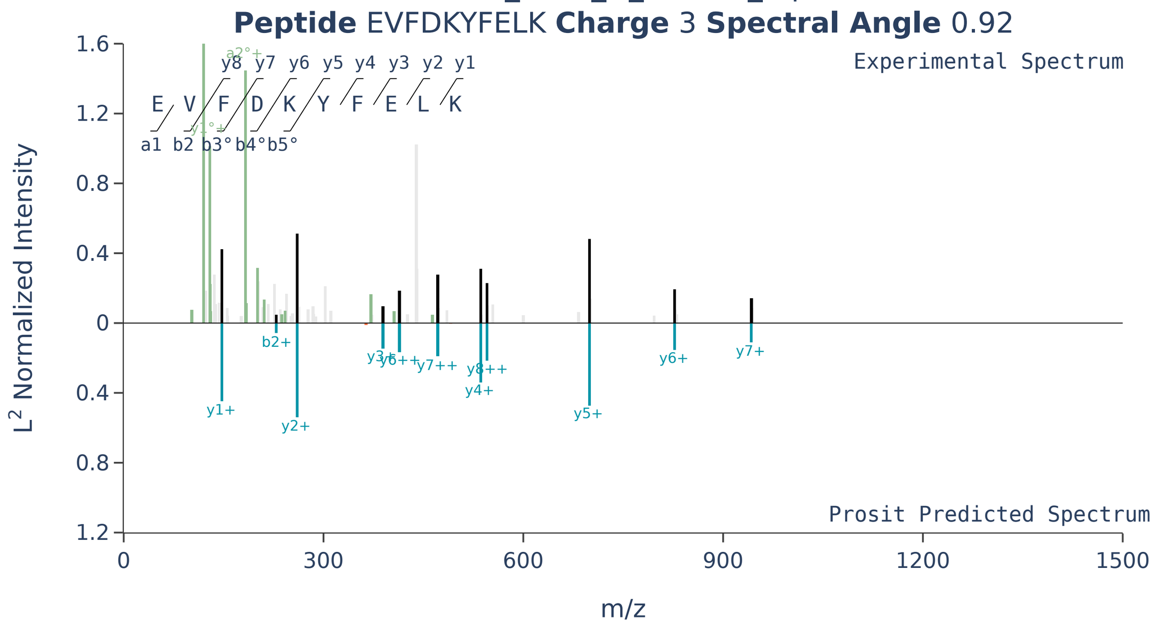

Source UnLabeled_HeLa_w_Listeria_rep4 **Scan** 35933
Peptide VAYGRQVYL **Charge** 2 **Spectral Angle** 0.89

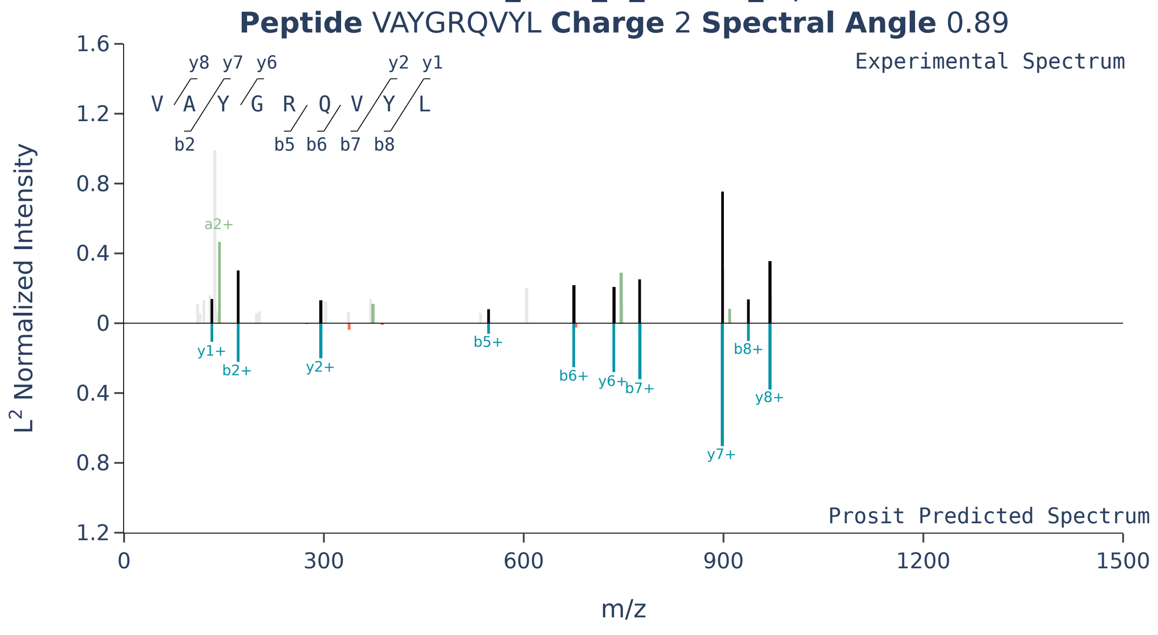

Source UnLabeled_HeLa_w_Listeria_rep1 **Scan** 46974
Peptide TTFGPGIKVDPASL **Charge** 2 **Spectral Angle** 0.82

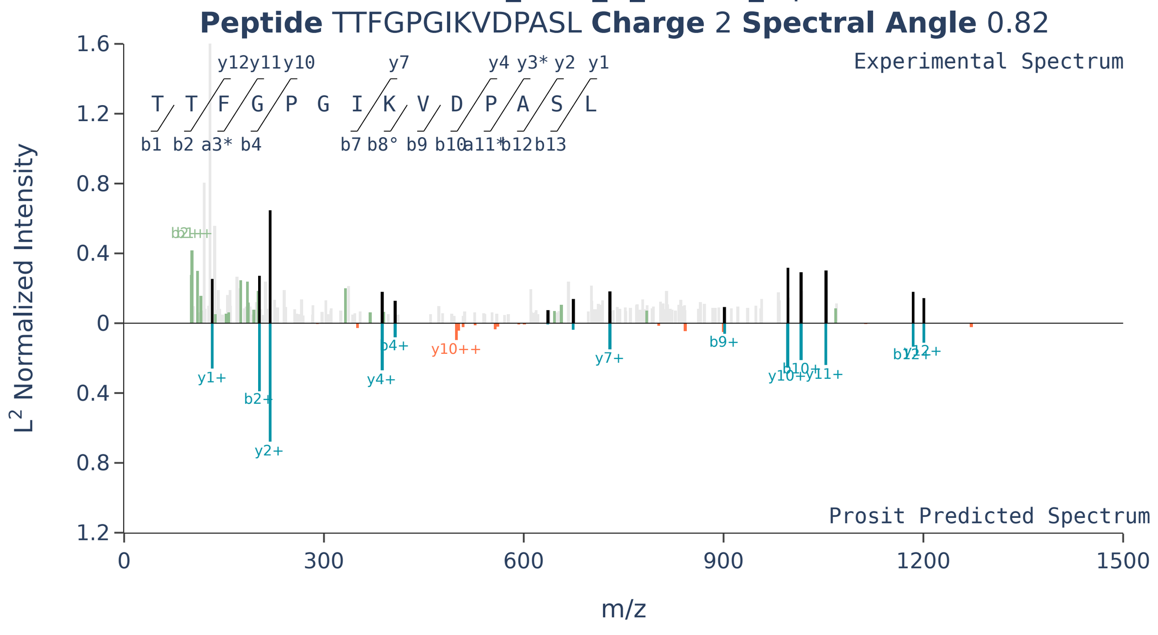

Source UnLabeled_HeLa_w_Listeria_rep1 **Scan** 49929
Peptide EVFDKYFEL **Charge** 2 **Spectral Angle** 0.82

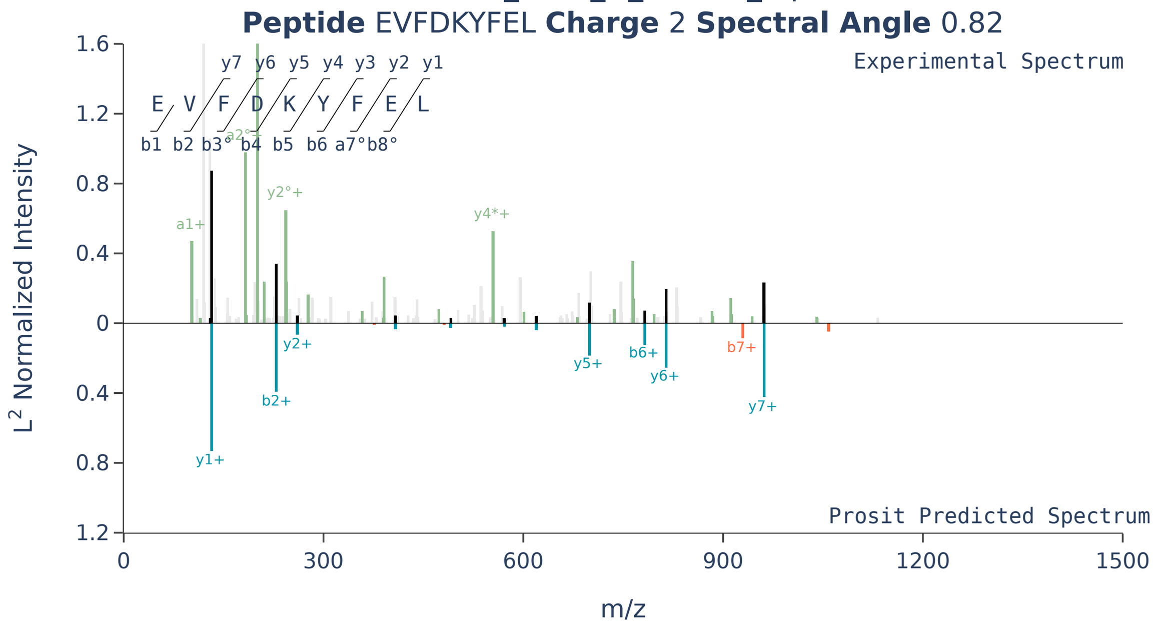

Source UnLabeled_HeLa_w_Listeria_rep2 **Scan** 52126
Peptide DLFDRAFVV **Charge** 2 **Spectral Angle** 0.91

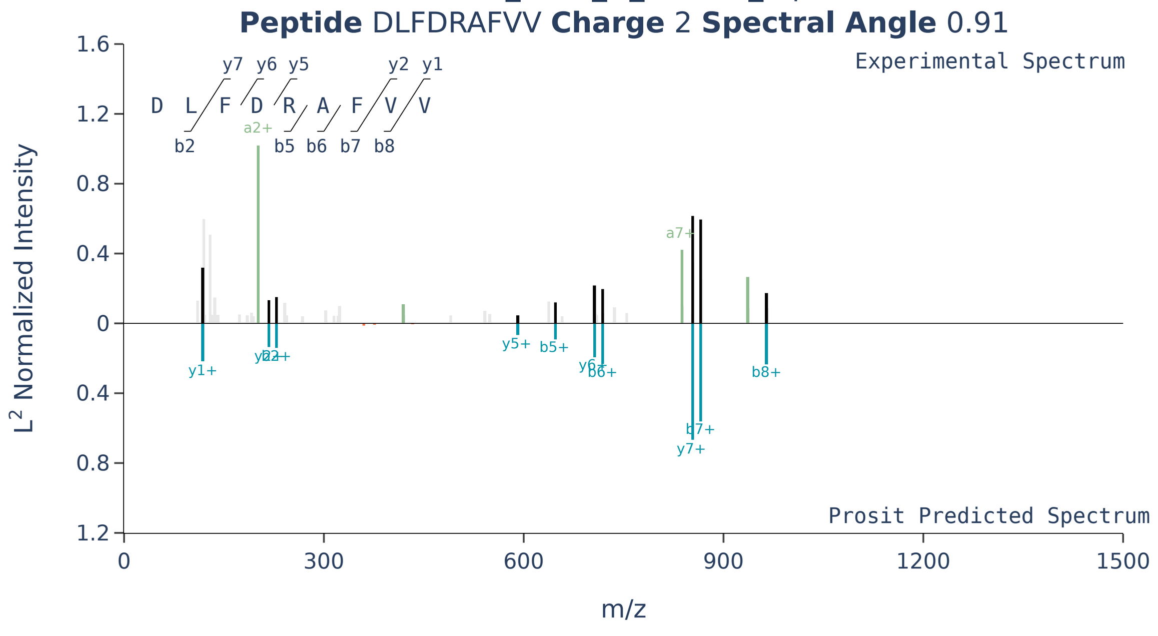

Source UnLabeled_HeLa_w_Listeria_rep1 **Scan** 43321
Peptide ILADIVKNV **Charge** 2 **Spectral Angle** 0.88

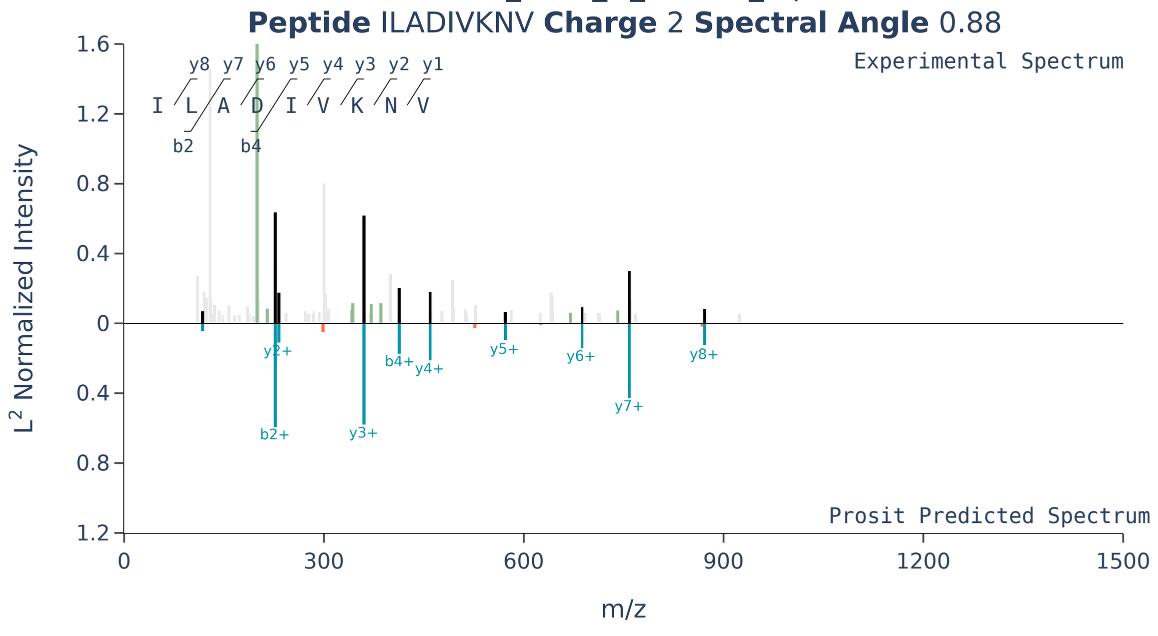

Source UnLabeled_HeLa_w_Listeria_rep4 **Scan** 51238
Peptide EVASWIVQV **Charge** 1 **Spectral Angle** 0.88

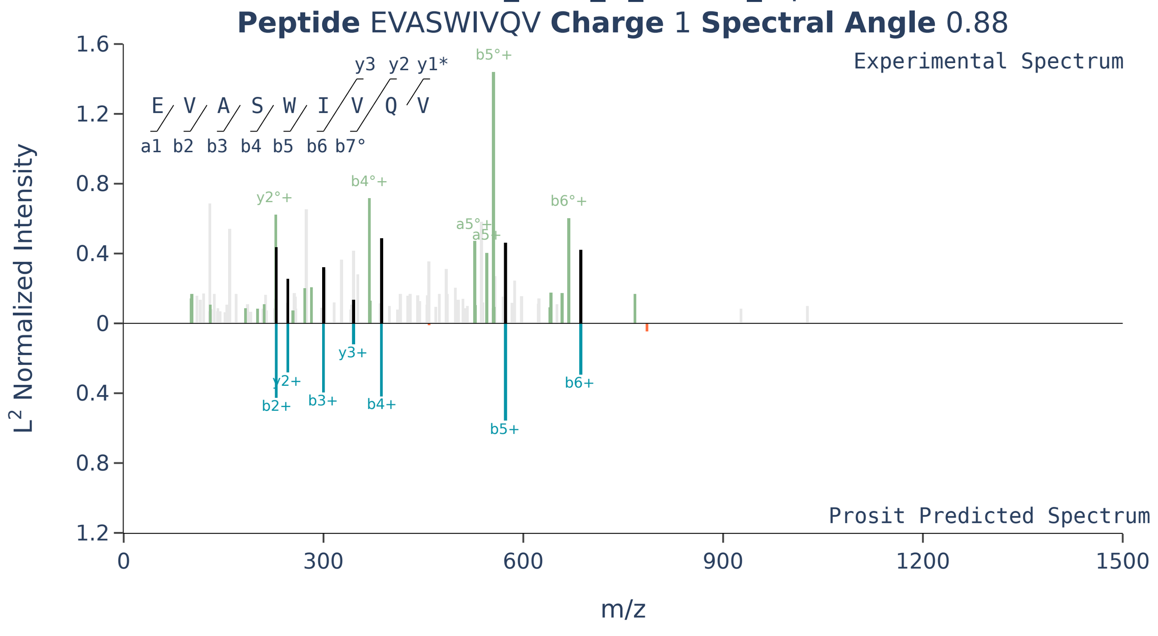

Source UnLabeled_HeLa_w_Listeria_rep2 **Scan** 49110
Peptide TNSEIILPV **Charge** 1 **Spectral Angle** 0.84

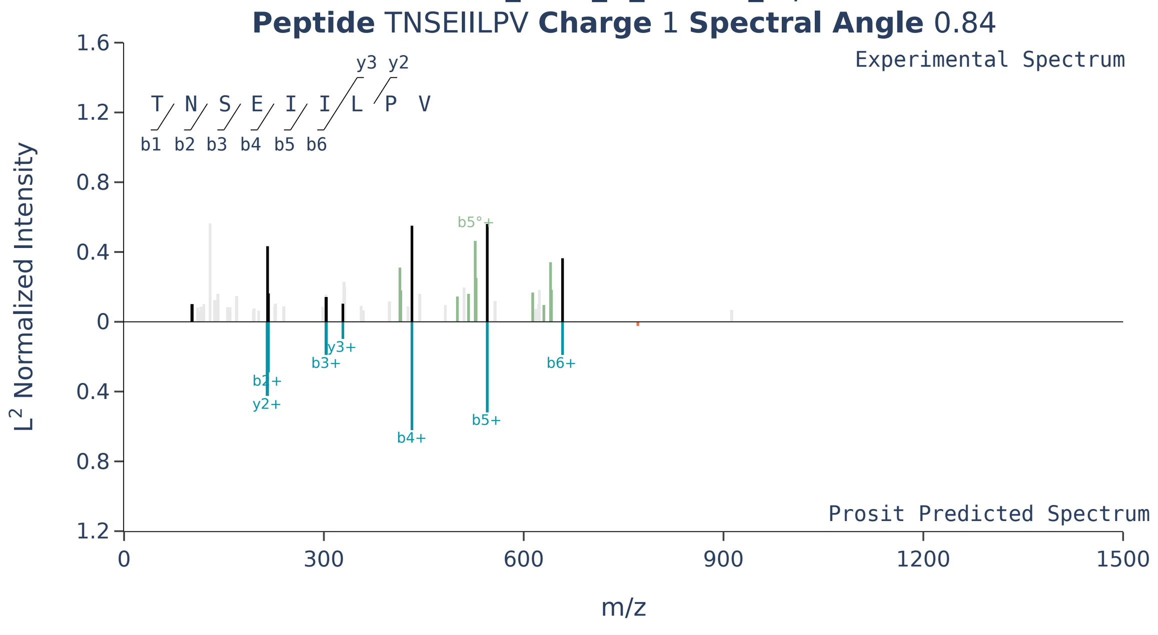

Source UnLabeled_HeLa_w_Listeria_rep1 **Scan** 33545
Peptide EVERPSLGV **Charge** 2 **Spectral Angle** 0.77

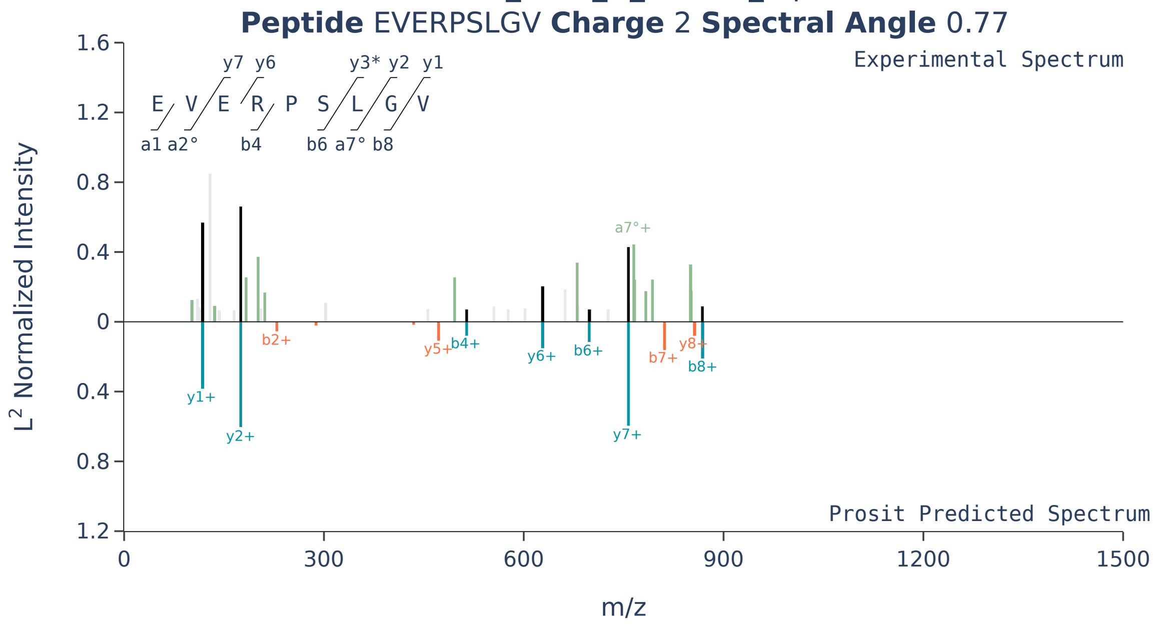

Source UnLabeled_HeLa_w_Listeria_rep1 **Scan** 42252
Peptide SVVGLIGNA **Charge** 1 **Spectral Angle** 0.82

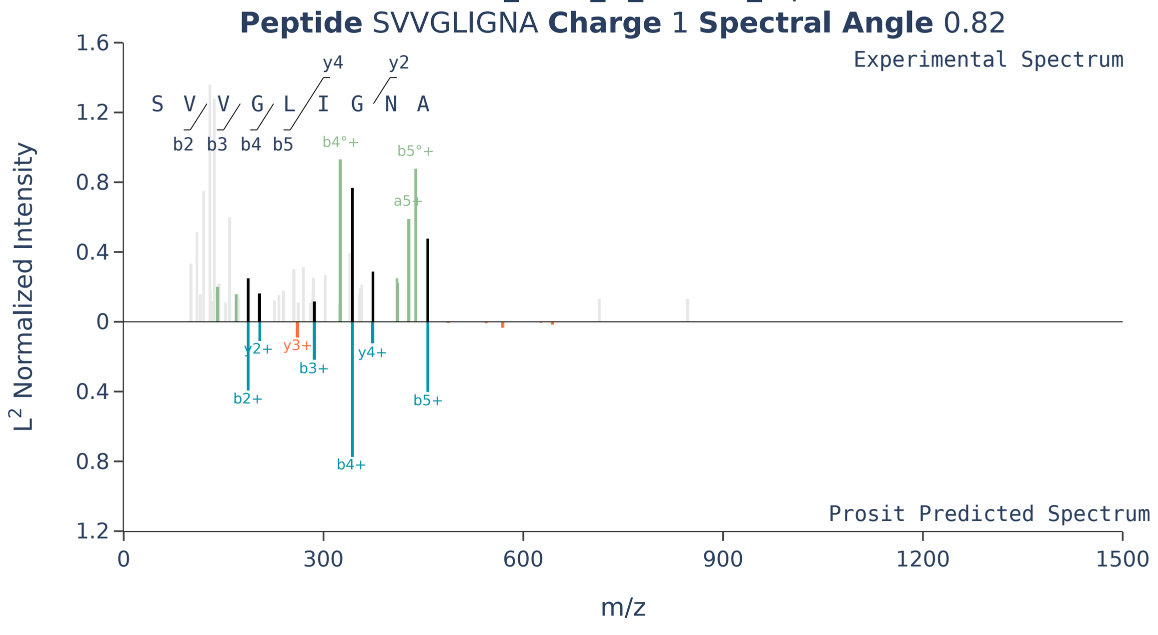

Source UnLabeled_HeLa_w_Listeria_rep2 **Scan** 60191
Peptide EIAIPTLFV **Charge** 1 **Spectral Angle** 0.87

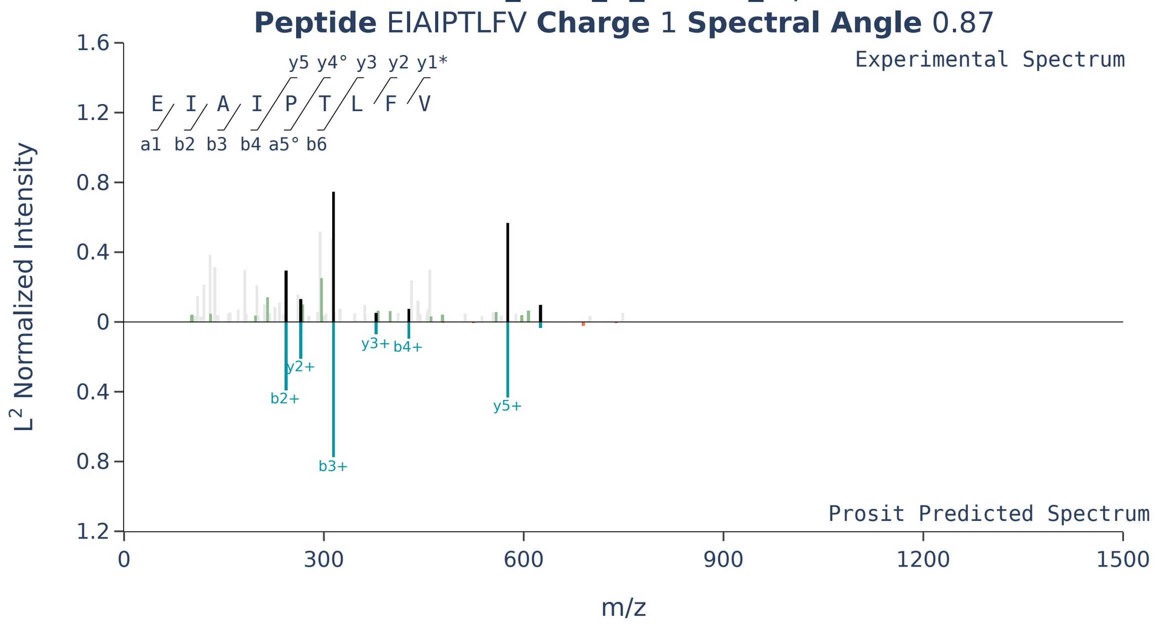

Source UnLabeled_HeLa_w_Listeria_rep2 **Scan** 46501
Peptide TVVAIAAGL **Charge** 1 **Spectral Angle** 0.89

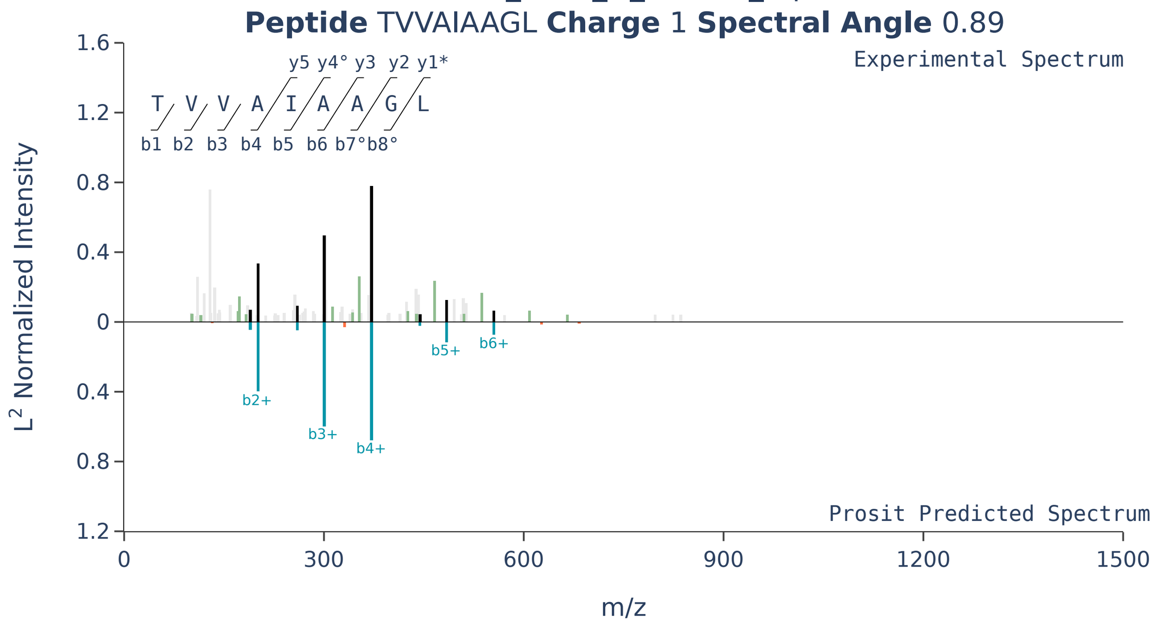

Source UnLabeled_HeLa_w_Listeria_rep1 **Scan** 38749
Peptide HLPEFTNEV **Charge** 2 **Spectral Angle** 0.84

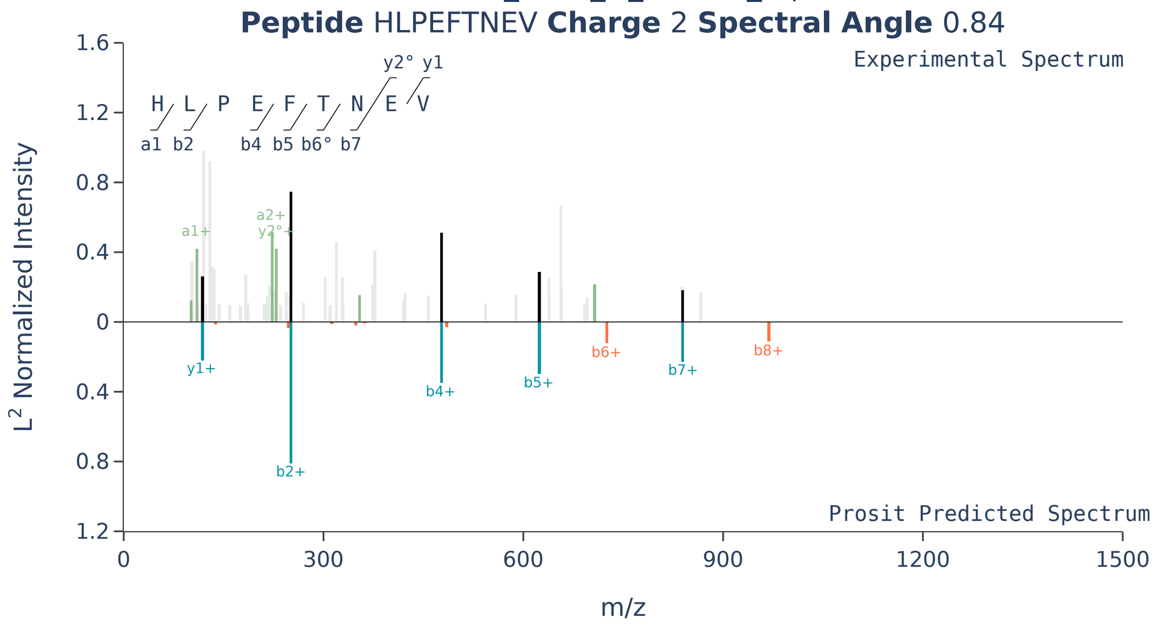

Source UnLabeled_HeLa_w_Listeria_rep2 **Scan** 42047
Peptide SINMPSLPV **Charge** 1 **Spectral Angle** 0.78

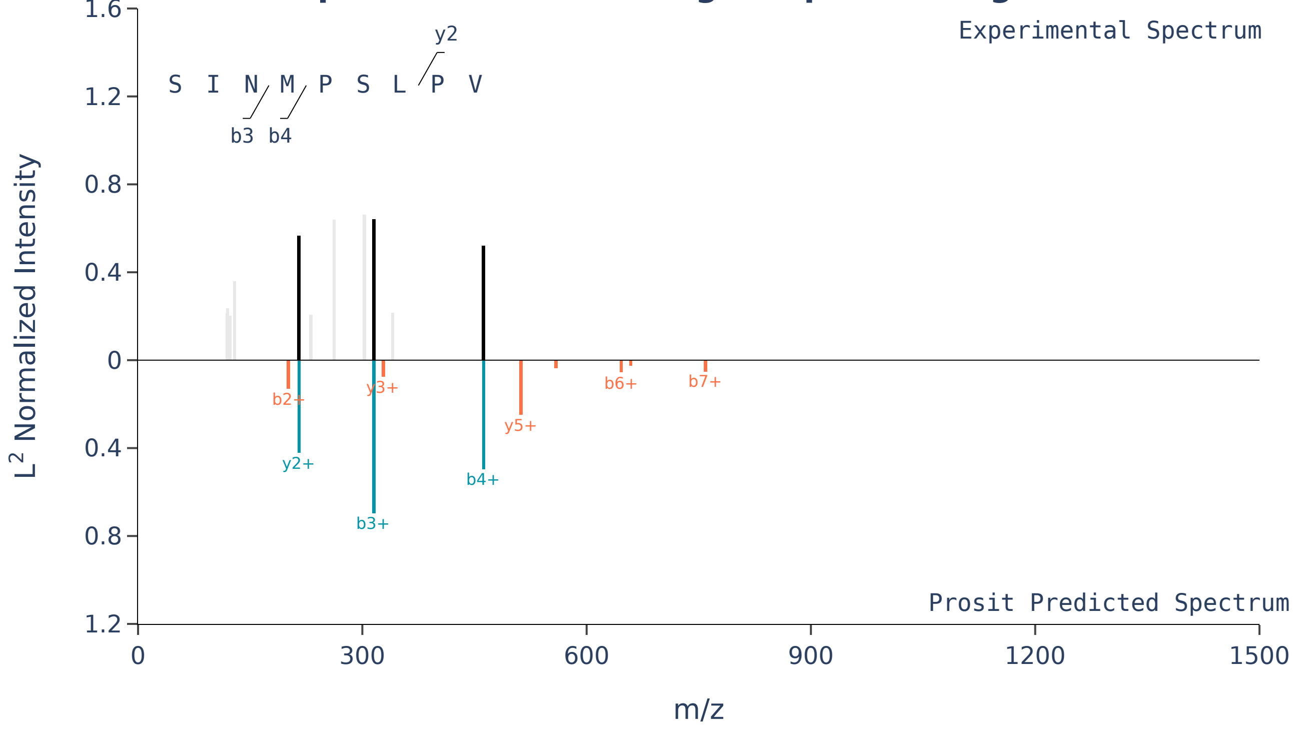

Source UnLabeled_HeLa_w_Listeria_rep4 **Scan** 38636
Peptide KIPPANIQTF **Charge** 2 **Spectral Angle** 0.68

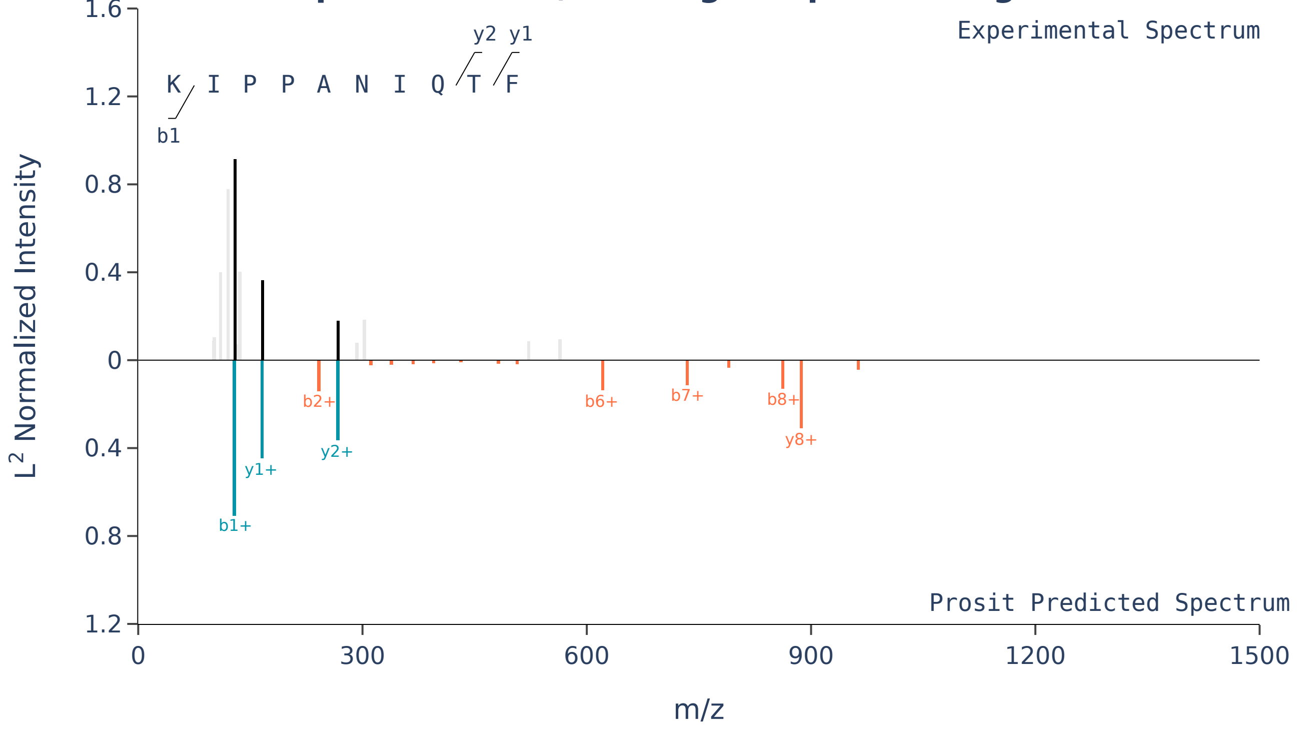

Source UnLabeled_HeLa_w_Listeria_rep1 **Scan** 41428
Peptide YKLGLAIHY **Charge** 2 **Spectral Angle** 0.84

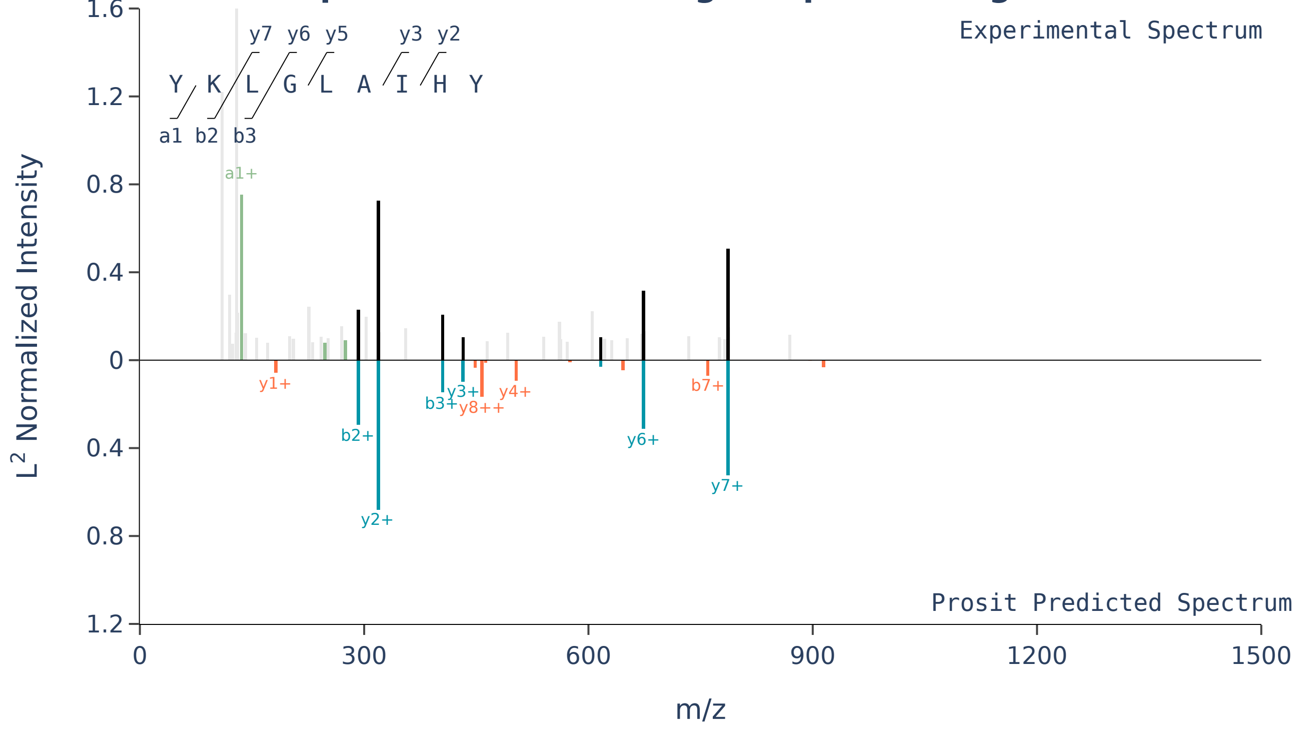

Source UnLabeled_HeLa_w_Listeria_rep2 **Scan** 36589
Peptide KLNDLISRY **Charge** 2 **Spectral Angle** 0.73

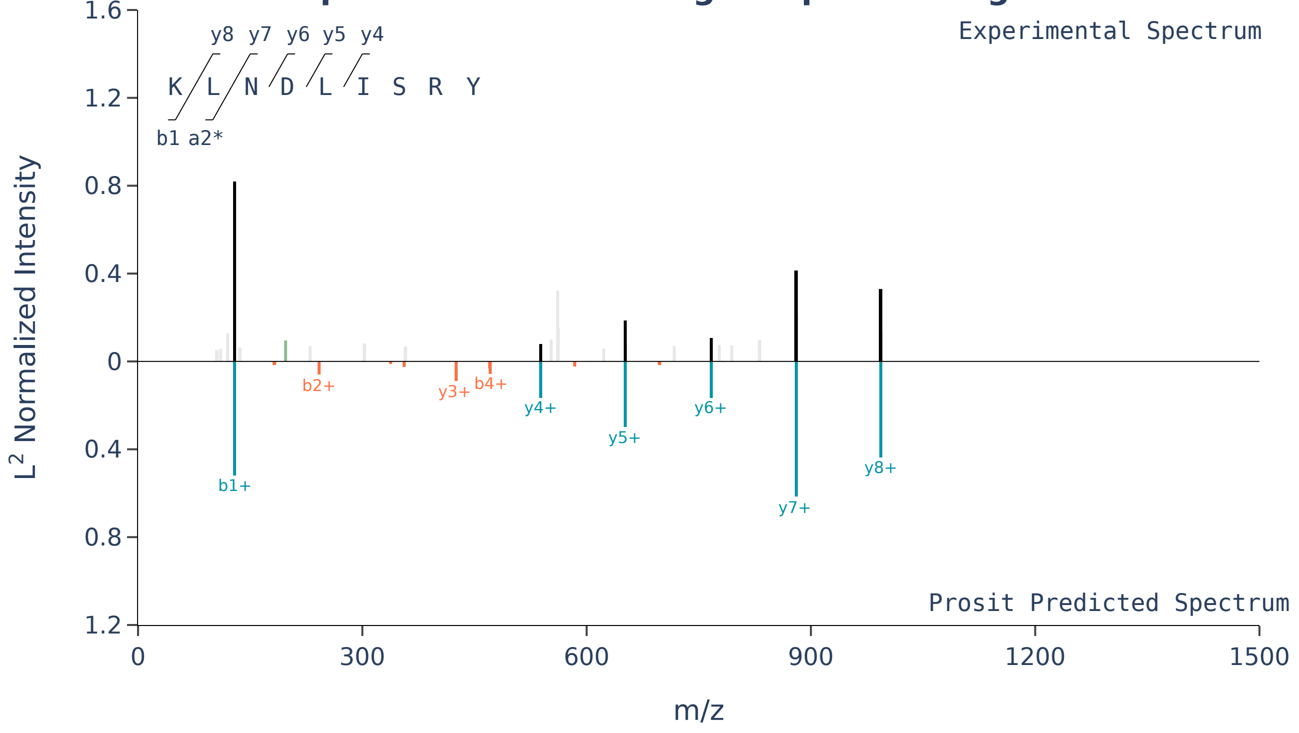

inSPIRE Spectral Plotting for listeria-mouse-240117

Experimental Spectrum Colour Code:

- Experimental peak matched to a Prosit predicted peak.
- Possible ion unknown to Prosit.
- Precursor matched peak.
- Experimental peak not matched to any potential ion.

Prosit Spectrum Colour Code:

- Prosit predicted peak matched to experimental spectrum.
- Prosit predicted peak not matched to experimental spectrum.

Additional Notes:

° indicates an ion with loss of H_2O.

* indicates an ion with loss of NH_3.

Loading [MathJax]/extensions/MathMenu.js

Source Lumos_A27_R2 **Scan** 13925
Peptide VSYKFSEYI **Charge** 2 **Spectral Angle** 0.96

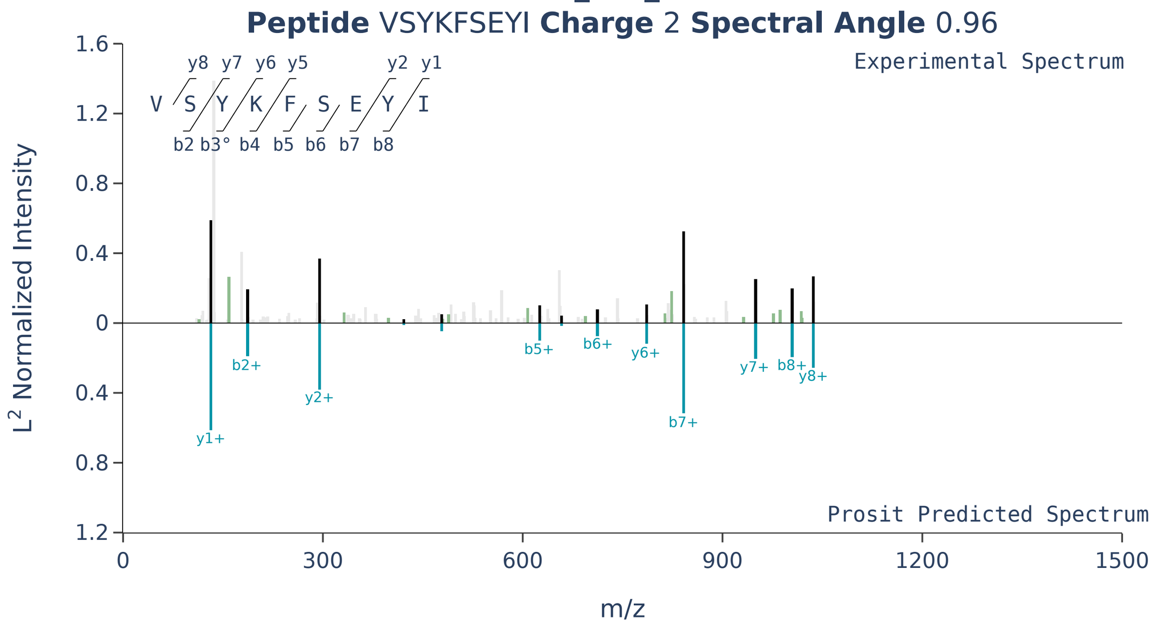

Source Lumos_A26_R2 **Scan** 10398
Peptide VGYGRVANF **Charge** 2 **Spectral Angle** 0.97

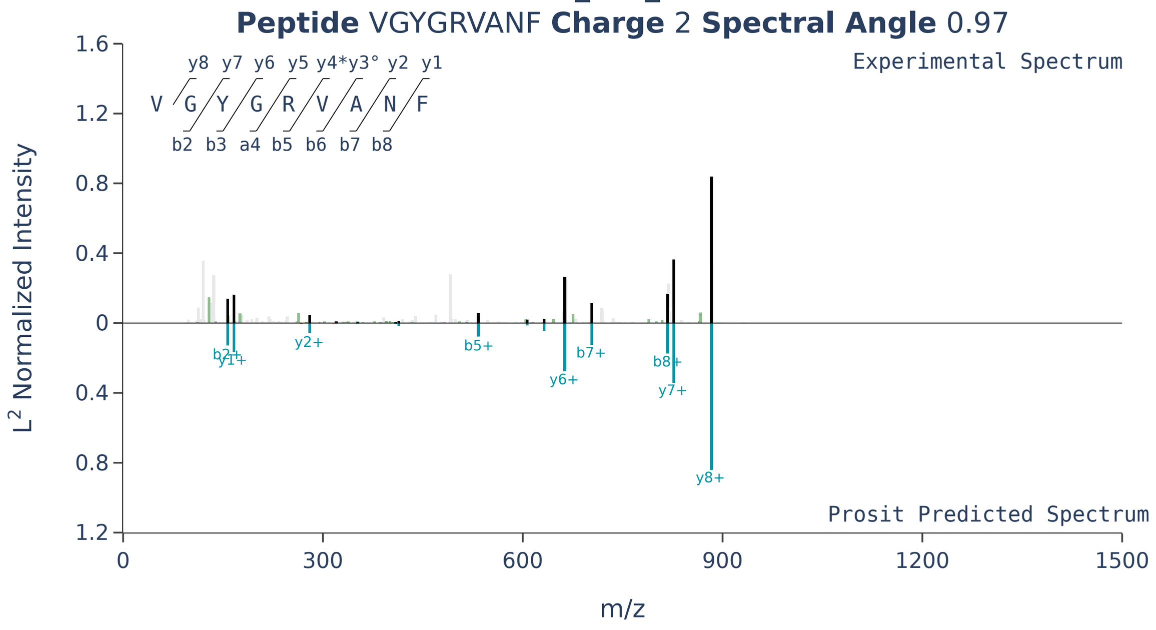

Source Lumos_A26_R2 **Scan** 11203
Peptide VAYGRQVYL **Charge** 2 **Spectral Angle** 0.93

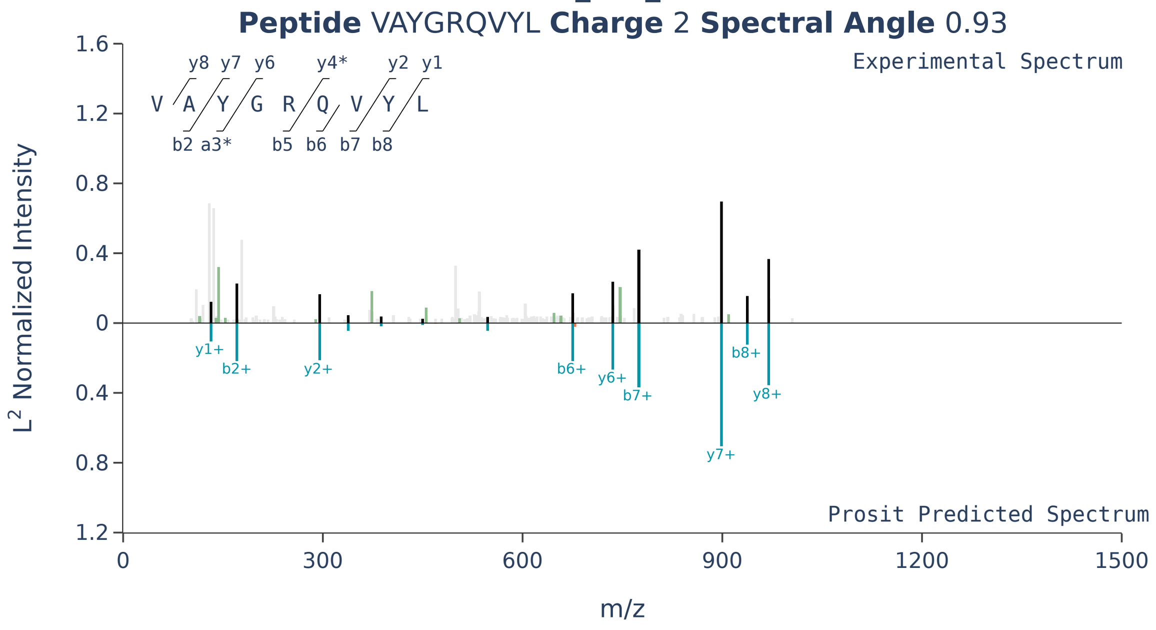

Source Lumos_A26_R2 **Scan** 17369
Peptide AILDFAGHNL **Charge** 2 **Spectral Angle** 0.93

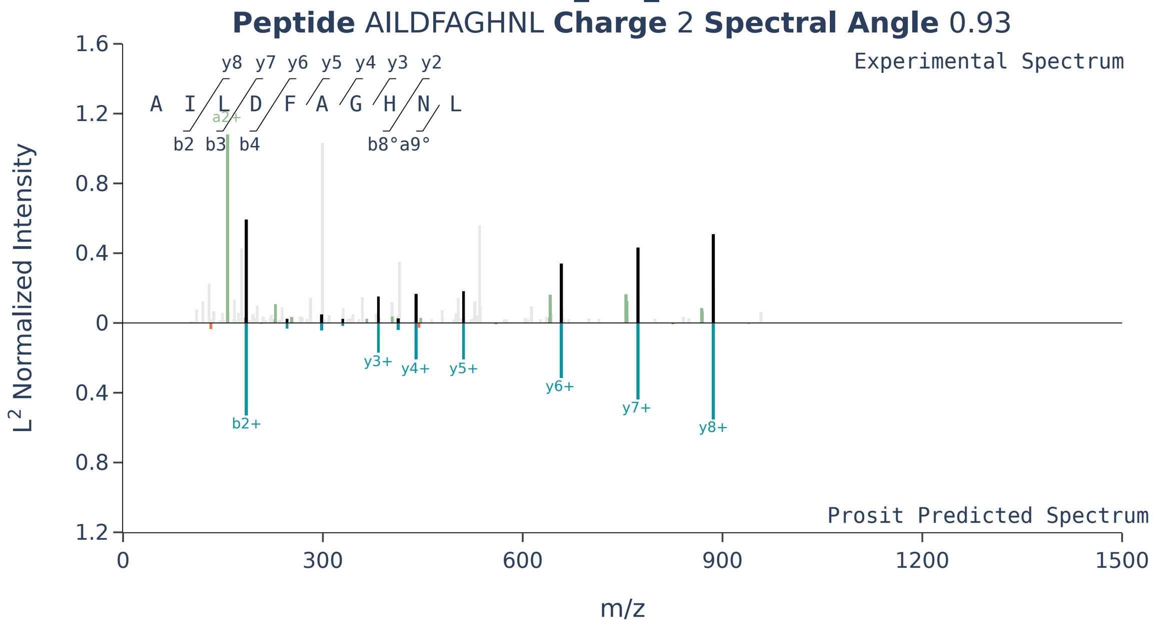

Source Lumos_A26_R1 **Scan** 13656
Peptide VSYTFSQPV **Charge** 1 **Spectral Angle** 0.87

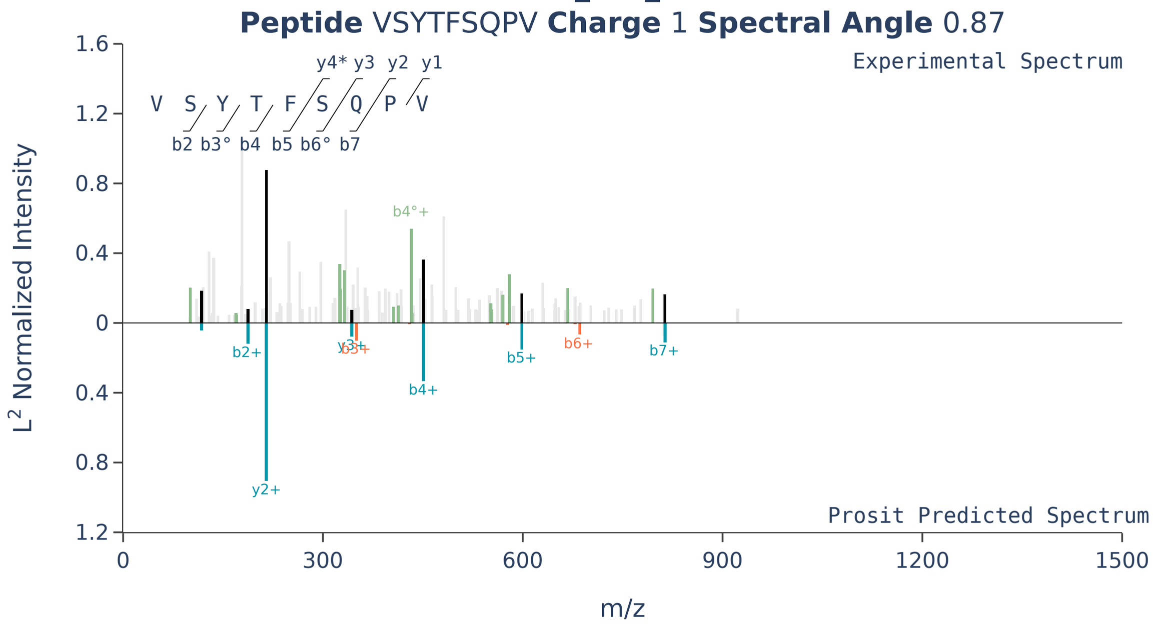

Source Lumos_A26_R2 **Scan** 9378
Peptide SIAEFRKL **Charge** 2 **Spectral Angle** 0.95

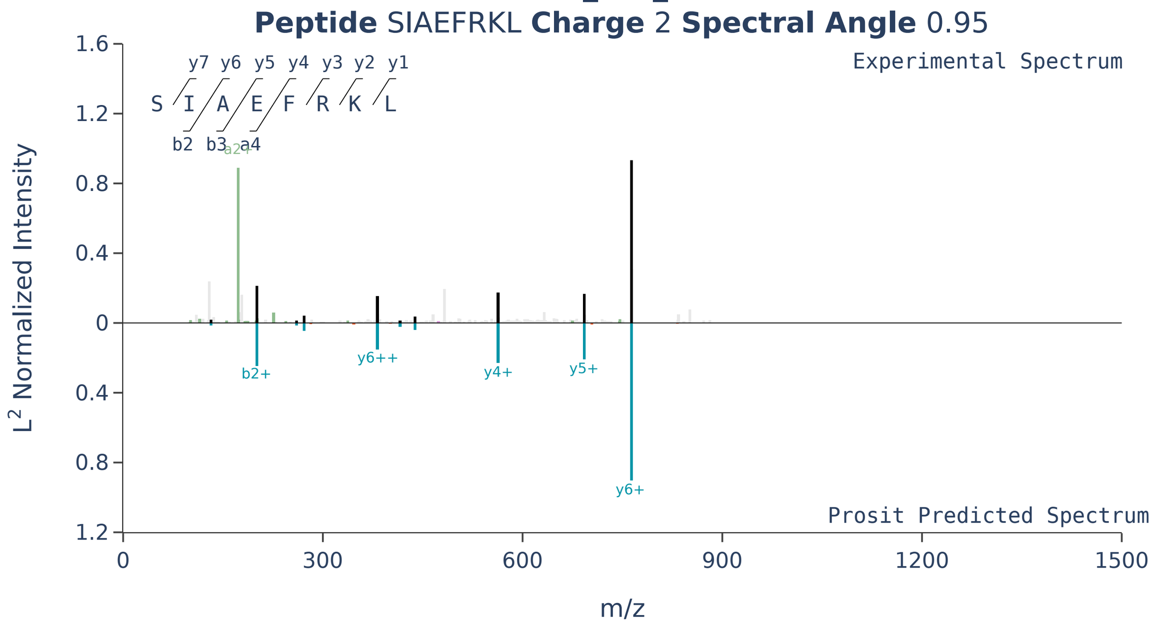

Source Lumos_A26_R2 **Scan** 13424
Peptide VNINRAIQL **Charge** 2 **Spectral Angle** 0.93

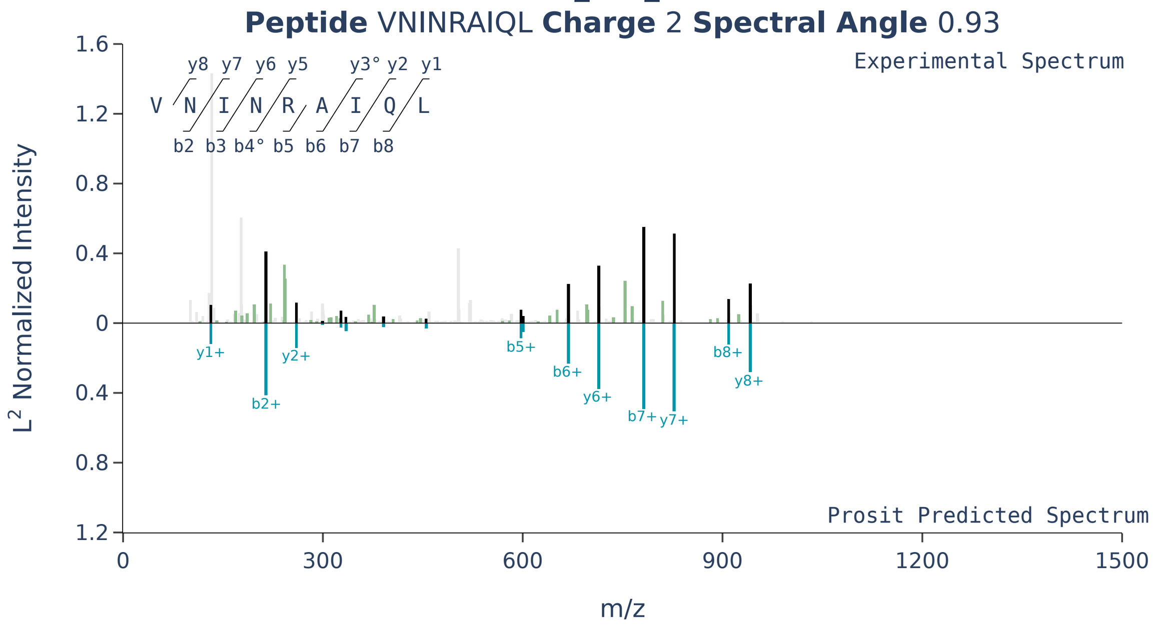

Source Lumos_A26_R1 **Scan** 11710
Peptide SNVAFAKL **Charge** 2 **Spectral Angle** 0.94

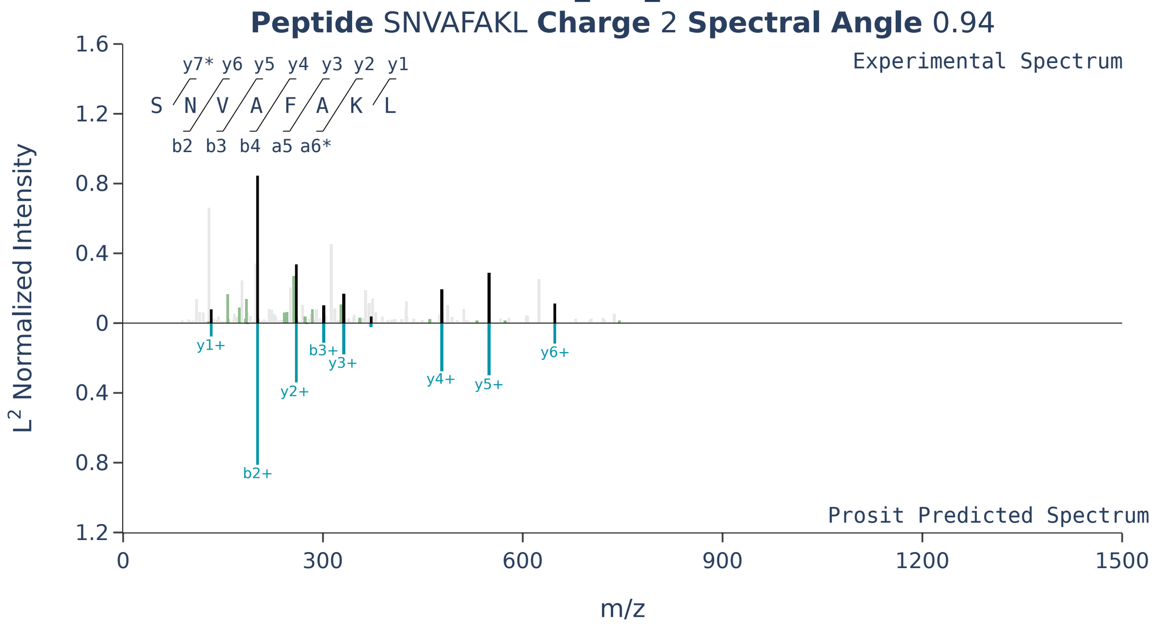

Source Lumos_A26_R2 **Scan** 19213
Peptide VIYIHLESF **Charge** 2 **Spectral Angle** 0.86

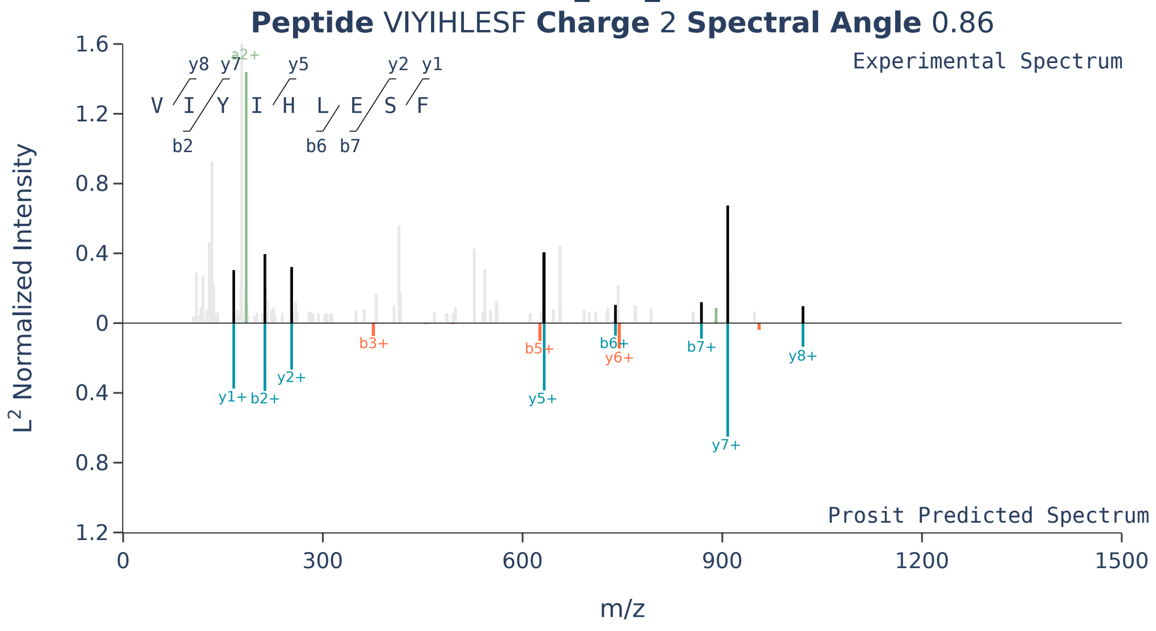

Source Lumos_A26_R1 **Scan** 12928
Peptide VIVDYRGL **Charge** 2 **Spectral Angle** 0.96

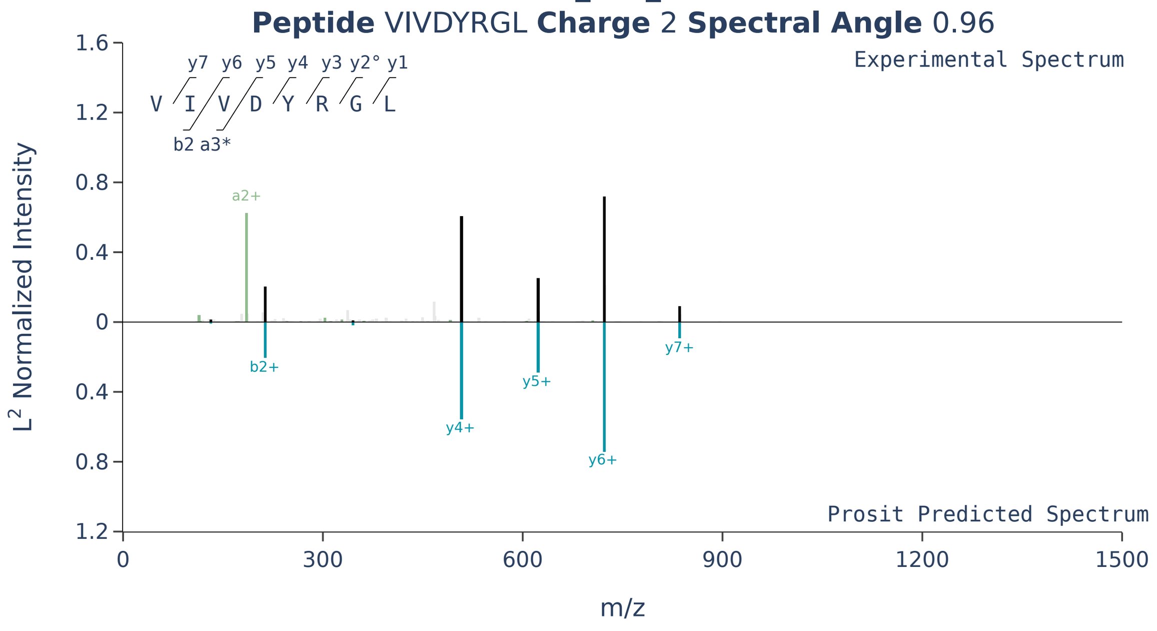

Source Lumos_A27_R1 **Scan** 12131
Peptide KNVIYIHL **Charge** 2 **Spectral Angle** 0.94

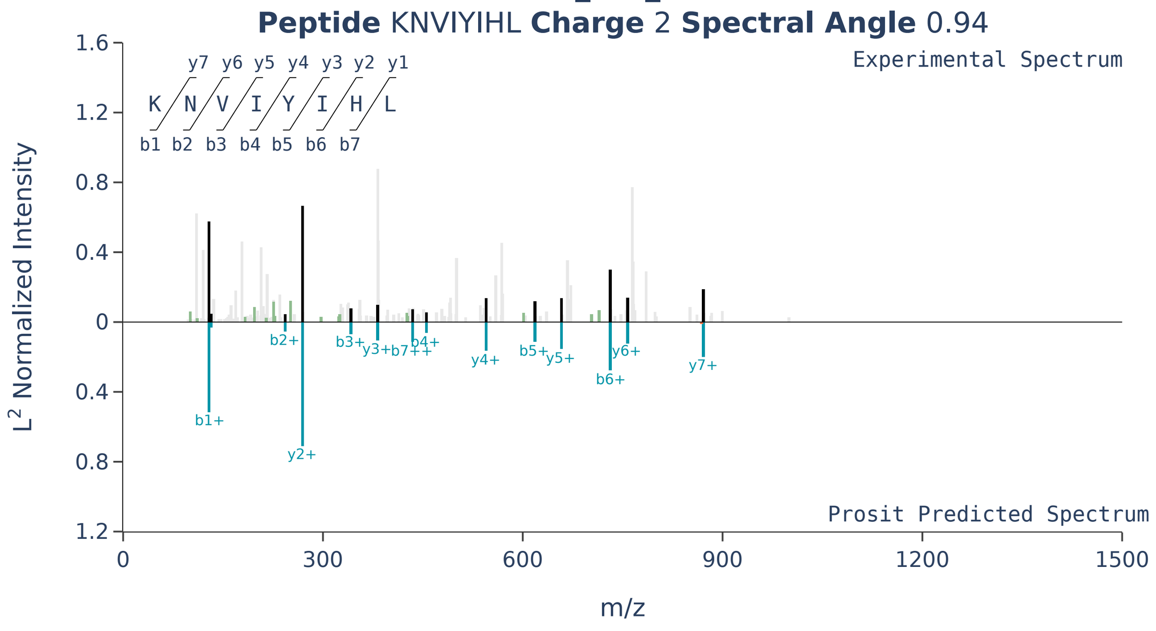

Source Lumos_A26_R1 **Scan** 14844
Peptide VTYDFARL **Charge** 2 **Spectral Angle** 0.64

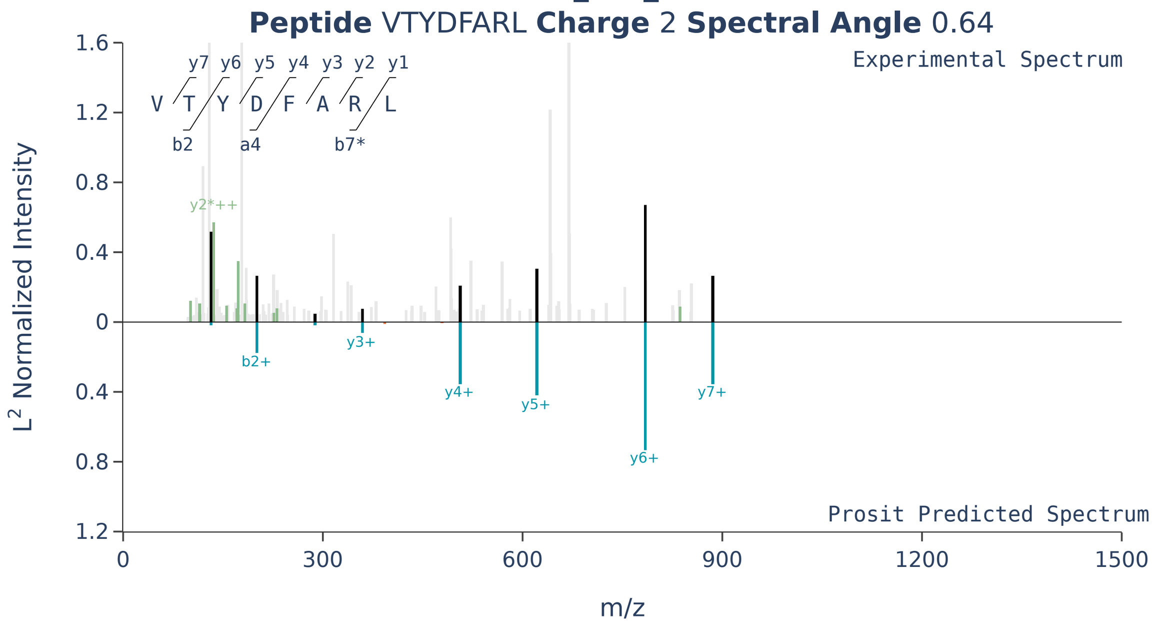

Source Lumos_A26_R1 **Scan** 14054
Peptide SYKFSEYI **Charge** 2 **Spectral Angle** 0.93

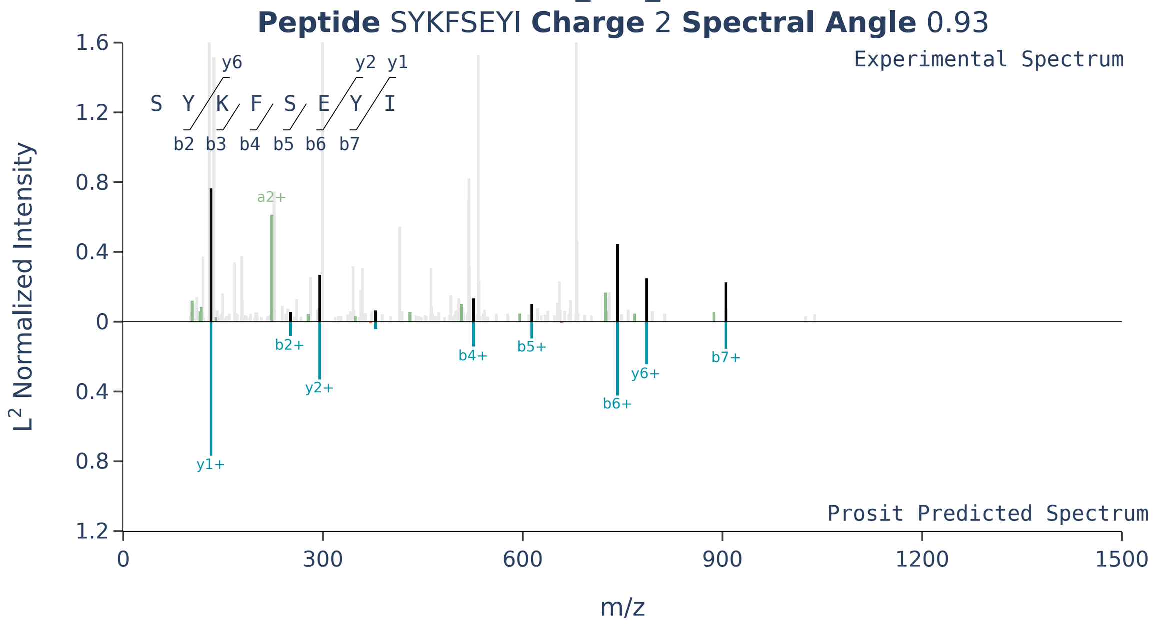

Source Lumos_A26_R1 **Scan** 2287
Peptide VNHMRANLM **Charge** 2 **Spectral Angle** 0.75

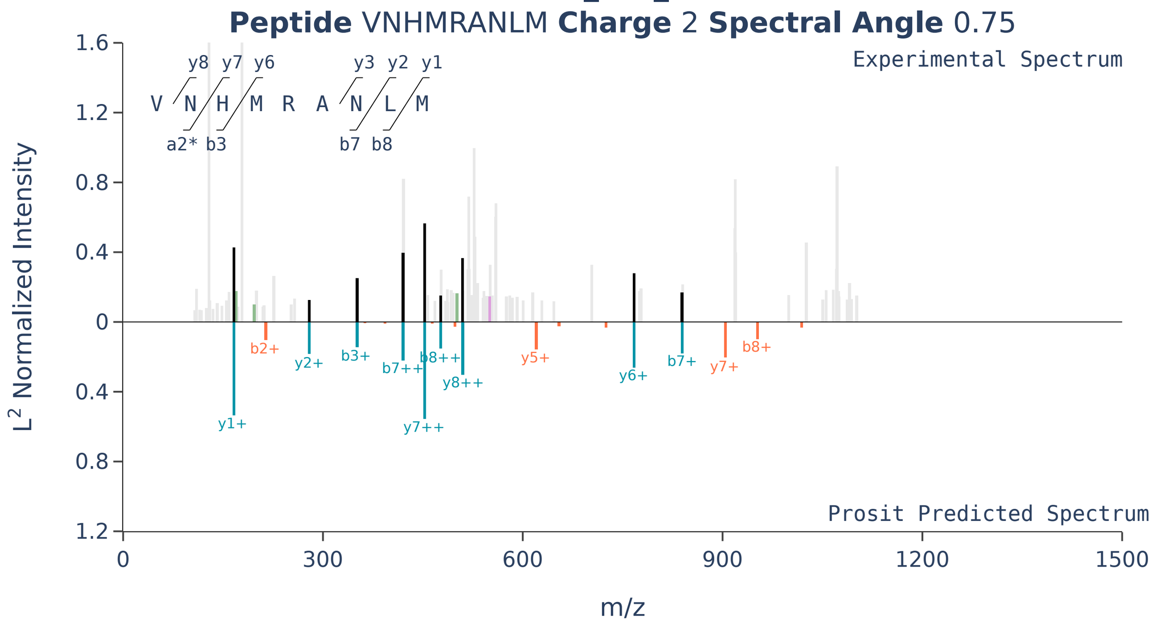

Source Lumos_A26_R1 **Scan** 14681
Peptide AGLKNLDVL **Charge** 2 **Spectral Angle** 0.96

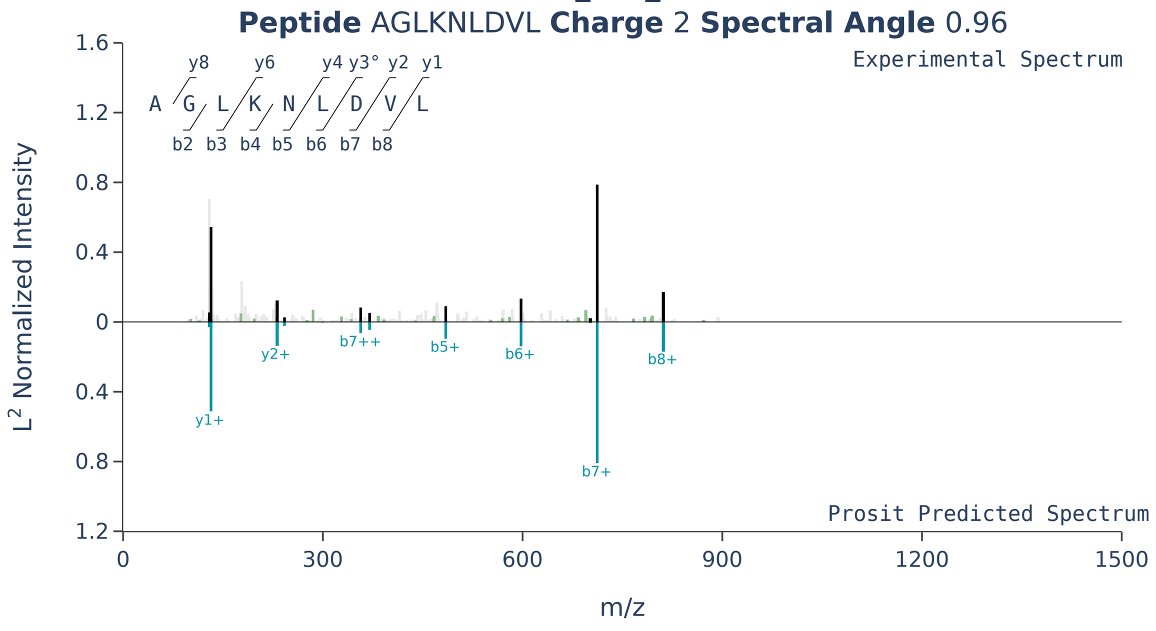

Source Lumos_A26_R1 **Scan** 15244
Peptide KNLTYLTL **Charge** 2 **Spectral Angle** 0.85

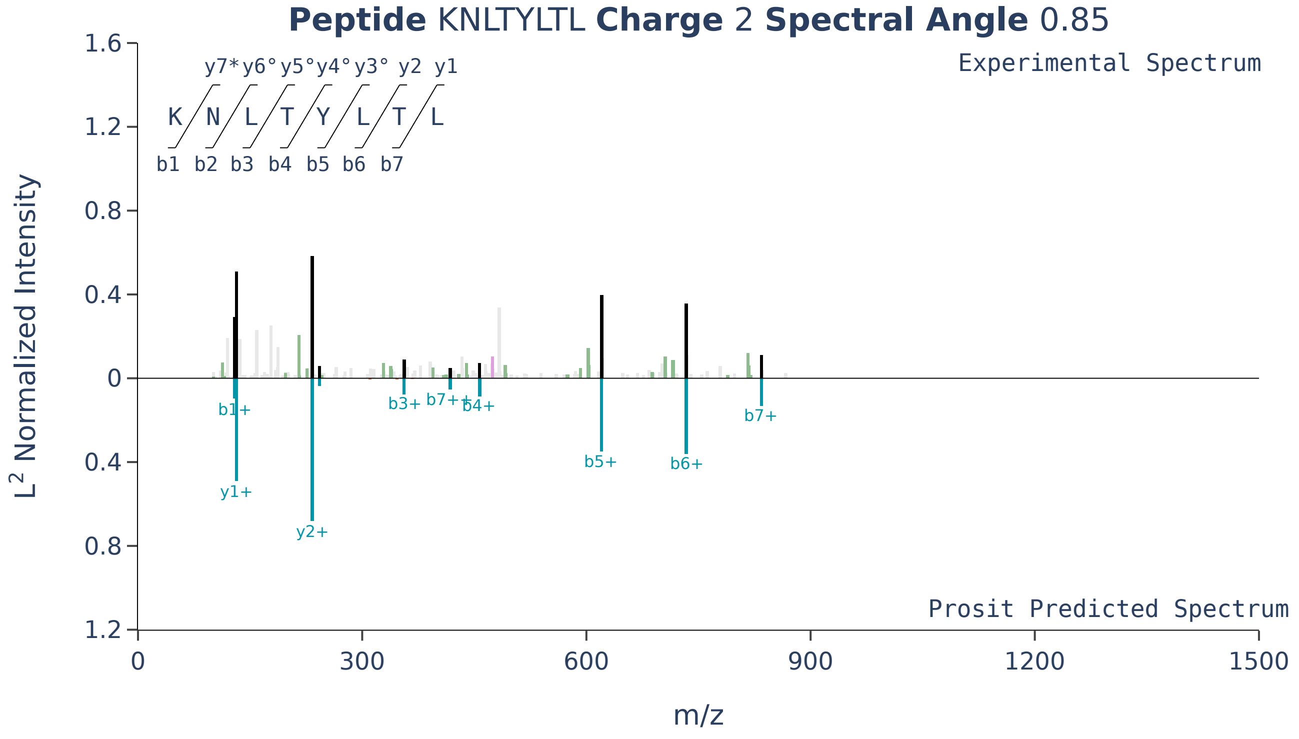

Source Lumos_A26_R2 **Scan** 7343
Peptide TSYAFRAA **Charge** 2 **Spectral Angle** 0.79

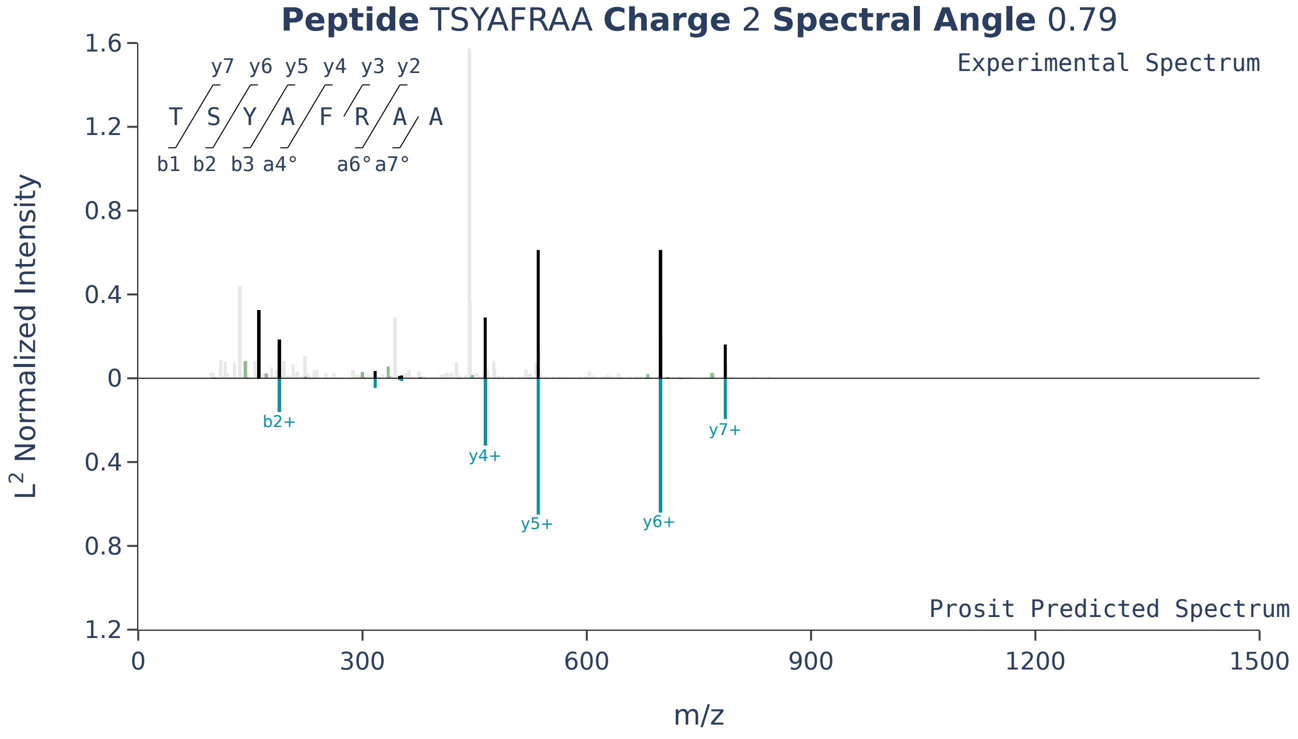

Source Lumos_A26_R2 **Scan** 16044
Peptide VIIEVARL **Charge** 2 **Spectral Angle** 0.92

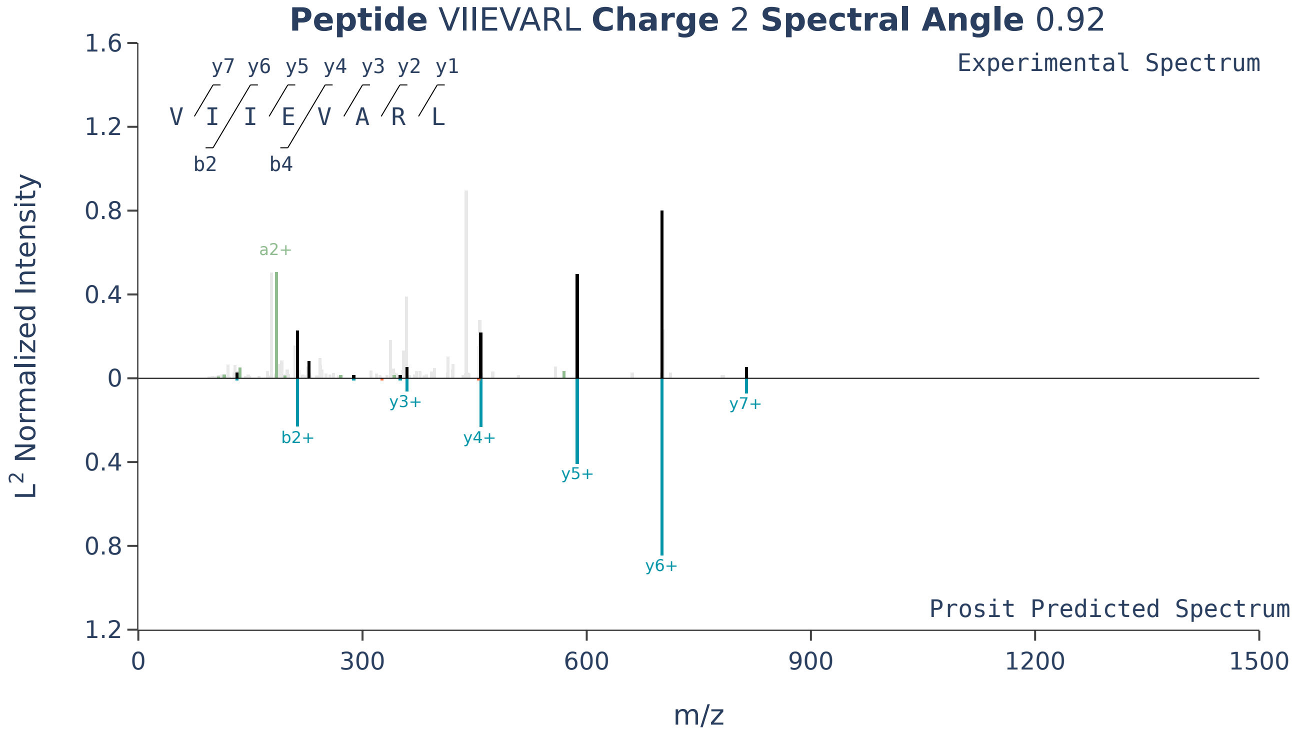

Source Lumos_A26_R2 **Scan** 10694
Peptide TAPKLAEL **Charge** 2 **Spectral Angle** 0.91

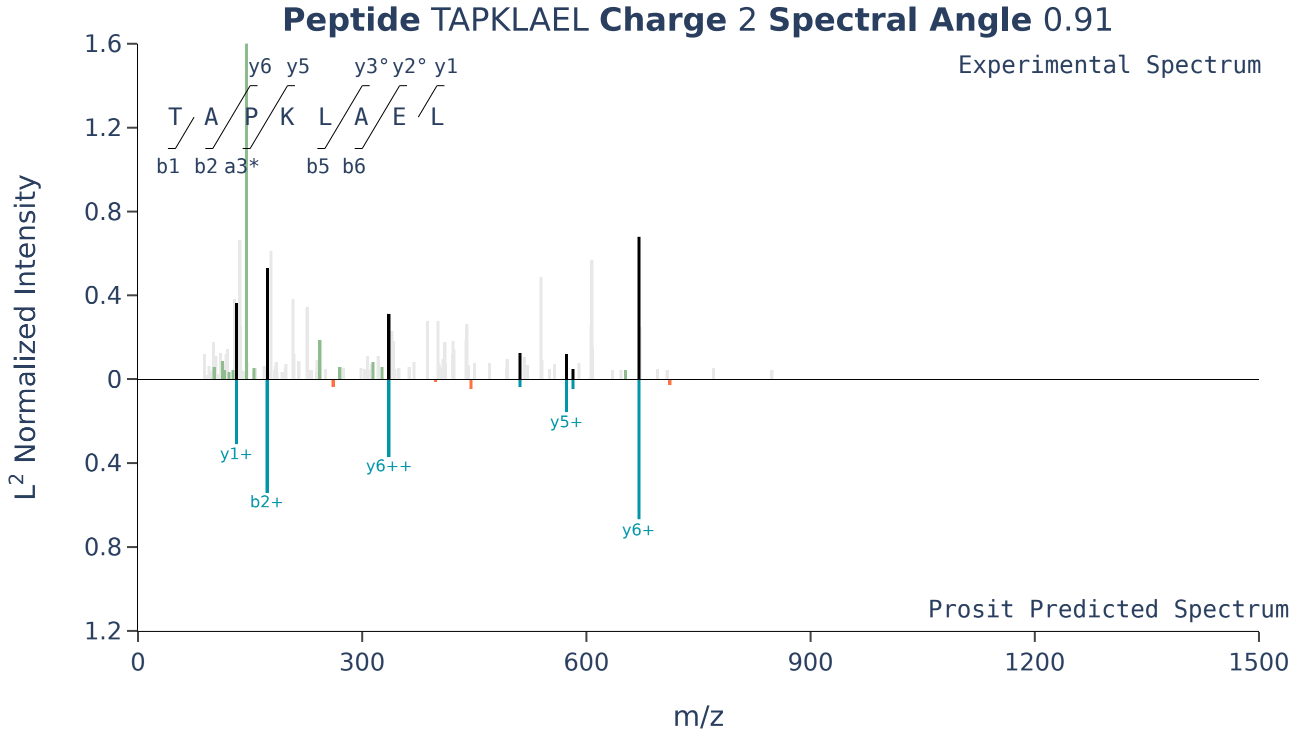

Source Lumos_A26_R2 **Scan** 14278
Peptide VTVSLPRV **Charge** 2 **Spectral Angle** 0.93

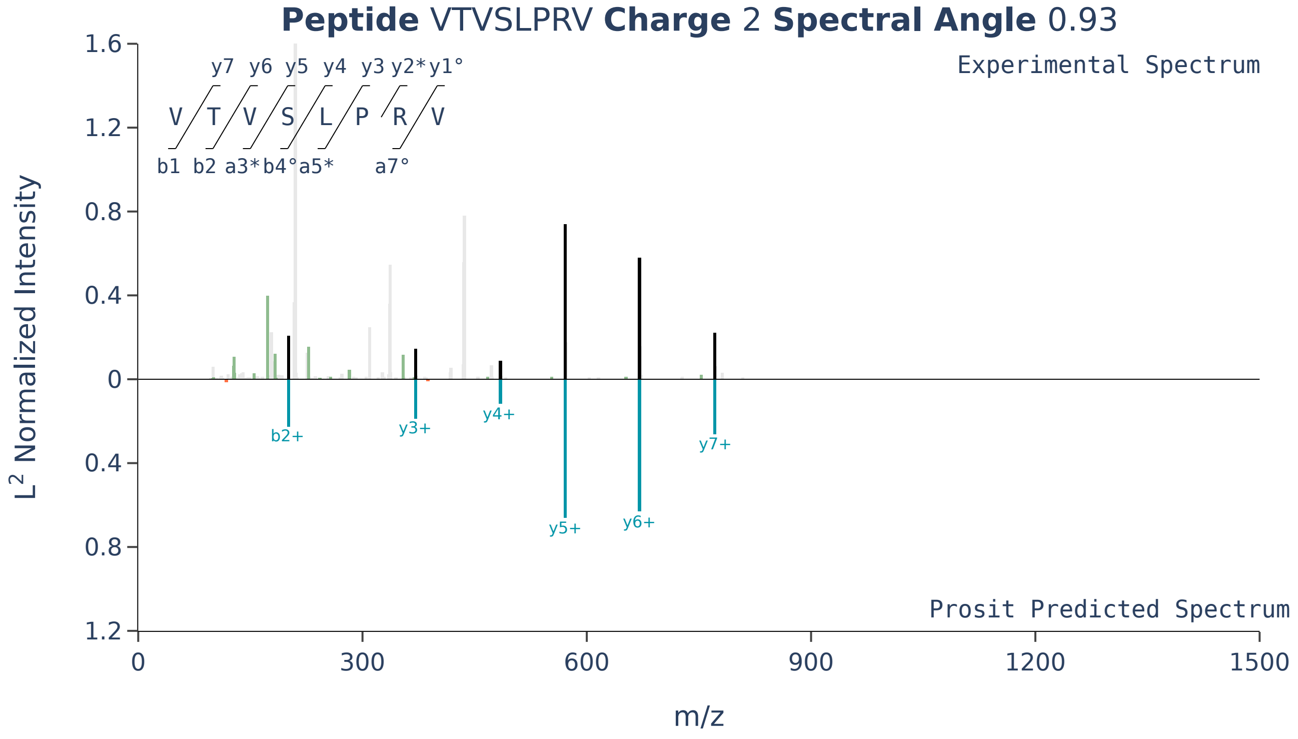

Source Lumos_A26_R1 **Scan** 11620
Peptide SLIHLKNL **Charge** 2 **Spectral Angle** 0.94

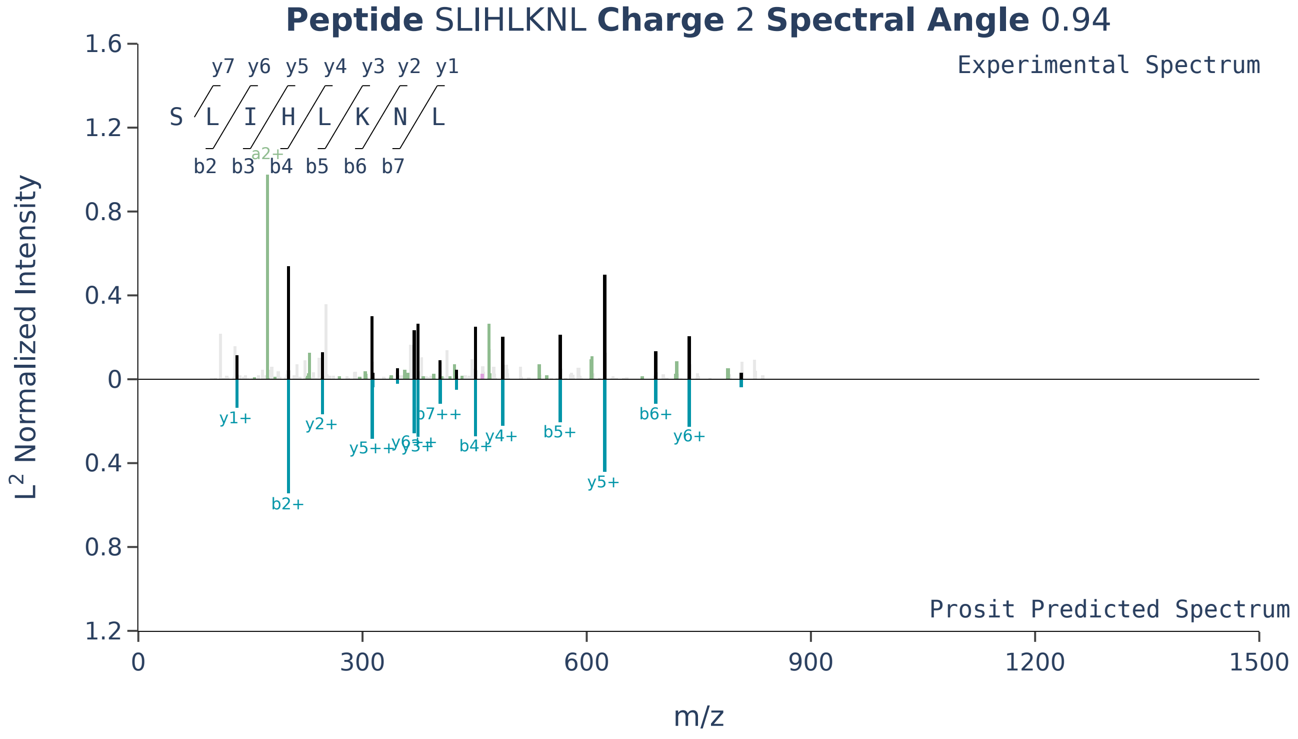

Source Lumos_A26_R2 **Scan** 3001
Peptide TGPRYETA **Charge** 2 **Spectral Angle** 0.86

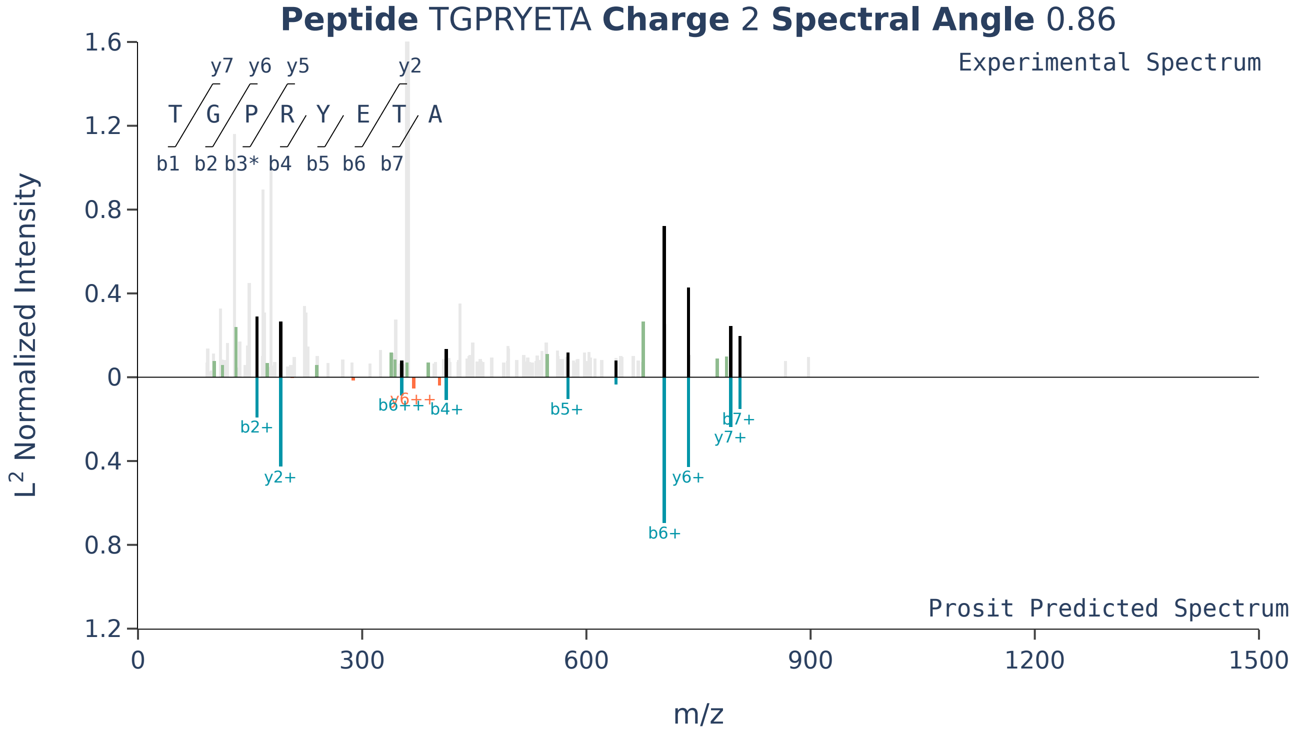

Source Lumos_A26_R1 **Scan** 17634
Peptide IIRAIVNL **Charge** 2 **Spectral Angle** 0.8

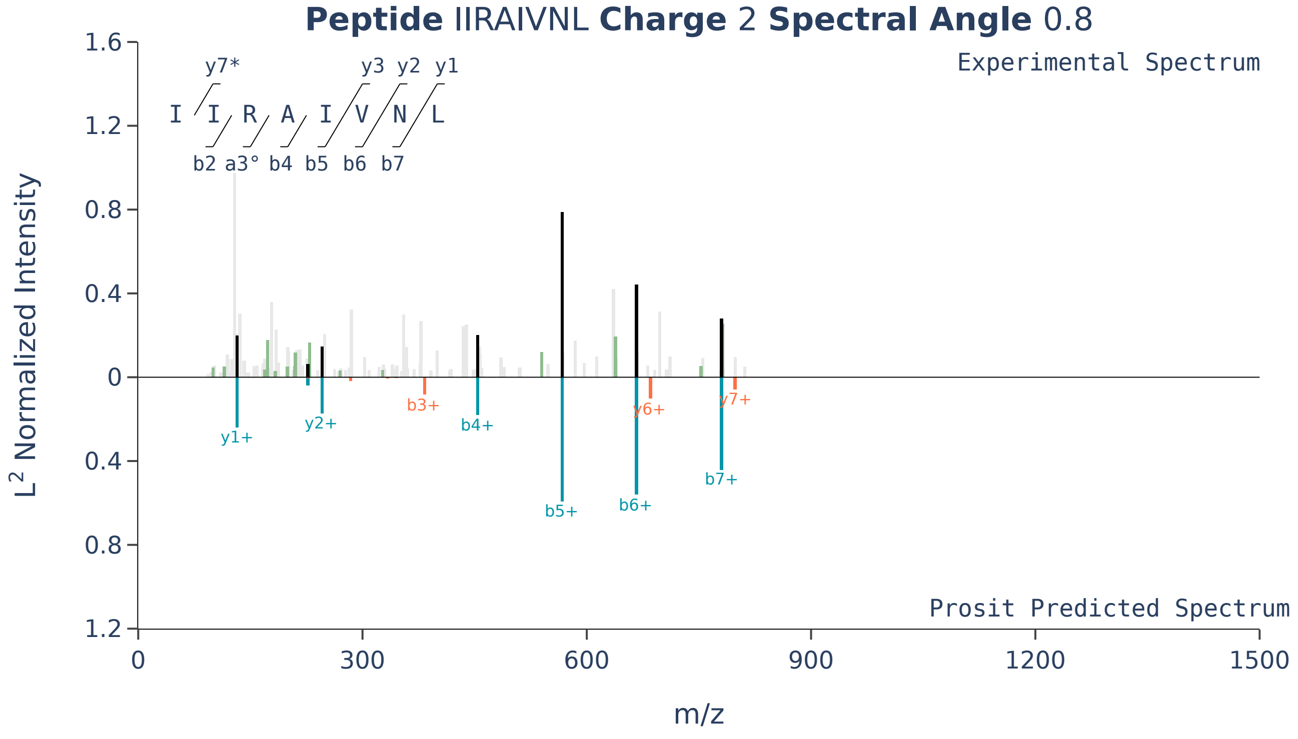

Supplement: File S3 [file EMS204118-supplement-File_S3.pdf]
