# Supplementary material for: PEPSeek-mediated identification of novel epitopes from viral and bacterial pathogens and the impact on host cell immunopeptidomes
Source: Mol Cell Proteomics. Author manuscript; Available in PMC 2025 Aug 8. (PMC12002930; doi:10.1016/j.mcpro.2025.100937)

File S4. Comparison between MS2 spectra of experimentally identified SARS-Cov2 derived peptides and MS2 spectra of the corresponding synthetic peptides. For all SARS-Cov2 derived peptides which were tested, synthetic peptides were ordered and measured via MS. Presented are the synthetic and experimental MS2 spectra for those 8 peptides. In this case, since the experimental spectra are taken for public datasets, different mass spectrometers and mass spectrometer settings were used between the experimental and the synthetic spectra, which leads to some variation in the measured intensities. We also compare both the experimental and synthetic MS2 spectra to the Prosit predicted MS2 spectrum for greater insight into the specific y- and b- ions present.

Experimental vs. Synthetic Colour Code:

- Matched peak between spectra. Possible y-, b-, or a-ion.
- Matched peak between spectra. Origin not clear.
- Peak not matched between spectra.

Prosit Comparison Colour Code:

- Experimental peak matched to a Prosit predicted peak.
- Experimental peak not matched to a Prosit predicted peak.
- Prosit predicted peak matched to experimental spectrum.
- Prosit predicted peak not matched to experimental spectrum.

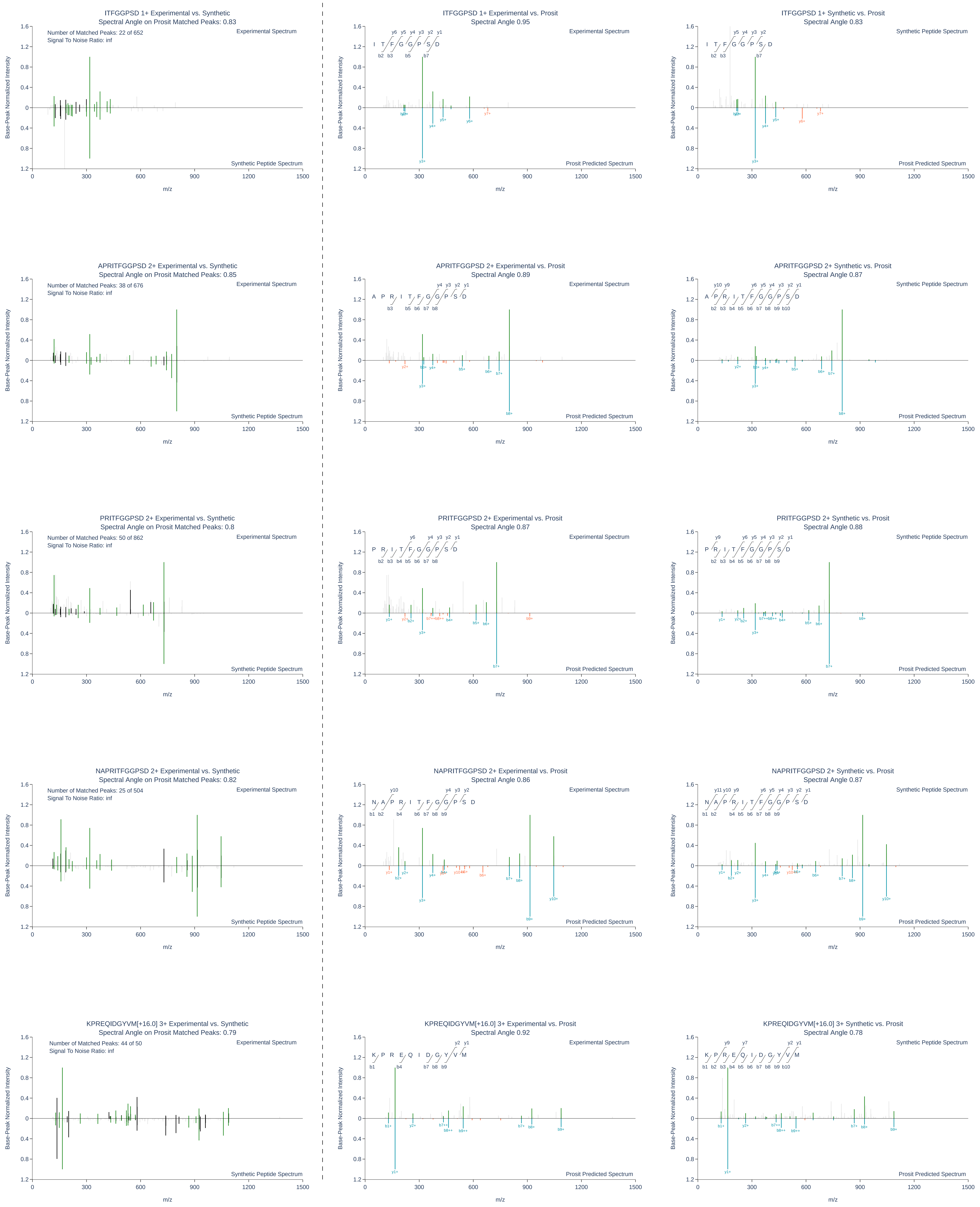

Experimental vs. Synthetic Colour Code:

- Matched peak between spectra. Possible y-, b-, or a-ion.
- Matched peak between spectra. Origin not clear.
- Peak not matched between spectra.

Prosit Comparison Colour Code:

- Experimental peak matched to a Prosit predicted peak.
- Experimental peak not matched to a Prosit predicted peak.
- Prosit predicted peak matched to experimental spectrum.
- Prosit predicted peak not matched to experimental spectrum.

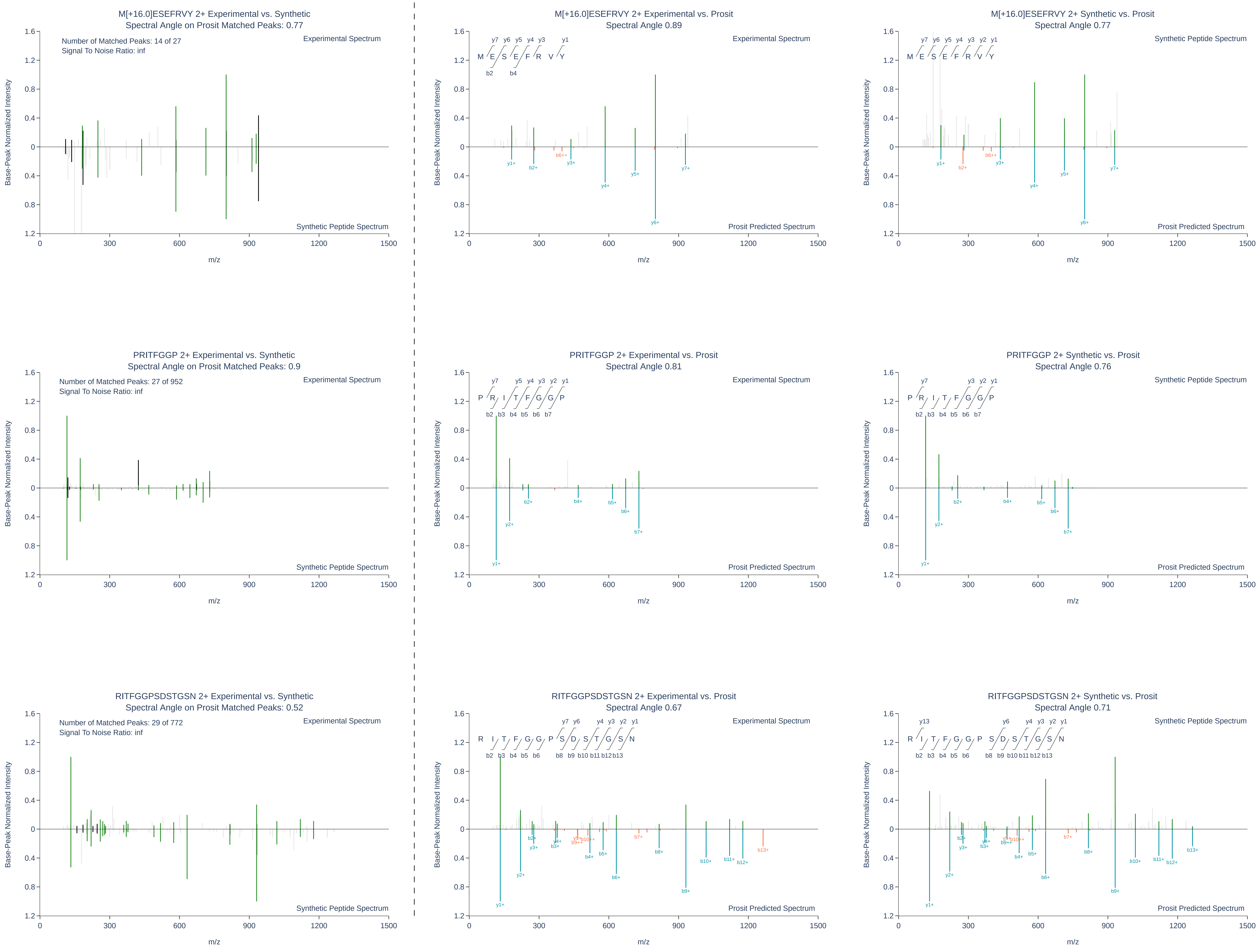

Supplement: File S4 [file EMS204118-supplement-File_S4.pdf]
