# Supplementary material for: PEPSeek-mediated identification of novel epitopes from viral and bacterial pathogens and the impact on host cell immunopeptidomes
Source: Mol Cell Proteomics. Author manuscript; Available in PMC 2025 Aug 8. (PMC12002930; doi:10.1016/j.mcpro.2025.100937)

File S6. Comparison between MS2 spectra of experimentally identified *L. monocytogenes*-derived peptides and MS2 spectra of the corresponding synthetic peptides. For all *L. monocytogenes*-derived epitope candidates, synthetic peptides were ordered and measured via MS. Of these 23 peptides, 18 were detected by a PEAKS DB search indicating successful synthesis. Presented are the synthetic and experimental MS2 spectra for those 18 peptides. We also compare both the experimental and synthetic MS2 spectra to the Prosit predicted MS2 spectrum for greater insight into the specific y- and b- ions present.

Experimental vs. Synthetic Colour Code:

- Matched peak between spectra. Possible y-, b-, or a-ion.
- Matched peak between spectra. Origin not clear.
- Peak not matched between spectra.

Prosit Comparison Colour Code:

- Experimental peak matched to a Prosit predicted peak.
- Experimental peak not matched to a Prosit predicted peak.
- Prosit predicted peak matched to experimental spectrum.
- Prosit predicted peak not matched to experimental spectrum.

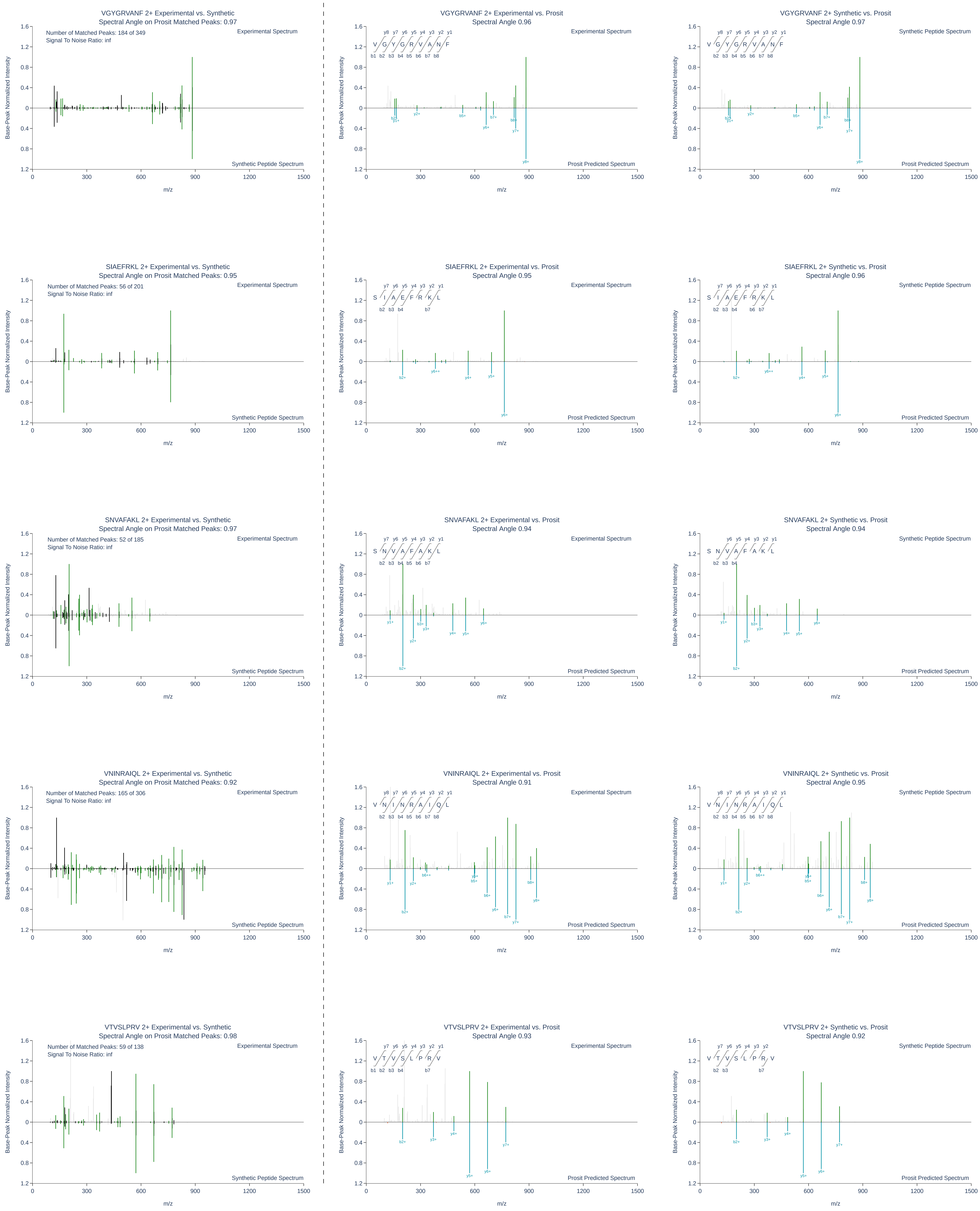

Experimental vs. Synthetic Colour Code:

- Matched peak between spectra. Possible y-, b-, or a-ion.
- Matched peak between spectra. Origin not clear.
- Peak not matched between spectra.

Prosit Comparison Colour Code:

- Experimental peak matched to a Prosit predicted peak.
- Experimental peak not matched to a Prosit predicted peak.
- Prosit predicted peak matched to experimental spectrum.
- Prosit predicted peak not matched to experimental spectrum.

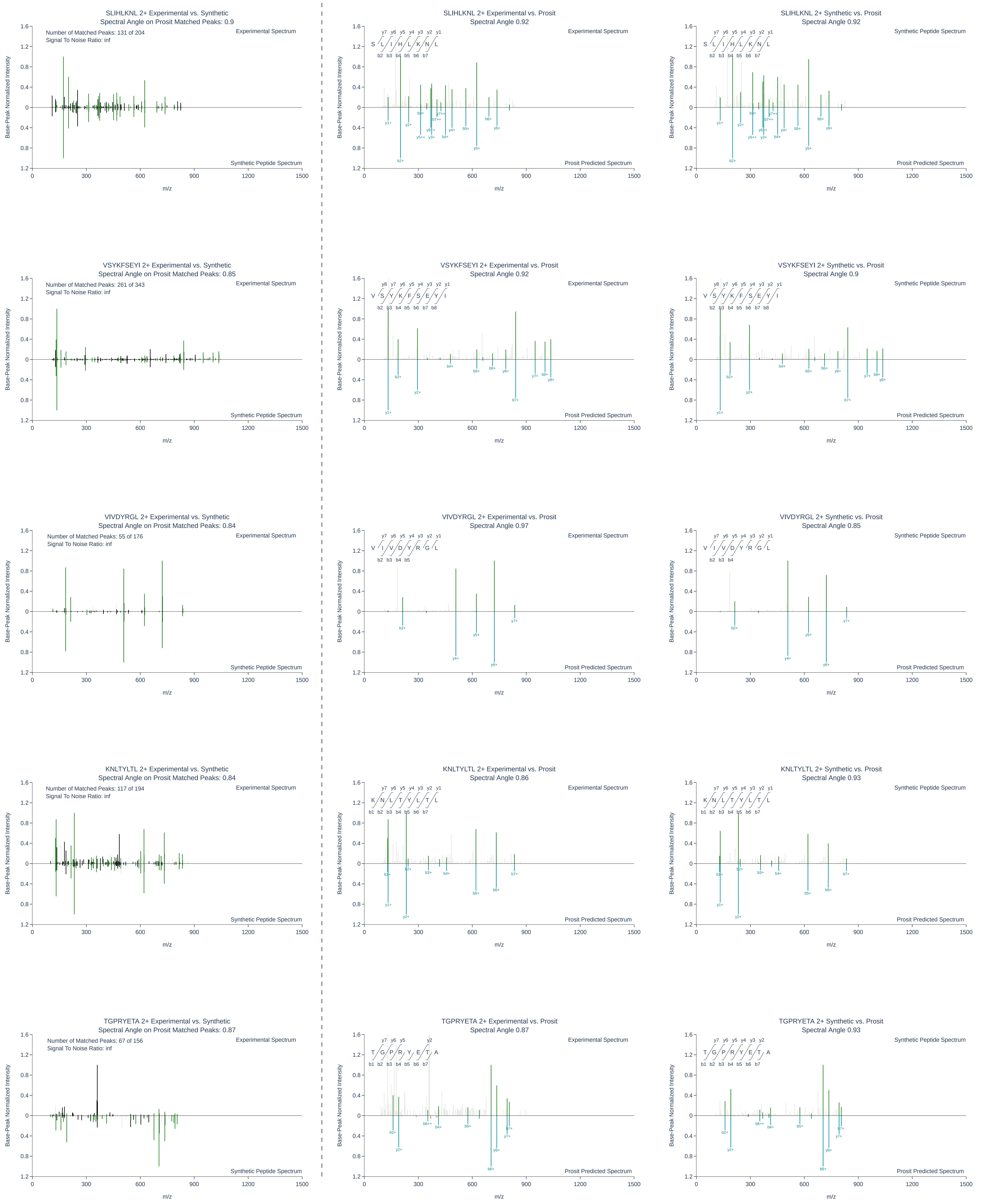

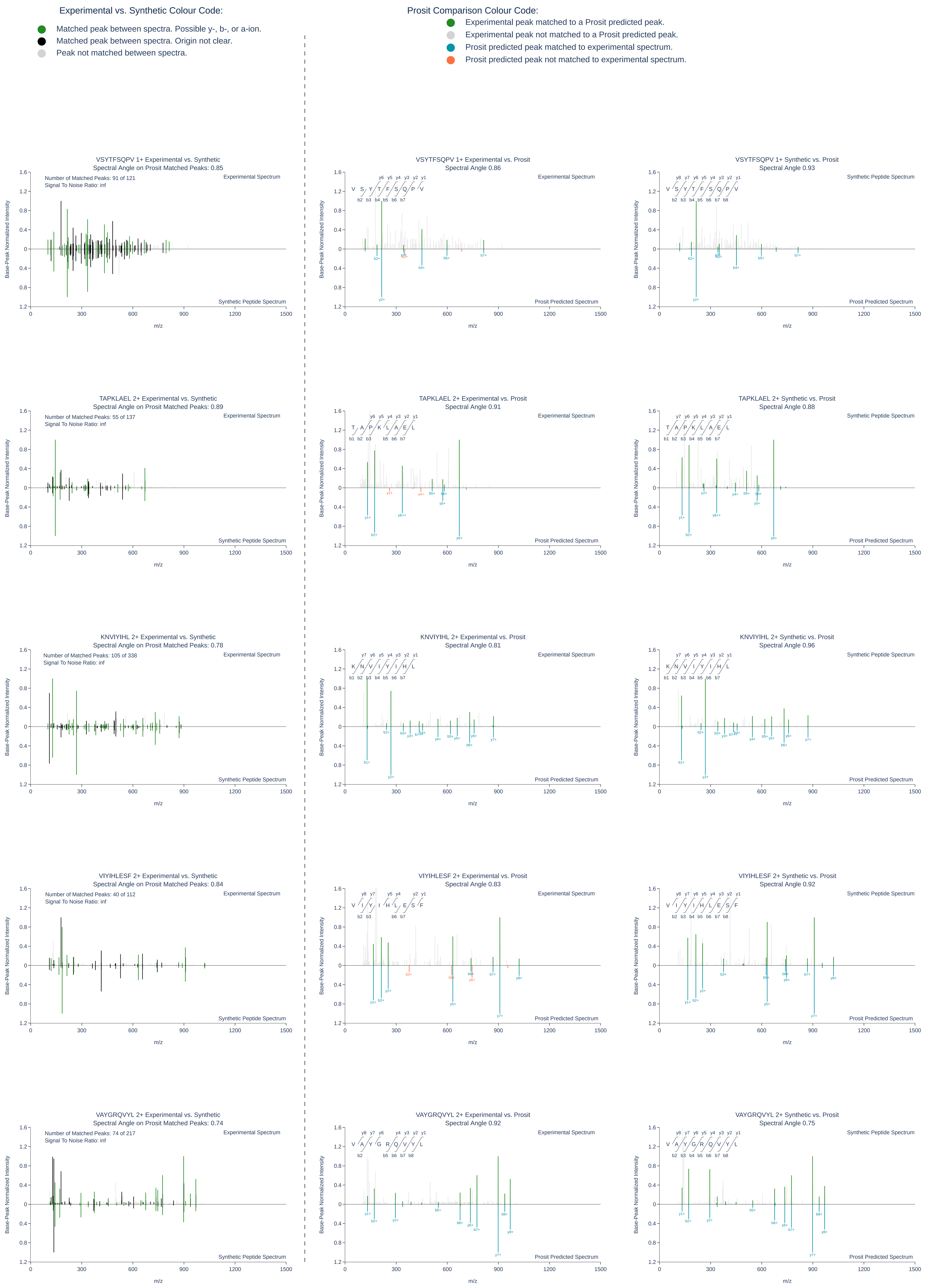
Experimental vs. Synthetic Colour Code:
Matched peak between spectra. Possible y-, b-, or a-ion.
Matched peak between spectra. Origin not clear.
Peak not matched between spectra.
Prosit Comparison Colour Code:
Experimental peak matched to a Prosit predicted peak.
Experimental peak not matched to a Prosit predicted peak.
Prosit predicted peak matched to experimental spectrum.
Prosit predicted peak not matched to experimental spectrum.
VSYTFSQPV 1+ Experimental vs. Synthetic
Spectral Angle on Prosit Matched Peaks: 0.85
Number of Matched Peaks: 91 of 121
Signal To Noise Ratio: inf
VSYTFSQPV 1+ Experimental vs. Prosit
Spectral Angle 0.86
VSYTFSQPV 1+ Synthetic vs. Prosit
Spectral Angle 0.93
TAPKLAEL 2+ Experimental vs. Synthetic
Spectral Angle on Prosit Matched Peaks: 0.89
Number of Matched Peaks: 55 of 137
Signal To Noise Ratio: inf
TAPKLAEL 2+ Experimental vs. Prosit
Spectral Angle 0.91
TAPKLAEL 2+ Synthetic vs. Prosit
Spectral Angle 0.88
KNVIYIHL 2+ Experimental vs. Synthetic
Spectral Angle on Prosit Matched Peaks: 0.78
Number of Matched Peaks: 105 of 338
Signal To Noise Ratio: inf
KNVIYIHL 2+ Experimental vs. Prosit
Spectral Angle 0.81
KNVIYIHL 2+ Synthetic vs. Prosit
Spectral Angle 0.96
VIYIHLESF 2+ Experimental vs. Synthetic
Spectral Angle on Prosit Matched Peaks: 0.84
Number of Matched Peaks: 40 of 112
Signal To Noise Ratio: inf
VIYIHLESF 2+ Experimental vs. Prosit
Spectral Angle 0.83
VIYIHLESF 2+ Synthetic vs. Prosit
Spectral Angle 0.92
VAYGRQVYL 2+ Experimental vs. Synthetic
Spectral Angle on Prosit Matched Peaks: 0.74
Number of Matched Peaks: 74 of 217
Signal To Noise Ratio: inf
VAYGRQVYL 2+ Experimental vs. Prosit
Spectral Angle 0.92
VAYGRQVYL 2+ Synthetic vs. Prosit
Spectral Angle 0.75
Experimental Spectrum
Synthetic Peptide Spectrum
Prosit Predicted Spectrum
Base-Peak Normalized Intensity
m/z

Experimental vs. Synthetic Colour Code:

- Matched peak between spectra. Possible y-, b-, or a-ion.
- Matched peak between spectra. Origin not clear.
- Peak not matched between spectra.

Prosit Comparison Colour Code:

- Experimental peak matched to a Prosit predicted peak.
- Experimental peak not matched to a Prosit predicted peak.
- Prosit predicted peak matched to experimental spectrum.
- Prosit predicted peak not matched to experimental spectrum.

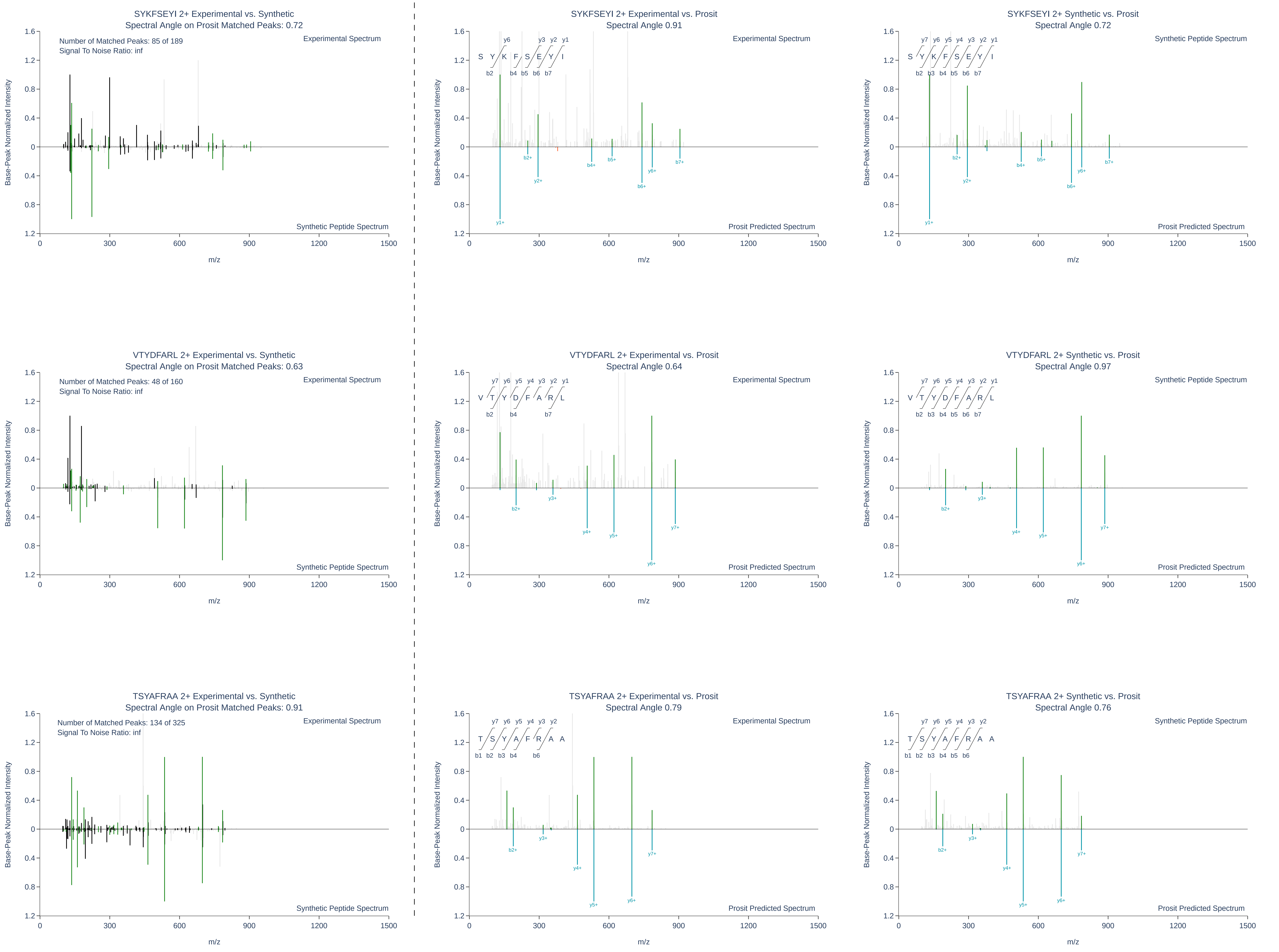

Supplement: File S6 [file EMS204118-supplement-File_S6.pdf]
