# Supplementary material for: PEPSeek-mediated identification of novel epitopes from viral and bacterial pathogens and the impact on host cell immunopeptidomes
Source: Mol Cell Proteomics. Author manuscript; Available in PMC 2025 Aug 8. (PMC12002930; doi:10.1016/j.mcpro.2025.100937)
Supplement: File S10 [file EMS204118-supplement-File_S10.pdf]

PEPSeek-mediated identification of novel epitopes from viral and bacterial pathogens and the impact on host cell immunopeptidomes

$ Correspondence to: e.j.a.m.sijts@uu.nl, michele.mishto@kcl.ac.uk, jliepe@mpinat.mpg.de.

Table S1	Overview of the datasets analysed in this study
Table S2	Synthetic peptides tested for T cell recognition
Table S3	Post-COVID-19 donors
Figure S1	Improvement of computational performance of the inSPIRE upgrade used by PEPSeek
Figure S2	Comparison of PSM identification rates on MHC-I immunopeptidomes using different search engines within the updated inSPIRE platform used by PEPSeek
Figure S3	Comparison of q-value and posterior error estimation using either standard strategies or PEPSeek across MHC-I immunopeptidome datasets
Figure S4	Self peptides in MHC-I immunopeptidomes of either infected or not infected cells identified by applying PEPSeek
Figure S5	Comparison of a putative SARS-COV2-derived peptide identified in the original study and a self peptide identified by PEPSeek in the MHC-I immunopeptidome of infected IHW01070 cells
Figure S6	Network of *C. trachomatis* and *L. monocytogenes* antigens identified in HeLa MHC-I immunopeptidomes by PEPSeek
Figure S7	Qualitative and quantitative analysis of *C. trachomatis* and *L. monocytogenes* antigens identified in HeLa MHC-I immunopeptidomes by PEPSeek

Data Source	Pathogen	Host cell	Host species	(original) search engine	search engines within PEPSeek platform	MHC-I alleles expressed in host
(1)	*L. monocytogenes*	HCT116	Human	PEAKS DB	PEAKS DB	HLA-A*01:01, HLA-A*02:01, HLA-B*18:01, HLA-B*45:01, HLA-C*05:01, HLA-C*07:01
(1)	*L. monocytogenes*	HeLa	Human	PEAKS DB	PEAKS DB	HLA-A*68:02, HLA-B*15:03, HLA-C*12:03
Generated	*L. monocytogenes*	macrophages	mouse	PEAKS DB	PEAKS DB	H-2-Kb
(2)	SARS-CoV-2	HEK293	Human	MaxQuant	MSFragger	HLA-A*02:01, HLA-A*03:01, HLA-B*07:02, HLA-C*07:02
(2)	SARS-CoV-2	Calu-3	Human	MaxQuant	MSFragger	HLA-A*24:02, HLA-A*68:01, HLA-B*07:02, HLA-B*51:01. HLA-C*15:02
(2)	SARS-CoV-2	IHW01070	Human	MaxQuant	MSFragger	HLA-A*01:01, HLA-A*02:01, HLA-B*08:01, HLA-B*13:02, HLA-C*07:01, HLA-C*03:03
(3)	SARS-CoV-2	HEK293	Human	Spectrum Mill	MSFragger	HLA-A*02:01, HLA-B*07:02, HLA-C*07:02
(3)	SARS-CoV-2	A549	Human	Spectrum Mill	MSFragger	HLA-A*25:01, HLA-A*30:01, HLA-B*18:01, HLA-B*44:03, HLA-C*12:03, HLA-C*16:01
Generated	*C. trachomatis*	HeLa	Human	PEAKS DB	PEAKS DB	HLA-A*68:02, HLA-A*03:19, HLA-B*15:03, HLA-C*12:03

Table S1. Overview of the datasets analysed in this study.

Epitope candidate	Peptide sequence	Pathogen	Antigen	Host	Successful synthesis	T cell response in our assays
$InlA_{339-346}$	KNLTYLTL	*L. monocytogenes*	Internalin A	mouse	yes	no
$InlB_{227-235}$	AGLKNLDVL	*L. monocytogenes*	Internalin B	mouse	no	n.a.
$LLO_{296-304}$	VAYGRQVYL	*L. monocytogenes*	Listeriolysin O	mouse	yes	no
$P45_{393-401}$	VGYGRVANF	*L. monocytogenes*	Peptidoglycan lytic protein P45	Mouse	yes	yes
$P45_{314-321}$	TSYAFRAA	*L. monocytogenes*	Peptidoglycan lytic protein P45	mouse	yes	no
$Lmo2677_{239-248}$	AILDFAGHNL	*L. monocytogenes*	Lmo2677 protein	mouse	no	n.a.
$PbpB_{374-381}$	SNVAFAKL	*L. monocytogenes*	PbpB protein	mouse	yes	no
$InlC_{269-277}$	VSYKFSEYI	*L. monocytogenes*	Internalin C	mouse	yes	yes
$InlC_{270-277}$	SYKFSEYI	*L. monocytogenes*	Internalin C	mouse	yes	yes
$InlC_{156-163}$	SLIHLKNL	*L. monocytogenes*	Internalin C	mouse	yes	no
$Lmo0927_{255-262}$	KNVIYIHL	*L. monocytogenes*	Lmo0927 protein	mouse	yes	yes
$Lmo0186_{149-157}$	VNINRAIQL	*L. monocytogenes*	Lmo0186 protein	mouse	yes	no
$InlA_{469-477}$	VSYTFSQPV	*L. monocytogenes*	Internalin A	mouse	yes	no
$ActA_{57-64}$	TGPRYETA	*L. monocytogenes*	Actin assembly-inducing protein	mouse	yes	no
$ActA_{506-513}$	TAPKLAEL	*L. monocytogenes*	Actin assembly-inducing protein	mouse	yes	no
$RL10_{26-33}$	VIVDYRGL	*L. monocytogenes*	50S ribosomal protein L10	mouse	yes	yes
$Lmo0927_{257-265}$	VIYIHLESF	*L. monocytogenes*	Lmo0927 protein	mouse	yes	no
$Lmo0131_{191-198}$	IIRAIVNL	*L. monocytogenes*	Lmo0131 protein	mouse	no	n.a.
$IleS_{116-123}$	SIAEFRKL	*L. monocytogenes*	Isoleucine--tRNA ligase	mouse	yes	no
$PepT_{398-405}$	VIIEVARL	*L. monocytogenes*	Peptidase T	mouse	no	n.a.
$PlcB_{80-88}$	VNHMRANLM	*L. monocytogenes*	Phospholipase C	mouse	no	n.a.
$RL5_{104-111}$	VTVSLPRV	*L. monocytogenes*	50S ribosomal protein L5	mouse	yes	no
$CitC_{393-400}$	VTYDFARL	*L. monocytogenes*	Isocitrate dehydrogenase [NADP]	mouse	yes	no
$Spike_{153-160}$	MESEFRVY	SARS-COV2	spike glycoprotein	human	yes	yes
$R1AB_{7012-7022}$	KPREQIDGYVM	SARS-COV2	Replicase polyprotein 1ab	human	yes	no
NP_{14-27}	RITFGGPSDSTGSN	SARS-COV2	nucleocapsid phosphoprotein	human	yes	yes

NP_{15-22}	ITFGGPSD	SARS-COV2	nucleocapsid phosphoprotein	human	yes	yes
NP_{13-22}	PRITFGGPSD	SARS-COV2	nucleocapsid phosphoprotein	human	yes	yes
NP_{11-22}	NAPRITFGGPSD	SARS-COV2	nucleocapsid phosphoprotein	human	yes	yes
NP_{12-22}	APRITFGGPSD	SARS-COV2	nucleocapsid phosphoprotein	human	yes	yes
NP_{13-20}	PRITFGGP	SARS-COV2	nucleocapsid phosphoprotein	human	yes	yes

Table S2. Synthetic peptides tested for T cell recognition. The peptide position within the antigen is described in the epitope candidate name. The column ‘T cell response in our assays’ reports the outcome of the assay evaluated according to the criteria described in the Results section and the Methods section. The *L. monocytogenes* epitope candidates were identified by PEPSeek in the mouse MHC-I immunopeptidomes. The SARS-COV2-derived epitope candidates are 8 out of 14 epitope candidates only identified by PEPSeek in the human MHC-I immunopeptidomes, and not described as antigenic peptides in literature so far (see Methods for the details of the criteria adopted).

Donor code	COVID-19 status	Age (years)	gender	MHC-I haplotypes
MM-HD-54	1 vaccination, no infection	27 at 1st visit	F	HLA-A*02:01
				HLA-A*03:01
				HLA-B*07:02
				HLA-B*13:02
				HLA-C*06:02
				HLA-C*07:02
MM-HD-54	2 vaccinations, no infection	27 at 1st visit	F	HLA-A*02:01
				HLA-A*03:01
				HLA-B*07:02
				HLA-B*13:02
				HLA-C*06:02
				HLA-C*07:02
MM-HD-54	3 vaccinations, <3 months after infection	27 at 1st visit	F	HLA-A*02:01
				HLA-A*03:01
				HLA-B*07:02
				HLA-B*13:02
				HLA-C*06:02
				HLA-C*07:02
MM-HD-62	No vaccination, <3 months after infection	28 at 1st visit	F	HLA-A*03:01
				HLA-A*31:01
				HLA-B*15:01
				HLA-B*18:01
				HLA-C*04:01
				HLA-C*12:05
MM-HD-50	1 vaccination, no infection	59 at 1st visit	M	HLA-A*01:01
				HLA-A*03:01
				HLA-B*07:02
				HLA-B*08:01
				HLA-C*07:01
				HLA-C*07:02
MM-HD-50	2 vaccinations, no infection	59 at 1st visit	M	HLA-A*01:01
				HLA-A*03:01
				HLA-B*07:02
				HLA-B*08:01
				HLA-C*07:01
				HLA-C*07:02
MM-HD-50	3 vaccinations, <3 months after infection	59 at 1st visit	M	HLA-A*01:01
				HLA-A*03:01

				HLA-B*07:02
				HLA-B*08:01
				HLA-C*07:01
				HLA-C*07:02
IDEA-064	2 vaccinations, <3 months after infection	54 at 1st visit	F	HLA-A*03:01
				HLA-A*30:02
				HLA-B*07:02
				HLA-B*35:01
				HLA-C*04:01
				HLA-C*15:05
IDEA-038	1 vaccination, no infection	46 at 1st visit	F	HLA-A*02:01
				HLA-A*25:01
				HLA-B*18:01
				HLA-B*49:01
				HLA-C*07:02
				HLA-C*12:03
IDEA-038	2 vaccinations, <3 months after infection	46 at 1st visit	F	HLA-A*02:01
				HLA-A*25:01
				HLA-B*18:01
				HLA-B*49:01
				HLA-C*07:02
				HLA-C*12:03
IDEA-038	2 vaccinations, >3 months after infection	46 at 1st visit	F	HLA-A*02:01
				HLA-A*25:01
				HLA-B*18:01
				HLA-B*49:01
				HLA-C*07:02
				HLA-C*12:03
MM-HD-63	3 vaccinations, <3 months after infection	35 at 1st visit	M	HLA-A*02:01
				HLA-A*11:01
				HLA-B*35:01
				HLA-B*44:03
				HLA-C*04:01
MM-HD-16	No vaccination, <6 months after infection	45 at 1st visit	M	HLA-A*24:02
				HLA-A*68:02
				HLA-B*14:01
				HLA-B*51:01
				HLA-C*08:02
				HLA-C*15:06
MM-HD-16	2 vaccinations, >6 months after infection	45 at 1st visit	M	HLA-A*24:02
				HLA-A*68:02
				HLA-B*14:01
				HLA-B*51:01
				HLA-C*08:02
				HLA-C*15:06
MM-HD-16	2 vaccinations, >6 months after infection	45 at 1st visit	M	HLA-A*24:02
				HLA-A*68:02
				HLA-B*14:01
				HLA-B*51:01
				HLA-C*08:02
				HLA-C*15:06
MM-HD-16	3 vaccinations, <3 months after infection	45 at 1st visit	M	HLA-A*24:02
				HLA-A*68:02
				HLA-B*14:01
				HLA-B*51:01
				HLA-C*08:02
				HLA-C*15:06

Table S3. Post-COVID-19 donors. Clinical information for the donors whose PBMCs has been used in the *ex vivo* assays to test the reactivity against the SARS-COV2-derived synthetic epitope candidates reported in **Table S2**.

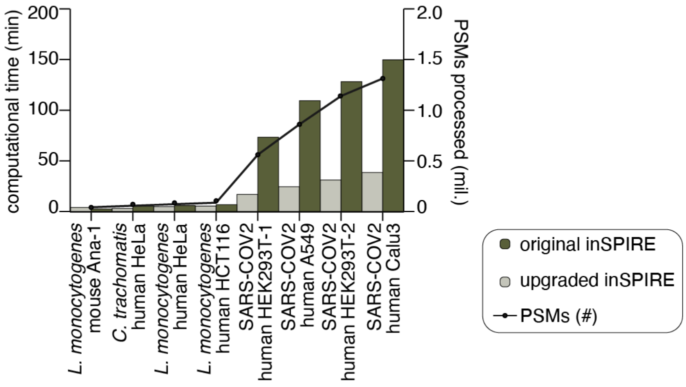

Figure S1. Improvement of computational performance of the inSPIRE upgrade used by PEPSeek. Time taken by the upgrade of inSPIRE software compared to the original version (4) to run the “featureGeneration” pipeline for all datasets in this study alongside the number of PSMs processed per dataset. The inSPIRE upgrade enhances the original algorithm with polars libraries for quicker data handling, upgrades to Python 3.11, and improves parallelization, resulting in a 270% speed increase in feature generation compared to the original inSPIRE in feature generation, and the ability to process over a million PSMs in 30-40 minutes.

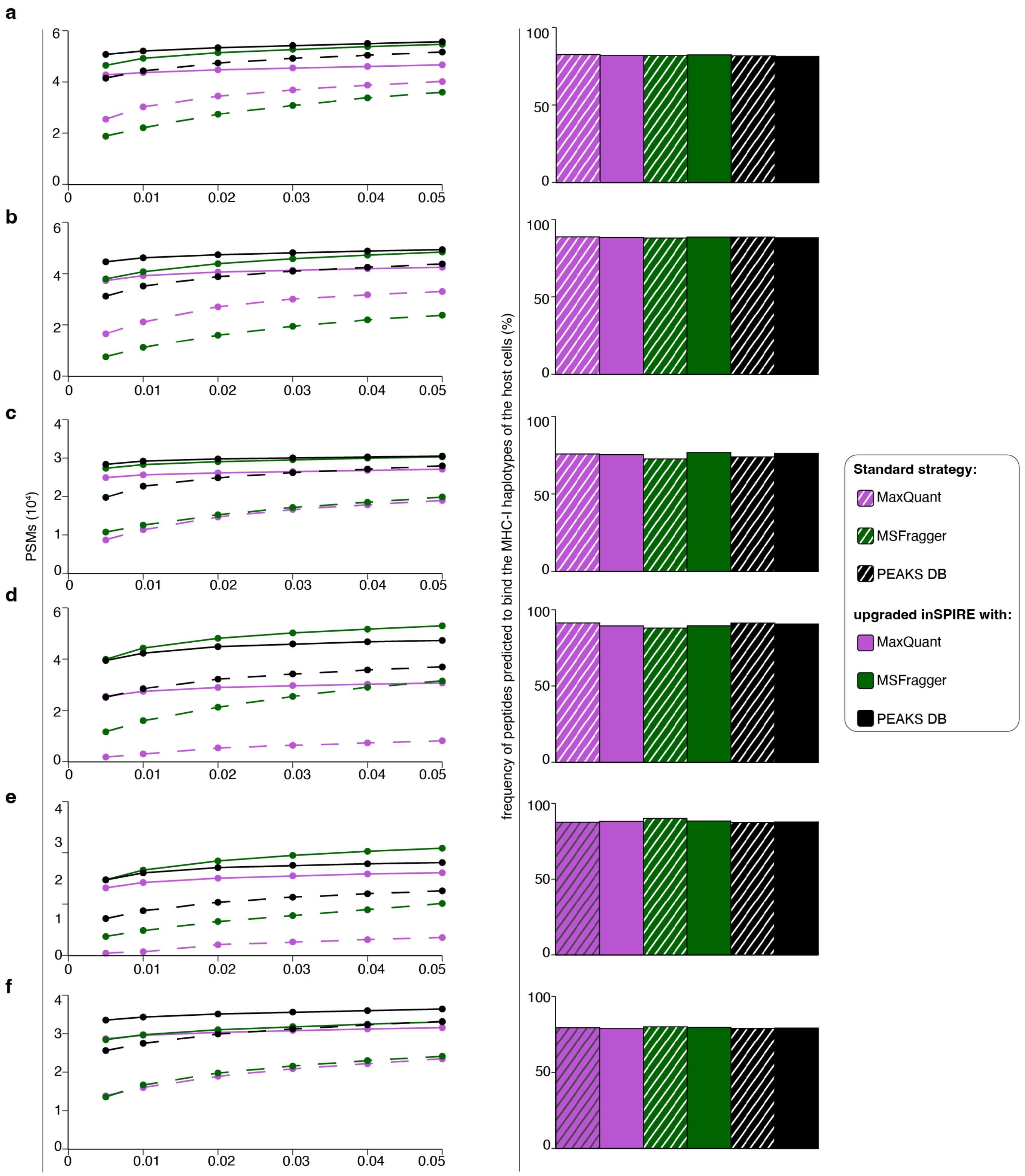

Figure S2. Comparison of PSM identification rates on MHC-I immunopeptidomes using different search engines within the updated inSPIRE platform used by PEPSeek. (**a-f**) Performance defined by the PSM identification rate of three representative search engines within the upgraded inSPIRE platform used by PEPSeek. The analysis with different search engines and rescoring combinations was carried out in all MHC-I immunopeptidomes analyzed in this study with the exception of the ion mobility-based datasets (SARS-COV2-infected Human A549 and HEK293T-2) which could not be analyzed via PEAKS DB with a standard license and were therefore excluded from this comparison. Performance is shown for MaxQuant (open access), MSFragger (open access), and PEAKS DB (commercial) search engines either applied as standard strategy or within the inSPIRE framework applied (see Methods for details). The number of PSMs identified within the FDR range 1-5% is shown on the left panels. This PSM yield indicates the sensitivity of the identification method in separating target PSMs from decoys. As a validation of the quality of the identifications, in the right panels we report the percentage of identified peptides at 1% FDR which

are predicted to bind the MHC-I alleles of the cognate cell host. The prediction of the MHC-I-peptide binding affinity is carried out by NetMHCpan and a cut-off of 2% on the percentage rank predicted binding affinity is used to define a 'binder'. Consistency across search engine/rescoring combinations is an indicator of the stable quality of identifications. Datasets analyzed: *L. monocytogenes*-infected Human HCT116 (**a**), and HeLa (**b**), as well as the mouse Ana-1 (**c**); SARS-COV2-infected Human Calu3 (**d**) and HEK293T-1 (**e**); *C. trachomatis*-infected Human HeLa (**f**).

Briefly, MSFragger generally outperformed MaxQuant but was less effective than PEAKS DB (a commercial software) for canonical peptide identification.

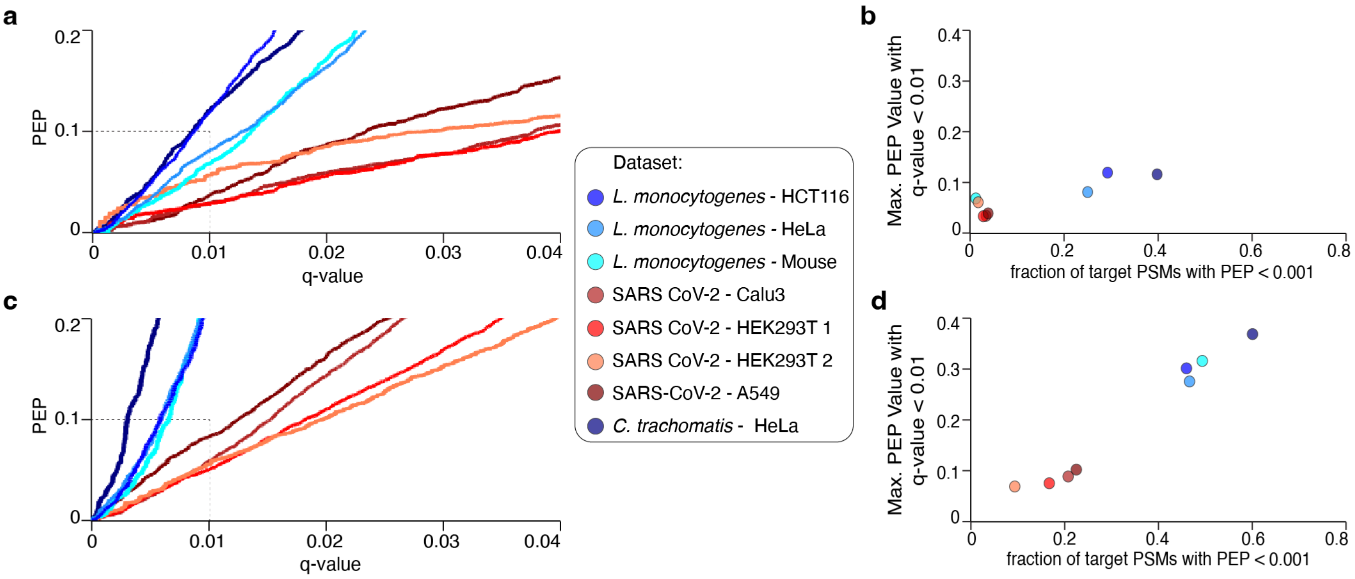

Figure S3. Comparison of q-value and posterior error estimation using either standard strategies or PEPSeek across MHC-I immunopeptidome datasets. (**a-d**) Line plots of posterior error estimates against q-value estimates per PSM (**a,c**), and scatter plot per dataset showing the maximum PEP for a PSM with q-value less than 0.01 against the fraction of target PSMs assigned with PEP less than 0.0001 (**b,d**) by applying either a standard search engine strategy (**a,b**) or PEPSeek (**c,d**). See **Table S1** for details of ethe search strategy applied for each dataset.

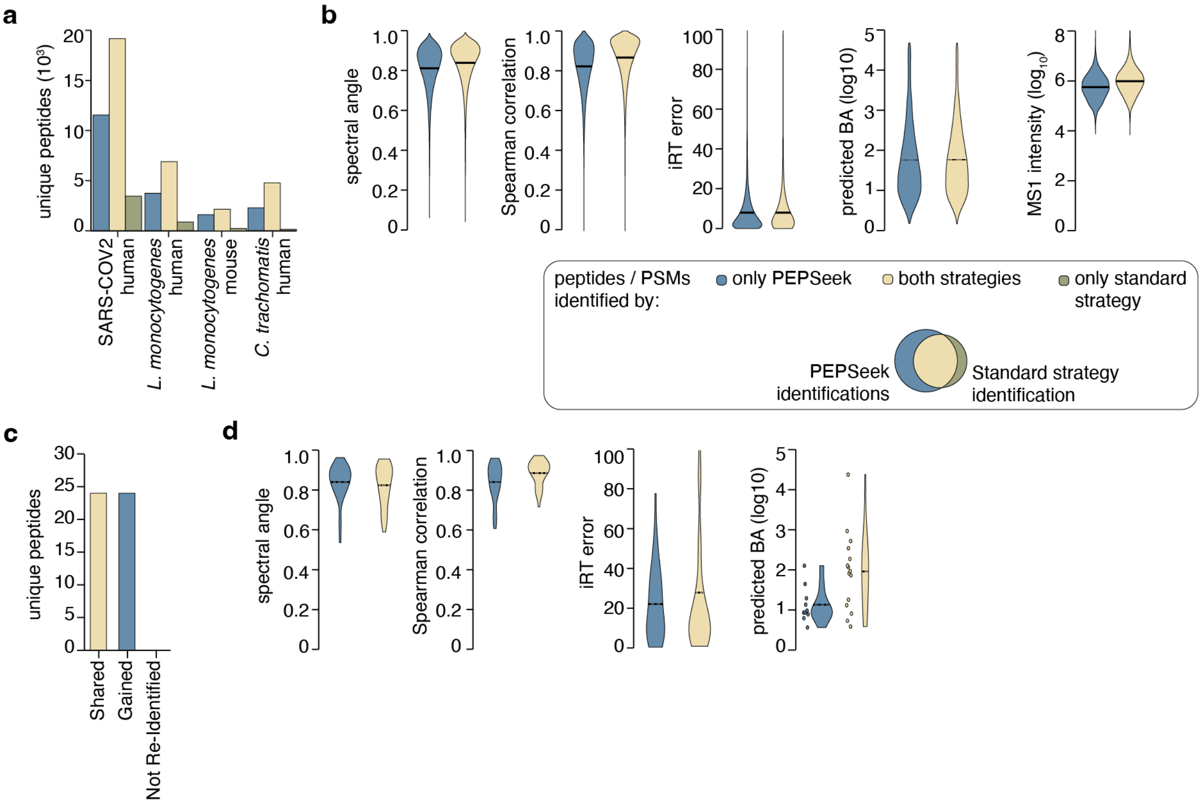

Figure S4. Self-peptides in MHC-I immunopeptidomes of either infected or not infected cells identified by applying PEPSeek. (**a**) The number of self-peptides gained, shared or lost by applying PEPSeek to the MHC-I immunopeptidomes of infected (and uninfected) cells compared to the (original) standard search strategies. (**b**) Distribution of spectral angles, Spearman correlation and iRTs between measured and Prosit-predicted MS2 spectra and MS1 precursors, respectively among the self-peptides identified only by PEPSeek (n = 79,378 PSMs) and those identified by both PEPSeek and the (original) standard search strategies (n = 203,190 PSMs, NetMHCpan predicted binding affinities for length 9 peptides identified only by PEPSeek (n = 9,861 peptides) and those identified by both PEPSeek and the standard strategy (n = 14,382 peptides) and MS2 ion coverage and MS1 precursor's ion intensity of the PSMs of self-peptides identified only by PEPSeek and those identified by both PEPSeek and the (original) standard search strategies. In order to maintain consistency with **Fig. 2**, PEPSeek identifications were carried out based on PEP value less than 0.1 as well as q-value less than 0.01 and identifications from the original studies use the same cut offs used in the identification of pathogen epitope candidates. (**c**) The number of SARS-COV2 peptides gained, shared or lost by applying PEPSeek to the MHC-I immunopeptidomes of infected (and uninfected) cells compared to the preliminary MSFragger search run by PEPSeek when analysing the SARS-COV2 infected immunopeptidome datasets in this study. (**d**) Distribution of spectral angles, Spearman correlation and iRTs between measured and Prosit-predicted MS2 spectra and MS1 precursors, respectively among the SARS-COV2 peptides identified only by PEPSeek (n = 33 PSMs) and those identified by both PEPSeek and the preliminary MSFragger search (n = 87 PSMs) as well as NetMHCpan predicted binding affinities for length 9 peptides identified only by PEPSeek (n = 10 peptides) and those identified by both PEPSeek and the preliminary MSFragger search (n = 15 peptides).

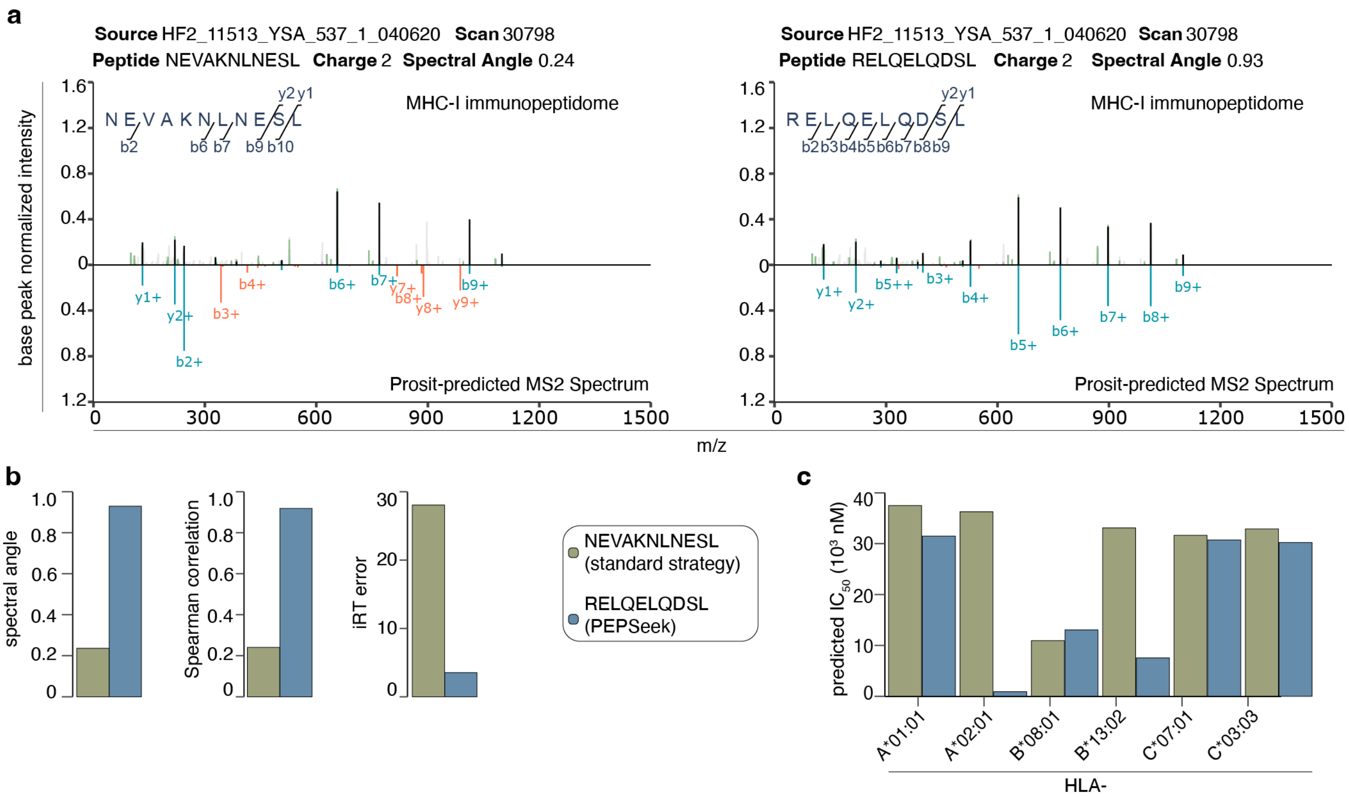

Figure S5. Comparison of a putative SARS-COV2-derived peptide identified in the original study and a self-peptide identified by PEPSeek in the MHC-I immunopeptidome of infected IHW01070 cells. (**a**) Comparison between the measured and the Prosit-predicted MS2 spectra of the putative SARS-COV2-derived peptide NEVAKNLNESL, which was assigned in the original study (2), and the Human TPR-derived peptide RELQELQDSL, which was assigned by PEPSeek. The experimental MS2 spectrum is shown on the positive y-axis. Detected peaks in the MS2 spectrum which are m/z matched to Prosit-predicted peaks of the corresponding peptide are indicated in black. Other potential y-, b-, or a-ions are indicated in green for the putative peptide. Peaks of unknown origin are indicated in grey. The corresponding Prosit predicted MS2 spectra for the cognate peptides are shown on the negative y-axis. Predicted peaks matched to the experimental spectrum are indicated in blue while predicted peaks absent from the experimental MS2 spectrum are indicated in orange. Double charged ions are marked as $^{++}$. Ions' neutral loss of water and of ammonia are symbolized by ^{O} and *, respectively. (**b**) Spectral angle, Spearman correlation and iRT error between measured and Prosit-predicted MS2 spectra and retention times of the 2 peptides sequences shown in (**a**), competing for the same MS2 spectrum and assigned either by the standard strategy in the original study or by PEPSeek. (**c**) Bar chart of NetMHCpan-predicted binding affinity between the MHC-I haplotype the host cell and the 2 peptide sequences shown in (**a**).

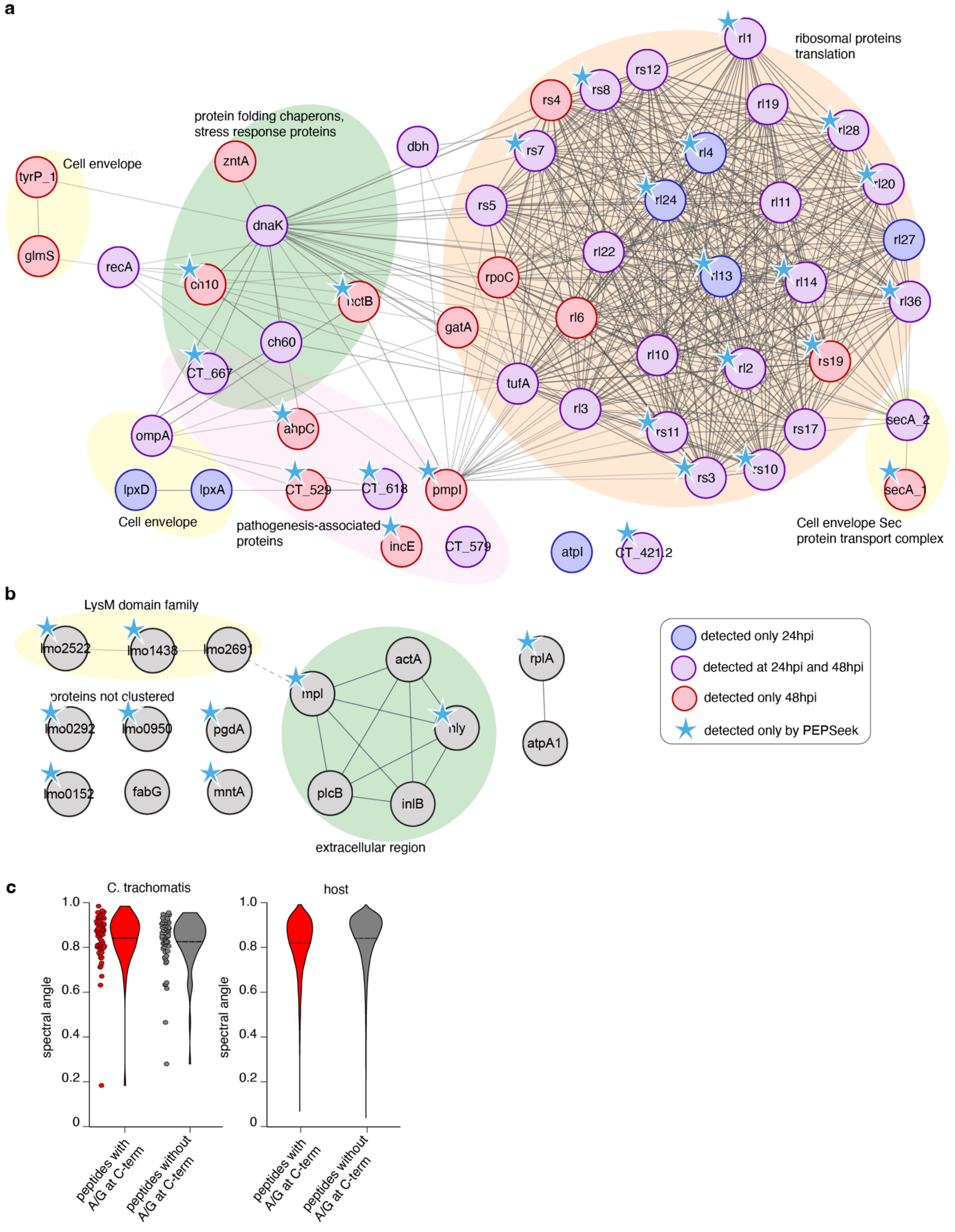

Figure S6. Network of *C. trachomatis* and *L. monocytogenes* antigens identified in HeLa MHC-I immunopeptidomes by PEPSeek. (**a**) String protein interaction network of *C. trachomatis* antigens detected at 24 h (blue), 48 h (red) or at both time points (purple) post infection identified by PEPSeek. Pathogenesis-associated proteins (dashed) include IncE (T3SS), CT_570/copD (T3SS), CT_667 (T3SS), CT_529 (T3SS), CT618 (T3SS), ahpC and pmpl. (**b**) String protein interaction network of *L.*

monocytogenes antigens identified by PEPSeek. (**c**) Distribution of spectral angles between Prosit predicted and experimental MS2 spectra for PSMs used to identify peptides with A/G at the C-terminus compared to those which do not have A/G at the C-terminus for *C. trachomatis* peptides (left) and host peptides (right).

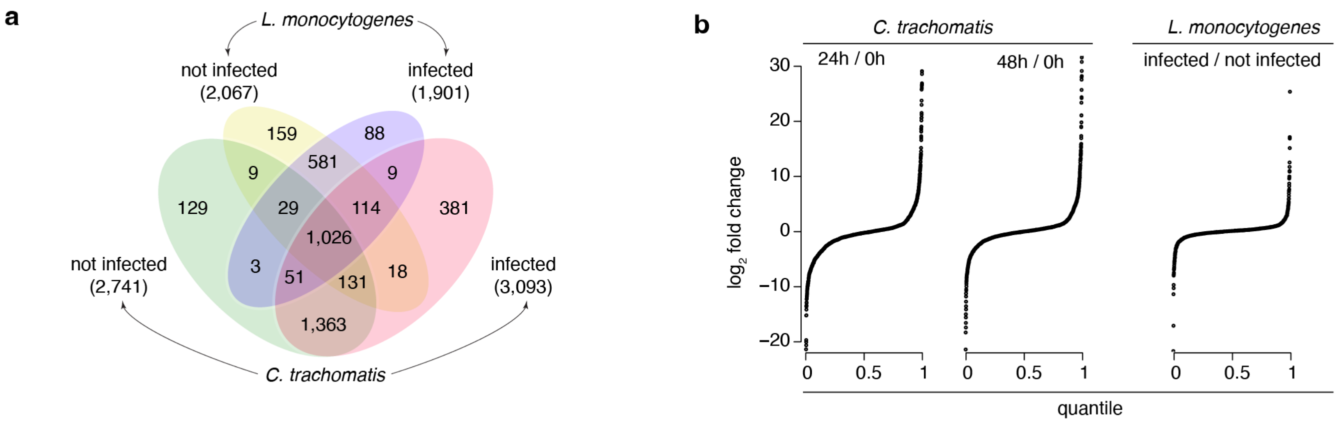

Figure S7. Qualitative and quantitative analysis of *C. trachomatis* and *L. monocytogenes*-induced antigens identified in HeLa MHC-I immunopeptidomes by PEPSeek. (**a**) Venn diagram of self-antigens detected in HeLa cells either infected or not infected with either *C. trachomatis* or *L. monocytogenes*. No significant enrichments were detected for either group. Numbers indicate number of antigens detected. (**b**) Self antigen presentation rate is altered upon infection. Shown are $\log_2$ fold changes of antigen presentability upon *C. trachomatis* or *L. monocytogenes* infection. Antigen presentability was defined as the summed $\log_2$ fold changes of all detected peptides derived from the same antigen.

References

1. Mayer, R. L., Verbeke, R., Asselman, C., Aernout, I., Gul, A., Eggermont, D., Boucher, K., Thery, F., Maia, T. M., Demol, H., Gabriels, R., Martens, L., Becavin, C., De Smedt, S. C., Vandekerckhove, B., Lentacker, I., and Impens, F. (2022) Immunopeptidomics-based design of mRNA vaccine formulations against Listeria monocytogenes. *Nat Commun* 13, 6075

2. Nagler, A., Kalaora, S., Barbolin, C., Gangaev, A., Ketelaars, S. L. C., Alon, M., Pai, J., Benedek, G., Yahalom-Ronen, Y., Erez, N., Greenberg, P., Yagel, G., Peri, A., Levin, Y., Satpathy, A. T., Bar-Haim, E., Paran, N., Kvistborg, P., and Samuels, Y. (2021) Identification of presented SARS-CoV-2 HLA class I and HLA class II peptides using HLA peptidomics. *Cell Rep* 35, 109305

3. Weingarten-Gabbay, S., Klaeger, S., Sarkizova, S., Pearlman, L. R., Chen, D. Y., Gallagher, K. M. E., Bauer, M. R., Taylor, H. B., Dunn, W. A., Tarr, C., Sidney, J., Rachimi, S., Conway, H. L., Katsis, K., Wang, Y., Leistritz-Edwards, D., Durkin, M. R., Tomkins-Tinch, C. H., Finkel, Y., Nachshon, A., Gentili, M., Rivera, K. D., Carulli, I. P., Chea, V. A., Chandrashekar, A., Bozkus, C. C., Carrington, M., Collection, M. C.-., Processing, T., Bhardwaj, N., Barouch, D. H., Sette, A., Maus, M. V., Rice, C. M., Clauser, K. R., Keskin, D. B., Pregibon, D. C., Hacohen, N., Carr, S. A., Abelin, J. G., Saeed, M., and Sabeti, P. C. (2021) Profiling SARS-CoV-2 HLA-I peptidome reveals T cell epitopes from out-of-frame ORFs. *Cell* 184, 3962-3980 e3917

4. Cormican, J. A., Horokhovskyi, Y., Soh, W. T., Mishto, M., and Liepe, J. (2022) inSPIRE: An Open-Source Tool for Increased Mass Spectrometry Identification Rates Using Prosit Spectral Prediction. *Mol Cell Proteomics* 21, 100432
