# Supplementary material for: PEPSeek-mediated identification of novel epitopes from viral and bacterial pathogens and the impact on host cell immunopeptidomes
Source: Mol Cell Proteomics. Author manuscript; Available in PMC 2025 Aug 8. (PMC12002930; doi:10.1016/j.mcpro.2025.100937)
Supplement: File S11 [file EMS204118-supplement-File_S11.pdf]

File S1. All pathogen-derived peptides identified via PEPSeek and the standard search engines. A .xlsx file containing information on the pathogen-derived epitope candidates described in this study. The Overview sheet contains the number of pathogen-derived epitope candidates identified by PEPSeek and standard methods for each MHC-I immunopeptidome described in **Table S1**. The remaining sheets contain details of the peptide sequences identified from each dataset.

File S2. All pathogen-derived proteins identified via PEPSeek and the standard search engines. A .xlsx file containing information on the pathogen antigens represented by the epitope candidates described in this study. The Overview sheet contains the number of the pathogen antigens represented by the epitope candidates identified by PEPSeek and standard methods for each MHC-I immunopeptidome described in **Table S1**. The remaining sheets contain details of the peptide sequences identified from each dataset.

File S3. Pair plots of experimental MS2 spectra and cognate Prosit-predicted MS2 spectra for all pathogen-derived epitope candidates identified in this study. Comparisons between the relevant experimentally measured MS2 spectrum and the cognate Prosit predicted MS2 spectrum for all pathogen epitope candidates identified in this study. For each comparison the experimental spectrum is shown on the positive y-axis. Detected peaks in the MS2 spectra which are m/z matched to Prosit predicted peaks of the corresponding peptide are indicated in black. Other potential y-, b-, or a-ions are indicated in green for the putative peptide. The precursor ion is shown in pink if present. Peaks of unknown origin are indicated in grey. The corresponding Prosit predicted spectrum for the cognate peptide is shown on the negative y-axis. Predicted peaks matched to the experimental spectrum are indicated in blue while predicted peaks absent from the experimental spectrum are indicated in orange. Double charged ions are marked as $^{++}$. Ions' neutral loss of water and of ammonia are symbolized by ^{O} and *, respectively.

File S4. Comparison between MS2 spectra of experimentally identified SARS-COV-2 derived peptides and MS2 spectra of the corresponding synthetic peptides. For all SARS-COV-2 derived peptides which were tested, synthetic peptides were ordered and measured via MS. Presented are the synthetic and experimental MS2 spectra for those 8 peptides. In this case, since the experimental spectra are taken for public datasets, different mass spectrometers and mass spectrometer settings were used between the experimental and the synthetic spectra, which leads to some variation in the measured intensities. We also compare both the experimental and synthetic MS2 spectra to the Prosit predicted MS2 spectrum for greater insight into the specific y- and b- ions present.

File S5. IFN-γ^{+} release by PBMCs of donors with SARS-COV-2 and vaccination history stimulated with a pool of SARS-COV2-derived epitope candidates identified only by PEPSeek. The measured IFN-γ^{+} concentration in PBMC cultures either stimulated and restimulated with synthetic epitope candidates, only stimulated with synthetic epitope candidates or stimulated and restimulated with synthetic random peptides is reported. The donor-peptide match depended on the predicted binding affinity between the peptides and the MHC-I haplotypes of each donor (see **File S1** and Methods for details). In all tabs, that values are mean of 2 technical replicates and we reported both the crude mean and the values upon subtraction of the background IFN-γ^{+} concentration in PBMCs, which was measured in plate's wells stimulated and restimulated with DMSO. In the tab 'single' and 'single-positive', we report the IFN-γ^{+} concentration of the donors who donated blood between 1 and 3 months after a diagnosed and resolved COVID-19 episode. In the tab 'longitudinal', we report the IFN-γ^{+} concentration of PBMCs withdrawn before as well as 7 and 23 weeks after a diagnosed and resolved COVID-19 episode. The synthetic epitope candidates used for the PBMC stimulation (see **Table S2**) are 8 out of 15 that are reported in **File S1**. Values are means of 2 technical replicates.

File S6. Comparison between MS2 spectra of experimentally identified *L. monocytogenes*-derived peptides and MS2 spectra of the corresponding synthetic peptides. For all Listeria derived peptides, synthetic peptides were ordered and measured via MS. Of these 23 peptides, 18 were detected by a PEAKS DB search indicating successful synthesis. Presented are the synthetic and experimental MS2 spectra for those 18 peptides. We also compare both the experimental and synthetic MS2 spectra to the Prosit predicted MS2 spectrum for greater insight into the specific y- and b- ions present.

File S7. Frequency of IFN-γ^{+} CD8^{+} T cells among mouse splenocytes stimulated with the *L. monocytogenes*-derived epitope candidates identified by PEPSeek. The measured frequency of the IFN-γ^{+} CD8^{+} T cells among mouse splenocytes stimulated with the 20 *L. monocytogenes*-derived epitope candidates identified by PEPSeek is reported for each experiment. Each experiment was carried out with splenocytes derived from 4 mice infected with *L. monocytogenes* and 4 mice uninfected. The synthetic peptide sequences are reported in **Table S2**. Values are means of 2 technical replicates. The FACS gating strategy is shown in **Fig. 4d**. The results of the 4 experiments are summarised in **Fig. 4e**. The figures of the 'exp 1' tab are also reported in **Fig. 4f**. P-values comparing the peptides of interest to OVA $_{257\text{-}264}$ are reported for each peptide's stimulation, and were computed by GraphPad Prism version 9 using two way ANOVA and Sidak's multiple comparisons test.

File S8. Gene set enrichment analysis for *C. trachomatis*-derived antigens detected in *C. trachomatis* infected HeLa cells. This table contains all enriched gene sets with false discovery rate (FDR) < 0.01. The column ‘selected’ indicates gene sets displayed in **Fig. 6a**.

File S9. Gene set enrichment analysis for *L. monocytogenes*-derived antigens detected in *L. monocytogenes* infected HeLa cells. This table contains all enriched gene sets with false discovery rate (FDR) < 0.01. The column ‘selected’ indicates gene sets displayed in **Fig. 6b**.
